# Formylation–Decarbonylation Relay Strategy for the Selective Hydrogenation of CO_2_ to CO

**DOI:** 10.1021/acscatal.5c07116

**Published:** 2026-01-23

**Authors:** James Luk, Luke Andrew, Garima Saini, Matthew J. Andrews, Emily Feeke, Aidan P. McKay, David B. Cordes, Michael Bühl, Amit Kumar

**Affiliations:** EaStCHEM, School of Chemistry, 7486 University of St Andrews, North Haugh, KY16 9ST St Andrews, United Kingdom

**Keywords:** Reverse Water Gas Shift, Pincer, Carbon Dioxide, Carbon Monoxide, Hydrogenation

## Abstract

We report here an alternative approach to conducting the reverse water–gas shift (RWGS) reaction catalyzed by a ruthenium pincer complex and an amine. In this strategy, a secondary amine is first formylated by CO_2_ and H_2_ to make H_2_O and formamide. The formamide then undergoes decarbonylation to produce CO with the concomitant regeneration of the amine. Both steps, formylation and decarbonylation, were independently investigated through catalytic optimization studies and DFT computations at the PBE0-D3­(BJ)_PCM(THF)_/def2-TZVP level. Using morpholine and Ru-MACHO pincer catalyst, a TON of 249 was achieved for the hydrogenation of CO_2_ to CO (at 70 bar, 170 °C for 90 h) with 100% selectivity.

## Graphical abstract

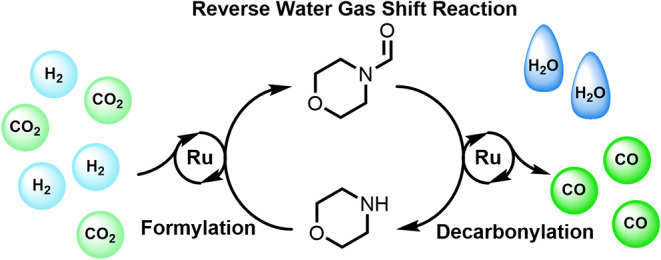

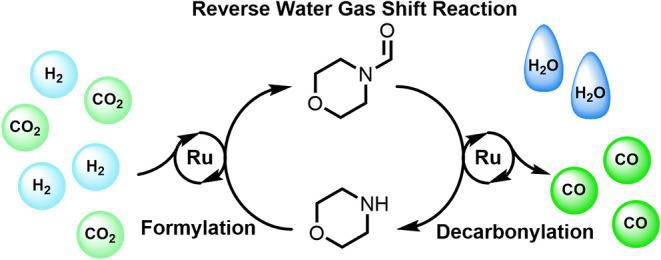

## Introduction

Carbon monoxide (CO) is a ubiquitous C_1_ synthon with prevalent applications across global industries, including bulk chemicals, metallurgy, and polymers.^1–4^ However, industrial CO is predominantly derived from fossil fuel-based steam methane reforming (SMR) and partial oxidation of hydrocarbon or coal, processes that are both energy-intensive and lead to CO_2_ emissions.^5^

The reverse water–gas shift (RWGS) reaction (CO_2_ + H_2_ → CO + H_2_O) presents a promising route to reduce the carbon footprint of CO production by converting CO_2_ into CO using H_2_, releasing water as the only byproduct. By pairing this with green hydrogen, the RWGS reaction offers a carbon-neutral pathway to CO and presents a compelling opportunity to valorize captured CO_2_ on a large scale, thereby improving the economic feasibility of carbon capture and storage (CCS).^6^ However, the RWGS reaction suffers from thermodynamic limitations as it is endothermic (Δ*H*° = +10 kcal/mol) and competes with exothermic methanation (CO_2_ + 4 H_2_ → CH_4_ + 2 H_2_O; Δ*H*° = −39.4 kcal/mol) at lower temperatures (250–500 °C).^6,7^ Most reported RWGS reactions are conducted using heterogeneous catalysts, but these suffer from high energy input requirements (e.g., >600 °C) as well as catalyst deactivation and selectivity challenges.^6,8^ A few homogeneous catalysts have been reported to perform the RWGS reaction at temperatures lower than 160 °C. The first example in this direction was reported by Tominaga et al. in 1994 where a tetranuclear ruthenium cluster H_4_Ru_4_(CO)_12_ in combination with bis­(triphenylphosphine)­iminium chloride ([PPN]­Cl) (5 equiv relative to ruthenium) in NMP solvent was found to exhibit a TON of 23 at 160 °C.^9^ In 2013, the same group reported that the RWGS reaction can be catalyzed by a mononuclear ruthenium complex [PPN]­[RuCl_3_(CO)_3_] in combination with [PPN]Cl (5 equiv relative to ruthenium) to produce CO with a TON of 87 at 160 °C in NMP (Figure 1).^10^ These processes work best in NMP (likely due to the need to stabilize charged organometallic species), which suffers from the issues of toxicity and heavy regulations. Han, Liu, and co-workers recently demonstrated the use of a trinuclear ruthenium cluster Ru_3_(CO)_12_ in combination with an ionic liquid HMimBF_4_ to achieve a TON of 5.3.^11^ The TON for this process was found to be quite low (5–6) although the process could be utilized in cascade catalysis to make other products such as acetic acid. He and co-workers have reported the use of Shvo’s catalyst in combination with LiCl in NMP for the RWGS reaction.^12^ Although a higher TON of 1555 at 160 °C was achieved, the use of a large excess of LiCl (100 equiv) can be problematic as it can be corrosive to the reactor and produces additional waste. Shvo’s catalyst in combination with an ionic liquid [BMIm]Cl has also been reported to catalyze the RWGS reaction, giving TON up to 106 at 140 °C in NMP solvent.^13,14^ These and some other related systems have also been utilized in tandem catalysis to make esters,^14,15^ aldehydes,^16,17^ alcohols,^13,18–24^ carboxylic acids,^25,26^ and alkylated amines.^11,27^ With the issues described above in the reported homogeneously catalyzed RWGS reaction, there is a need to develop new catalytic approaches for RWGS reactions under mild temperatures and avoid the use of corrosive reagents and toxic solvents.

**Figure 1:**
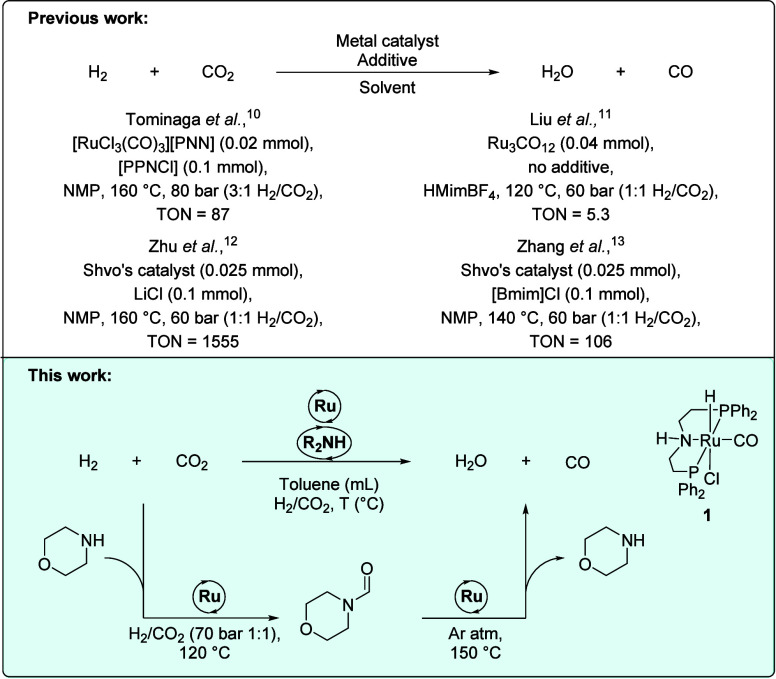
Previously reported conditions and corresponding TONs for the RWGS reaction (top) and the approach reported herein.

*N*-Formylation of amines using CO_2_ and H_2_ to make formamides has been reported in the presence of pincer catalysts of ruthenium,^25,26^ manganese,^27^ iron,^28^ and cobalt.^29^ In particular, Ru-MACHO-type pincer catalysts have been demonstrated to be highly active for this process showing TONs up to 1.9 M^26^ in a single batch, as well as in a pilot plant.^30^ We envisioned that coupling this process with the tandem decarbonylation of formamides could allow us to achieve the RWGS reaction under mild conditions. Building on this strategy, we report here a fundamentally new approach to performing the RWGS reaction assisted by an amine. In this approach, an amine is first formylated by its reaction with CO_2_ and H_2_ to make a formamide and water. The subsequent decarbonylation of formamide produces CO and regenerates the amine. The use of amines in the RWGS reaction, as proposed here, offers the potential for seamless integration with CO_2_ capture processes that also employ amines as absorbents. This would be analogous to a strategy reported by Prakash, where CO_2_ capture by amines was directly coupled with subsequent hydrogenation to methanol using Ru-MACHO pincer catalysts.^31^

## Results and Discussion

To achieve the proposed amine-assisted RWGS reaction in one pot, it was important to find an amine for which the formylation and subsequent decarbonylation steps could be achieved under the same reaction conditions. Previous reports using transition-metal pincer catalysts suggest primary amines to be the most suitable candidates for the *N*-formylation of amines.^25–29^ However, we recently discovered that primary formamides can undergo simultaneous dehydrogenation (to make isocyanates) and decarbonylation, forming urea derivatives that would be undesired for the envisioned RWGS reaction.^32^ In order to achieve the proposed RWGS reaction, we focused our study on secondary amines for which the dehydrogenation of the corresponding formamides is not possible. To get an initial idea of selecting secondary amines, we calculated the thermodynamics of the corresponding *N*-formylation and decarbonylation steps. As shown in Table 1, the formylation of the mixed aromatic–aliphatic secondary amine, *N*-methylaniline (entry 1), is endergonic, whereas that of the aliphatic secondary amine dimethylamine (entry 2) is exergonic. The reactions involving cyclic amines (entries 4 and 5) are slightly less exergonic than those of dimethylamine. In contrast, the reaction for decarbonylation showed all substrates to be endergonic with the mixed aromatic aliphatic amine (entry 1) being the least endergonic followed by cyclic amines (entries 3 and 5) and dimethylamine (entry 2), which were significantly more endergonic.

**Table 1:**
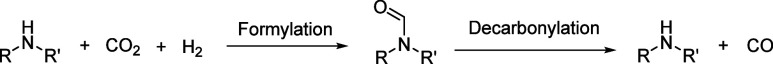

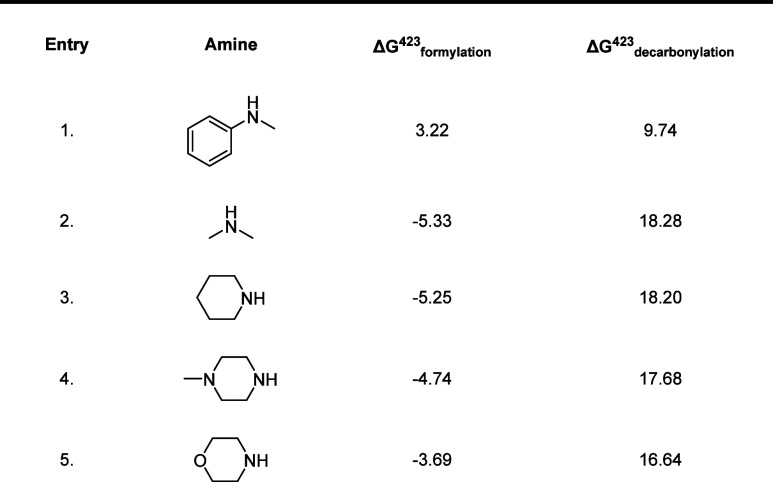
Thermodynamics for the Formylation and Decarbonylation^a^

a PBE0-D3­(BJ)_PCM(THF)_/def2-TZVP//RI-BP86_PCM(THF)_/def2-SVP level. Δ*G* values are in kcal mol^–1^.

Based on the thermodynamic predictions made by DFT computations, we studied the formylation of a few secondary amines using conditions adapted from the report by Ding et al. on the *N*-formylation of amines.^26^ As shown in Figure 2, heterocyclic amines such as morpholine, piperazine, and piperidine derivatives showed excellent conversion to the corresponding formamides (**1**–**5**). In contrast, dialkylamines, dibutylamine, and dibenzylamine achieved moderate conversion to the corresponding formamides (**6**, **7**), whereas no conversion was observed for aniline derivatives (**8**–**11**), likely due to the poor nucleophilicity of amines consistent with positive Δ*G* as mentioned above (Table 1). In all reactions, the products were formed without the observation of formic acid, consistent with previous reports of *N*-formylation of amines disclosing the necessity of amines to regenerate the catalyst from it’s corresponding formate species.^31^ Based on this study, *N*-formylmorpholine was selected as a model substrate for the next step, i.e., decarbonylation of formamide.

**Figure 2:**
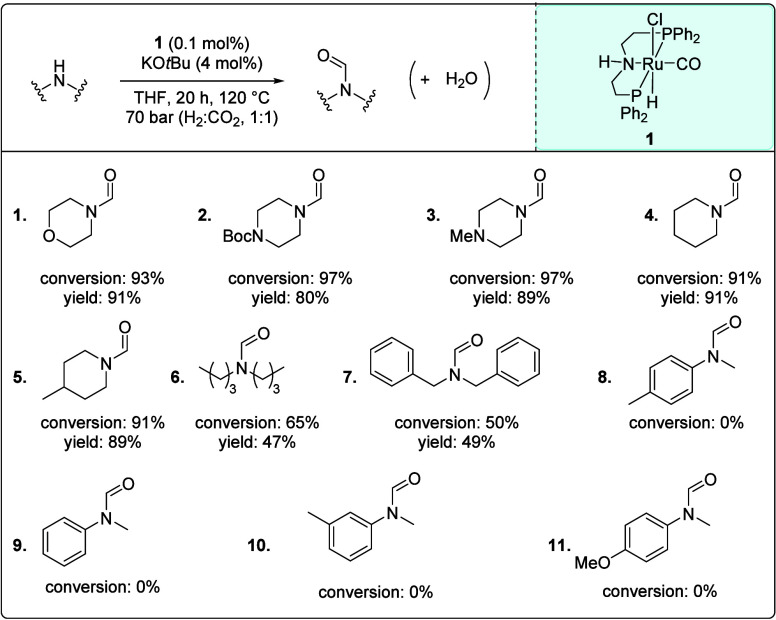
Formylation of secondary amines to secondary formamides. Standard reaction conditions: Amine (10 mmol), **1** (0.1 mol %), KO*t*Bu (4 mol %), THF (1 mL), 20 h, 120 °C (oil bath), 70 bar (H_2_:CO_2_ = 1:1). Yields were determined by ^1^H NMR spectroscopy using 1,3,5-trimethoxybenzene as an internal standard.

Catalytic decarbonylation of *N*-formylmorpholine was initially studied by using complex **1** and KO*t*Bu under various conditions. Refluxing a THF solution of *N*-formylmorpholine in a sealed flask at 120 °C for 18 h in the presence of 1 mol % complex **1** and 5 mol % KO*t*Bu led to 15% conversion of *N*-formylmorpholine to morpholine (13%) and CO (13%, Table 2, entry 1). A small amount of H_2_ and CO_2_ gas (total: 4%) was also observed by the GC-TCD (Gas Chromatography–Thermal Conductivity Detector), likely due to the reaction of the formed CO with a trace amount of water present in the reaction mixture through the water gas shift reaction. Indeed, heating water (1 mL, 150 °C oil bath) in a sealed J-Young’s flask in the presence of 0.01 mmol of complex **1** and 0.04 mmol of KO*t*Bu in toluene (2 mL) under 1 bar of CO atmosphere produced CO_2_ and H_2_ (TON ∼ 420), confirming that indeed complex **1** can promote the water gas shift reaction under these reaction conditions.

**Table 2:**
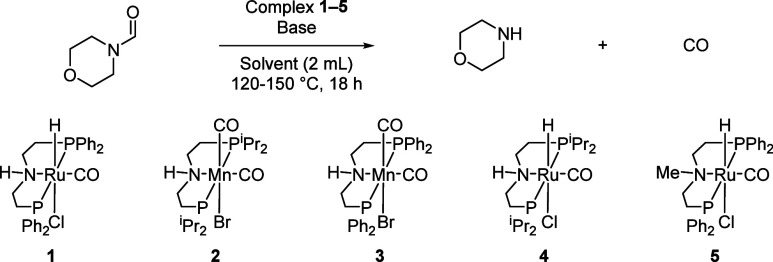
Optimization Summary for Decarbonylation of N-Formylmorpholine^a^

Entry	Precatalyst (mol %)	Base (mol %)	Solvent	*T* (°C)	Formamide Conversion (%)	Amine Yield (%)	CO Yield/Selectivity (%)
1	**1** (1)	KO*t*Bu (5)	THF	120	15	13	13/96
2	**1** (1)	KO*t*Bu (5)	Toluene	120	19	14	14/97
3	**1** (1)	KO*t*Bu (5)	Anisole	120	7	6	4/87
4	**1** (1)	KO*t*Bu (5)	Toluene	150	23	20	15/86
5	**1** (1)	KO*t*Bu (10)	Toluene	150	32	25	16/90
6	**1** (1)	KO*t*Bu (20)	Toluene	150	45	32	18/96
7	**1** (1)	NaO*t*Bu (10)	Toluene	150	26	17	18/85
8	**1** (1)	K_2_CO_3_ (10)	Toluene	150	40	28	28/89
9	**1** (1)	Cs_2_CO_3_ (10)	Toluene	150	41	23	23/98
10	**1** (1)	K_2_CO_3_ (20)	Toluene	150	42	27	27/93
11	**2** (1)	K_2_CO_3_ (10)	Toluene	150	5	n.o.	2/76
12	**3** (1)	K_2_CO_3_ (10)	Toluene	150	12	n.o.	2/89
13	**4** (1)	K_2_CO_3_ (10)	Toluene	150	8	5	3/84
14	**5** (1)	K_2_CO_3_ (10)	Toluene	150	16	15	4/75
15	**1** (2)	K_2_CO_3_ (10)	Toluene	150	64	51	48/83
16^b^	**1** (1)	K_2_CO_3_ (10)	Toluene	150	0	0	

a Standard reaction conditions: *N*-formylmorpholine (0.86 mmol), precatalyst (1–2 mol %), base (5–20 mol %), solvent (2 mL), 18 h, 150 °C (oil bath). Amine yields were determined by ^1^H NMR spectroscopy using 1,3,5-trimethoxybenzene as an internal standard. CO yield was determined by analyzing reaction headspace by GC-TCD, while selectivity was determined as the total CO yield over the sum of all the detectable gases. n.o. stands for not observed.

b Reaction was carried out under 1 bar CO atmosphere.

A similar yield (14%) and selectivity (97%) of CO were observed in toluene (entry 2), whereas a much lower yield (4%) was observed when anisole was used as the solvent (entry 3). Increasing the temperature and base loading showed a slight increment in the conversion and yield of the decarbonylation process (entries 4–6). Increasing KO*t*Bu loading to 10% and 20% (entries 5, 6) or using NaO*t*Bu (10%, entry 7) showed similar CO yield (16–18%). K_2_CO_3_ and Cs_2_CO_3_ (entries 8 and 9, respectively) both increased the yield of decarbonylation with K_2_CO_3_ being the most effective, producing CO in 28% yield. Similar to KO*t*Bu, upon increasing the loading of K_2_CO_3_ to 20 mol %, no significant increase in CO yield was observed (entry 10, in comparison to entry 8). Performing the reaction in the presence of other MACHO complexes of manganese and ruthenium (**2**–**5**, entries 11–14) showed a negligible yield of CO. These experiments suggest that the most effective conditions for the decarbonylation of *N*-formylmorpholine are **1** (1 mol %), K_2_CO_3_ (10 mol %), toluene (2 mL), and 150 °C (entry 8).

Using the optimized reaction conditions (Table 2, entry 8), we studied the decarbonylation of various formamide substrates, in particular those prepared in Figure 2, with the aim of finding a suitable amine for which both formylation and subsequent decarbonylation steps could be achieved. As shown in Figure 3, *N*-formylmorpholine, *N*-formylpiperidine, *N*-formyl-4-methylpiperazine, and *N*-formyl-4-methylpiperidine were decarbonylated to produce the corresponding amines and CO in 24–33% yields (entries 1–4). *N*-Formyl-*N*-methyl aniline (entry 5) showed a slightly higher yield of amine (37%) and CO (34%), whereas dibutyl- and dibenzyl-formamides showed yields of amines and CO in the range of 19–28% (entries 6–9). In all of these cases, the conversion of formamide was similar to the yield of the corresponding amine (see Table S3).

**Figure 3:**
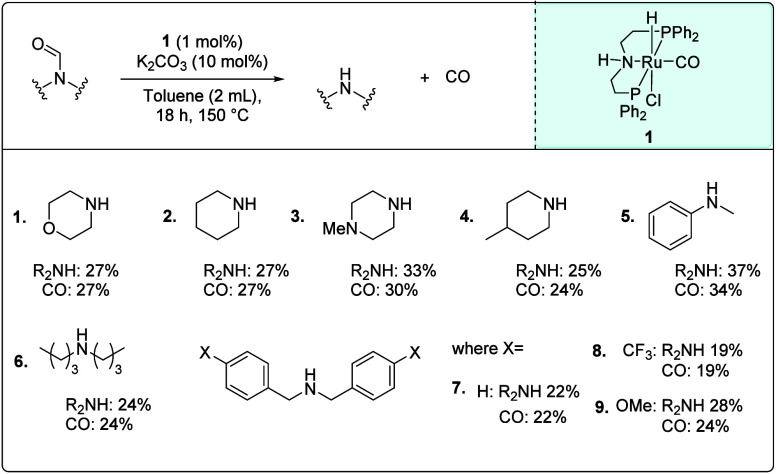
Decarbonylation of various formamides. Reaction conditions: **1** (1 mol %), K_2_CO_3_ (10 mol %), toluene (2 mL), 18 h, 150 °C. Amine yields were determined by ^1^H NMR spectroscopy using 1,3,5-trimethoxybenzene as an internal standard. CO yields were determined by analyzing the headspace by GC-TCD, while the selectivity was determined as the total CO yield over the sum of all the detectable gases.

Having studied the reaction conditions for the formylation and decarbonylation, we paid attention to understanding the reaction mechanism, focusing on the decarbonylation process. The optimization studies (Table 2, entries 1–14) and substrate scope (Figure 3) for the decarbonylation of formamides show that the yield of CO lies between 4% and 28% despite changing various reaction conditions. A possible reason for this could be the deactivation of catalyst in the presence of an excess of CO. Indeed, doubling the amount of complex **1** to 2 mol % and keeping the remaining conditions the same doubled the conversion of *N*-formylmorpholine (64%) to morpholine (51%) and CO (48%, entry 15, Table 2). Additionally, when the decarbonylation reaction was carried out under an atmosphere of CO (1 bar), a quantitative recovery of *N*-formylmorpholine was observed (Table 2, entry 16), confirming that the presence of CO disfavors the decarbonylation reaction.

Another possibility for the lower decarbonylation yield could be the reversibility of the decarbonylation reaction, i.e., the carbonylation of amines to form formamides under the reaction conditions. Indeed, refluxing morpholine in toluene in the presence of 1 bar of CO, 1 mol % complex **1**, and 4 mol % KO*t*Bu led to the formation of *N*-formylmorpholine in 5% yield. Carbonylation of amines to make formamides has been reported to be catalyzed by various transition metals or bases in the past.^33–37^ These results suggest that indeed carbonylation and decarbonylation reactions are reversible under the reaction conditions.

To further probe into the reversibility, the decarbonylation of *N*-formylmorpholine was studied under varying concentrations of *N*-formylmorpholine, keeping the amount of complex **1A** (0.01 mmol), K_2_CO_3_ (0.1 mmol), and toluene (2 mL) the same. Interestingly, increasing the concentration of *N*-formylmorpholine led to an approximately linear relationship between the TON and *N*-formylmorpholine concentration (Figure 4). This is also suggestive of the reversibility of decarbonylation and carbonylation steps, where the yield of decarbonylation can be pushed by increasing the concentration of formamide.

**Figure 4:**
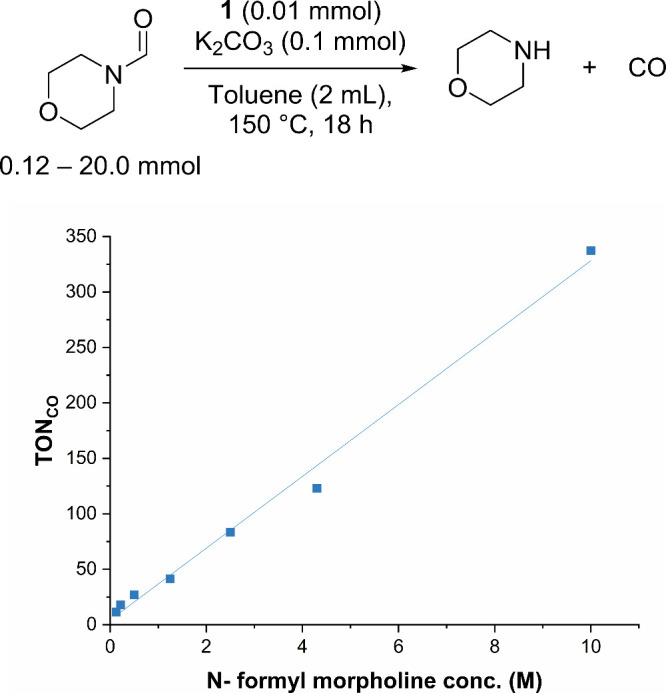
Graph of TON for catalytic decarbonylation vs initial concentration of *N*-formylmorpholine, where TON_CO_ refers to the TON of CO wrt complex **1**.

We speculate that the formed CO could bind with the active species **1A** to form the ruthenium dicarbonyl complex **1D** (Figure 5A), possibly slowing the catalysis. Indeed, charging a J-Young’s NMR tube containing **1** (0.01 mmol), KO*t*Bu (0.02 mmol), and toluene-*d*_8_ (∼0.5 mL) under an atmosphere of CO and allowing the mixture to react at 60 °C (18 h) resulted in the quantitative formation of the dicarbonyl complex **1D** (hydride: ^1^H −6.02 ppm, t, *J*_HP_ = 17.5 Hz), as also reported in the past.^32,38^ Replacing the NMR tube’s atmosphere with Ar and heating overnight (80 °C) resulted in no observable change of organometallic species. However, the addition of *N*-formylmorpholine (0.086 mmol, ∼9 equiv relative to complex **1**) and heating for 18 h (150 °C) resulted in full conversion of the dicarbonyl complex to a mixture of unidentified organometallic species, while *N*-formylmorpholine was found to be converted (30% conversion) to morpholine (27% yield). These results suggest that dicarbonyl complex **1D** can be converted into a catalytically active species in the presence of excess formamides, whereas excess CO leads to catalyst inhibition (Table 2, entry 16).

**Figure 5:**
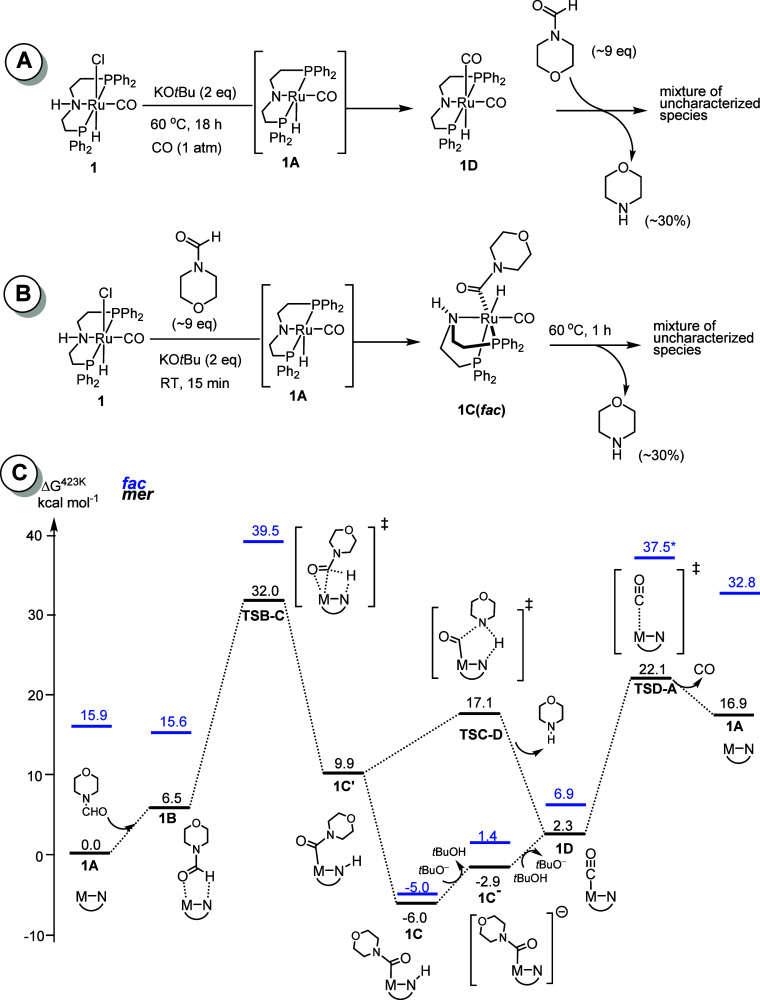
(A) Formation of ruthenium dicarbonyl complex **1D** and its reactivity with *N*-formylmorpholine. (B) Formation of complex **1C** upon reaction of **1** with *N*-formylmorpholine and its reactivity upon heating (1 h, 60 °C). (C) Energy profile showing important mechanistic steps in the decarbonylation of *N*-formylmorpholine using an activated Ru-MACHO catalyst (PBE0-D3­(BJ)_PCM(THF)_/def2-TZVP//RI-BP86_PCM(THF)_/def2-SVP level). Black data *mer*, blue data *fac* isomers (* denotes approximate value; see ESI).

The addition of *N*-formylmorpholine (0.086 mmol) to **1** (0.01 mmol) and KO*t*Bu (0.02 mmol) at room temperature showed nearly quantitative conversion of **1** to a new complex in 15 min. The ^1^H NMR (Figures S52 and S52A) spectrum of the formed complex showed the appearance of a doublet of doublets centered at δ −5.64 ppm with *J*_HP_ = 94 Hz and 26 Hz, suggesting that the hydride is coupling with two different phosphorus atoms, possibly one in *trans* and the other in the *cis* geometry. Indeed, running a ^1^H­{^31^P} NMR spectrum (Figure S54) transformed this doublet of doublets into a singlet at δ −5.64 ppm. The ^31^P­{1H} NMR spectrum showed two doublets at δ 49.92 and 48.72 ppm with *J*_PP_ = 11 Hz, characteristic of *cis* phosphorus atoms bound to ruthenium. A ^1^H, ^31^P HMBC NMR spectrum (Figure S55) showed that the hydride signal couples with only one of the ^31^P signals, δ 48.72 ppm, likely as it is *cis* to the hydride whereas the ^31^P signal at δ 49.92 ppm does not couple with the hydride, presumably as it is *trans* to the hydride. These NMR spectra suggest that the formed new complex has *facial* geometry instead of the *meridional* geometry as present in starting complex **1**. The structure of the new complex was further confirmed by a single crystal X-ray diffraction (Figure 6) and the complex was characterized to be **1C (fac)** where ruthenium was bound to a PNP ligand in facial coordination geometry, a hydride, a CO, and a formylmorpholine ligand. Hydrogen bonding between the N–*H* proton and C=*O* of *N*-formylmorpholine was also observed in the X-ray structure (Figure 6). Heating the reaction mixture for 1 h at 60 °C after the formation of complex **1C** led to the decarbonylation of *N*-formylmorpholine to morpholine (Figure 5B).

**Figure 6:**
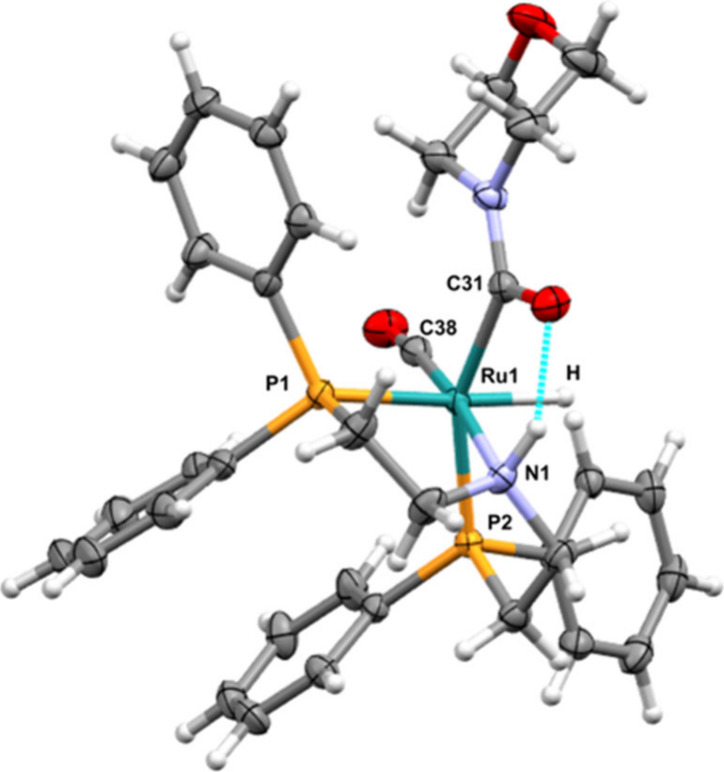
X-ray crystal structure of **1C (fac)** with thermal ellipsoids drawn at the 50% probability level. Selected bond distances (Å) and angles (°): Ru1–H 1.63(3), Ru1–P1 2.3388(5), Ru1–P2 2.3037(5), Ru1–N1 2.1913(17), Ru1–C31 2.137(2), Ru1–C38 1.837(2), P1–Ru1–H 164.3(11), P2–Ru1–H 81.0(11), P2–Ru1–P1 108.278(19), N–Ru1–H 86.8(11), N–Ru1–P1 82.21(5), and N–Ru1–P2 81.49(5).

To get further insight into the reaction mechanism, we computed a DFT pathway for the decarbonylation of *N*-formylmorpholine using complex **1A**, as shown in Figure 5C. Initially, we modeled the reaction steps for the meridional isomer of the pincer ligand (black lines in Figure 5C). The reaction proceeds through the formation of a Ru-(CONR_2_) intermediate (**1C**) that, after rearrangement to a higher-lying intermediate **1C′**, eliminates amine via metal–ligand cooperation, forming the ruthenium dicarbonyl complex **1D**. An alternative pathway via direct CO elimination from **1C** affording a Ru-amido intermediate can be excluded because the corresponding transition state (**TSC–CO**, not shown) is much higher in energy at Δ*G* ^423^ = 38.3 kcal mol^–1^ above **1C′**. A base-assisted mechanism is also possible for this step (modeled through deprotonation of **1C′** by *t*BuO^–^ affording an anionic intermediate **1C** ^**–**^; see Figure 5C), presumably with low kinetic hindrance. It is noteworthy that Δ*G* ^423^ for the reaction of complex **1A** + *N*-formylmorpholine → Complex **1D** + morpholine is 16.9 kcal mol^–1^. Furthermore, the release of CO from complex **1D** to regenerate complex **1A** was found to be uphill by 14.6 kcal mol^–1^, supporting the hypothesis that an increase in the CO pressure could result in a slower reaction rate by slowing the rate of CO release.

Following the discovery that intermediate **1C** adopts a facial rather than a meridional conformation of the MACHO ligand, we recalculated the key steps for the corresponding *fac* isomers (blue data in Figure 5C). Surprisingly, *fac*-**1C** is computed to be slightly less stable than *mer*-**1C**, in apparent contrast to its observation in solution and in the solid state, but the difference is only 1 kcal mol^–1^, arguably within the accuracy of DFT. For all other minima and those transition states that could be located, *fac* isomers are computed well above their *mer* analogues, suggesting that, while *fac*-**1C** can be isolated at room temperature, the actual catalytic turnover may proceed via the *mer* isomers because of the lower barriers on that path; for instance, the barrier for CO loss from *fac*-**1C** via *fac*-**TSD-A** is indicated to exceed 40 kcal mol^–1^ and that from *mer*-**1C** via *mer*-**TSD-A** is only Δ*G* ^‡^ = 28.1 kcal mol^–1^ (see Figure 5C). This argument is based on the assumption that facile interconversion between *fac* and *mer* isomers will only be possible at the stage of five-coordinated complex **1A**. It should be noted that the computed barrier for the formation of *fac*-**1C** via *fac*-**TSB-C** is too high to be overcome at room temperature (39.5 kcal mol^–1^ at 423 K, cf. Figure 5C, 35.9 at RT). There must be other routes to *fac*-**1C**, either via other pathways with lower barriers (for a selection of possible intermediates that might be involved, see Figure S185) or other mechanisms (such as tunneling).^39^ Detailed study of these possibilities would be interesting but is, at this point, deemed outside the scope of this paper.

Based on these observations, we propose a summary of the reaction pathway as outlined in Figure 7, involving two catalytic cycles: Cycle A for the formylation of amines and Cycle B for the decarbonylation to regenerate amine. Based on previous reports,^31,40^ it is likely that the reaction starts with the formation of a dihydride complex **1F** from the reaction of the amido-complex **1A** with H_2_. Insertion of CO_2_ to the ruthenium dihydride complex **1F** leads to the formation of the ruthenium formyl species **1E**, which reacts with an amine to form a carbamate salt. The dehydration of this carbamate salt leads to the formation of a formamide intermediate, which enters the second catalytic cycle, reacting with ruthenium amido complex **1A** to form Ru­(CONR_2_) species **1C**. Release of an amine from this complex leads to the formation of ruthenium dicarbonyl species **1D**, which can eliminate CO in the presence of a formamide (Figure 5B) under the reaction condition to regenerate the amido complex **1A**. Among these main reactions, two side reactions can also occur, which can inhibit the overall process: (a) water gas shift reaction to produce CO_2_ from the reaction of CO and H_2_O and (b) carbonylation of amines to make formamides.

**Figure 7:**
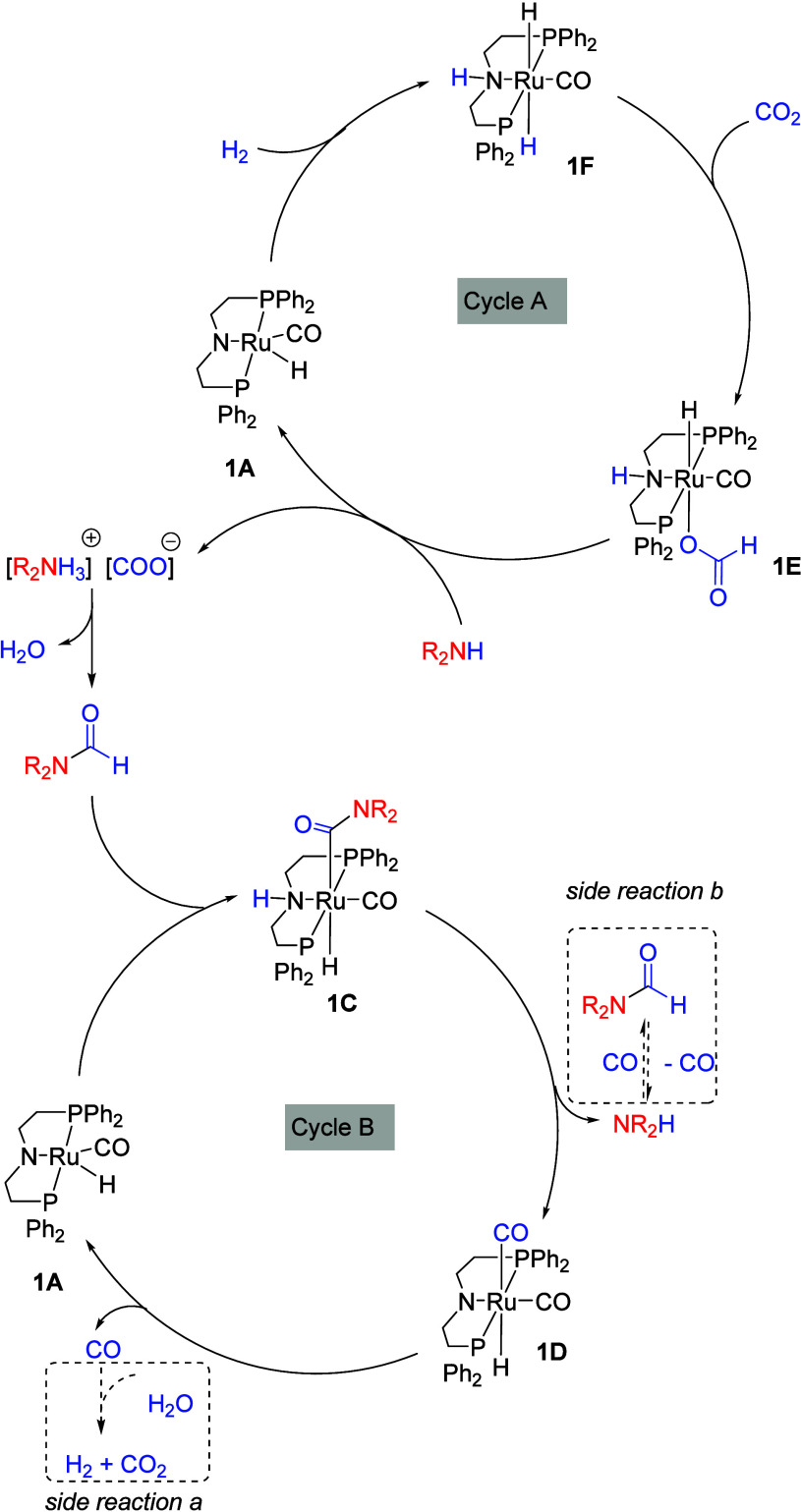
Proposed catalytic cycle for the amine-assisted RWGS reaction catalyzed by the Ru-MACHO complex **1** in the presence of base.

Having independently elucidated the formylation and decarbonylation steps, as well as the overall reaction mechanism, we next turned our attention to achieving the RWGS reaction in one-pot. Initially, morpholine (10 mmol) was heated in toluene (2 mL) in the presence of complex **1** (0.01 mmol) and K_2_CO_3_ (0.1 mmol) at 120 °C under 70 bar total pressure (H_2_:CO_2_ = 1:1) for 18 h to accomplish the formylation step. The reactor was then depressurized by releasing H_2_ and CO_2_, purged with argon for 30 min, and subsequently held at 150 °C for an additional 18 h to promote decarbonylation. However, this overall process resulted in only negligible CO formation (TON = 2), while *N*-formylmorpholine was detected in 82% yield in the final reaction mixture (Figure 8).

**Figure 8:**
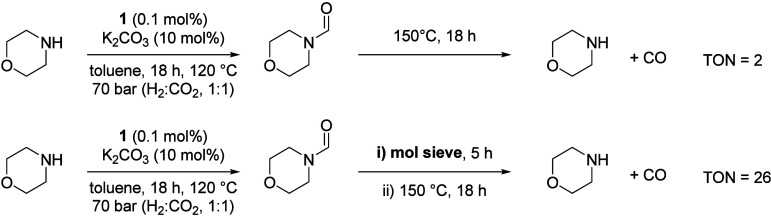
Sequential two-step-one-pot formylation/decarbonylation to achieve overall RWGS reaction.

We speculate that the low CO yield arises from the reaction of CO with H_2_O generated during the formylation step, producing CO_2_ and H_2_ via the water–gas shift reaction. Consistent with this hypothesis, substantial amounts of H_2_ and CO_2_ (∼3.4 mmol combined) were observed. We therefore hypothesized that removal of the water byproduct using 3 Å molecular sieves could drive the RWGS reaction more effectively.

Accordingly, the formylation of morpholine was first carried out using the Ru-MACHO complex **1** (0.1 mol %) under the conditions described in Table 2, entry 8 (120 °C, 70 bar total pressure, H_2_:CO_2_ = 1:1) for 18 h. After completion of the reaction time, the reactor was cooled to room temperature and depressurized to atmospheric pressure. Subsequently, 3 Å molecular sieves were added to the reaction mixture, which was allowed to stand at room temperature for 5 h to facilitate drying. The mixture was then transferred to a J-Young flask and heated in an oil bath at 150 °C for an additional 18 h to enable decarbonylation. This modified two-step, one-pot protocol afforded CO in 2.6% yield (TON = 26), confirming that the use of 3 Å molecular sieves positively influences the efficiency of the RWGS reaction.

To streamline the process while suppressing the WGS side reaction, we developed a one-step strategy using a four-chamber pressure reactor (Figure S2). The reaction mixture was added in one chamber with three adjacent chambers containing 3 Å molecular sieves to sequester water vapor. Under our initial conditions, 70 bar, H_2_:CO_2_ (1:1), **1** (0.02 mmol), K_2_CO_3_ (0.1 mmol), morpholine (10 mmol), 150 °C, an initial TON of 17 was achieved in 18 h (Table 3, entry 1). Increasing the temperature to 170 °C led to a TON of 20 (Table 3, entry 2), which was increased to 249 (Table 3, entry 3) when the reaction time was increased to 90 h. The selectivity of CO in these cases was found to be 100%. The only other gases detected by GC-TCD were found to be CO_2_ and H_2_. Two control experiments were performed: one in the absence of complex **1** and the other in the absence of morpholine. In both cases, no CO formation was detected by GC–TCD analysis, confirming that both complex **1** and morpholine are essential for the observed RWGS reaction (Table 3, entries 4 and 5).

**Table 3:**
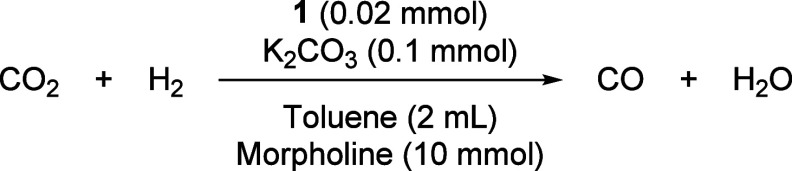
One-Step RWGS Reaction^a^

Entry	Time (h)	*T* (°C)	TON	CO Selectivity
1	18	150	17	100%
2	18	170	20	100%
3	90	170	249	100%
4^b^	18	150	0	
5^c^	18	150	0	

a Standard reaction conditions: **1** (0.02 mmol), K_2_CO_3_ (0.1 mmol), toluene (2 mL), morpholine (10 mmol), 70 bar (1:1 H_2_/CO_2_). CO yield and selectivity was determined by GC-TCD analysis.

b The reaction was performed in the absence of **1**.

c The reaction was performed in the absence of morpholine.

## Conclusion

In conclusion, we demonstrate here the proof of concept of a novel approach to conduct the reverse water gas shift reaction using an organic secondary amine as a cocatalyst. The overall process involves two steps, formylation of an amine, followed by the decarbonylation of the resultant formamide; both steps are catalyzed by a ruthenium pincer complex. Catalytic optimization studies as well as mechanistic investigation suggest two competing side reactions disfavoring the decarbonylation step, limiting the yield of CO production: (a) water gas shift reaction and (b) carbonylation of amine to make formamide. Indeed, when a higher concentration of *N*-formylmorpholine (10 mol dm^–3^) was used, a higher TON (up to 338) for the decarbonylation was achieved. To avoid the water gas shift reaction, the reaction was conducted in a multichamber pressure reactor; specifically, the reaction was conducted in one of the chambers, whereas molecular sieves were kept in three chambers to remove the water generated from the process. Using this strategy, a TON of 249 was achieved for the production of CO at 70 bar (CO_2_:H_2_ = 1:1) and 170 °C over 90 h. Remarkably, the process was found to be 100% selective toward CO and the formation of methane or methanol was not observed, unlike the processes reported using heterogeneous catalysts. We believe this approach can open new possibilities for CO_2_ utilization since the use of amines can allow this process to be integrated with CO_2_ capture.

## Supplementary Material





## Data Availability

The research data supporting this publication can be accessed from the University of St Andrews Research Portal, https://doi.org/10.17630/a2279782-f3d8-4f32-82ae-700b124b93b1.

## Notes

Additional experimental details including catalytic methods, NMR spectra for all compounds, and GC-MS, GC-TCD, and single-crystal X-ray diffraction data, as well as computational details, optimized coordinates and energies (PDF)
CCDC 2516422 contains the supplementary crystallographic data for this paper. This data can be obtained free of charge via www.ccdc.cam.ac.uk/data_request/cif (CIF)
