# Supplementary material for: Formylation–Decarbonylation Relay Strategy for the Selective Hydrogenation of CO2 to CO
Source: ACS Catal. 2026 Jan 23;16(3):2309–17. doi: 10.1021/acscatal.5c07116 (PMC12887936; doi:10.1021/acscatal.5c07116)
Supplement: Supplementary file 1 [file cs5c07116_si_001.pdf]

Supplementary Information

Formylation–Decarbonylation Relay Strategy for the Selective Hydrogenation of CO_2 to CO

James Luk[a], Luke Andrew,[a] Garima Saini,[a] Matthew J. Andrews,[a] Emily Feeke,[a] Aidan P. Mckay,[a] David B Cordes,[a] Michael Bühl,[a]* and Amit Kumar*[a]

[a] EaStCHEM, School of Chemistry, University of St Andrews

North Haugh, KY16 9ST St Andrews, (UK)

E-mail: Amit Kumar: ak336@st-andrews.ac.uk; Michael Bühl: buehl@st-andrews.ac.uk.

Contents

1. General Information 4

2. General Procedures 4

2.1. General procedure for the formylation of amines 4

2.2. General procedure for the decarbonylation of *N*-formyl morpholine 5

2.3. General procedure for the sequential formylation and decarbonylation 5

2.4. General procedure corresponding to one-pot reverse water gas shift reaction 6

2.5. General procedure for mechanistic NMR experiments 6

2.6. NMR data 7

2.6.1. NMR data acquired from the formylation of amines 7

2.6.2. NMR data acquired from decarbonylation of N-formyl morpholine 13

2.6.3. NMR data acquired from the decarbonylation of formamides 21

2.6.4. NMR data acquired from the decarbonylation of *N*-formyl morpholine at different concentrations 27

2.6.5. NMR data acquired from the sequential formylation and decarbonylation of morpholine 29

2.6.6. NMR data acquired from one-pot reverse water gas shift reaction 30

2.7. Characterisation data acquired for mechanistic study 33

2.7.1. NMR data from mechanistic study 33

2.7.2. Crystallographic data 38

3. ESI-MS data 39

3.1. ESI-MS data from mechanistic study 39

4. GC-MS data 39

4.1.1. GC-MS data acquired from the formylation of amines 39

4.1.2. GC-MS data acquired from the decarbonylation of N-formyl morpholine 47

4.1.3. GC-MS data corresponding to decarbonylation corresponding to formamides 55

4.1.4. GC-MS data acquired from the decarbonylation of N-formyl morpholine at different morpholine concentrations 61

4.1.5. GC-MS data acquired from the sequential formylation and decarbonylation of morpholine 63

4.1.6. GC-MS data acquired from the one-pot reverse water gas shift reaction 64

5. GC-TCD data 67
5.1. GC-TCD data acquired from decarbonylation of N-formyl morpholine 67
5.2. GC-TCD data acquired from the decarbonylation of formamides 75
5.3. GC-TCD data acquired from the decarbonylation of N-formyl morpholine at different morpholine concentrations 80
5.4. GC-TCD data acquired from the sequential formylation and decarbonylation of morpholine. 82
5.5. GC-TCD data acquired from the one-pot reverse water gas shift reaction .. 83
6. Computation Details 85
6.1. General Considerations 85
6.2. Possible *fac* conformations of **1** + N-formylmorpholine 86
6.3. Computational raw data 86
7. References 164

1. General Information

All manipulations were carried out under an inert atmosphere of argon or nitrogen using standard Schlenk and glove-box techniques unless specified. All chemicals (e.g. diamines, diols, formic acid, etc) were purchased from Sigma-Aldrich, Thermofisher, TCI or Strem, unless stated otherwise, and used as received. Complexes **2**,[1] **3**,[1] **4**,[2] and **5**[3] were synthesised according to literature procedures. THF and toluene were dried by a solvent purification system and degassed using freeze-pump-thaw technique before being dried on 4Å molecular sieves before use, while anisole was only dried on 4Å molecular sieves and degassed using freeze-pump-thaw technique. The Quadracell reactor was purchased from Asynt (https://www.asynt.com/product/quadracell-4-position-high-pressure-reactor/).

All NMR spectroscopic experiments were carried out at 298 K using a Bruker Avance II 400 400 MHz or a Bruker AV-III HD 500 MHz, unless stated otherwise, and reported in ppm (δ). NMR spectroscopy abbreviations: b - broad, s – singlet, d – doublet, t – triplet, q – quartet, m – multiplet. Error in the estimation of conversion and yields is ±5%. Gas Chromatography (GC-MS) was performed on an Agilent 8860 series GC using an Agilent 5977B GC/MSD, fitted with an Agilent J&W HP-5ms Ultra Inert GC-column (30 m, 0.25 mm, 0.25 µm, 7-inch cage). Helium was used as carrier gas with a column flow of 12 mL/min. Inlet temperature (250 °C) and initial oven temperature (50 °C) were used for an initial hold of 4.2 minutes, before an oven ramp of 10 °C/min-1 for 15 minutes until 250 °C where it was held until 39 minutes. The gas headspace was analysed using an Agilent 8860 gas chromatography (columns: Porapak Q, 2.74 m × 2 mm and 5 Å molecular sieves, 1.83 m × 2 mm, temperature: 60 °C, 2 min, ramped to 150 °C over 4.5 min) equipped with a thermal conductivity detector (TCD) with Ar (30 ml/min) as the carrier gas. Calibration was carried out with certified calibration mixtures of H_2 and CO, and CO_2. Yields were quantified by first determining gas concentrations from GC-TCD peak areas using external calibration, then converting to moles using the ideal gas law based on the reactor headspace volume (mL) and reaction conditions. Due to the large excess of CO_2 and H_2, their accurate conversion was not possible to be obtained under our setting.

2. General Procedures

2.1. General procedure for the formylation of amines

Complex **1** (0.01 mmol) and KO*t*Bu (0.04 mmol) were added to a microwave vial before being vacuum cycled with argon three times. THF (1 mL) and the desired amine (1 mmol) were then added to the microwave vial under an argon flow. An autoclave was then vacuum cycled with argon three times. The microwave vial was then pierced with two needles before being placed in the autoclave under argon. The autoclave was sealed and

pressurised with 70 bar H_2:CO_2 and heated at 120 °C for 20 h. At the end of reaction time, the autoclave was cooled down to room temperature, and pressure was carefully released. 1,3,5-Trimethoxybenzene (0.67 mmol) internal standard was added to the reaction mixture, and the reaction mixture was then analysed by ^{1}H NMR spectroscopy and GC-MS. The characterisation data of the formed formamides match well with the literature.[3–6]

2.2. General procedure for the decarbonylation of *N*-formyl morpholine

In a glovebox, complex **1** (0.01 mmol) and base (0.1 mmol) were added to a 250 mL J-Youngs flask, followed by the addition of a solvent (2 mL) and the desired formamide (0.86 or 1 mmol). The J-Young's flask was then sealed and heated at 150 °C for 18 h. The reaction mixture was then cooled to room temperature. The gases present in the headspace were taken through a syringe and analysed by GC-TCD. 1,3,5-Trimethoxybenzene (0.17 mmol) was then added as an internal standard, and the reaction mixture was analysed by ^{1}H NMR spectroscopy and GC-MS.

2.3. General procedure for the sequential formylation and decarbonylation

The general procedure outline from section 2.1. was performed using morpholine as the amine (Figure S1, I). At the end of the reaction time, the reaction mixture was brought to room temperature, and the pressure was released to 1.5 bar before the remaining pressure was released slowly through the Schlenk line and the mixture was purged with argon (30 min). If desired, the mixture was then transferred to a flask containing activated 3 Å molecular sieves under argon (Figure S1, II). After 5 h, the reaction mixture was transferred to a 250 mL J-Youngs flask and heated at 150 °C for 18 h (Figure S1, III). The reaction mixture was then cooled to room temperature. The gases present in the headspace were taken through a syringe and analysed by GC-TCD. 1,3,5-Trimethoxybenzene (0.33 mmol) was then added as an internal standard, and the reaction mixture was analysed by ^{1}H NMR spectroscopy and GC-MS.

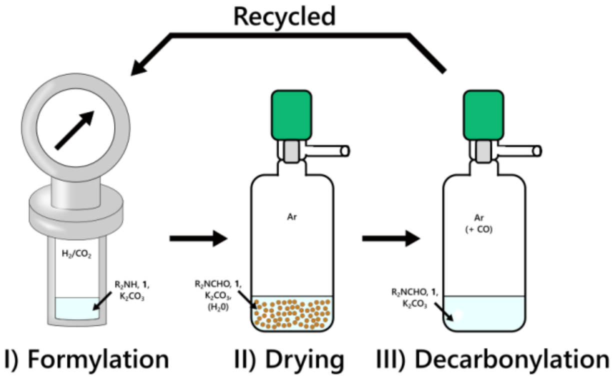

Figure S1: Steps I-III involved in sequential formylation and decarbonylation of morpholine for overall RWGS reaction.

2.4. General procedure corresponding to one-pot reverse water gas shift reaction

In a glovebox, complex **1** (0.02 mmol), K_2CO_3 (0.1 mmol), morpholine (10 mmol) and toluene (2 mL) were added to one of the chambers of a quadracell reactor (Figure S2), while the other 3 chambers were filled with activated 3 Å molecular sieves. The reactor was then sealed before being pressurised with 70 bar (H_2:CO_2). The reactor was then heated at the desired temperature for the desired time. The reactor was cooled to room temperature after completion of the reaction time. The gases present in the headspace were taken through a syringe and analysed by GC-TCD. 1,3,5-Trimethoxybenzene (0.33 mmol) was then added as an internal standard, and the reaction mixture was analysed by ^{1}H NMR spectroscopy and GC-MS.

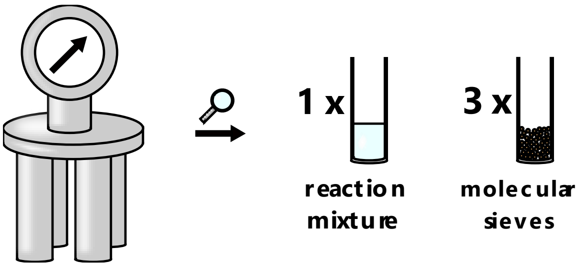

Figure S2: Visualisation of internal components of chambers of quadracell reactor.

2.5. General procedure for mechanistic NMR experiments

In a glovebox, complex **1** (0.01 mmol), KO*t*Bu (0.02 mmol, 2 eqv.) and d_8-toluene (~0.5 mL) were added to a J-Young's NMR tube. The NMR tube was then placed under CO atmosphere and ^{1}H and ^{31}P{^{1}H} NMR spectra recorded. The CO pressure was then removed, and N-formylmorpholine (0.086 mmol) was added under argon before the NMR tube was heated to 150 °C, recording ^{1}H and ^{31}P{^{1}H} NMR at t = 1 h, and t = 18 h.

2.6. NMR data

2.6.1. NMR data acquired from the formylation of amines

Table S1: Formylation of amines to corresponding formamides.[a]

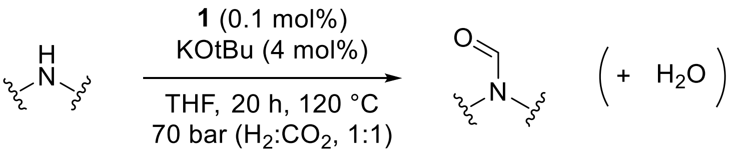

Entry	Substrate	Conversion (%)	Yield (%)
**1**	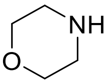	93	91
**2**	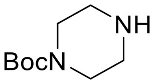	97	80
**3**	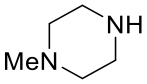	97	89
**4**	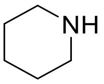	91	91
**5**	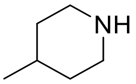	91	89
**6**	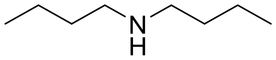	65	47
**7**	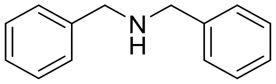	50	49
**8**	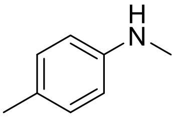	n.o.	n.o.
**9**	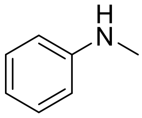	n.o.	n.o.
**10**	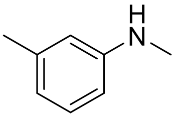	n.o.	n.o.
**11**	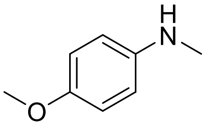	n.o.	n.o.
**12**	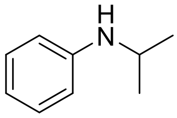	n.o.	n.o.

[a]Standard reaction conditions: Amine (10 mmol), **1** (0.1 mol%), KO*t*Bu (4 mol%), THF (1 mL), 20 h, 120 °C, 70 bar (H_2:CO_2=1:1). Yields were determined by ^{1}H NMR spectroscopy using 1,3,5-trimethoxybenzene as an internal standard. n.o. = not observed.

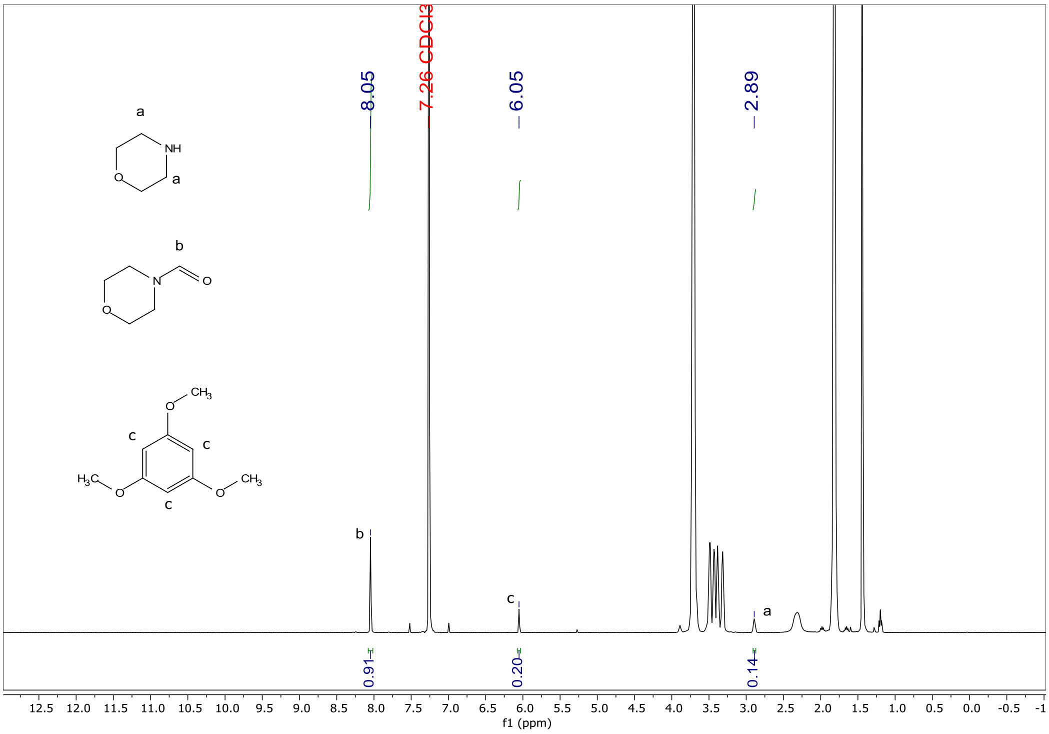

Figure S3: ^{1}H NMR spectrum ($CDCl_3$, 400 MHz, 298 K) corresponding to Table S1, entry 1.

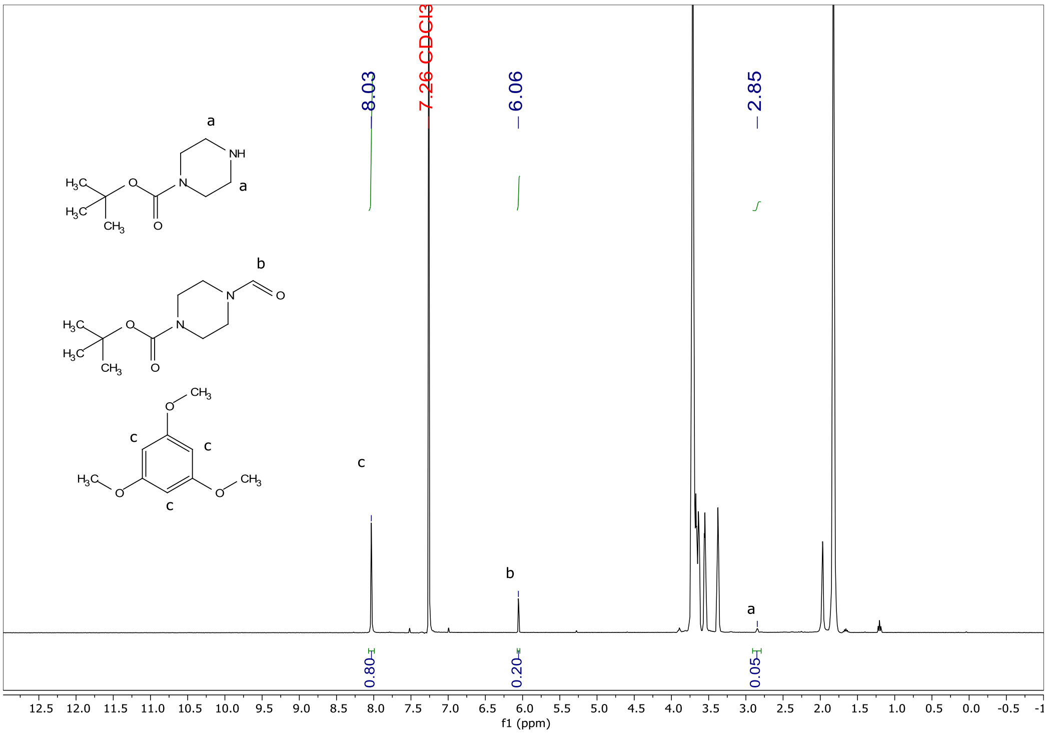

Figure S4: ^{1}H NMR spectrum ($CDCl_3$, 400 MHz, 298 K) corresponding to Table S1, entry 2.

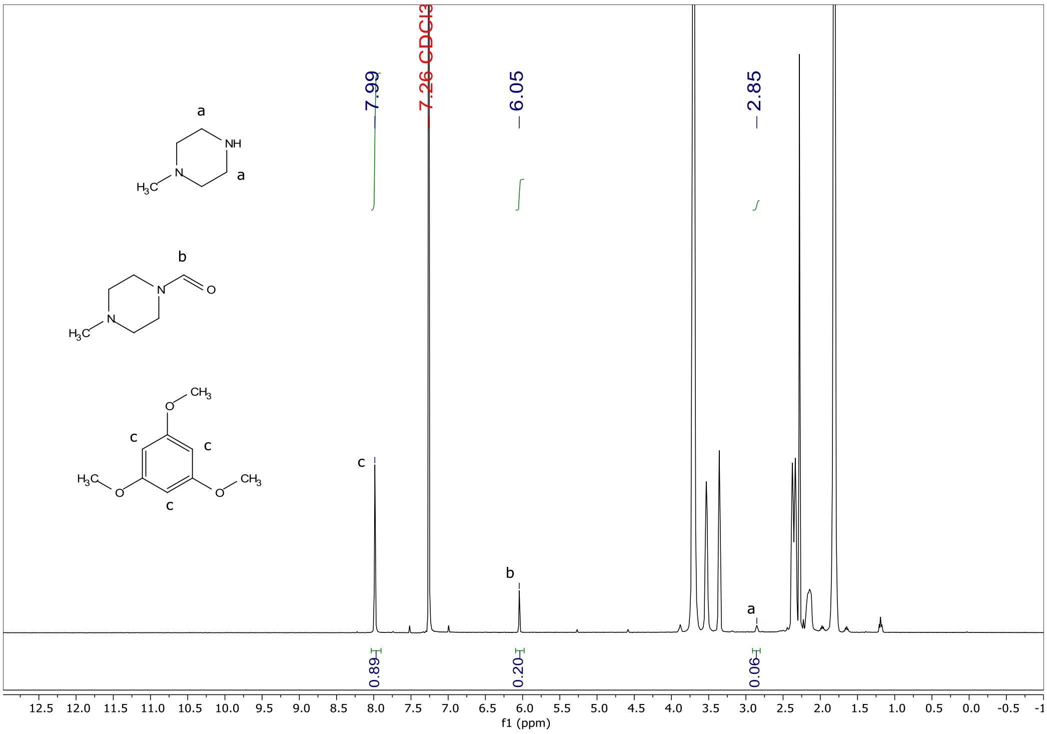

Figure S5: ¹H NMR spectrum ($CDCl_3$, 400 MHz, 298 K) corresponding to Table S1, entry 3.

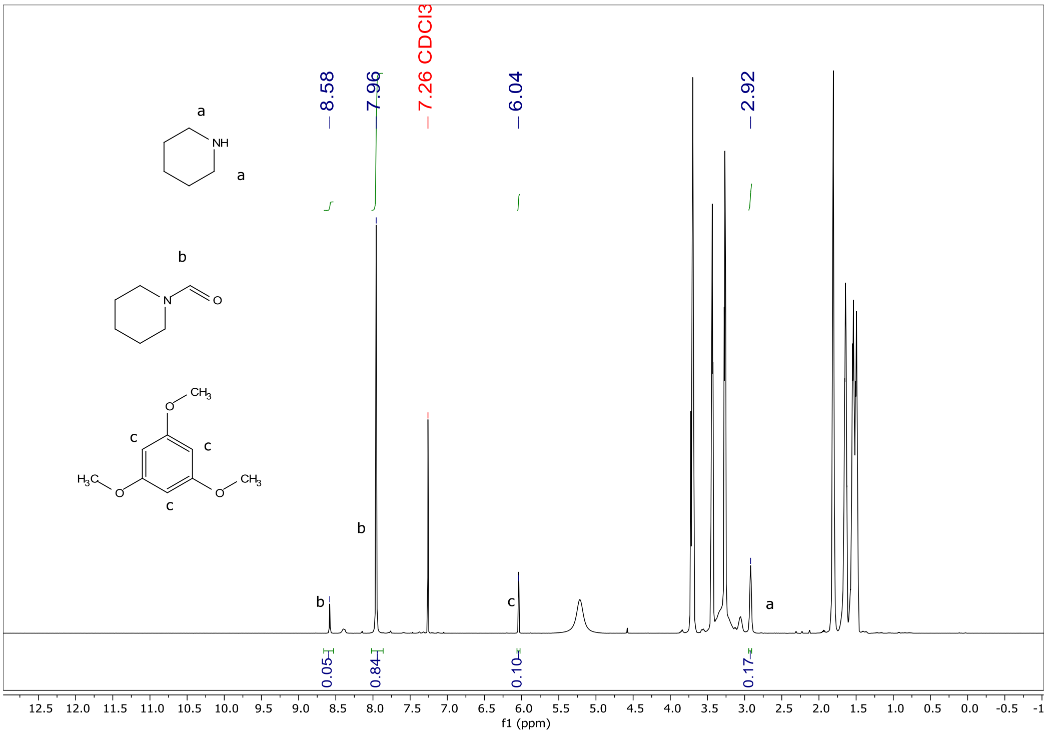

Figure S6: ¹H NMR spectrum ($CDCl_3$, 400 MHz, 298 K) corresponding to Table S1, entry 4.

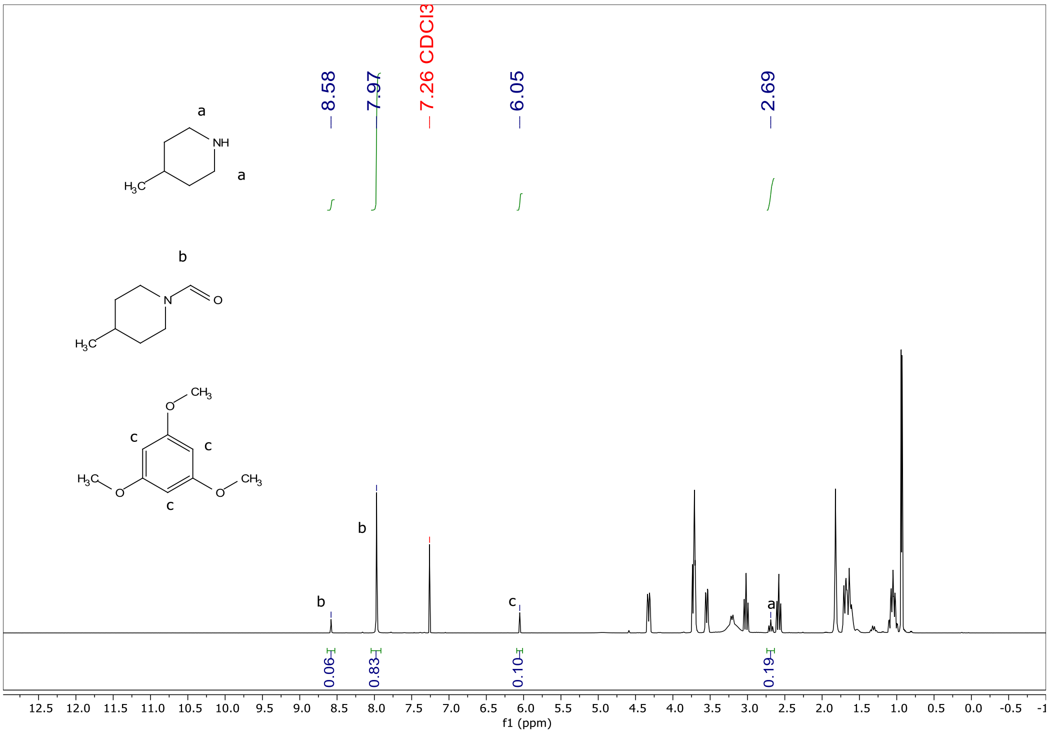

Figure S7: ^{1}H NMR spectrum ($CDCl_3$, 400 MHz, 298 K) corresponding to Table S1, entry 5.

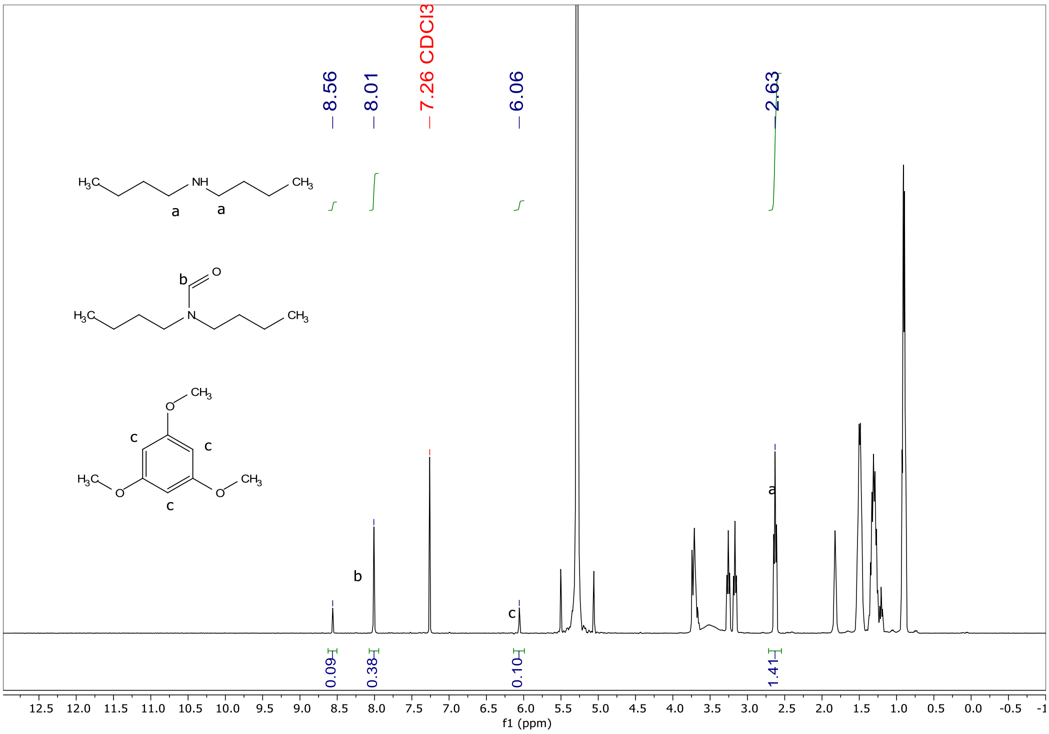

Figure S8: ^{1}H NMR spectrum ($CDCl_3$, 400 MHz, 298 K) corresponding to Table S1, entry 6.

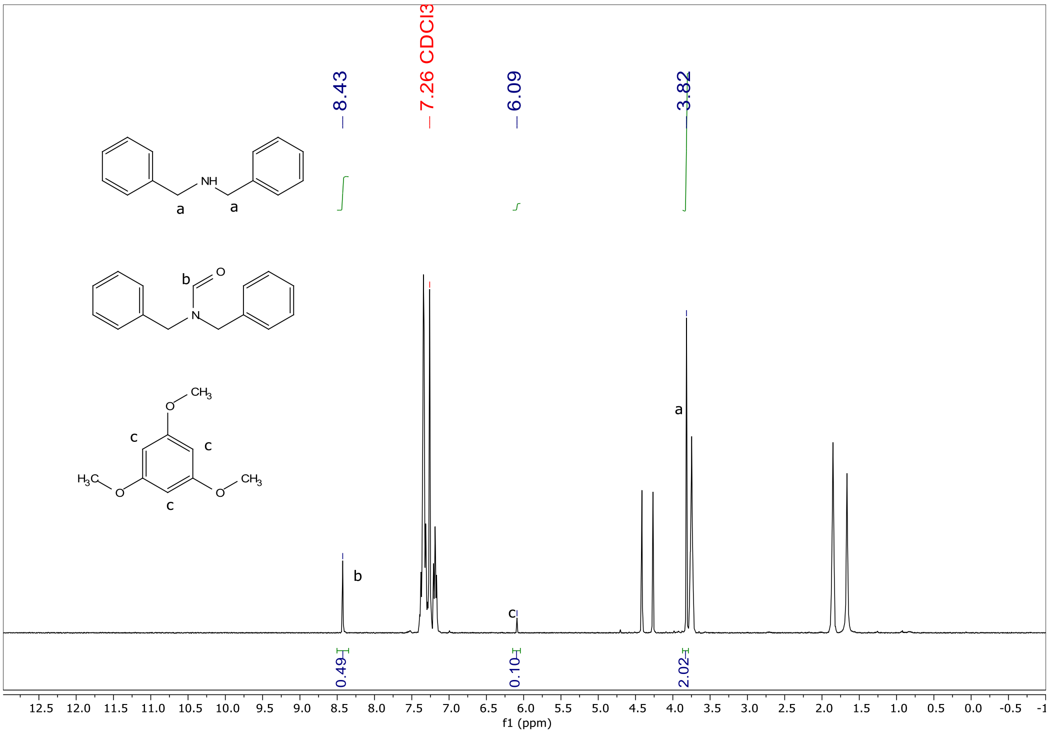

Figure S9: ^{1}H NMR spectrum ($CDCl_3$, 400 MHz, 298 K) corresponding to Table S1, entry 7.

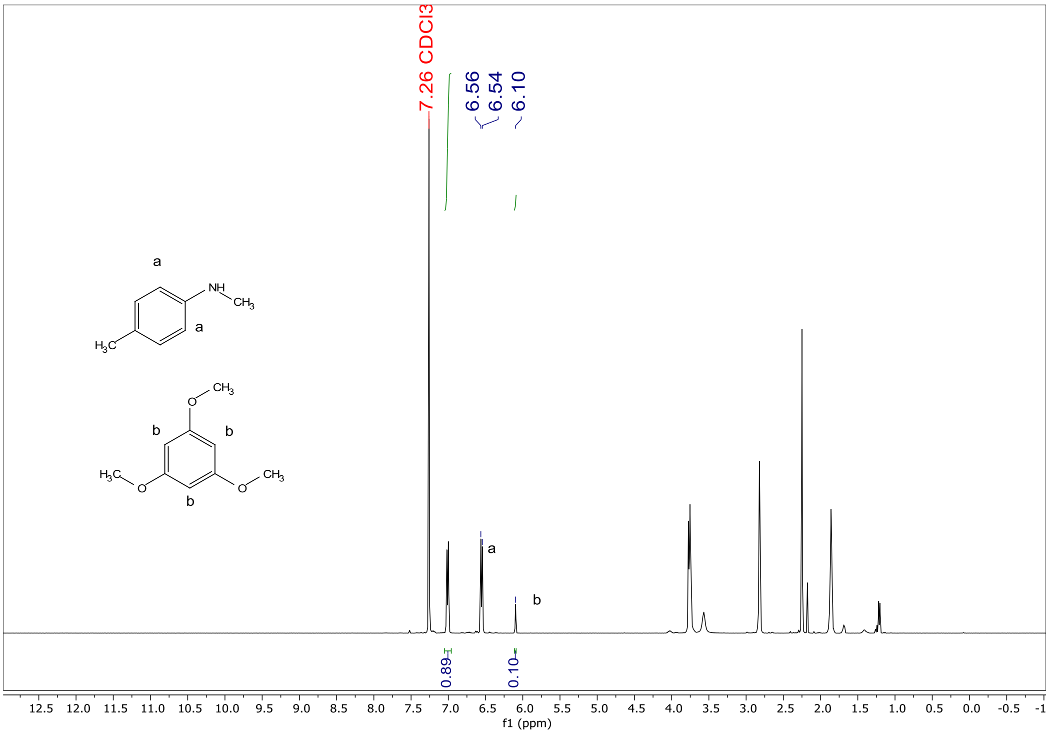

Figure S10: ^{1}H NMR spectrum ($CDCl_3$, 400 MHz, 298 K) corresponding to Table S1, entry 8.

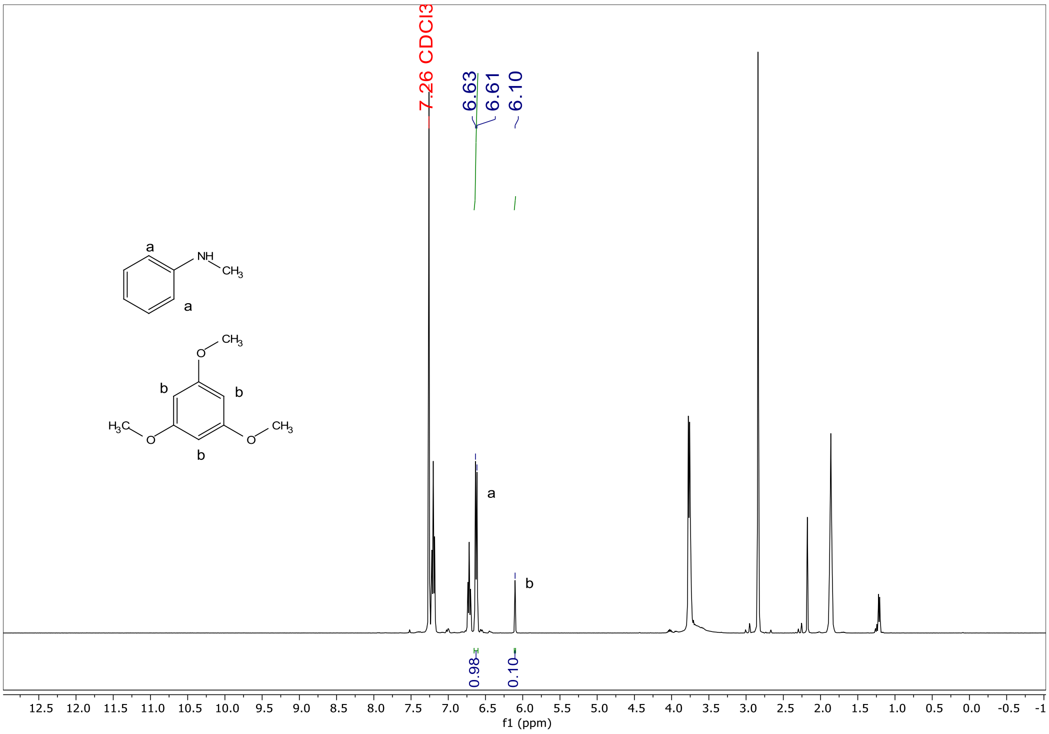

Figure S11: ^{1}H NMR spectrum ($CDCl_3$, 400 MHz, 298 K) corresponding to Table S1, entry 9.

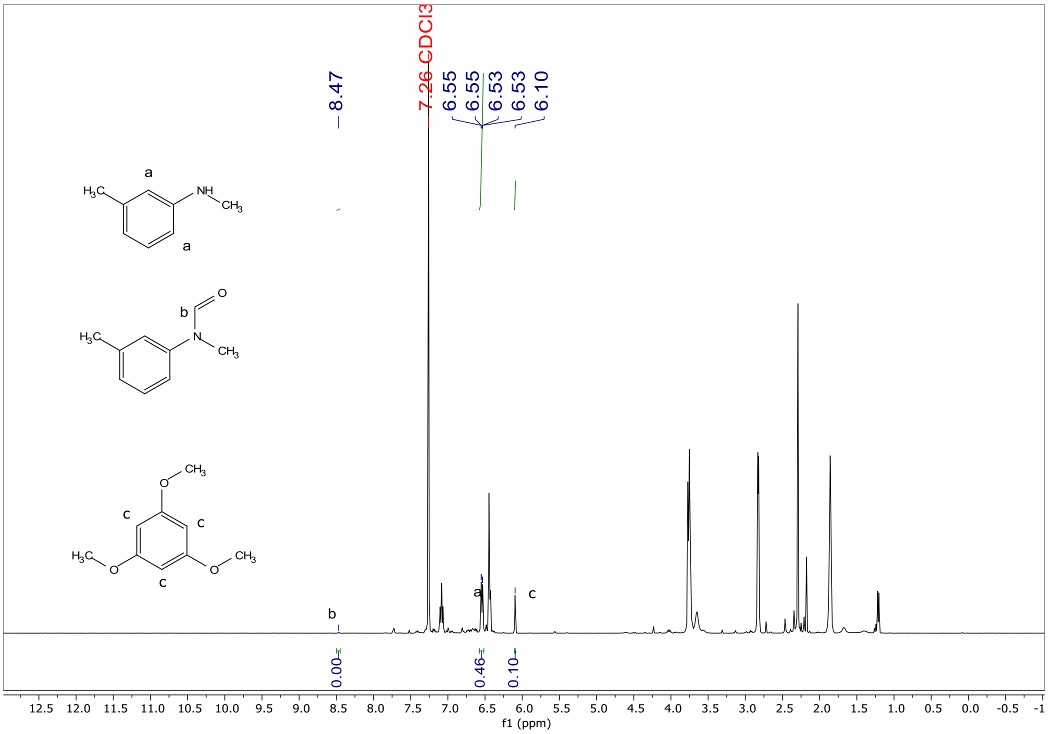

Figure S12: ^{1}H NMR spectrum ($CDCl_3$, 400 MHz, 298 K) corresponding to Table S1, entry 10.

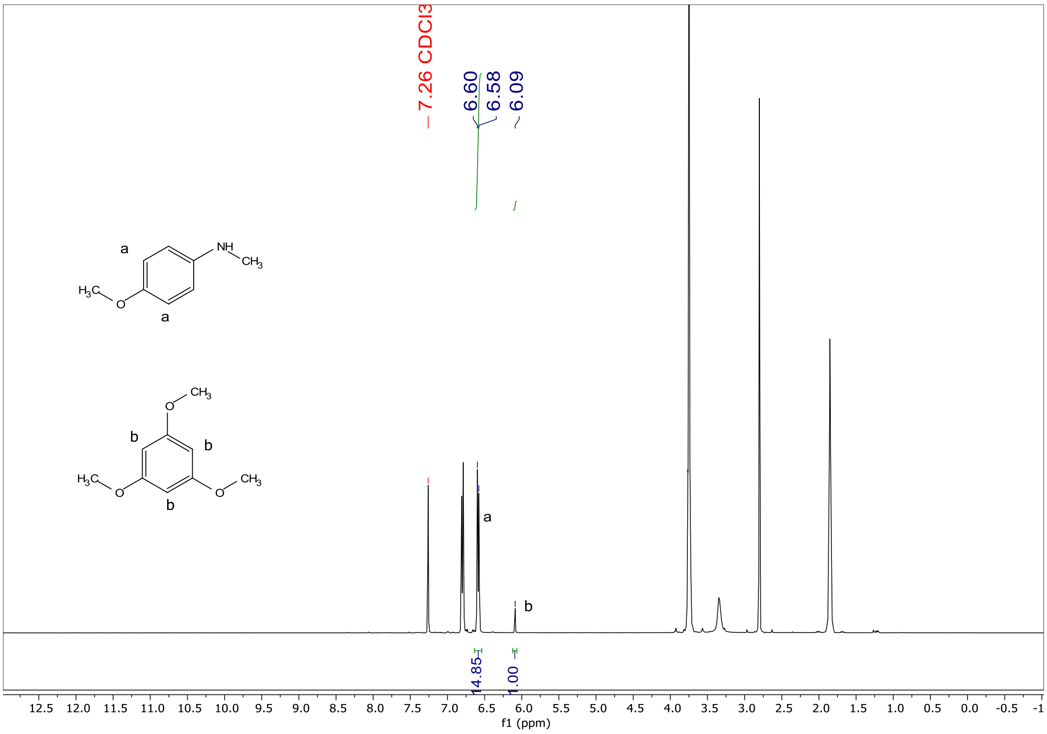

Figure S13: ^{1}H NMR spectrum ($CDCl_3$, 400 MHz, 298 K) corresponding to Table S1, entry 11.

2.6.2. NMR data acquired from decarbonylation of N-formyl morpholine

Table S2: Optimisation table for decarbonylation of N-formyl morpholine.[a]

Entry	Pre-catalyst (mol%)	Base (mol%)	Solvent (mL)	T (°C)	Conversion (%)	Amine yield (%)	CO yield /selectivity (%)
**1**	**1** (1.2)	KO*t*Bu (4)	THF	120	15	13	13/96
**2**	**1** (1)	KO*t*Bu (4)	Toluene	120	19	14	14/97
**3**	**1** (1)	KO*t*Bu (4)	Anisole	120	7	6	4/87
**4**	**1** (1)	KO*t*Bu (4)	Toluene	150	23	20	15/86
**5**	**1** (1)	KO*t*Bu (10)	Toluene	150	32	25	16/90
**6**	**1** (1)	KO*t*Bu (20)	Toluene	150	45	32	18/96
**7**	**1** (1)	NaO*t*Bu (10)	Toluene	150	26	17	18/85
**8**	**1** (1)	K_2CO_3 (10)	Toluene	150	40	28	28/89
**9**	**1** (1)	Cs_2CO_3 (10)	Toluene	150	41	23	23/98
**10**	**1** (1)	K_2CO_3 (20)	Toluene	150	42	27	27/93
**11**	**2** (1)	K_2CO_3 (10)	Toluene	150	5	n.o.	2/76
**12**	**3** (1)	K_2CO_3 (10)	Toluene	150	12	n.o.	2/89
**13**	**4** (1)	K_2CO_3 (10)	Toluene	150	8	n.o.	3/84
**14**	**5** (1)	K_2CO_3 (10)	Toluene	150	16	15	4/75

**15**	**1** (2)	K_2CO_3 (10)	Toluene	150	64	51	48/83
**16**[b]	**1** (1)	K_2CO_3 (10)	Toluene	150	0	0	n.o.

[a]standard reaction conditions: N-formylmorpholine (1 mmol), precatalyst, base, solvent (2 mL), 18 h. Yields were determined by ^{1}H NMR spectroscopy using 1,3,5-trimethoxybenzene as an internal standard.[b]reaction was carried out under 1 bar CO atmosphere.

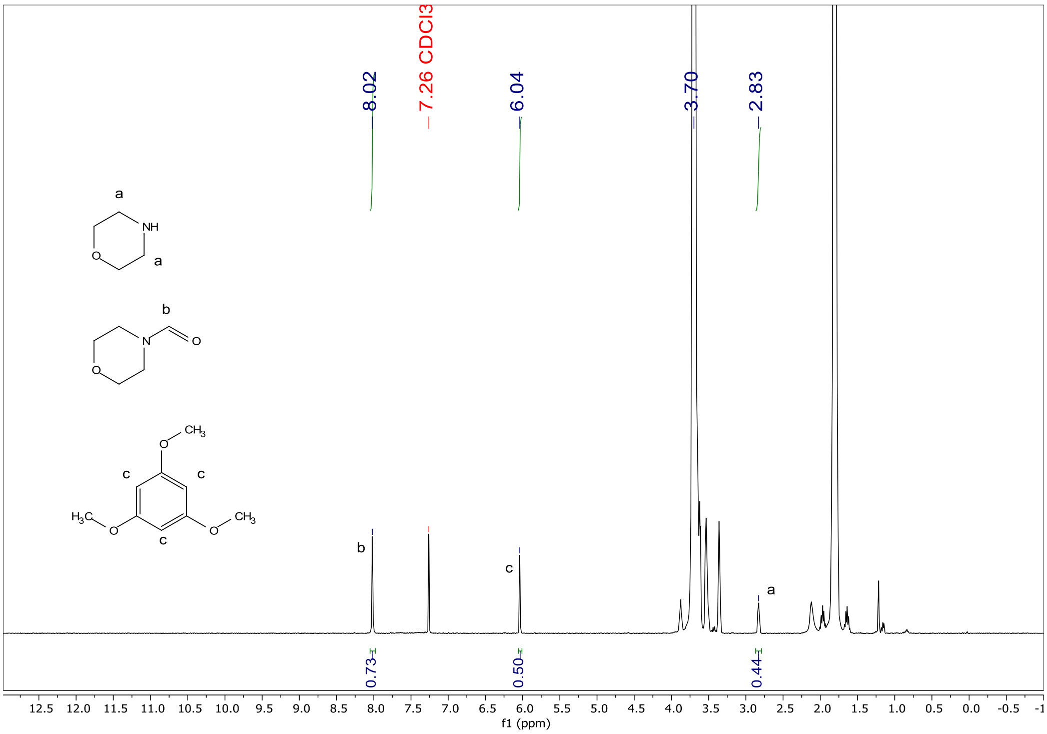

Figure S14: ^{1}H NMR spectrum ($CDCl_3$, 400 MHz, 298 K) corresponding to Table S2, entry 1.

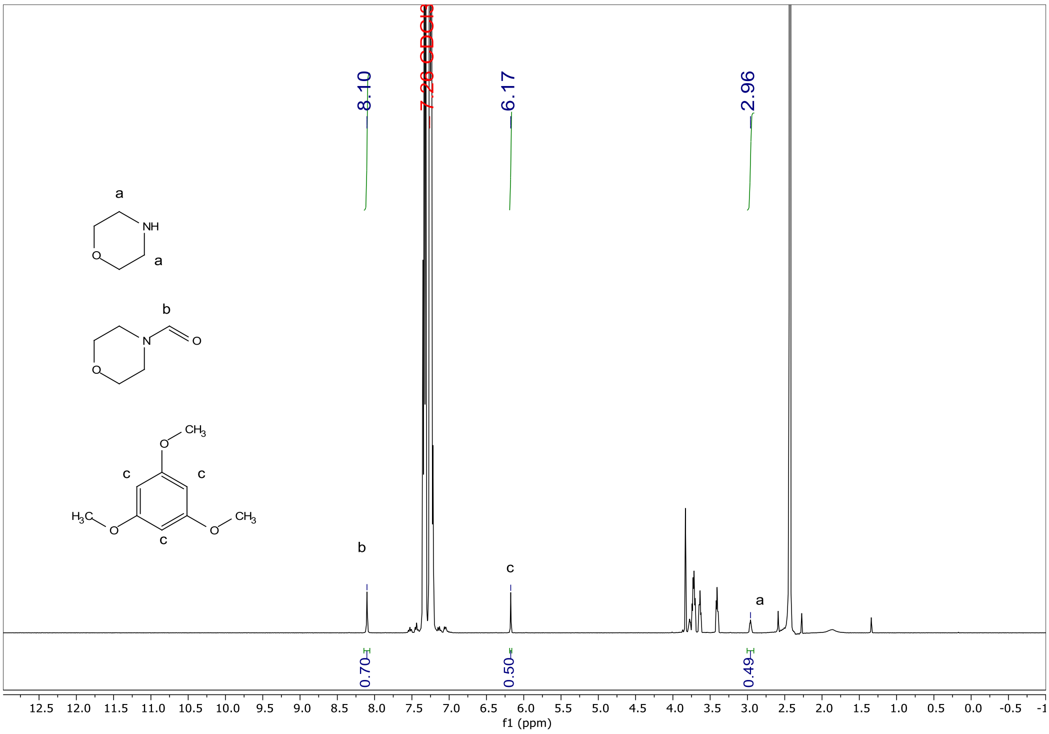

Figure S15: ^{1}H NMR spectrum ($CDCl_3$, 400 MHz, 298 K) corresponding to Table S2, entry 2.

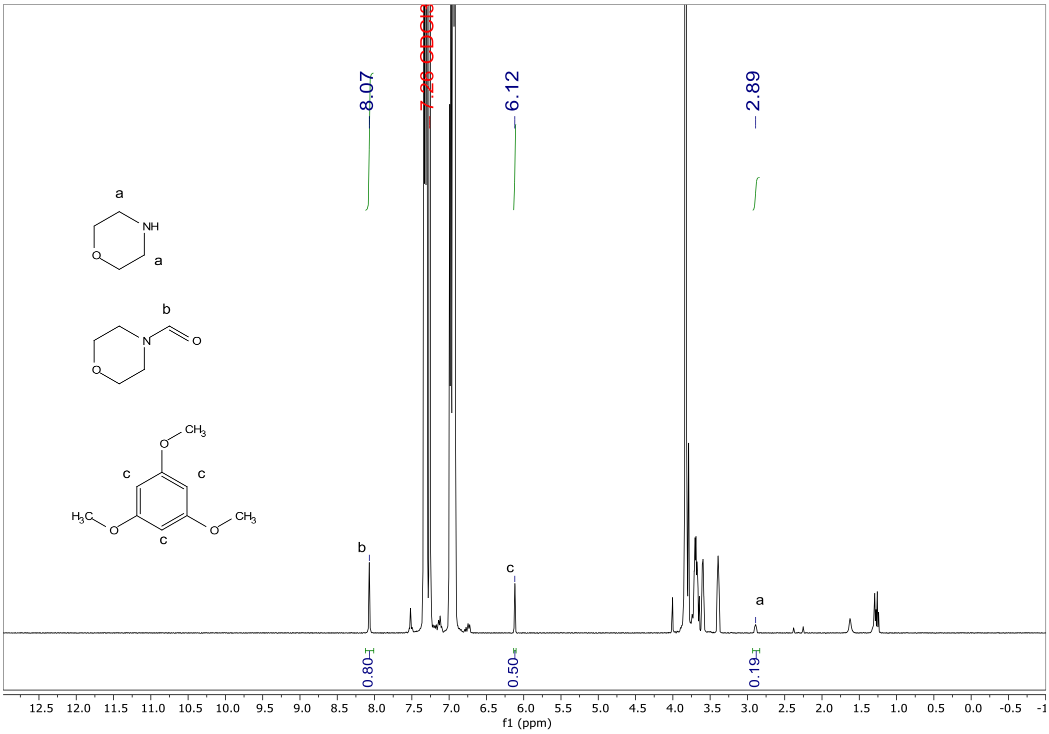

Figure S16: ^{1}H NMR spectrum ($CDCl_3$, 400 MHz, 298 K) corresponding to Table S2, entry 3.

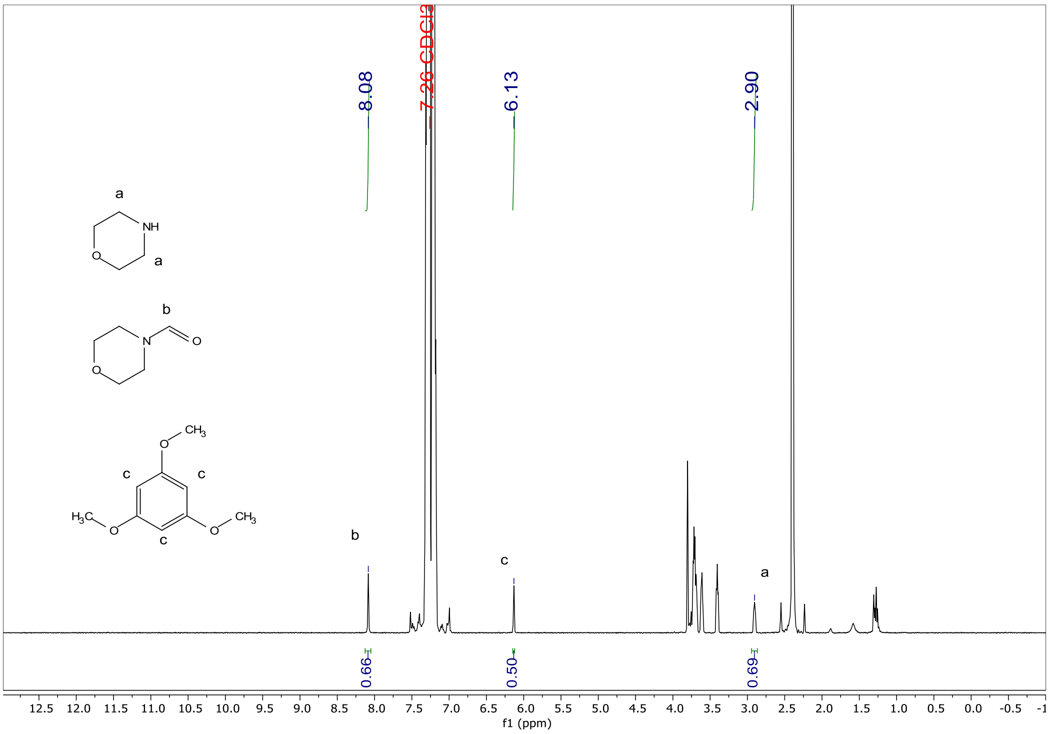

Figure S17: ^{1}H NMR spectrum ($CDCl_3$, 400 MHz, 298 K) corresponding to Table S2, entry 4.

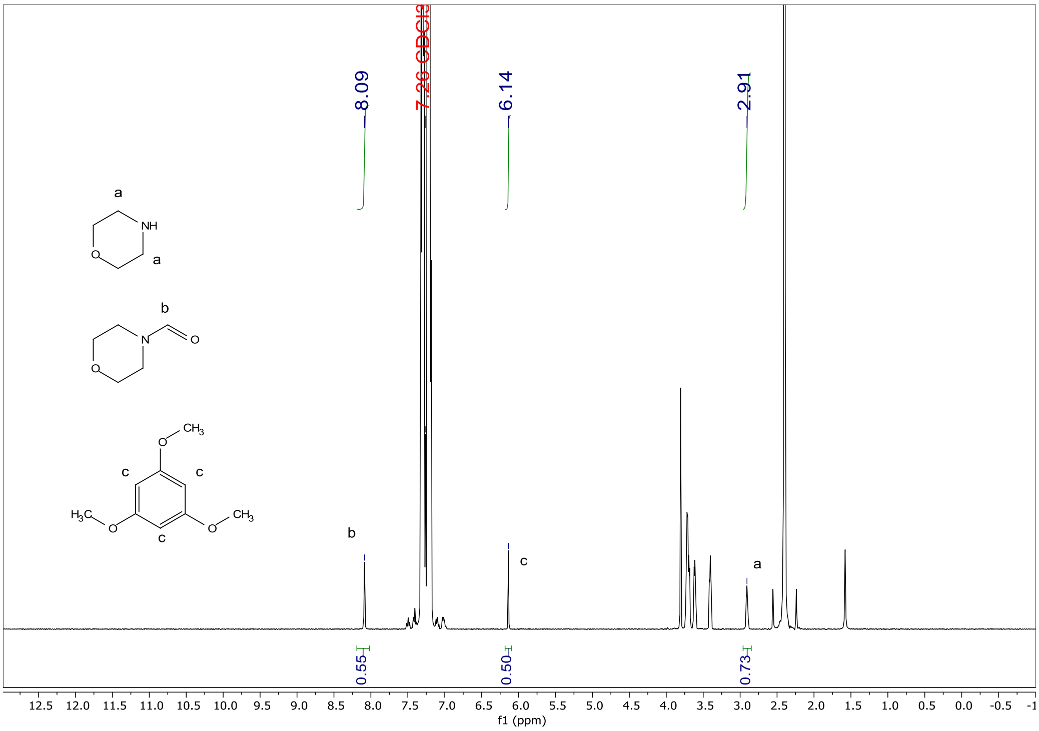

Figure S18: ^{1}H NMR spectrum ($CDCl_3$, 400 MHz, 298 K) corresponding to Table S2, entry 5.

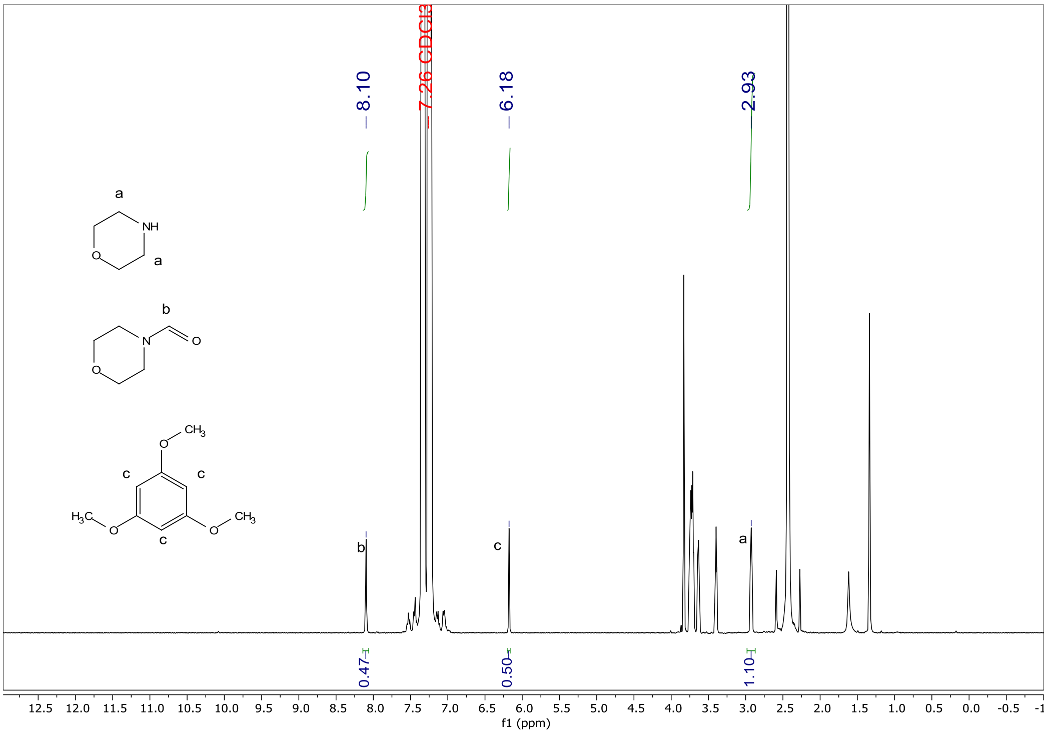

Figure S19: ^{1}H NMR spectrum ($CDCl_3$, 400 MHz, 298 K) corresponding to Table S2, entry 6.

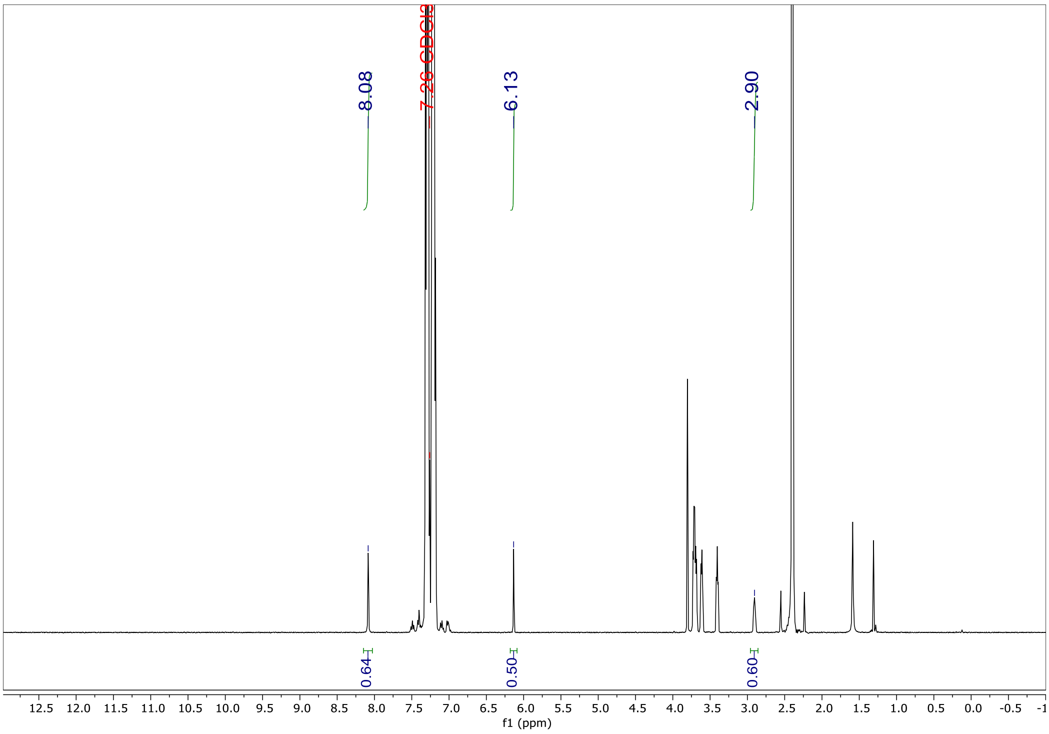

Figure S20: ^{1}H NMR spectrum ($CDCl_3$, 400 MHz, 298 K) corresponding to Table S2, entry 7.

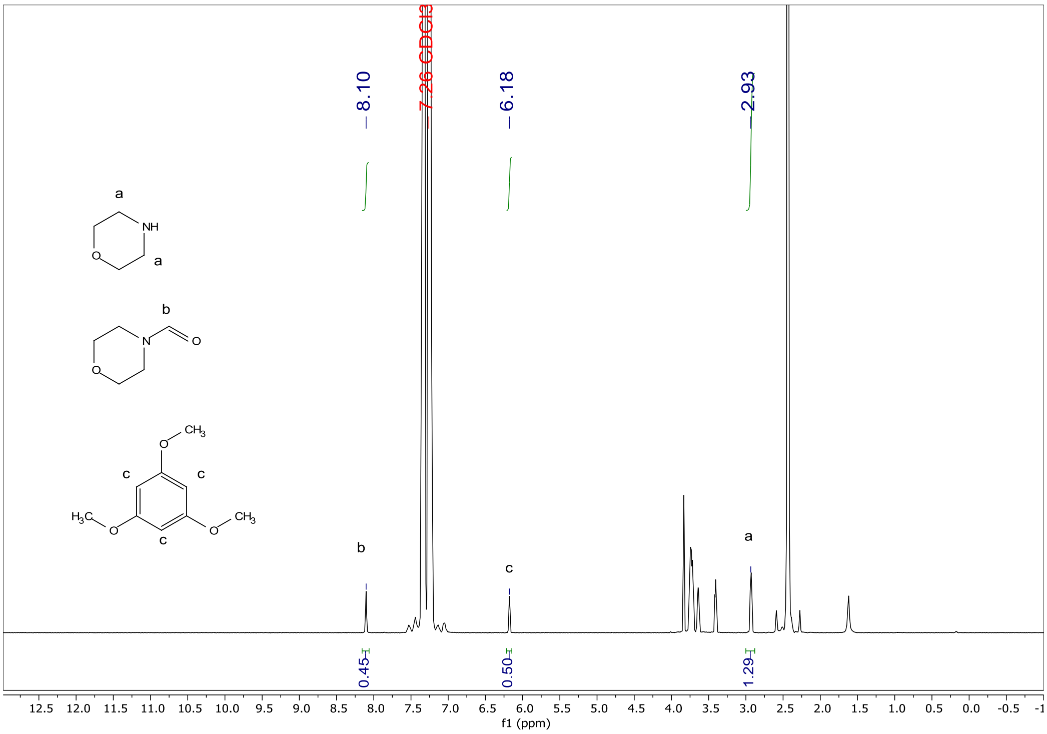

Figure S21: ^{1}H NMR spectrum ($CDCl_3$, 400 MHz, 298 K) corresponding to Table S2, entry 8.

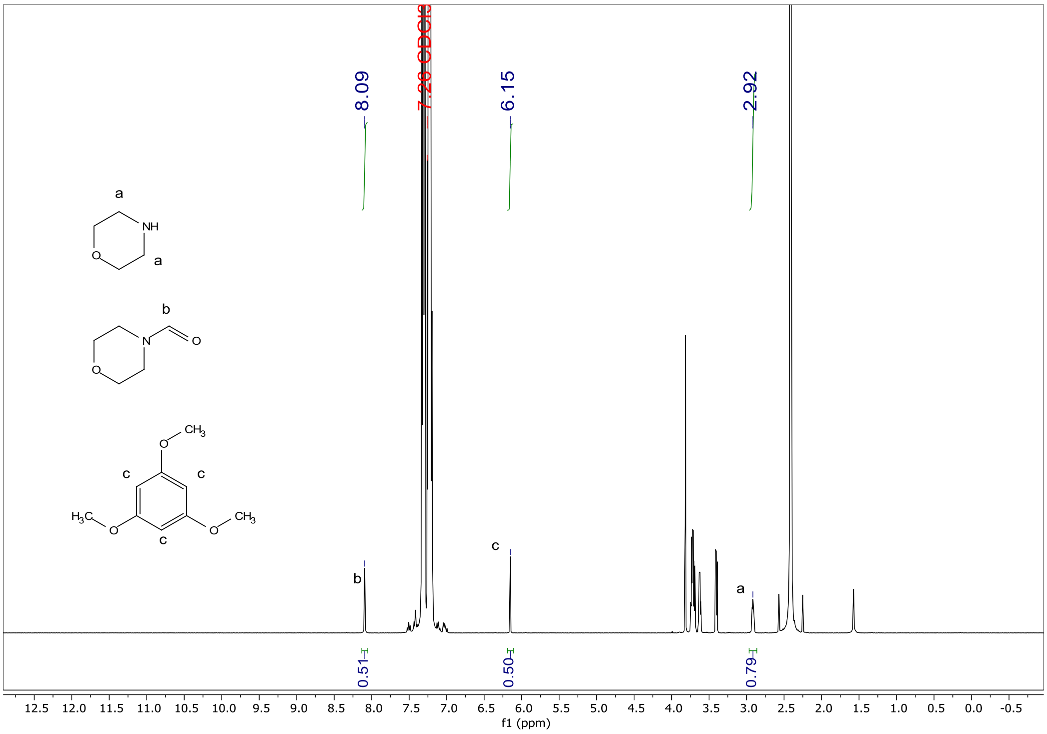

Figure S22: ^{1}H NMR spectrum ($CDCl_3$, 400 MHz, 298 K) corresponding to Table 2, entry 9.

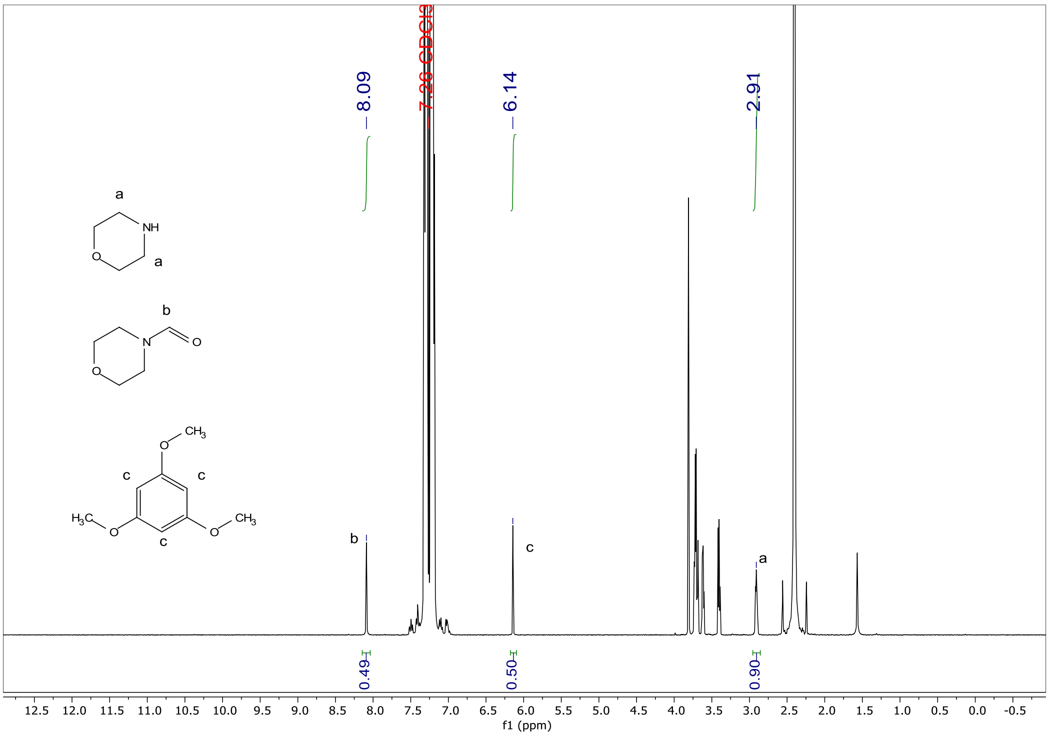

Figure S23: ^{1}H NMR spectrum ($CDCl_3$, 400 MHz, 298 K) corresponding to Table S2, entry 10.

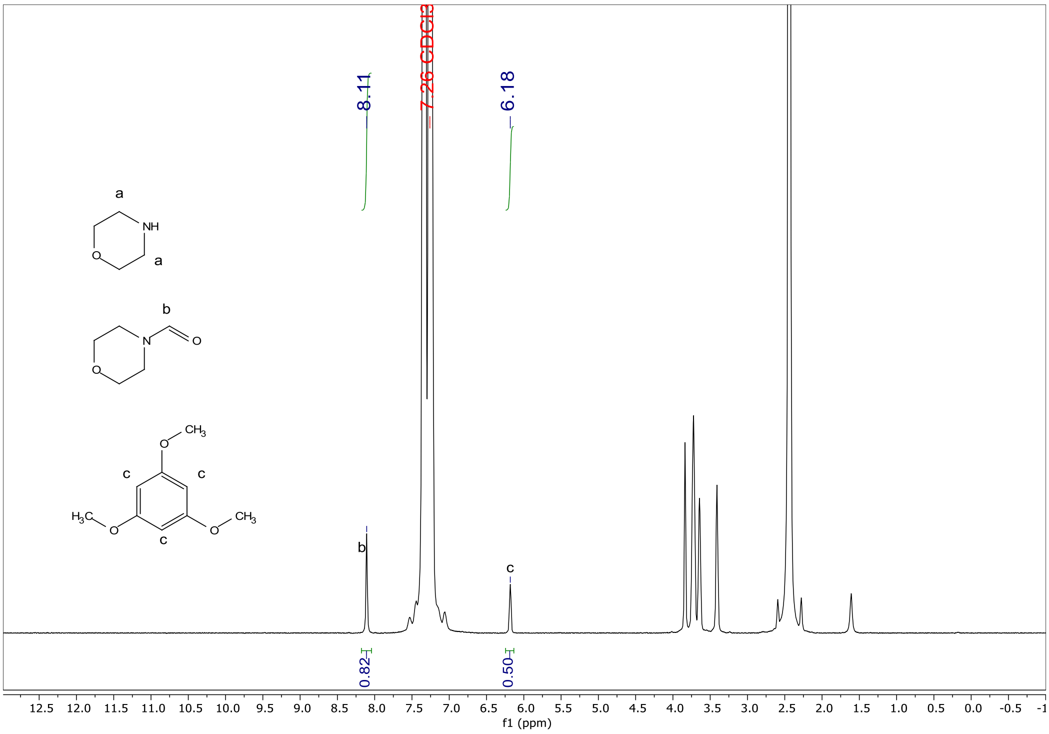

Figure S24: ^{1}H NMR spectrum ($CDCl_3$, 400 MHz, 298 K) corresponding to Table S2, entry 11.

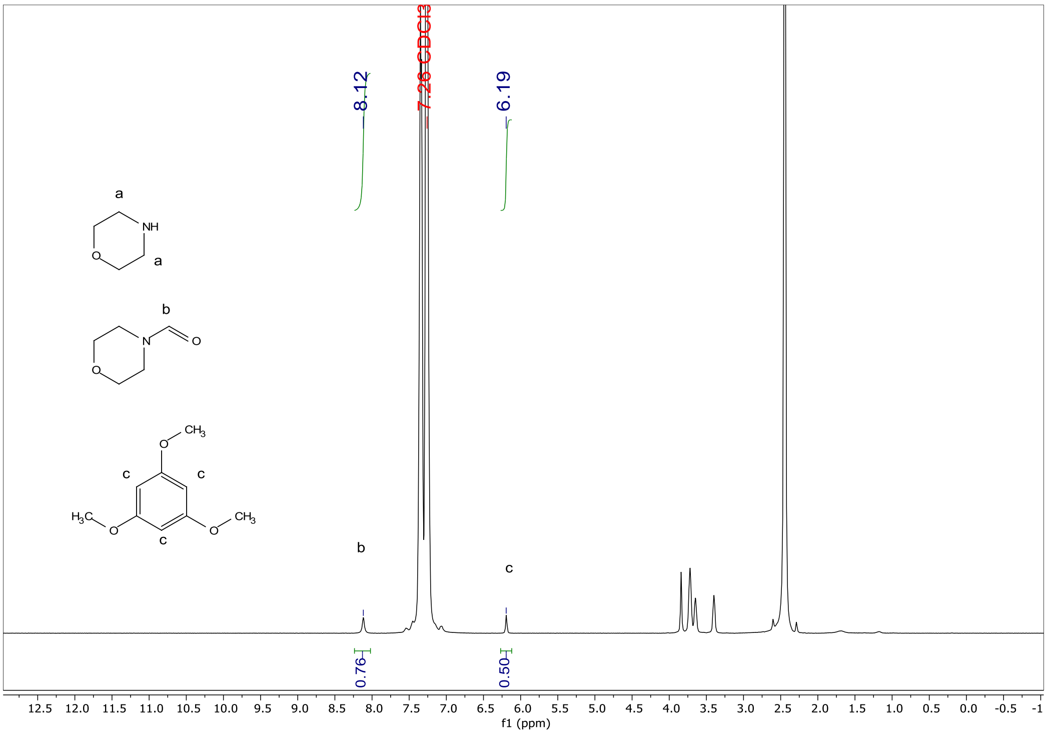

Figure S25: ^{1}H NMR spectrum ($CDCl_3$, 400 MHz, 298 K) corresponding to Table S2, entry 12.

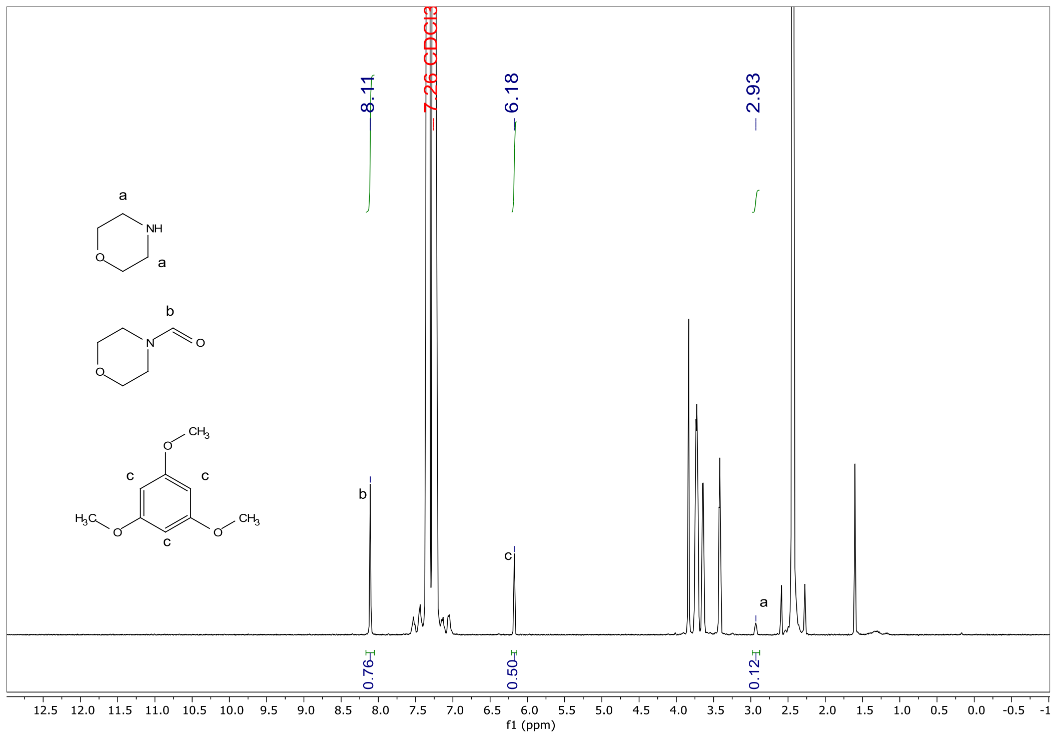

Figure S26: ^{1}H NMR spectrum ($CDCl_3$, 400 MHz, 298 K) corresponding to Table S2, entry 13.

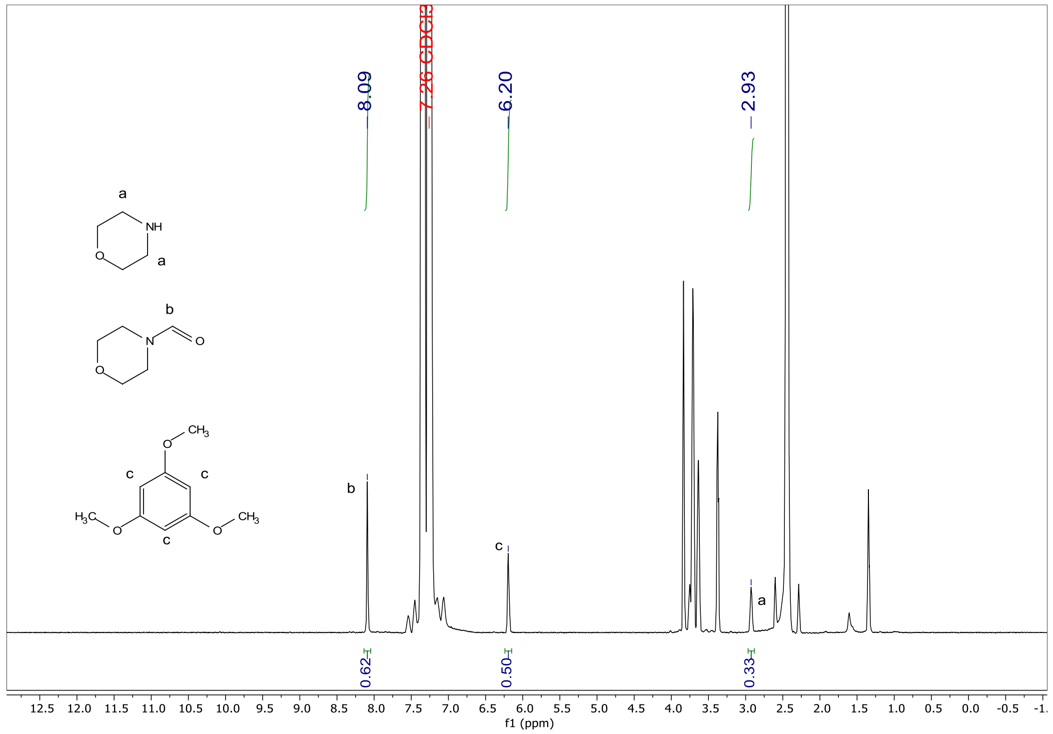

Figure S27: ^{1}H NMR spectrum ($CDCl_3$, 400 MHz, 298 K) corresponding to Table S2, entry 14.

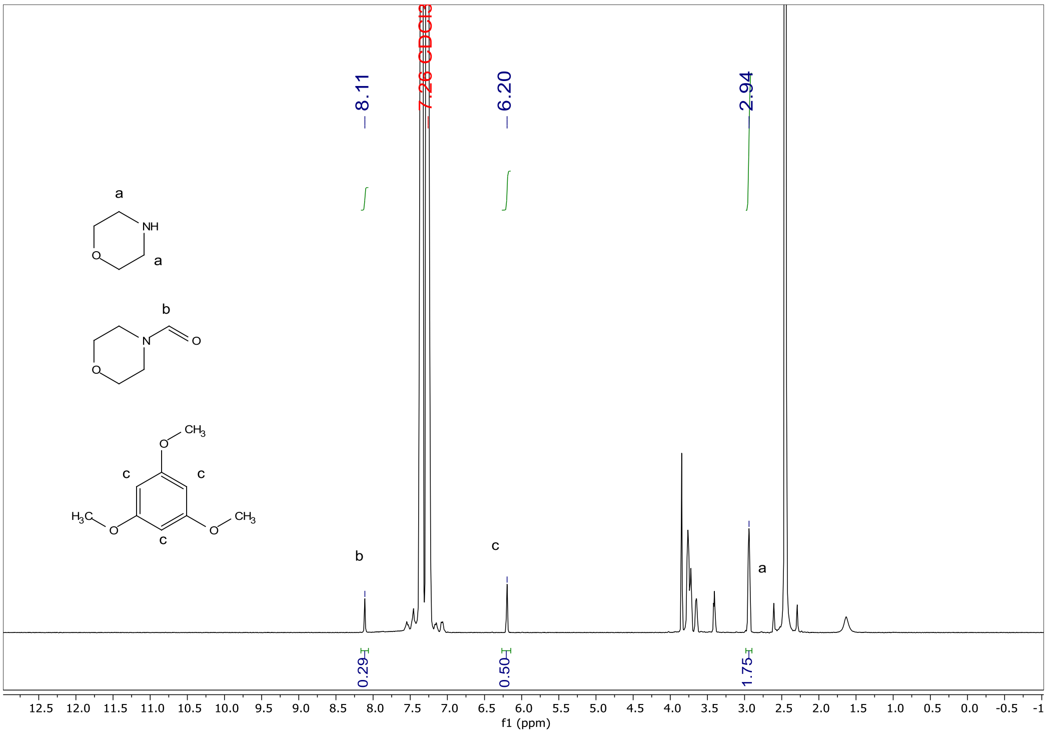

Figure S28: ^{1}H NMR spectrum ($CDCl_3$, 400 MHz, 298 K) corresponding to Table S2, entry 15.

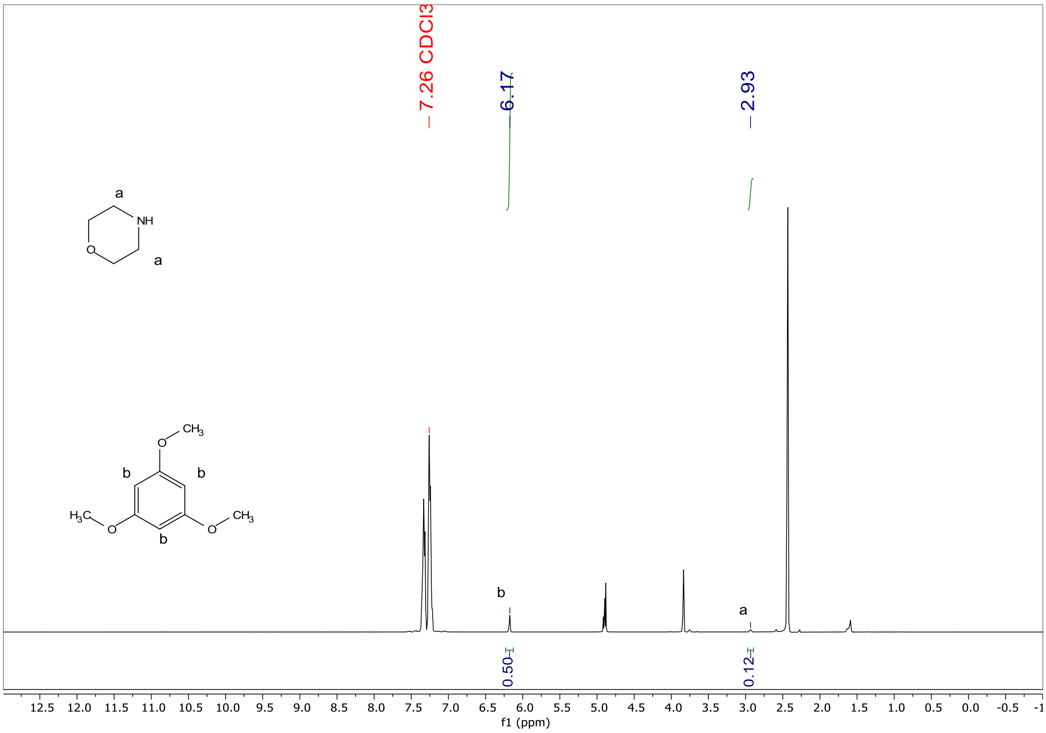

Figure S29: ^{1}H NMR spectrum ($CDCl_3$, 400 MHz, 298 K) corresponding to Table S2, entry 16.

2.6.3. NMR data acquired from the decarbonylation of formamides

Table S3. Substrate scope for decarbonylation of formamides[a]

Entry	Substrate	Conversion (%)	Amine yield (%)	CO yield /selectivity (%)
**1**	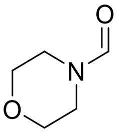	35	27	27
**2**	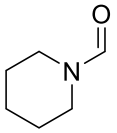	38	27	27
**3**	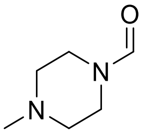	47	30	30
**4**	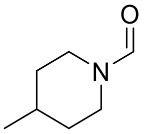	35	25	24
**5**	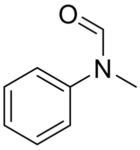	44	36	34
**6**	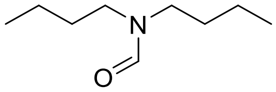	30	24	24
**7**	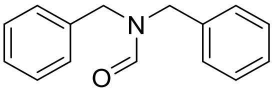	22	22	22
**8**	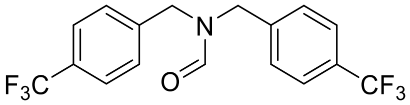	19	19	19
**9**	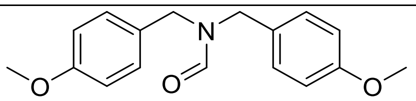	30	33	24

[a]Reaction conditions: **1** (1 mol%), K_2CO_3 (10 mol%), toluene (2 mL), 18 h, 150 °C. Conversion and amine yield were determined by ^{1}H NMR spectroscopy using 1,3,5-trimethoxybenzene as an internal standard. CO yield determined by analysing headspace by GC-TCD analysis.

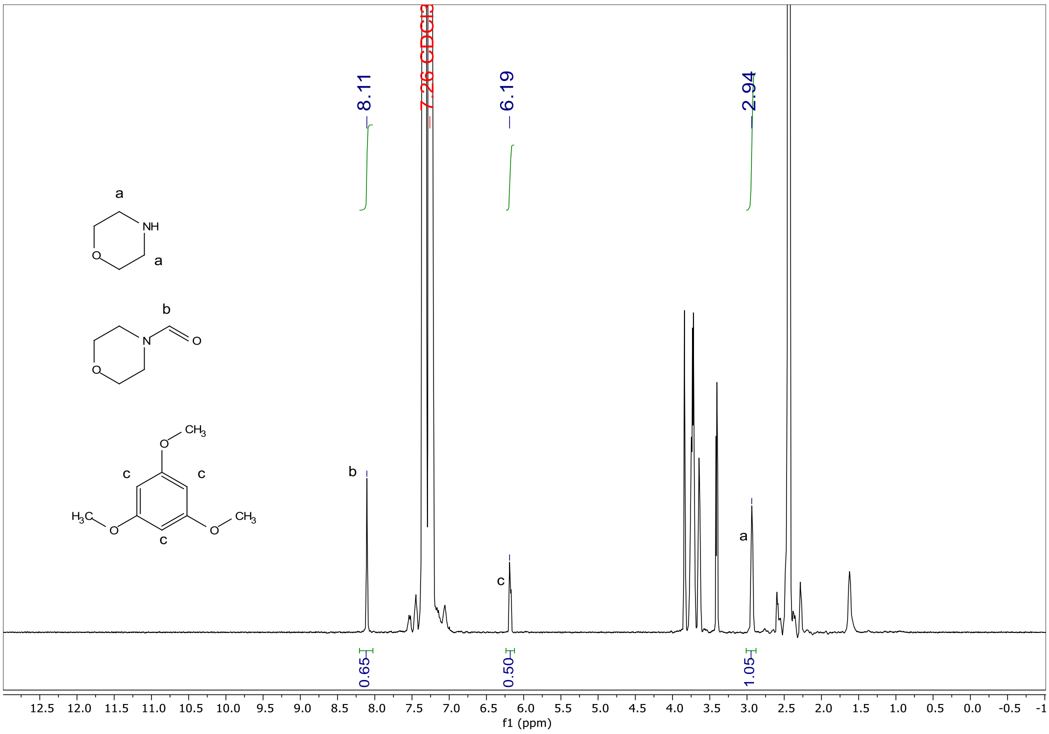

Figure S30: ¹H NMR spectrum ($CDCl_3$, 400 MHz, 298 K) corresponding to Table S3, entry 1.

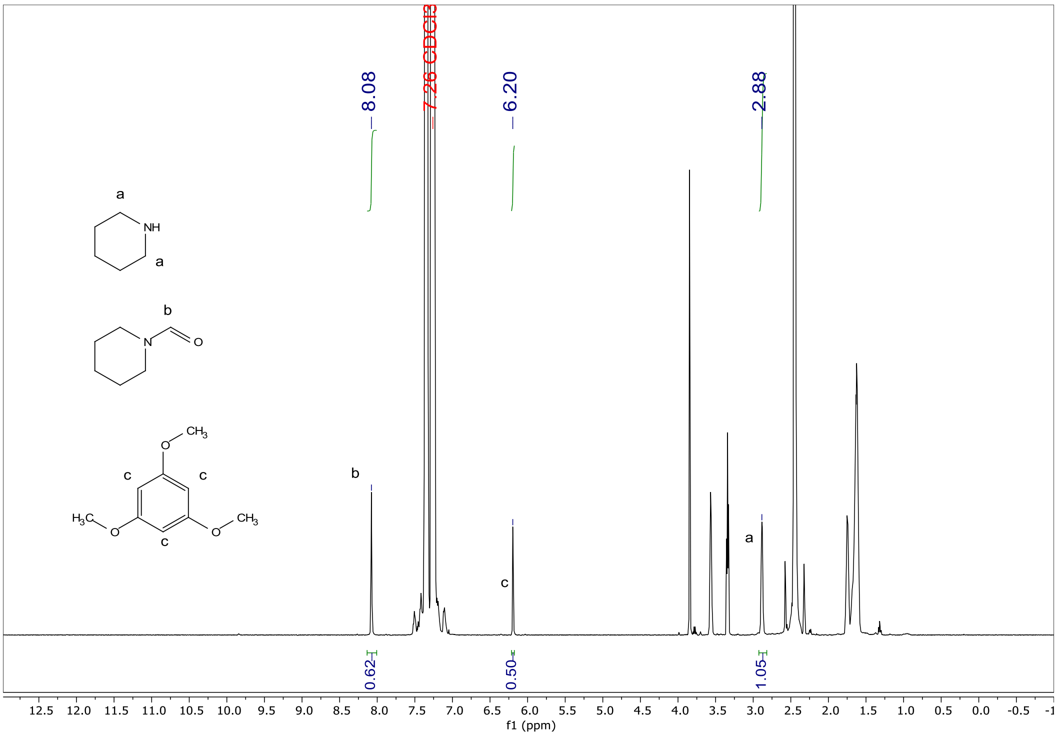

Figure S31: ¹H NMR spectrum ($CDCl_3$, 400 MHz, 298 K) corresponding to Table S3, entry 2.

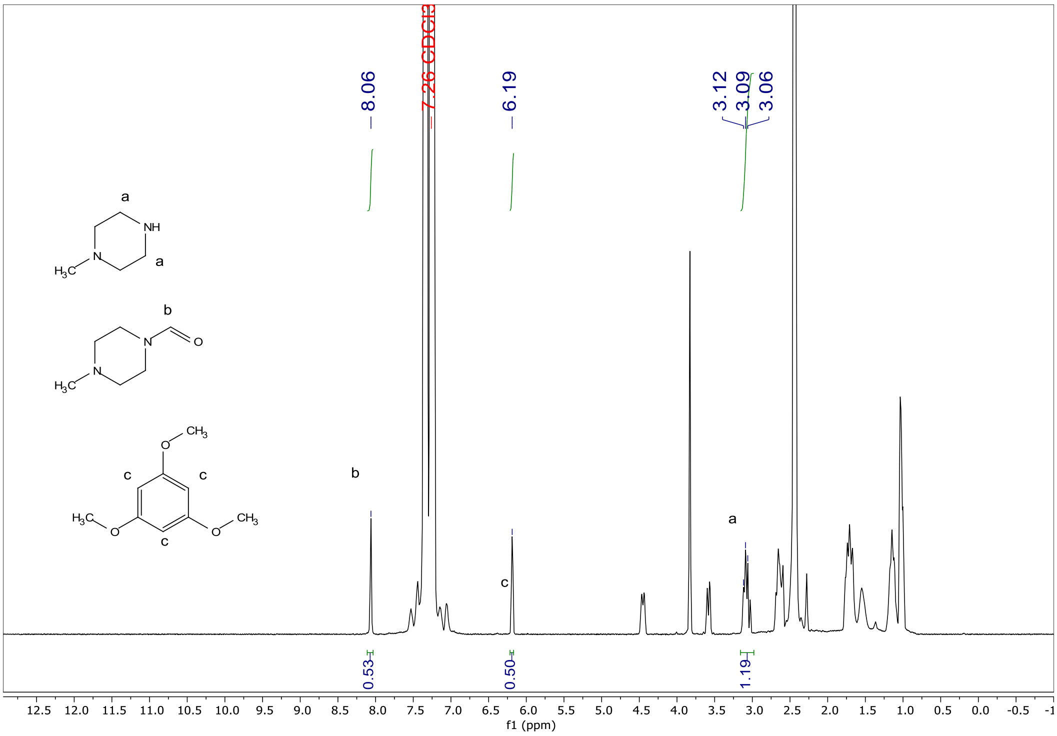

Figure S32: ^{1}H NMR spectrum ($CDCl_3$, 400 MHz, 298 K) corresponding to Table S3, entry 3.

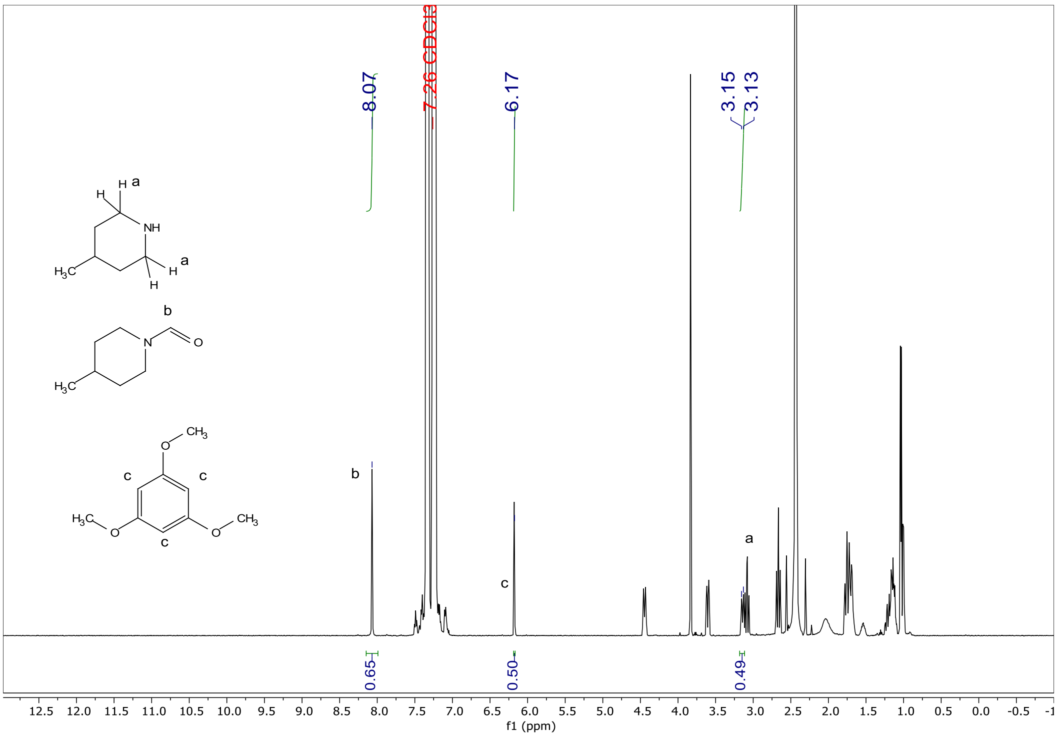

Figure S33: ^{1}H NMR spectrum ($CDCl_3$, 400 MHz, 298 K) corresponding to Table S3, entry 4.

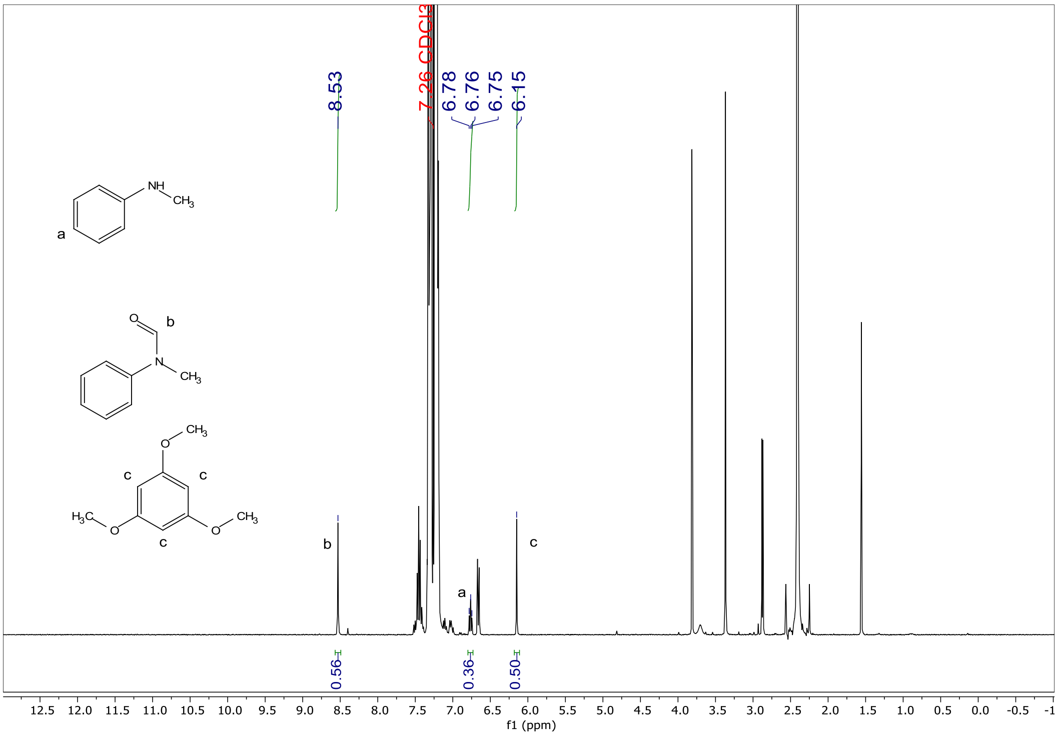

Figure S34: ^{1}H NMR spectrum ($CDCl_3$, 400 MHz, 298 K) corresponding to Table S3, entry 5.

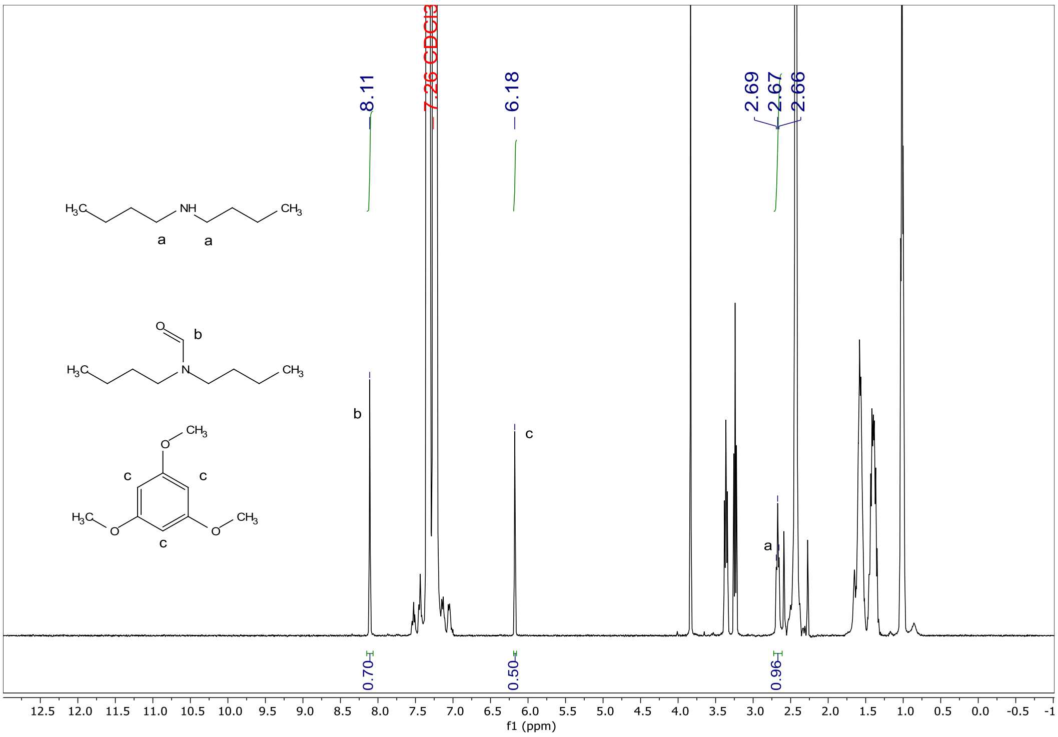

Figure S35: ^{1}H NMR spectrum ($CDCl_3$, 400 MHz, 298 K) corresponding to Table S3, entry 6.

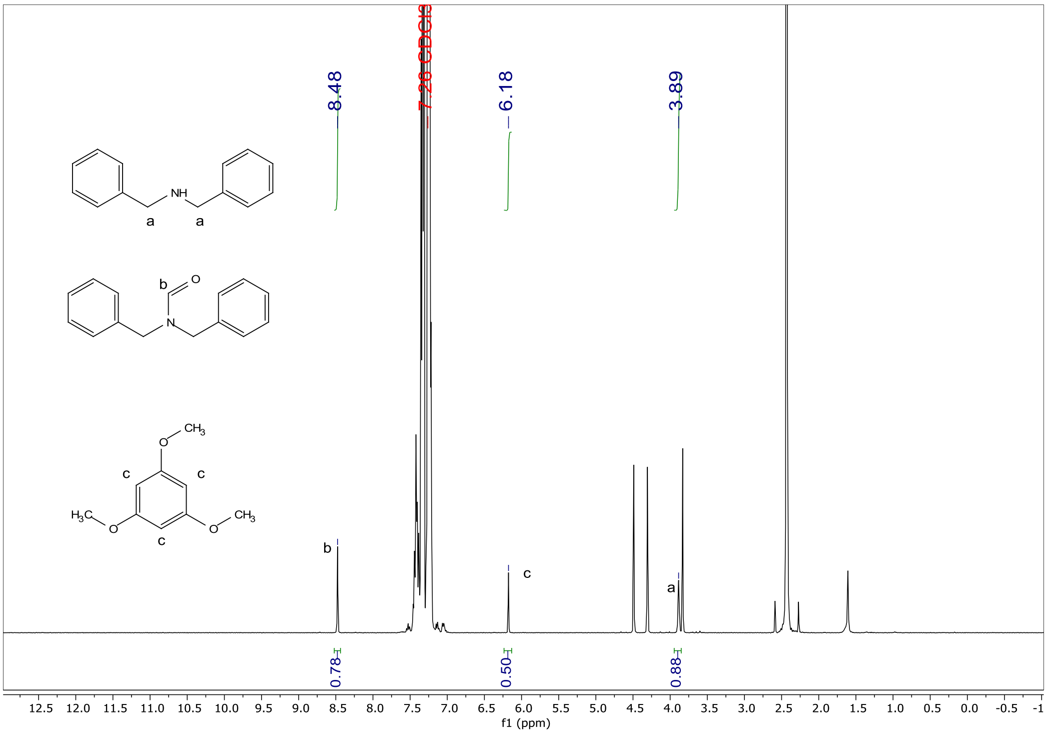

Figure S36: ^{1}H NMR spectrum ($CDCl_3$, 400 MHz, 298 K) corresponding to Table S3, entry 7.

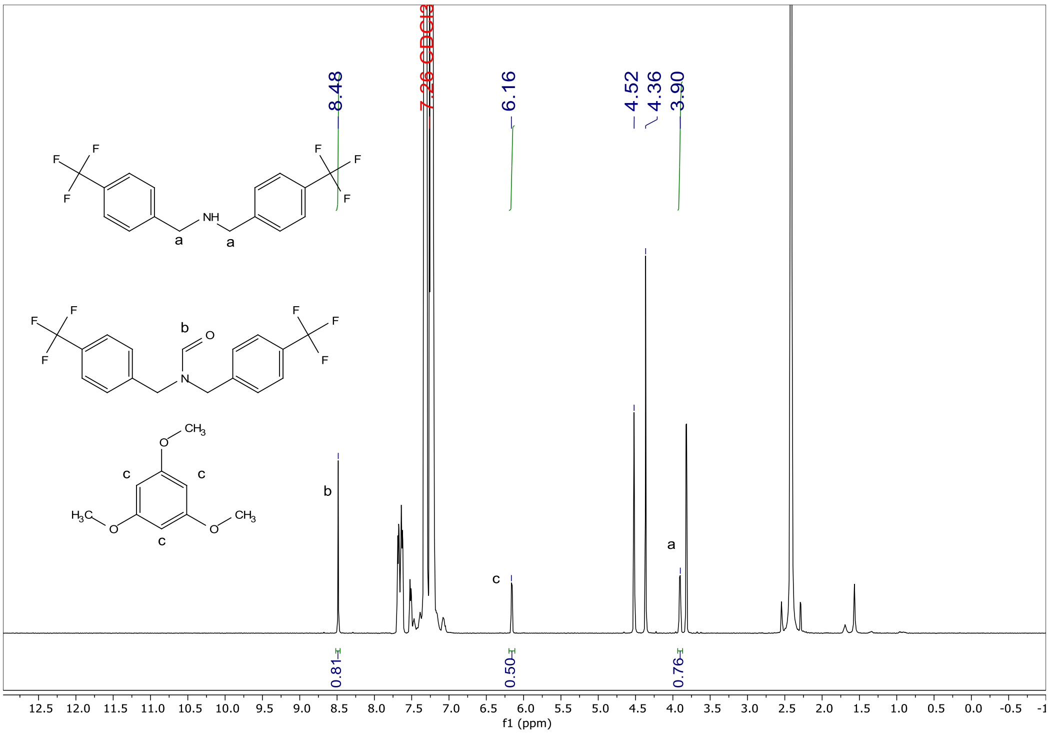

Figure S37: ^{1}H NMR spectrum ($CDCl_3$, 400 MHz, 298 K) corresponding to Table S3, entry 8.

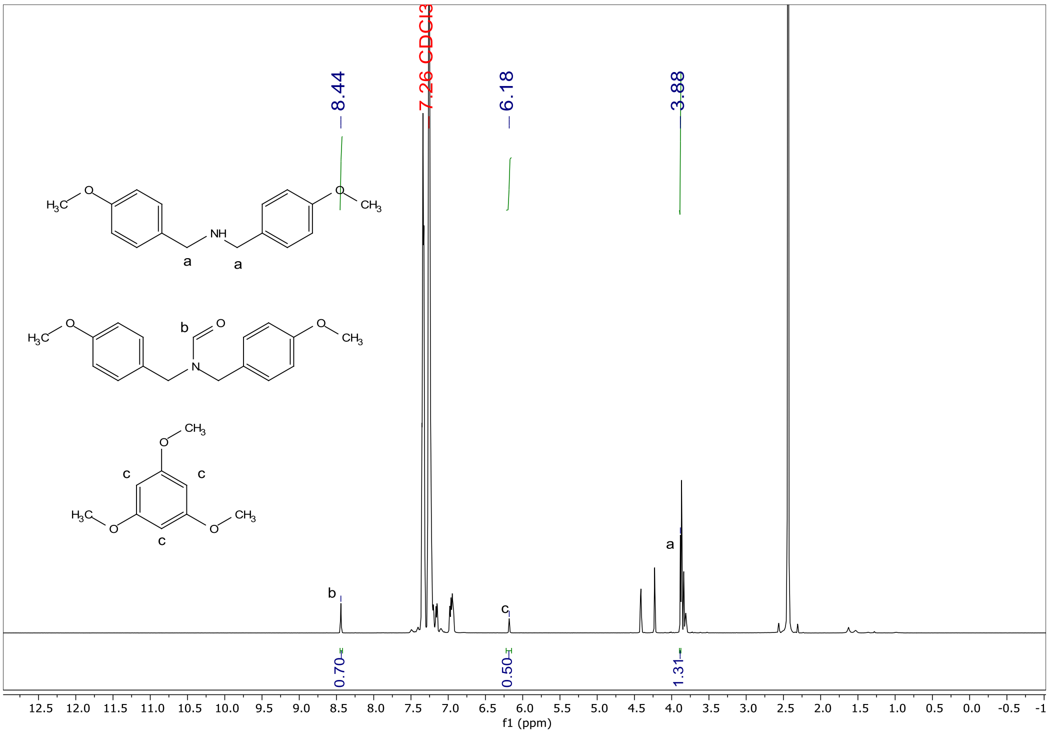

Figure S38: ¹H NMR spectrum (CDCl₃, 400 MHz, 298 K) corresponding to Table S3, entry 9.

2.6.4. NMR data acquired from the decarbonylation of *N*-formyl morpholine at different concentrations

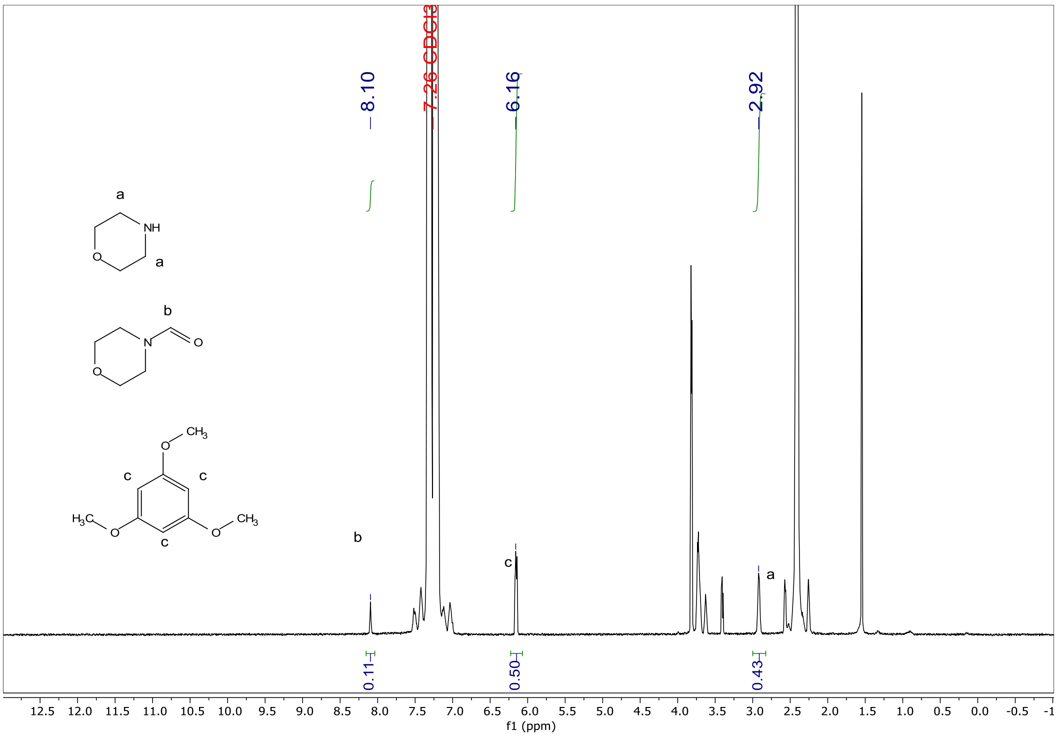

Figure S39: ¹H NMR spectrum (CDCl₃, 400 MHz, 298 K) corresponding to decarbonylation corresponding to N-formyl morpholine (0.125 M).

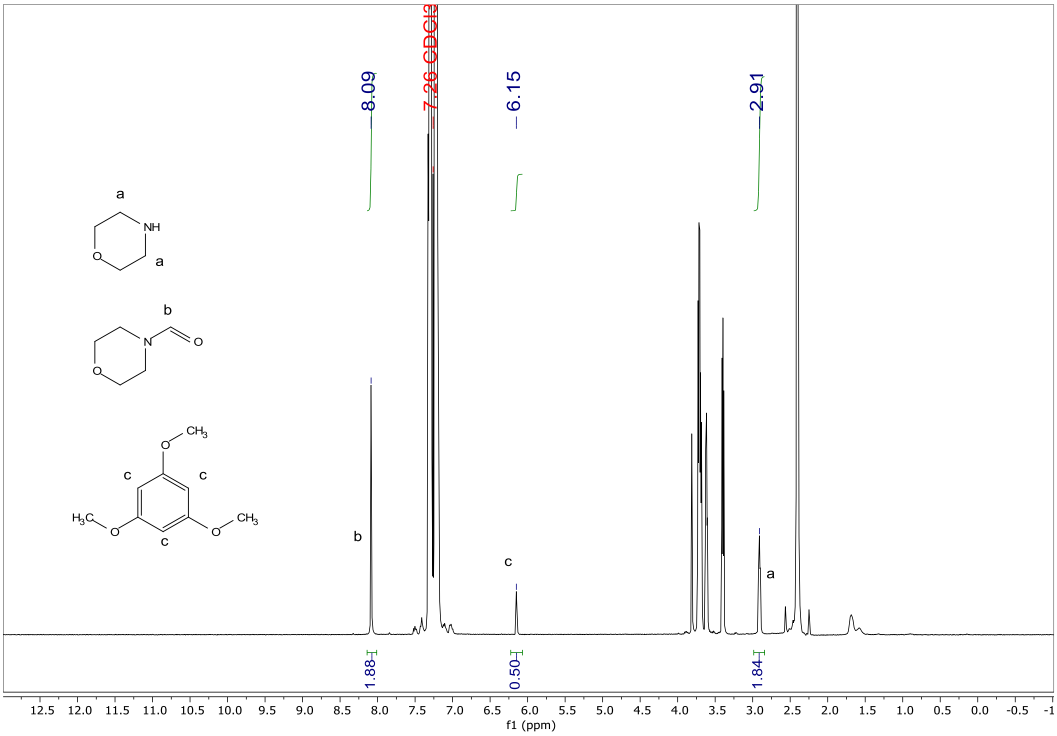

Figure S40: ¹H NMR spectrum ($CDCl_3$, 400 MHz, 298 K) corresponding to decarbonylation corresponding to N-formyl morpholine (1.25 M).

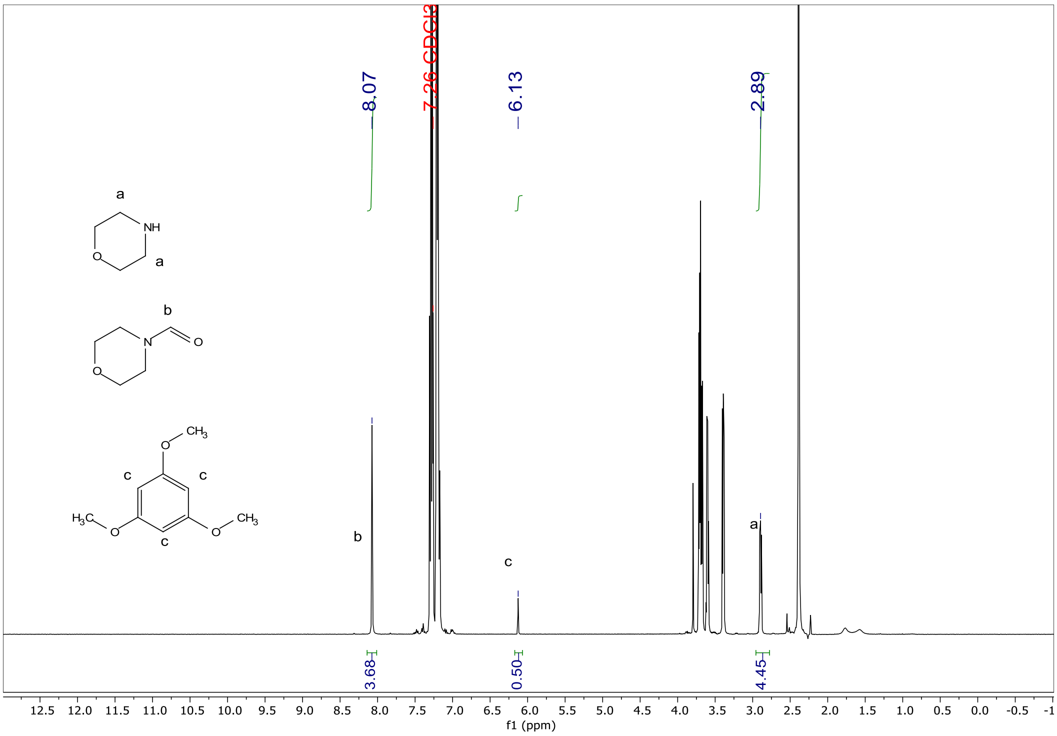

Figure S41: ¹H NMR spectrum ($CDCl_3$, 400 MHz, 298 K) corresponding to decarbonylation corresponding to N-formyl morpholine (2.5 M).

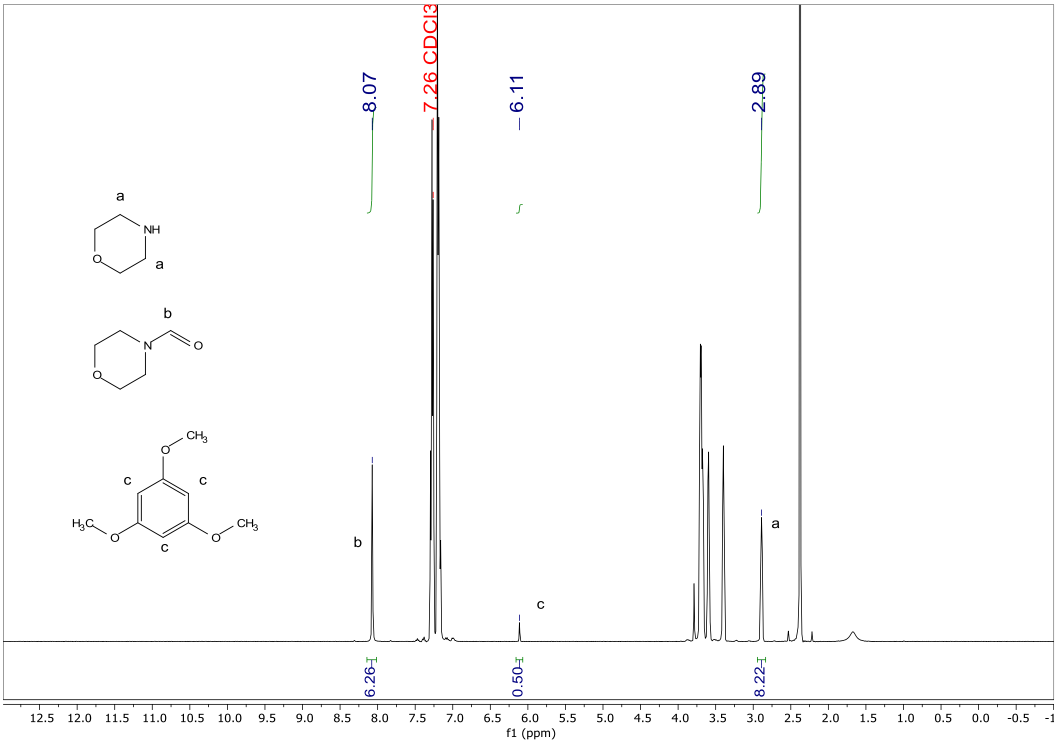

Figure S42: ¹H NMR spectrum (CDCl₃, 400 MHz, 298 K) corresponding to decarbonylation corresponding to N-formyl morpholine (4.6 M).

2.6.5. NMR data acquired from the sequential formylation and decarbonylation of morpholine

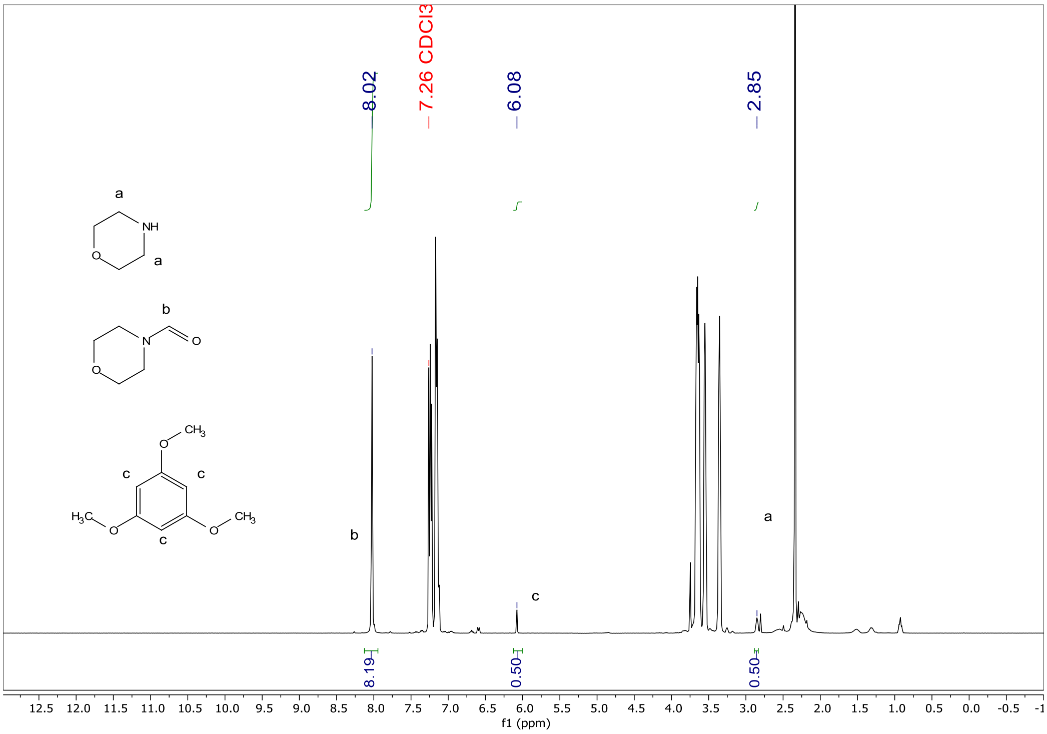

Figure S43: ¹H NMR spectrum (CDCl₃, 400 MHz, 298 K) corresponding to sequential formylation/decarbonylation of morpholine (with no drying).

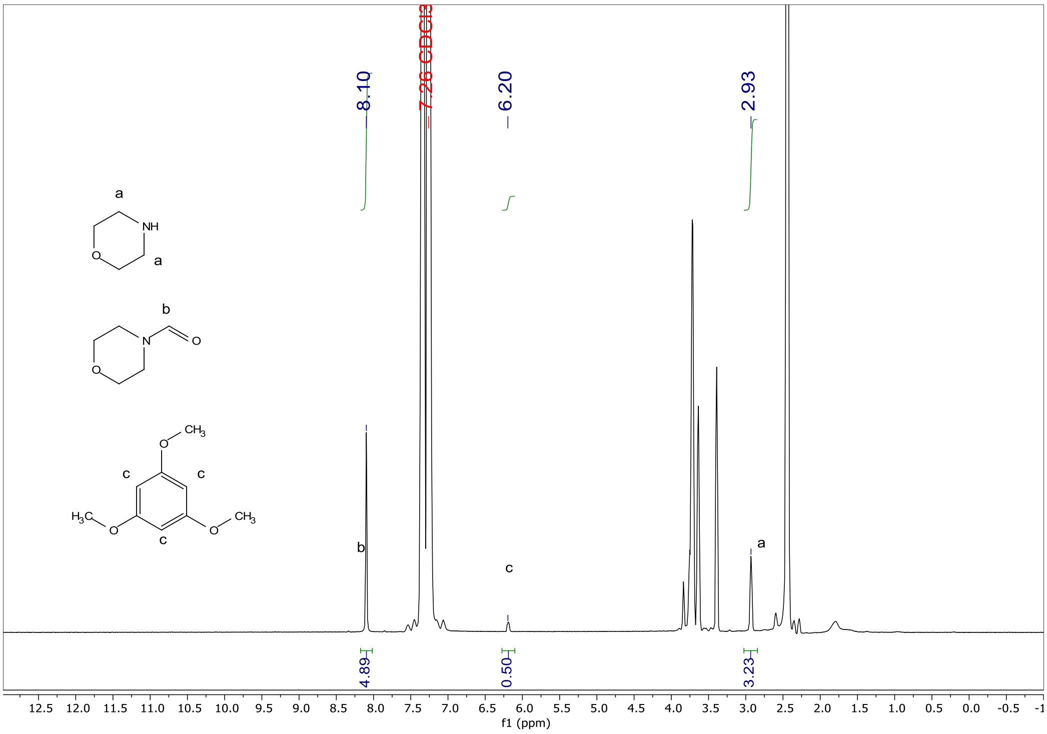

Figure S44: ^{1}H NMR spectrum ($CDCl_3$, 400 MHz, 298 K) corresponding to sequential formylation/decarbonylation of morpholine (with drying).

2.6.6. NMR data acquired from one-pot reverse water gas shift reaction

Table S4: One-step RWGS reaction.[a]

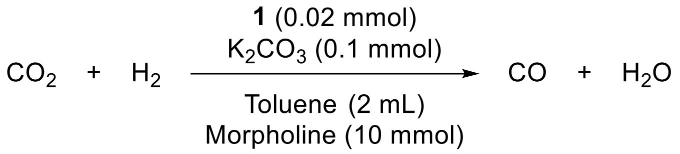

Entry	Time	T (°C)	TON	CO selectivity
**1**	18	150	17	100%
**2**	18	170	20	100%
**3**	90	170	249	100%
**4[b]**	18	150	0	-
**5[c]**	18	150	0	-

[a]standard reaction conditions: **1** (0.02 mmol), K_2CO_3 (0.1 mmol), toluene (2 mL), morpholine (10 mmol), 70 bar (1:1 H_2/CO_2). CO yield and selectivity was determined by GC-TCD analysis. [b]The reaction was performed in the absence of **1**. [c]The reaction was performed in the absence of morpholine.

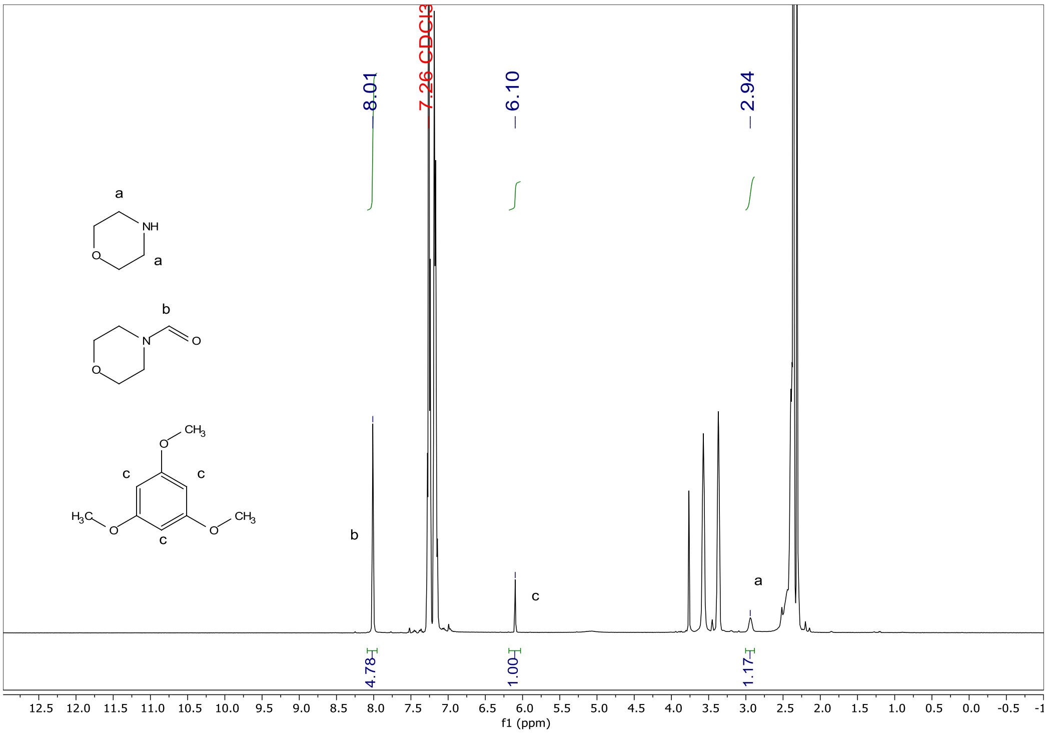

Figure S45: ^{1}H NMR spectrum ($CDCl_3$, 400 MHz, 298 K) corresponding to Table 3, entry 1.

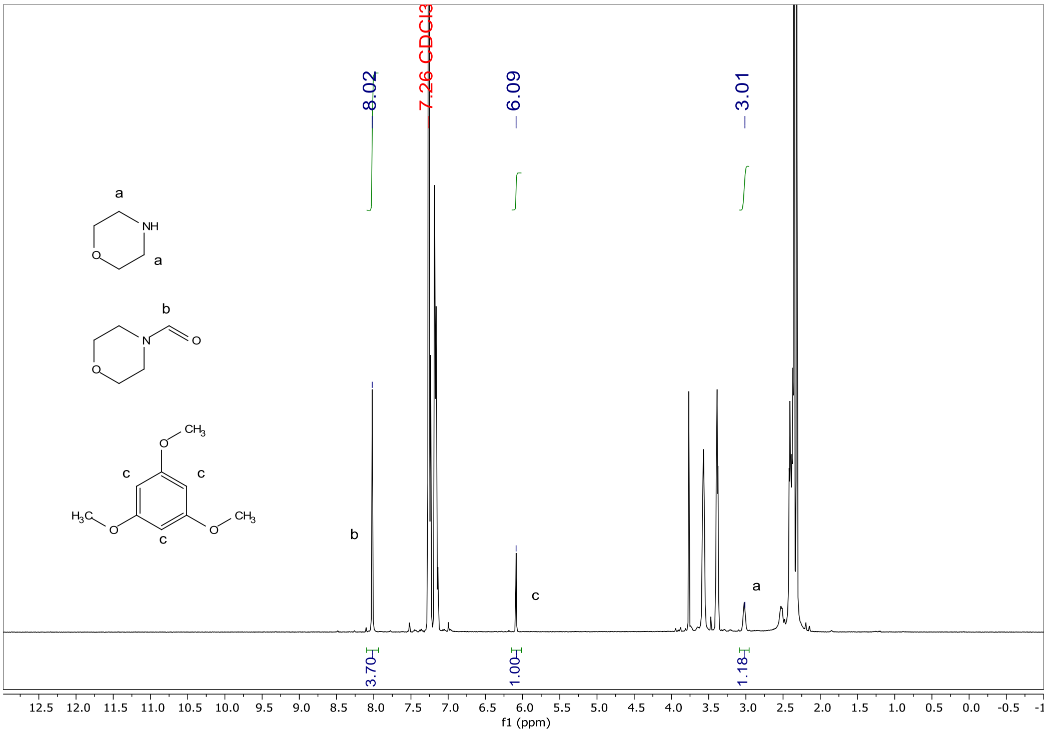

Figure S46: ^{1}H NMR spectrum ($CDCl_3$, 400 MHz, 298 K) corresponding to Table 3, entry 2.

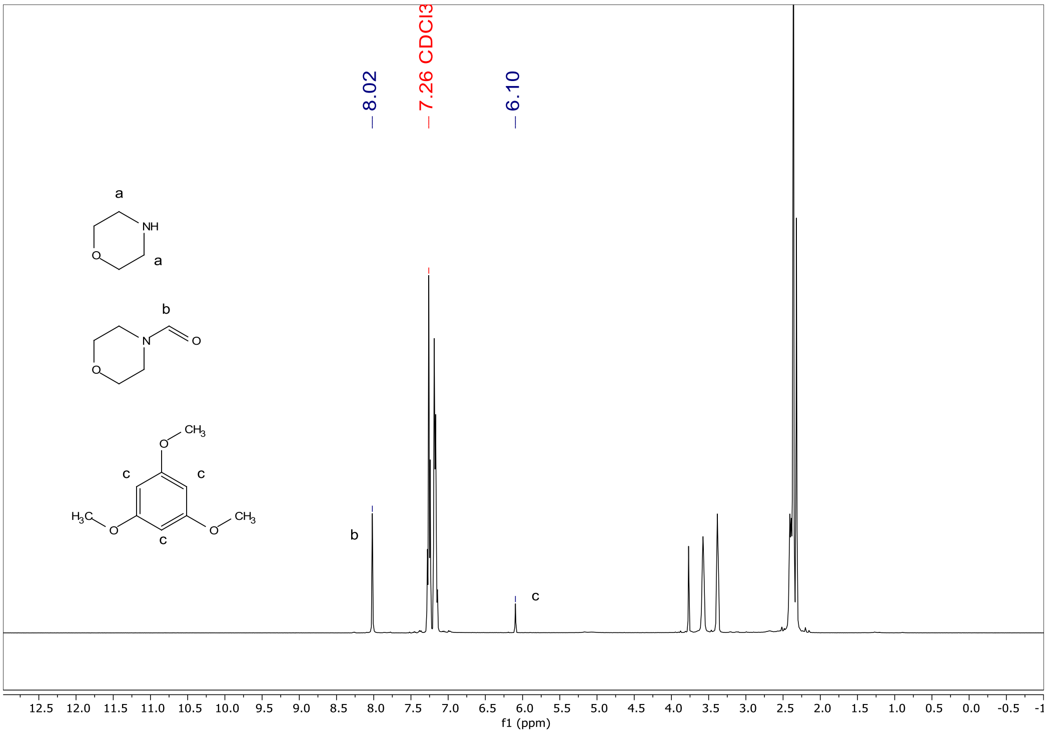

Figure S47: ^{1}H NMR spectrum ($CDCl_3$, 400 MHz, 298 K) corresponding to Table 3, entry 3.

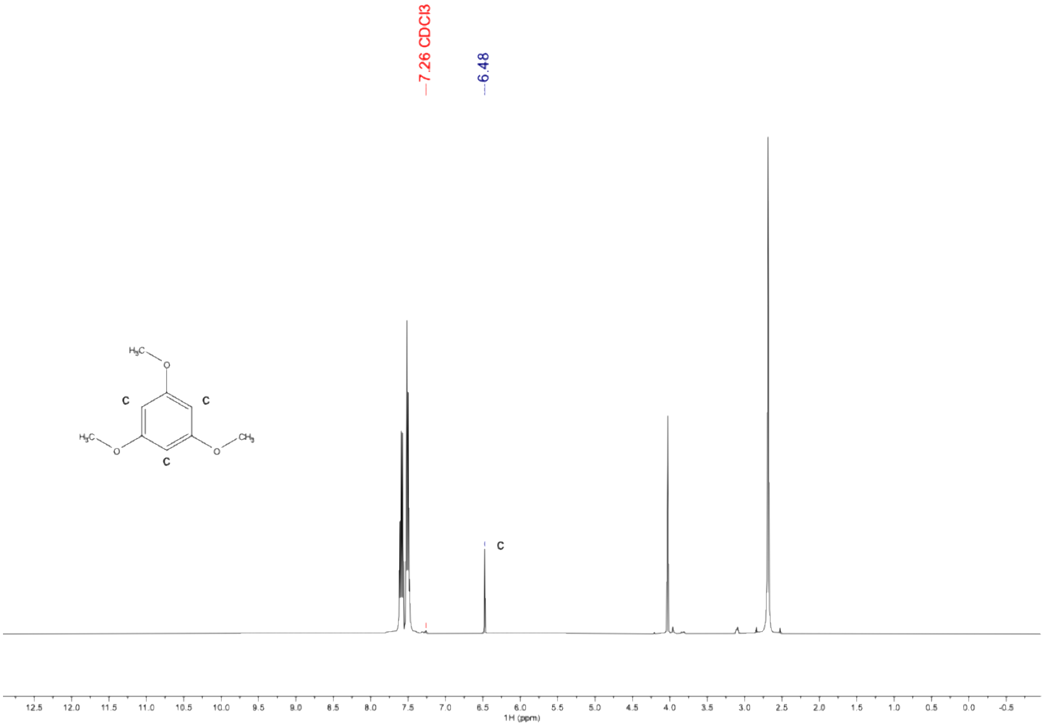

Figure S48: ^{1}H NMR spectrum ($CDCl_3$, 400 MHz, 298 K) corresponding to Table 3, entry 4.

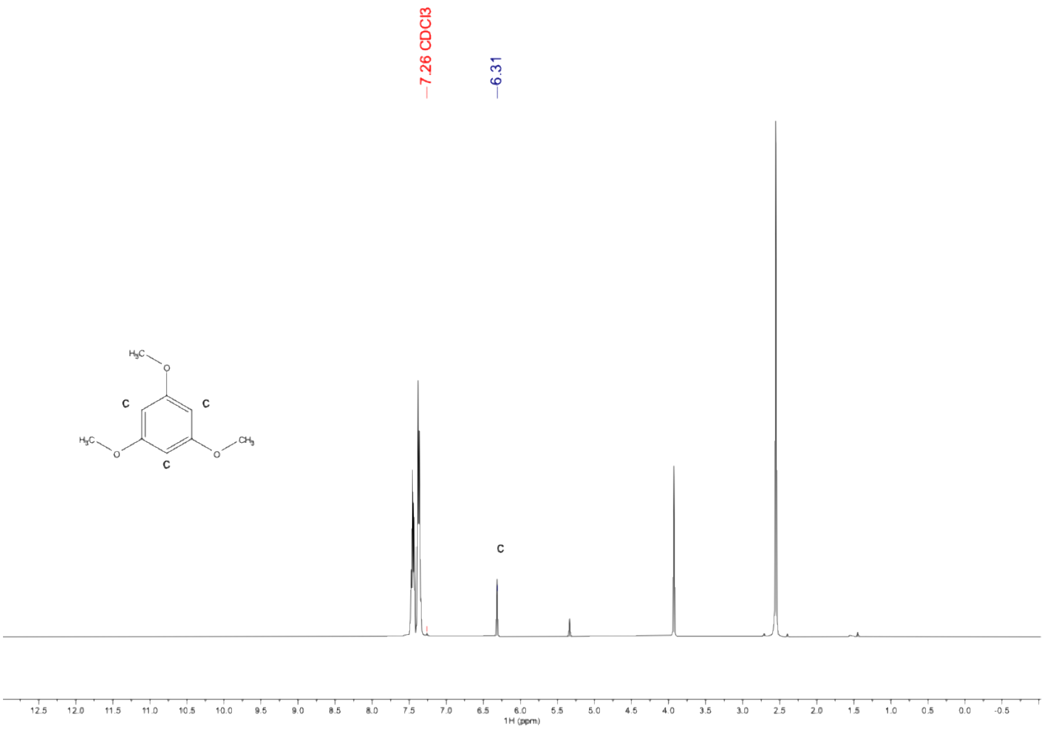

Figure S49: ^{1}H NMR spectrum ($CDCl_3$, 400 MHz, 298 K) corresponding to Table 3, entry 5.

2.7. Characterisation data acquired for mechanistic study

2.7.1. NMR data from mechanistic study

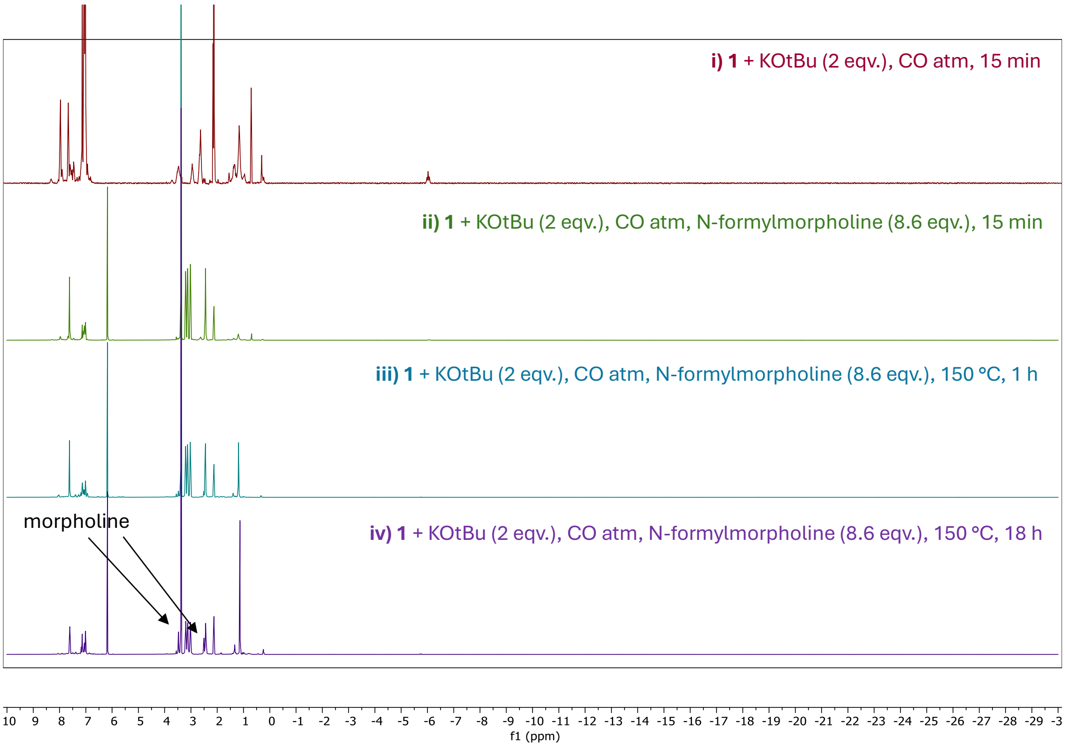

Figure S50: ^{1}H NMR spectra corresponding to complex **1D** and subsequent reaction with *N*-formylmorpholine (**i** – **1** (0.01 mmol), KO*t*Bu (0.02 mmol), CO atm, 15 min, **ii** – **i** + N-formylmorpholine (0.086 mmol), 15 min, **iii** – **ii** + 150 °C, 1 h, **iv** – **iii** + 150 °C, 17 h).

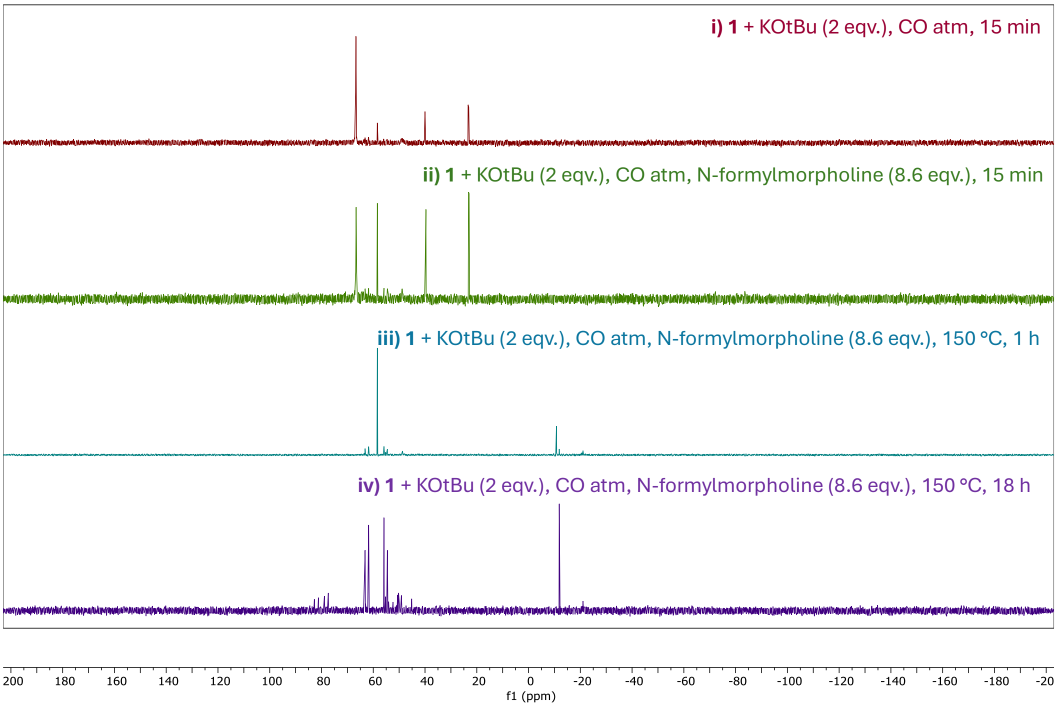

Figure S51: ^{31}P{^{1}H} NMR spectra corresponding to complex **1D** and subsequent reaction with *N*-formylmorpholine (**i** – **1** (0.01 mmol), KO*t*Bu (0.02 mmol), CO atm, 15 min, **ii** – **i** + N-formylmorpholine (0.086 mmol), 15 min, **iii** – **ii** + 150 °C, 1 h, **iv** – **iii** + 150 °C, 17 h).

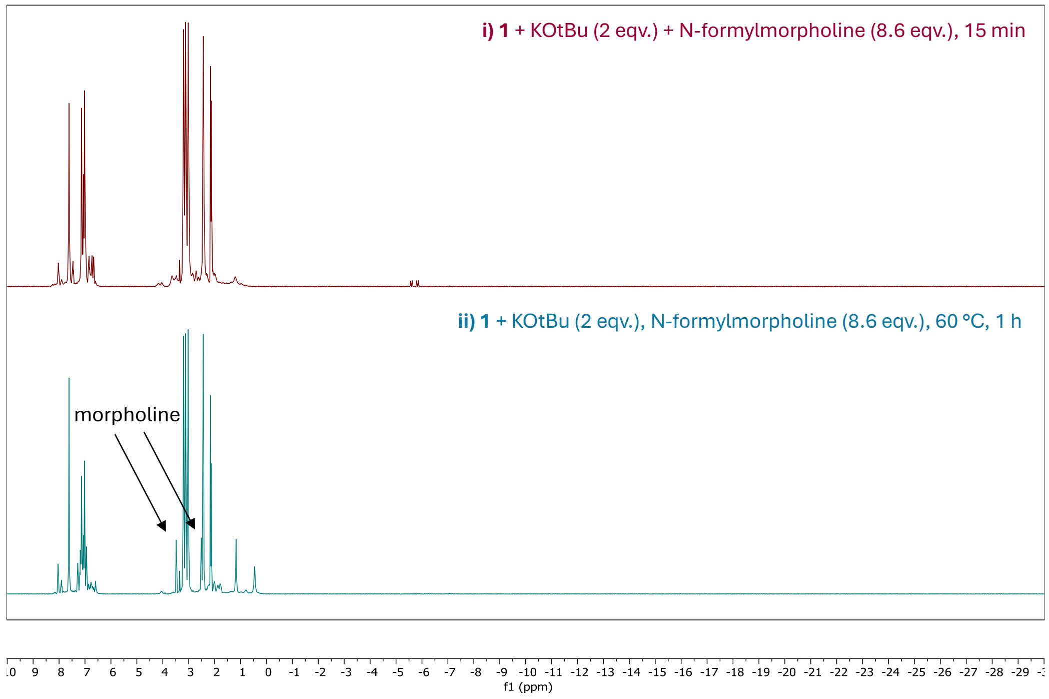

Figure S52: ^{1}H NMR spectra corresponding to complex **1C** and subsequent reaction heating 60 °C, 1 h (**i** – **1** (0.01 mmol), KO*t*Bu (0.02 mmol), N-formylmorpholine (0.086 mmol) RT, 15 min (for expansion, see Figure S52A), **ii** – **i** + 60 °C, 1h).

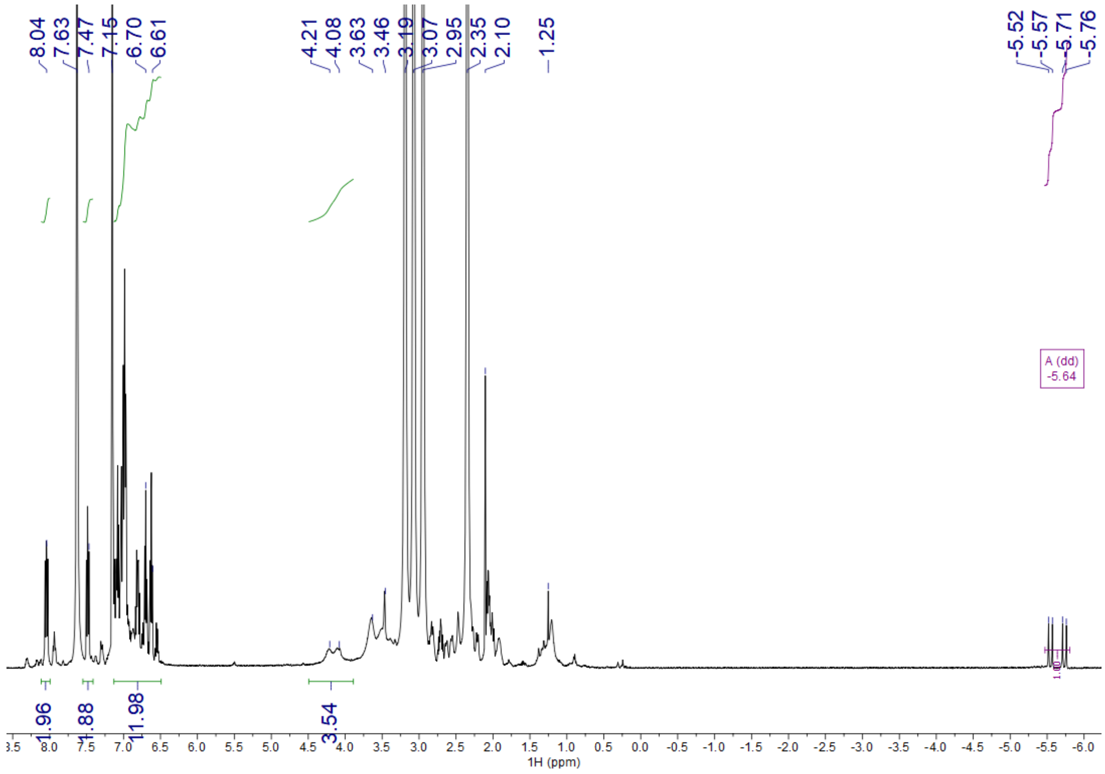

Figure S52A: Crude ^{1}H NMR spectr*um for the formation of* complex **1C** (**1** (0.01 mmol), KO*t*Bu (0.02 mmol), N-formylmorpholine (0.086 mmol) 15 min, RT).

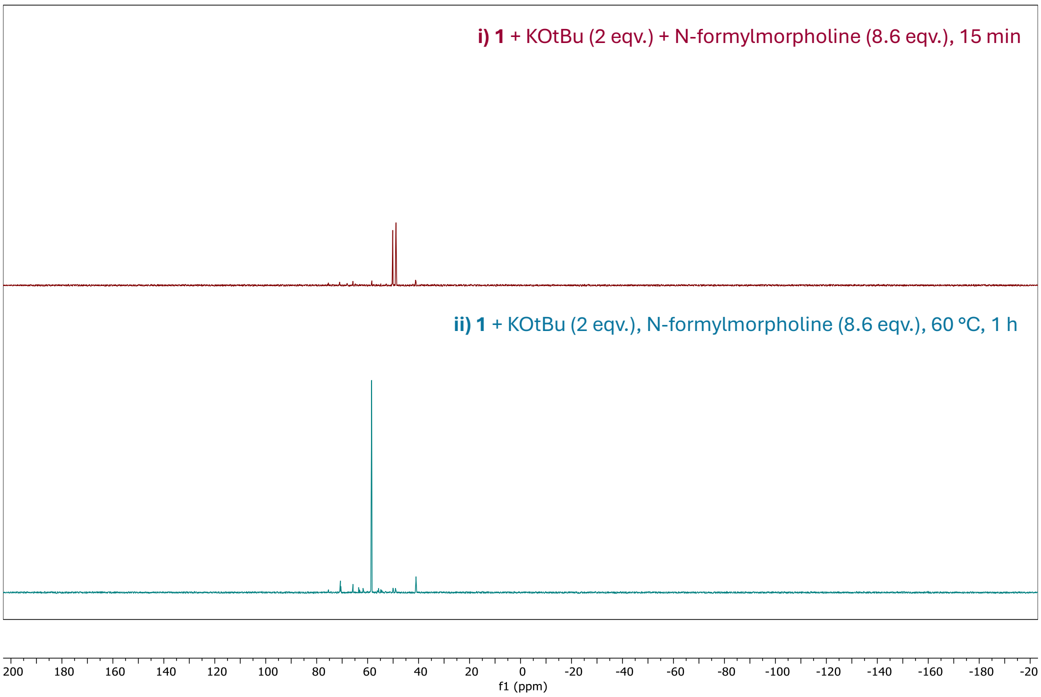

Figure S53: $^{31}P\{^{1}H\}$ NMR spectra corresponding to complex **1C** and subsequent reaction heating 60 °C, 1 h (**i** – **1** (0.01 mmol), KO*t*Bu (0.02 mmol), N-formylmorpholine (0.086 mmol) RT, 15 min (for expansion, see Figure S53A), **ii** – **i** + 60 °C, 1h).

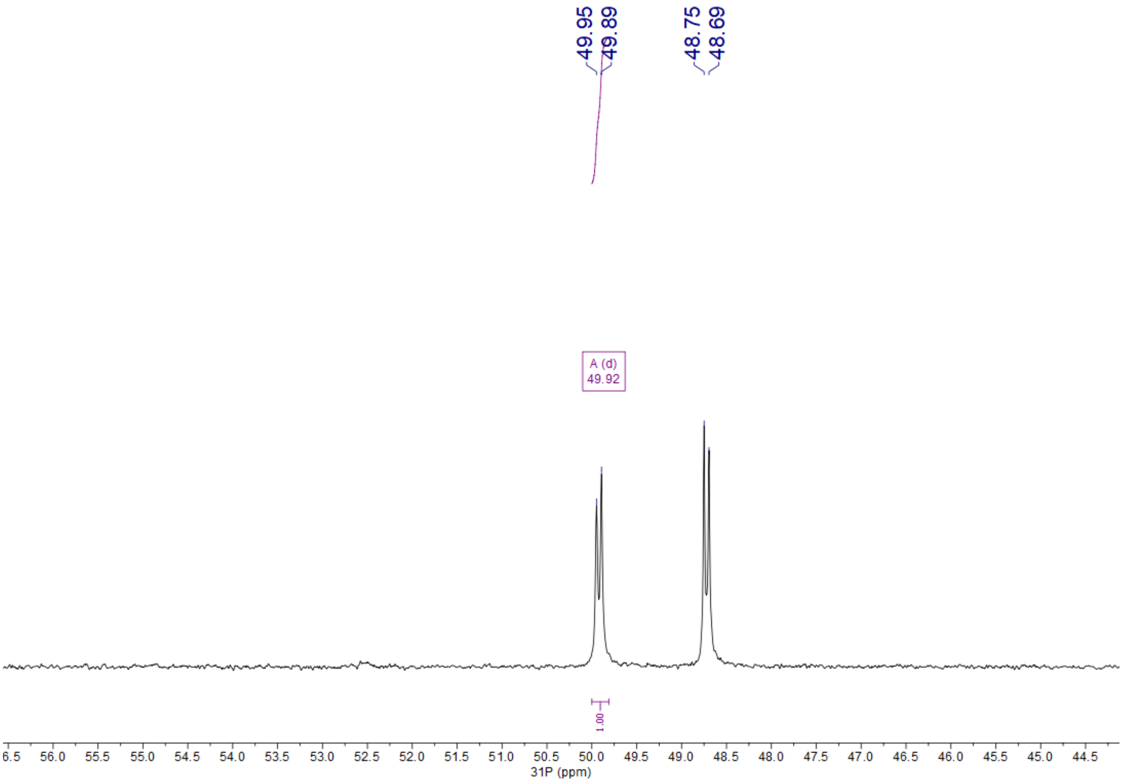

Figure S53A: Crude : $^{31}P\{^{1}H\}$ NMR spectrum *for the formation of complex 1C* (**1** (0.01 mmol), KO*t*Bu (0.02 mmol), N-formylmorpholine (0.086 mmol) 15 min, RT).

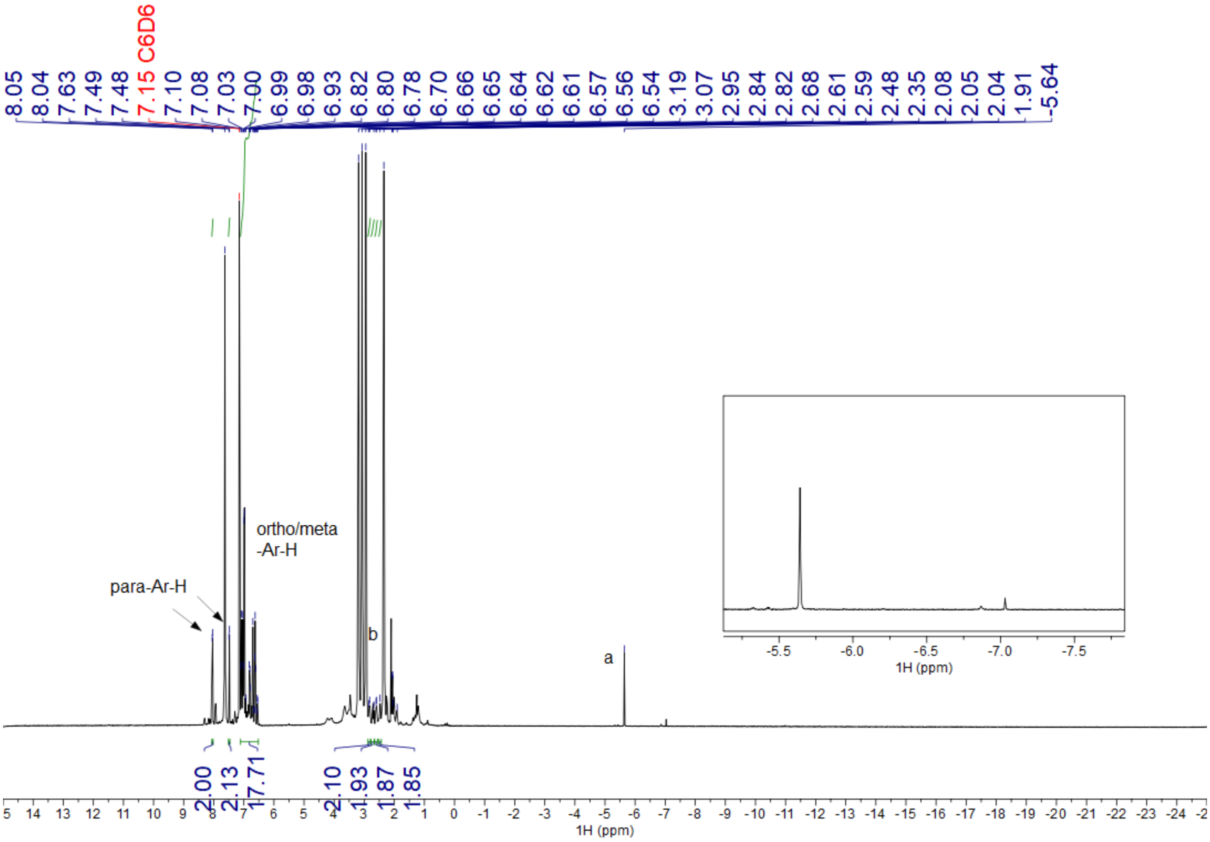

Figure S54: $^{1}H\{^{31}P\}$ NMR spectrum corresponding to complex **1C** (**1** (0.01 mmol), KO*t*Bu (0.02 mmol), N-formylmorpholine (0.086 mmol) 15 min, RT).

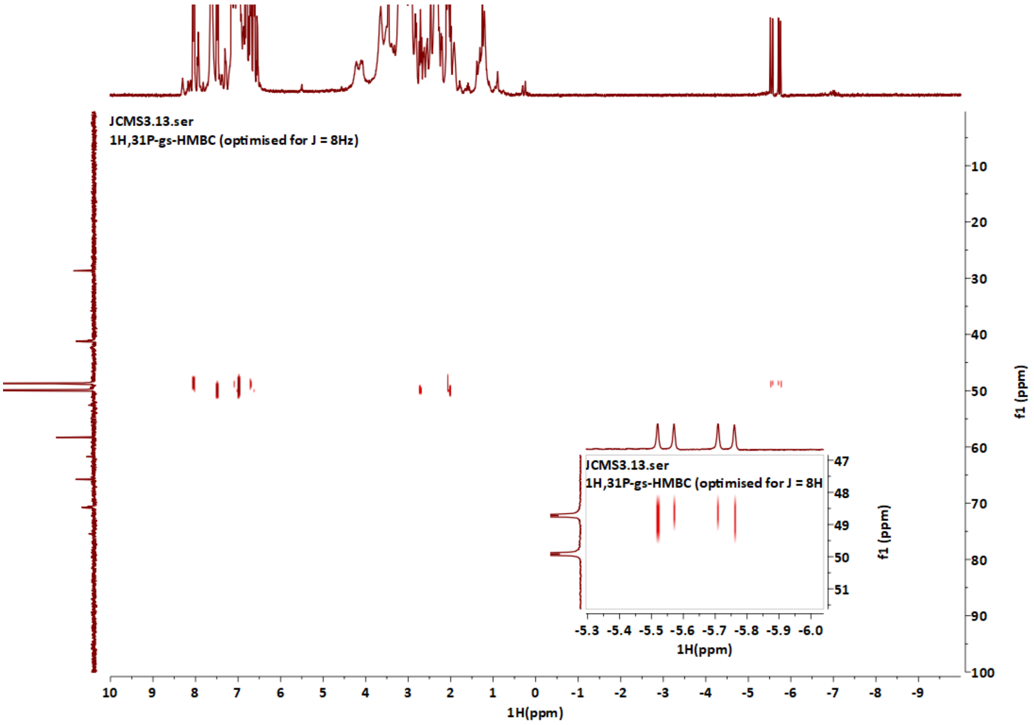

Figure S55: ^{1}H, ^{31}P HMBC NMR spectrum corresponding to complex **1C** (**1** (0.01 mmol), KO*t*Bu (0.02 mmol), N-formylmorpholine (0.086 mmol) 15 min, RT).

2.7.2. Crystallographic data

X-ray diffraction data for compound 1C were collected at 100 K using a Rigaku XtaLAB Synergy-S diffractometer equipped with a HyPix-Arc 100° Hybrid Photon Counting (HPC) detector, PhotonJet-S microfocus sealed-tube X-ray source [Cu Kα radiation (λ = 1.54187 Å)], and MAX optics. Data were collected (using a calculated strategy) and processed (including correction for Lorentz, polarization and absorption) using CrysAlisPro.[7] The structure was solved by dual-space methods (SHELXT[8]) and refined by full-matrix least-squares against F^2 (SHELXL-2019/3[9]). Non-hydrogen atoms were refined anisotropically, and hydrogen atoms were refined using a riding model except for the hydrogen atoms on Ru1 and N4 which were located from the difference Fourier map and refined isotropically, with the hydrogen on N4 subject to a distance restraint. The toluene solvate was positioned on an inversion point and disordered as a 180° flip. The methyl group (C44) and para- hydrogen were modelled in separate parts with their occupancy fixed at 0.5 (after the free refinement of the methyl converged on 0.5). All calculations were performed using the Olex2[10] interface. Selected crystallographic data: $C_{37.5}H_{42}N_2O_3P_2Ru$, M = 731.74, monoclinic, a = 10.62498(12), b = 20.5434(3), c = 15.44586(16) Å, β = 96.6191(10)°, U = 3348.94(7) Å^3, T = 100 K, space group $P2_1/n$ (no. 14), Z = 4, 58945 reflections measured, 6633 unique (R_{int} = 0.0292), which were used in all calculations. The final R_1 [$I > 2\sigma(I)$] was 0.0266 and wR_2 (all data) was 0.0650. CCDC 2516422 contains the supplementary crystallographic data for this paper. These data can be obtained free of charge from The Cambridge Crystallographic Data Centre via www.ccdc.cam.ac.uk/structures.

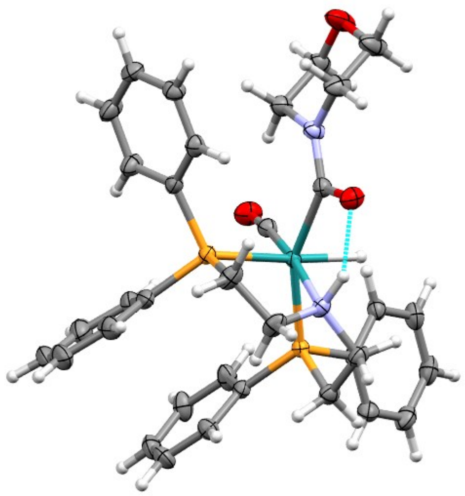

Figure S56: Single crystal X-ray structure of complex **1C**. ORTEP thermal ellipsoid plots plotted at 50% probability level.

3. ESI-MS data

3.1. ESI-MS data from mechanistic study

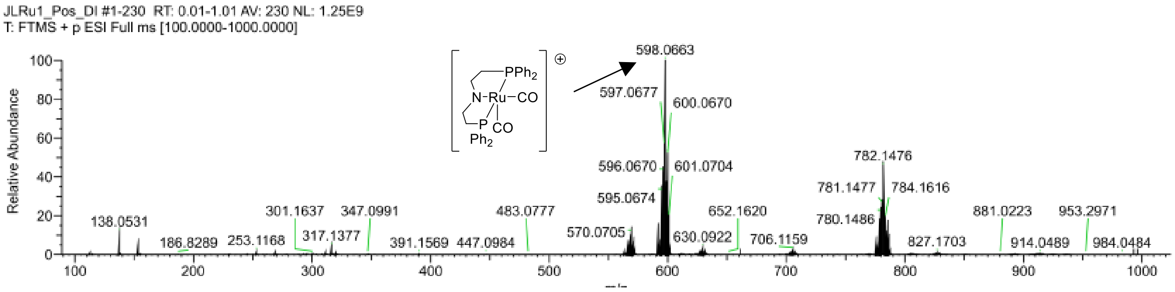

*Figure S57: ESI-MS of resultant reaction mixture of **1** (0.01 mmol), KOtBu (0.02 mmol), N-formylmorpholine (0.086 mmol), d_8-toluene (~0.5 mL), 18 h, 150 °C.*

4. GC-MS data

4.1.1. GC-MS data acquired from the formylation of amines

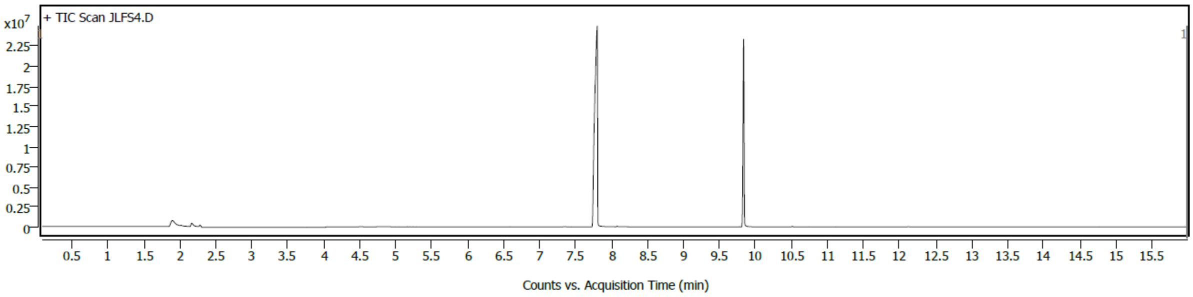

Figure S58: Gas chromatograph corresponding to Table S1, entry 1.

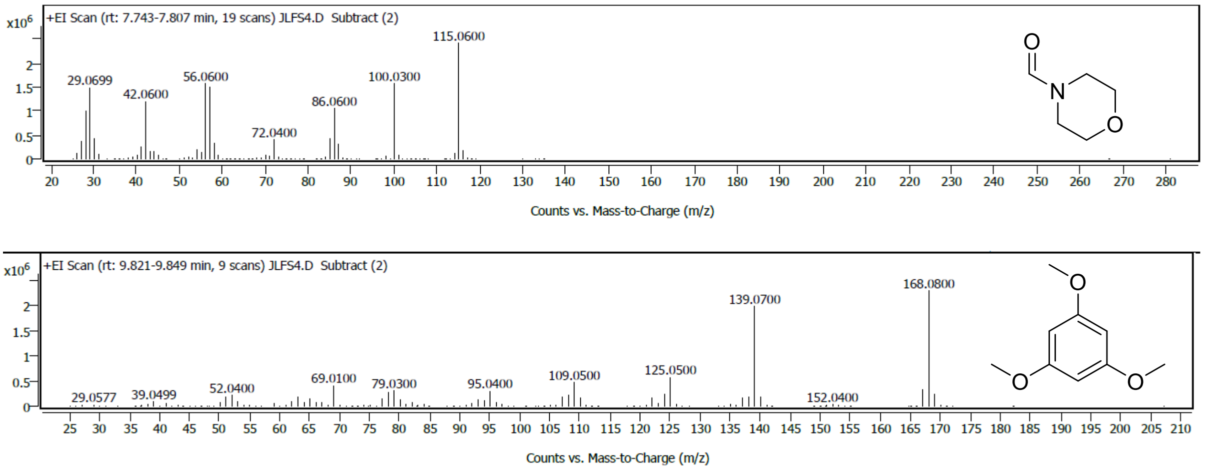

Figure S59: Mass spectrum corresponding to Figure S58.

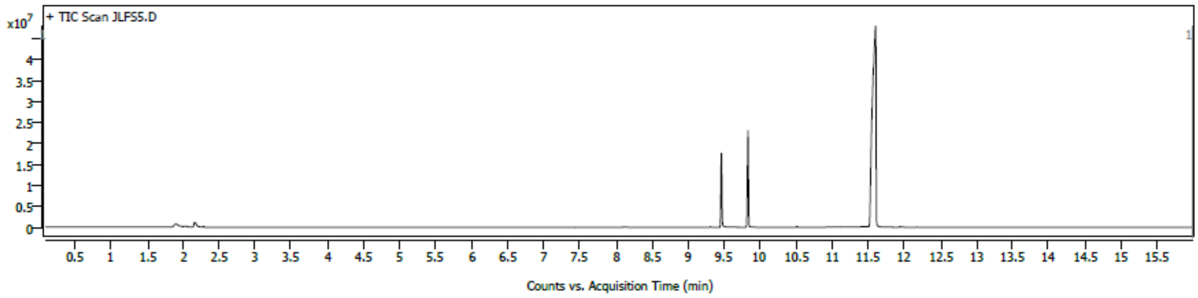

Figure S60: Gas chromatograph corresponding to Table S1, entry 2.

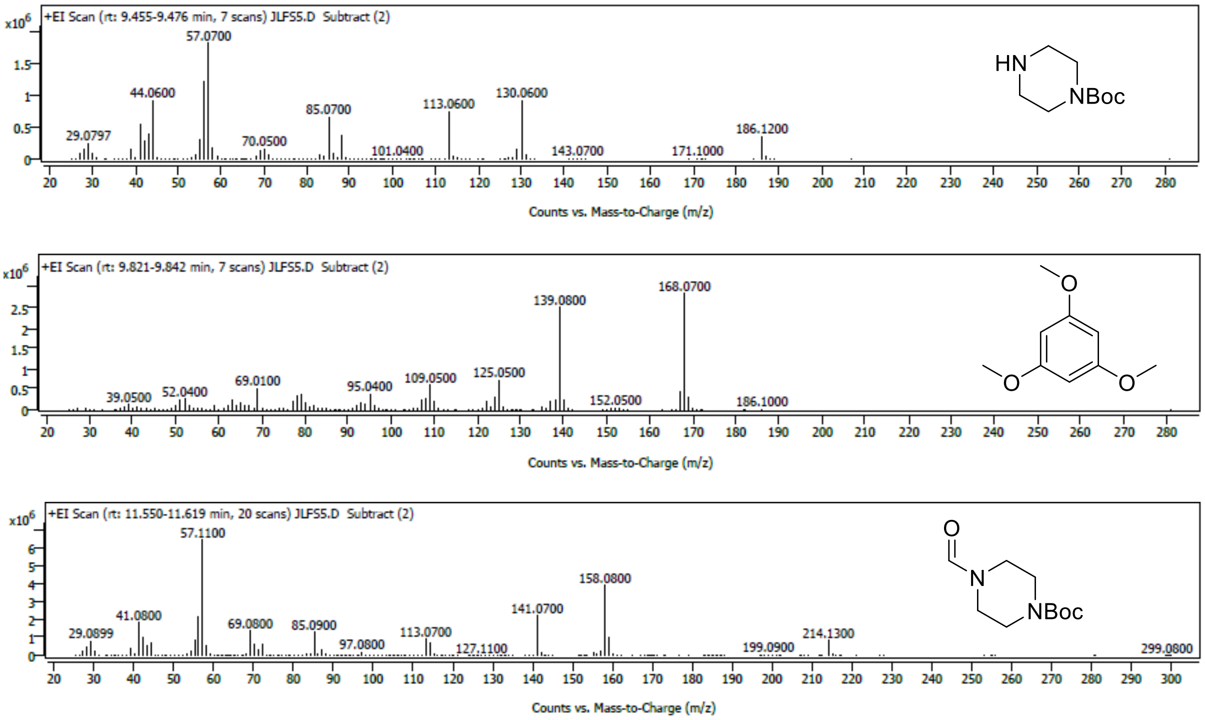

Figure S61: Mass spectrum corresponding to Figure S60.

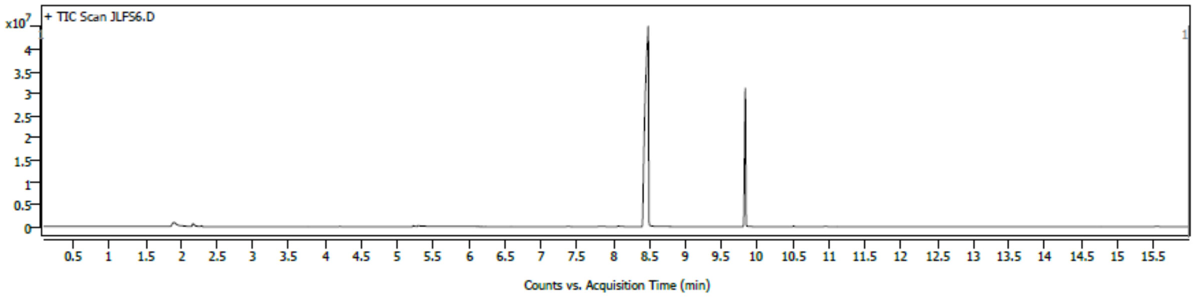

Figure S62: Gas chromatograph corresponding to Table S1, entry 3.

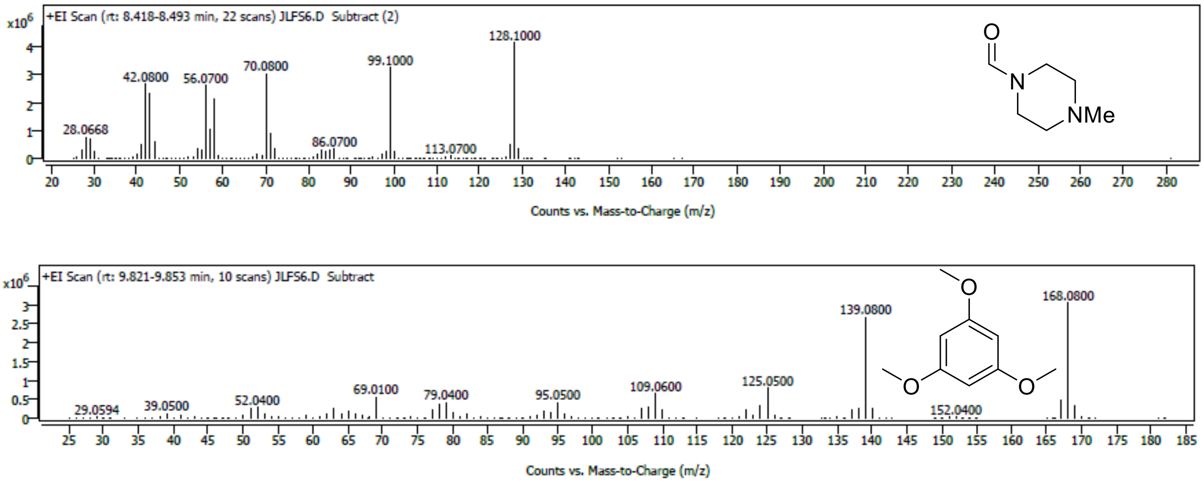

Figure S63: Mass spectrum corresponding to Figure S62.

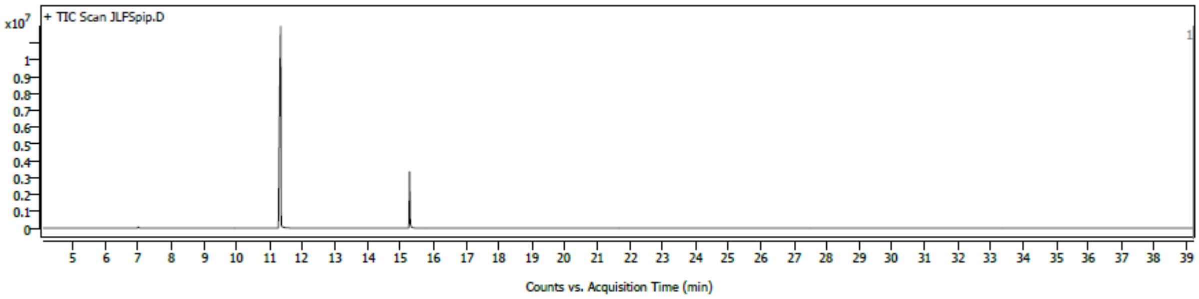

Figure S64: Gas chromatograph corresponding to Table S1, entry 4.

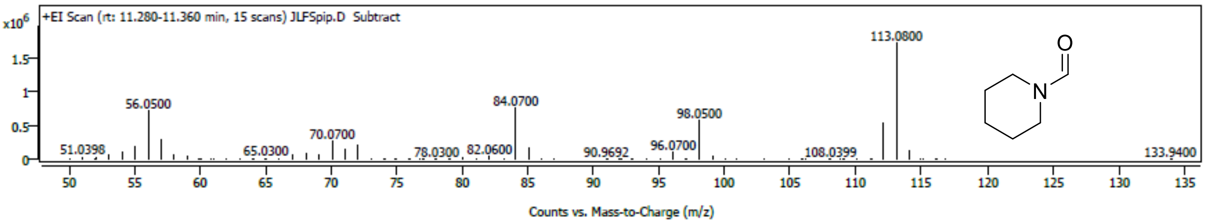

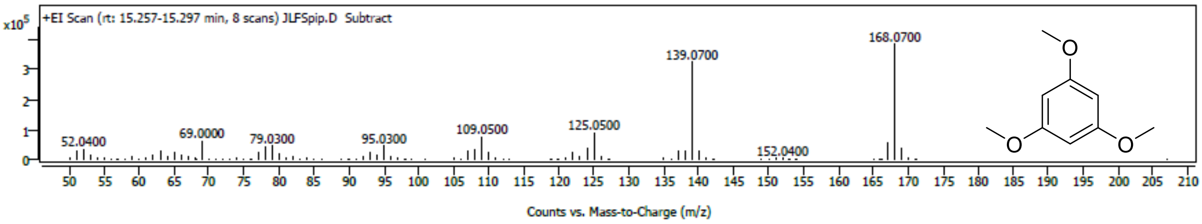

Figure S65: Mass spectrum corresponding to Figure S64.

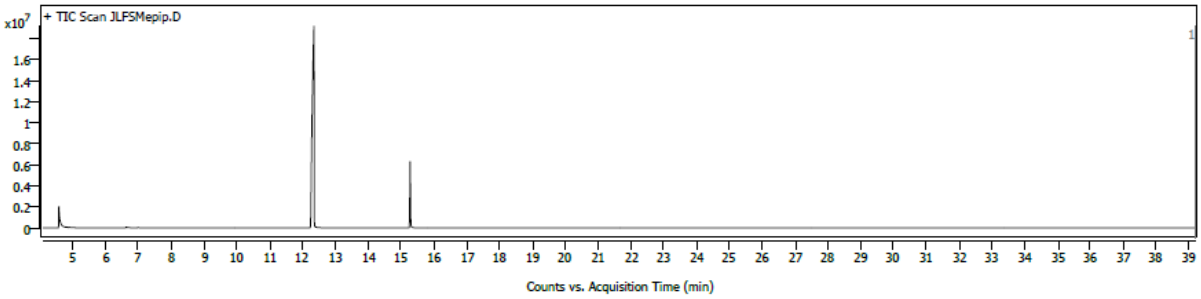

Figure S66: Gas chromatograph corresponding to Table S1, entry 5.

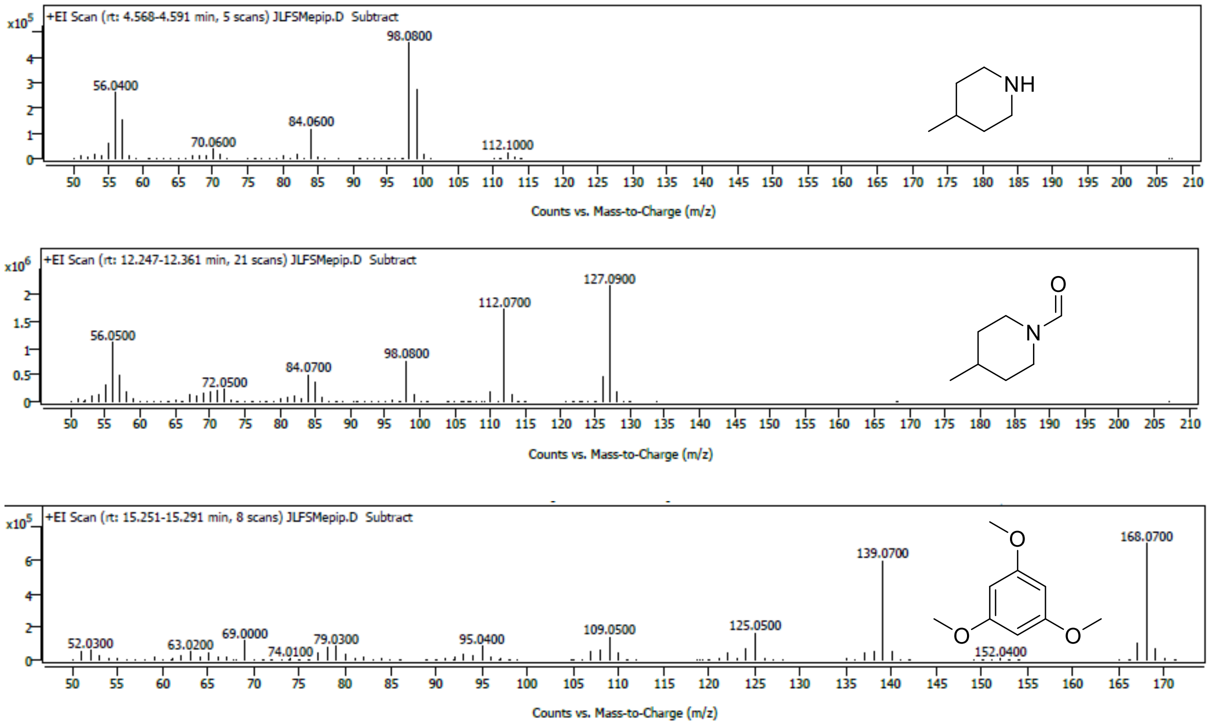

Figure S67: Mass spectrum corresponding to Figure S66.

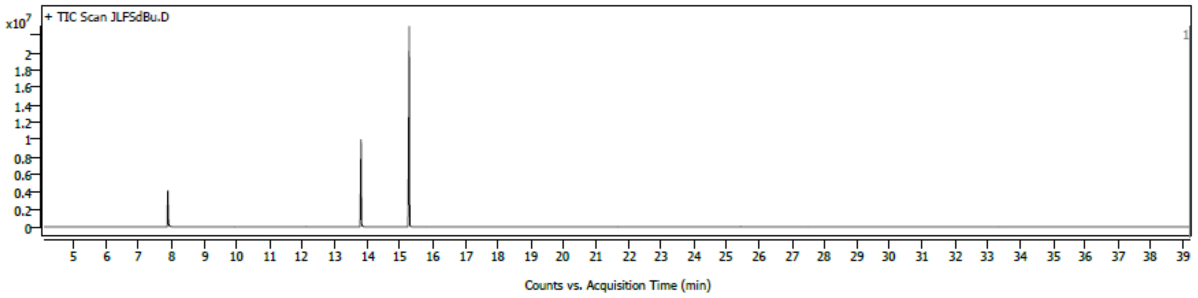

Figure S68: Gas chromatograph corresponding to Table S1, entry 6.

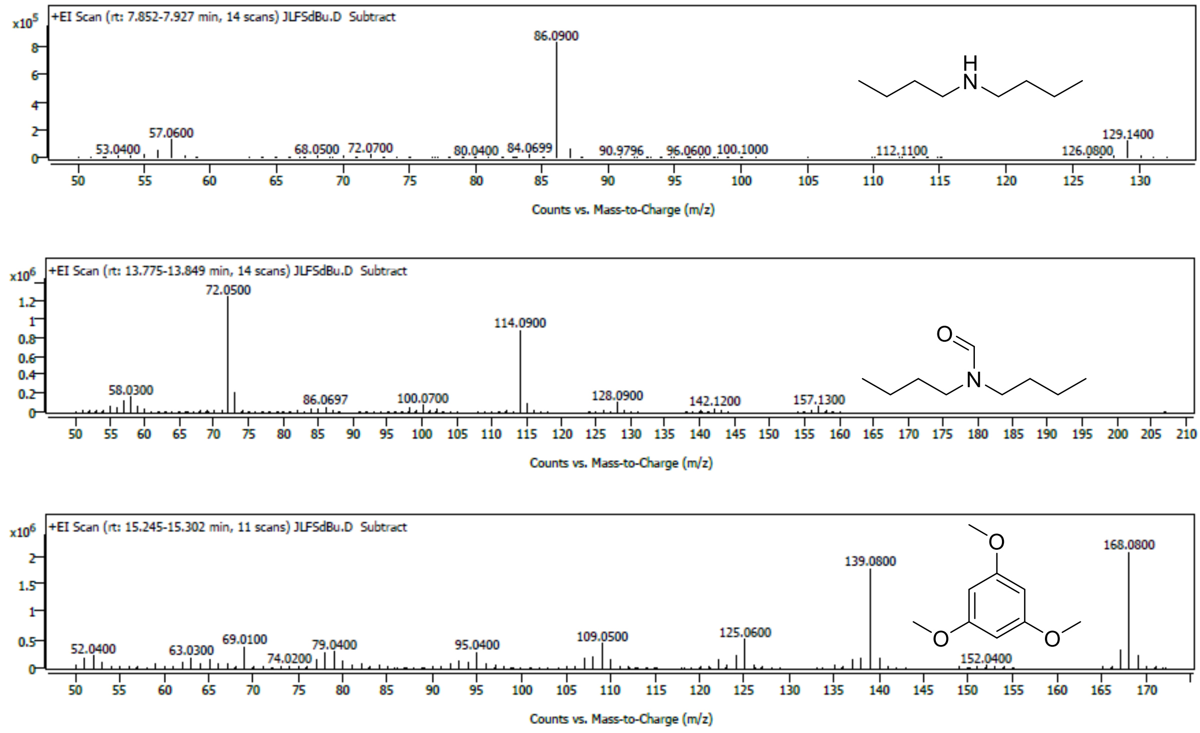

Figure S69: Mass spectrum corresponding to Figure S68.

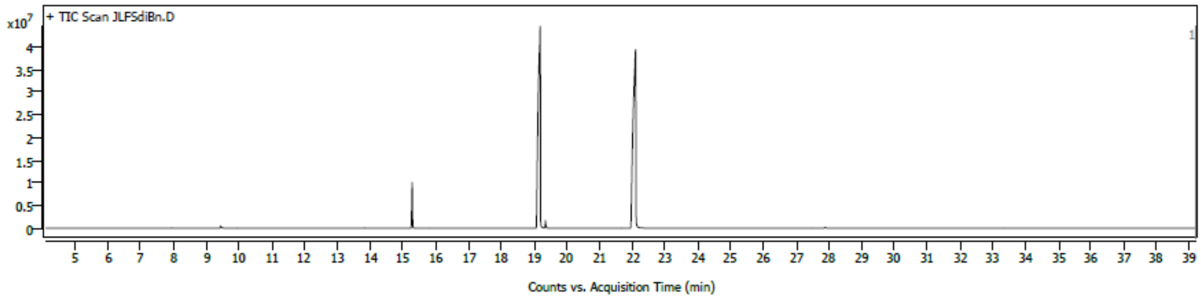

Figure S 70: Gas chromatograph corresponding to Table S1, entry 7.

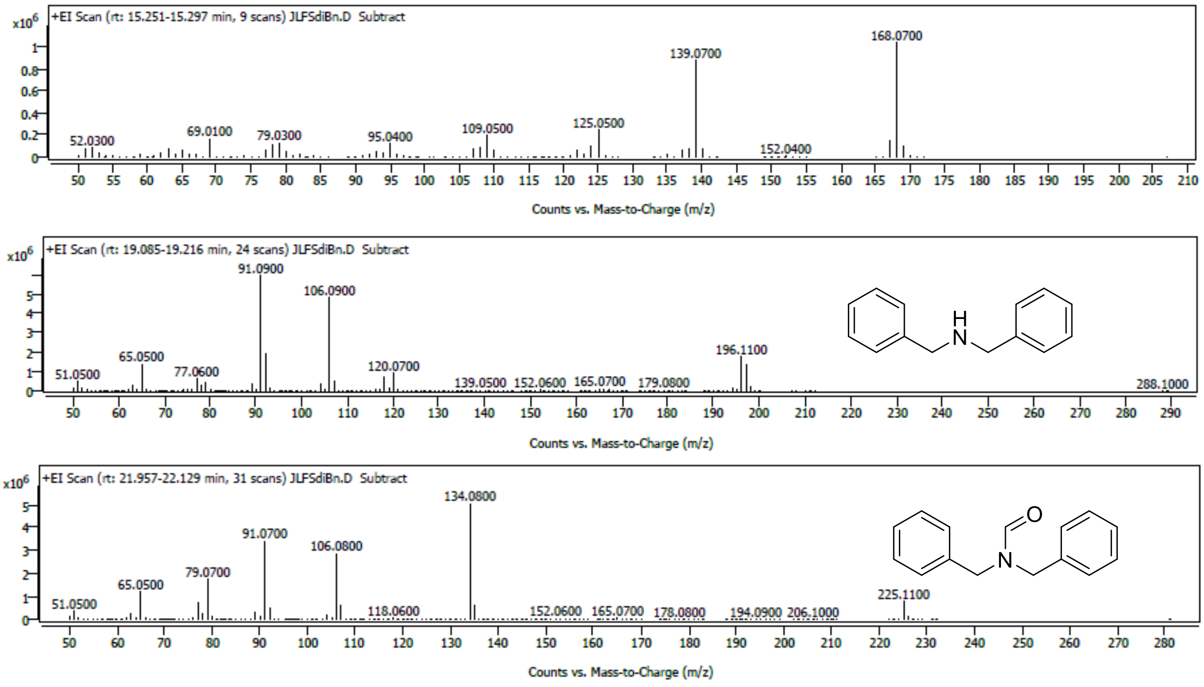

Figure S71: Mass spectrum corresponding to Figure S 70.

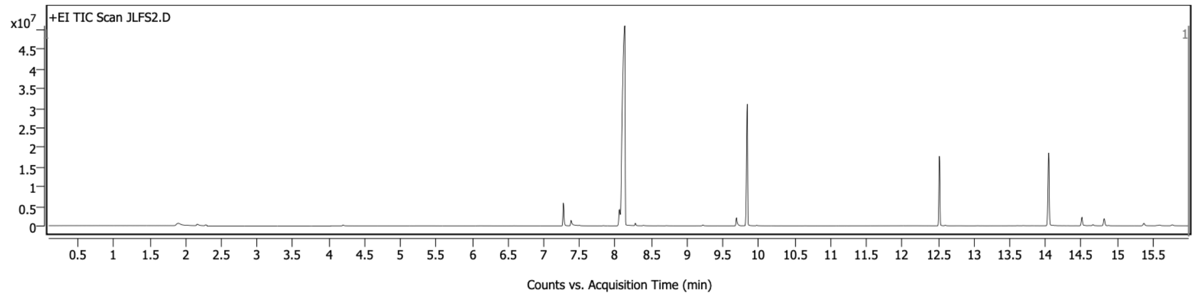

Figure S72: Gas chromatograph corresponding to Table S1, entry 8.

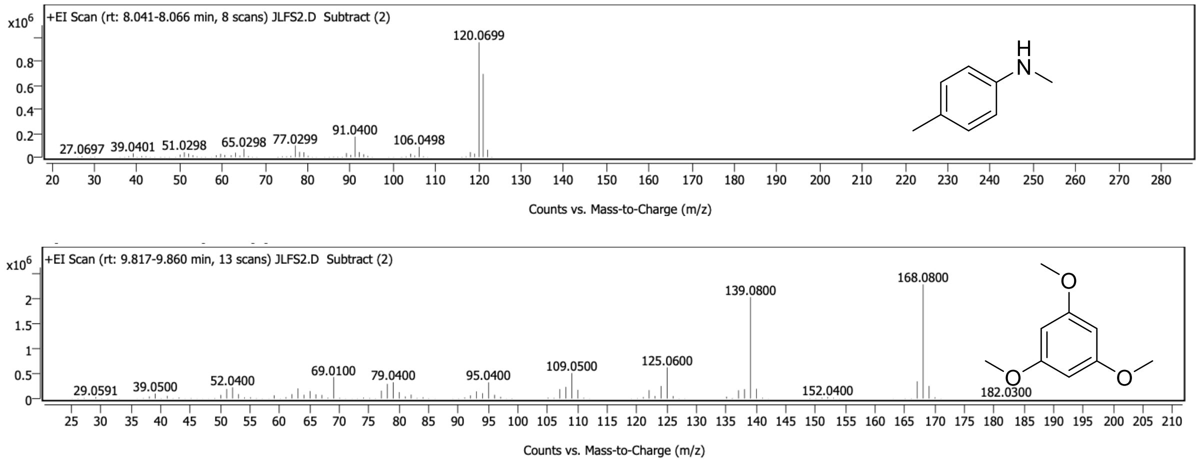

Figure S73: Mass spectrum corresponding to Figure S72.

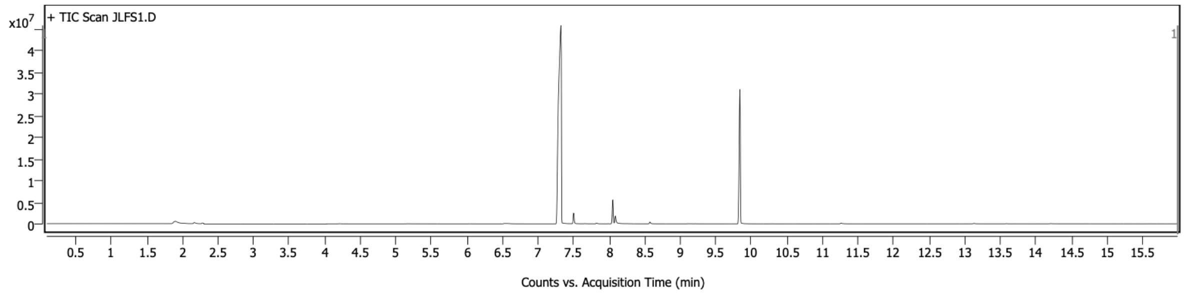

Figure S74: Gas chromatograph corresponding to Table S1, entry 9.

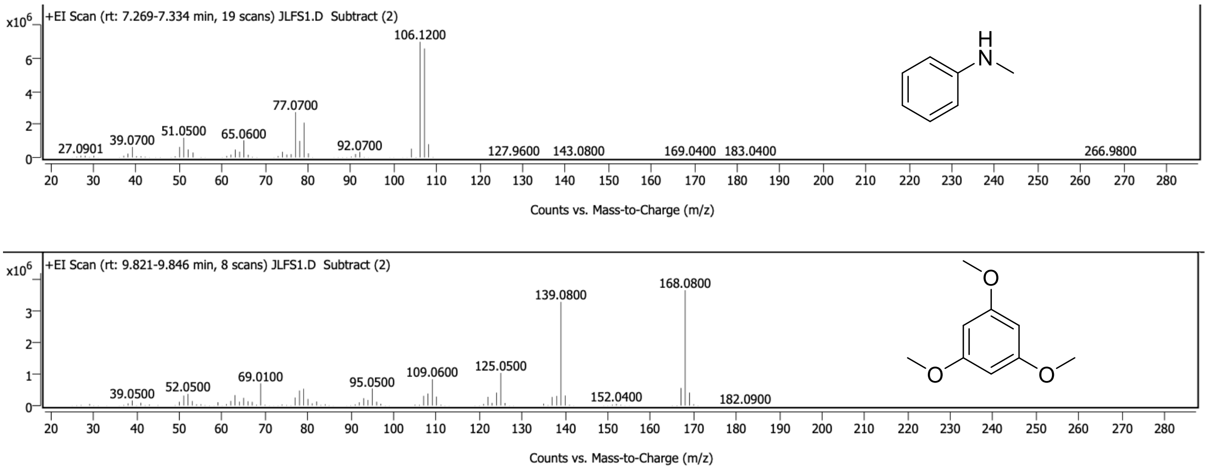

Figure S75: Mass spectrum corresponding to Figure S74.

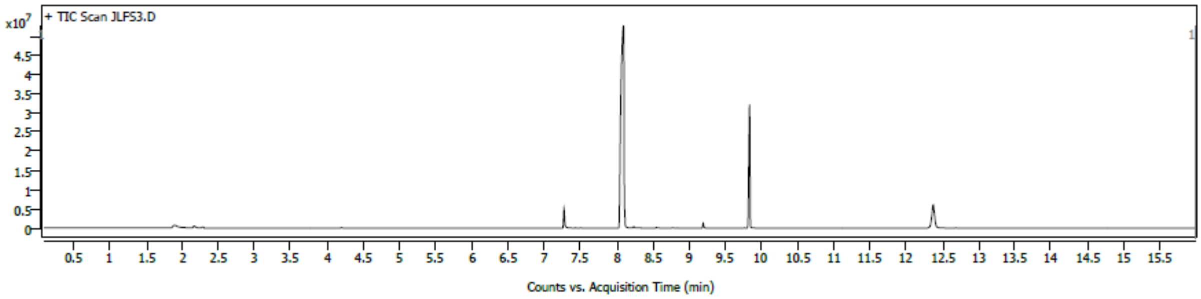

Figure S76: Gas chromatograph corresponding to Table S1, entry 10.

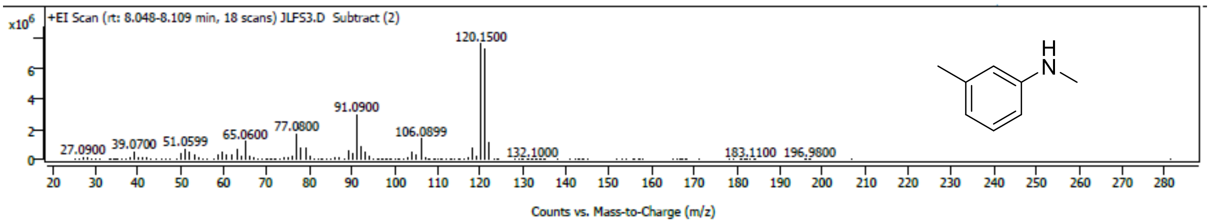

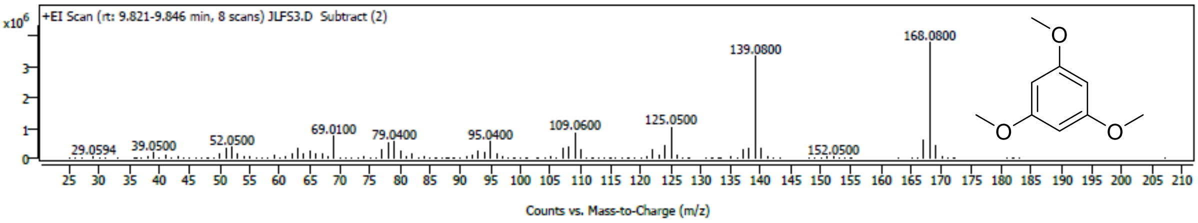

Figure S77: Mass spectrum corresponding to Figure S76.

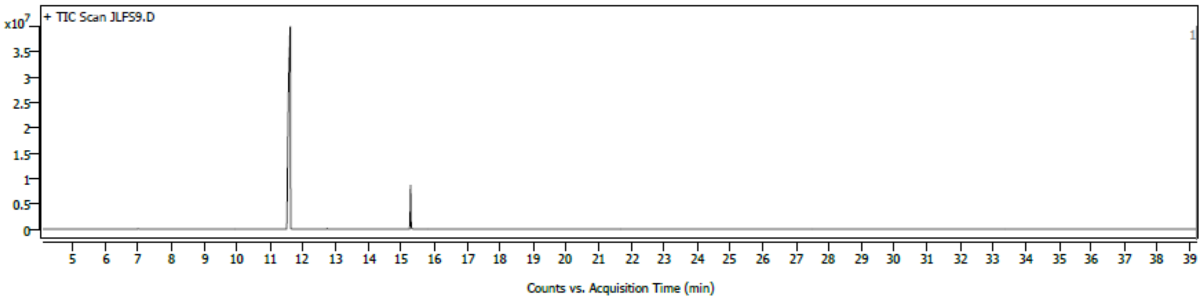

Figure S 78: Gas chromatograph corresponding to Table S1, entry 11.

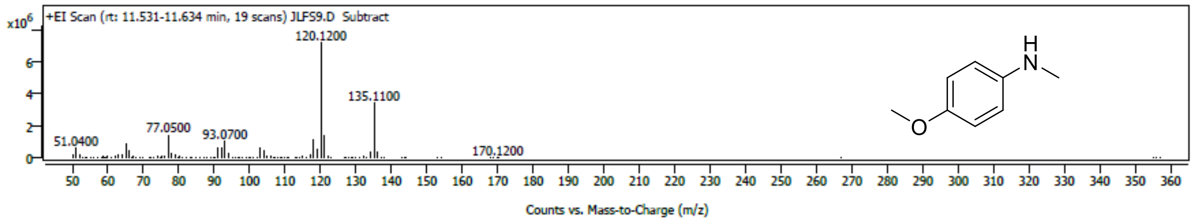

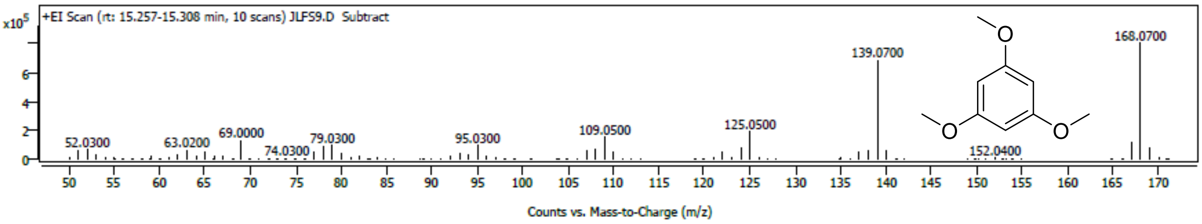

Figure S79: Mass spectrum corresponding to Figure S 78.

4.1.2. GC-MS data acquired from the decarbonylation of N-formyl morpholine

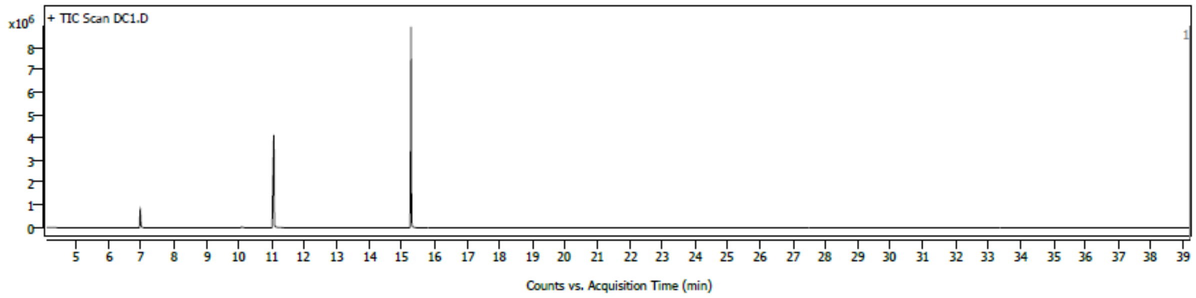

Figure S80: Gas chromatograph corresponding to Table S2, entry 1.

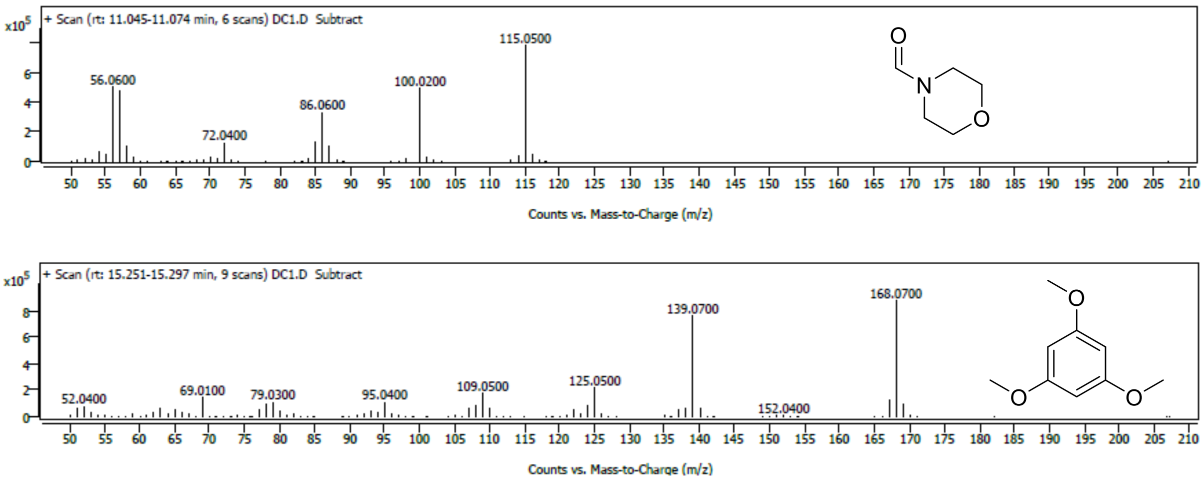

Figure S81: Mass spectra corresponding to Figure S80.

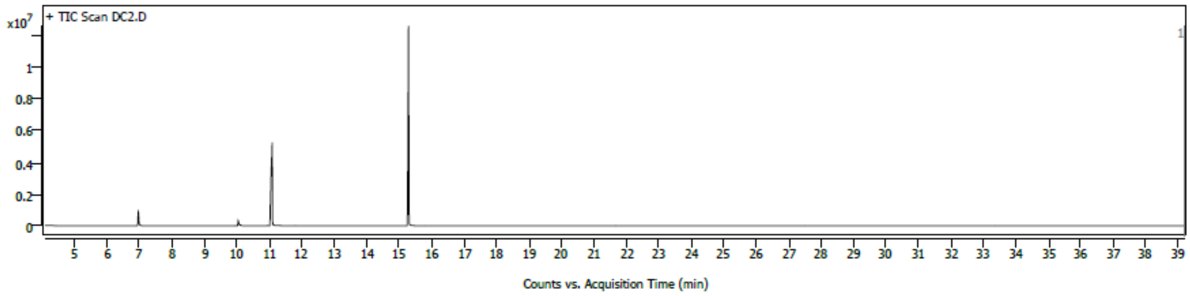

Figure S82: Gas chromatograph corresponding to Table S2, entry 2.

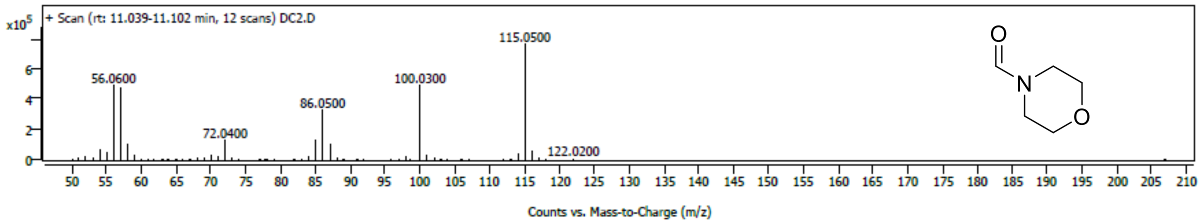

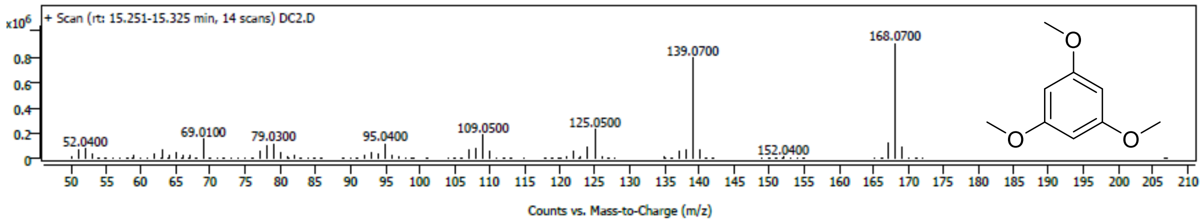

Figure S83: Mass spectra corresponding to Figure S82.

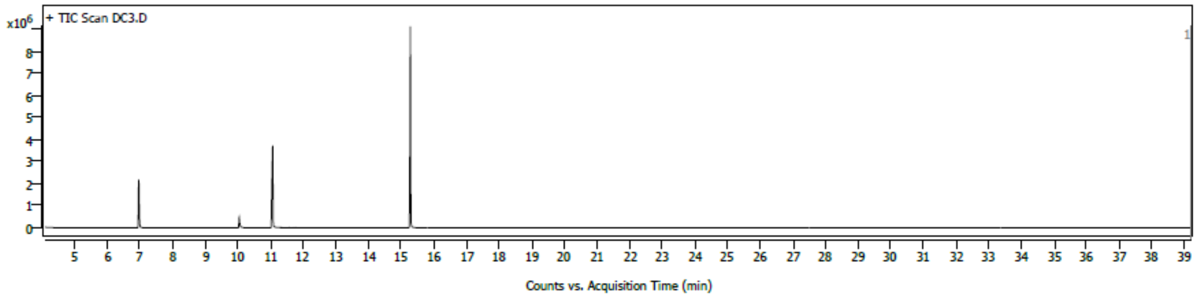

Figure S84: Gas chromatograph corresponding to Table S2, entry 3.

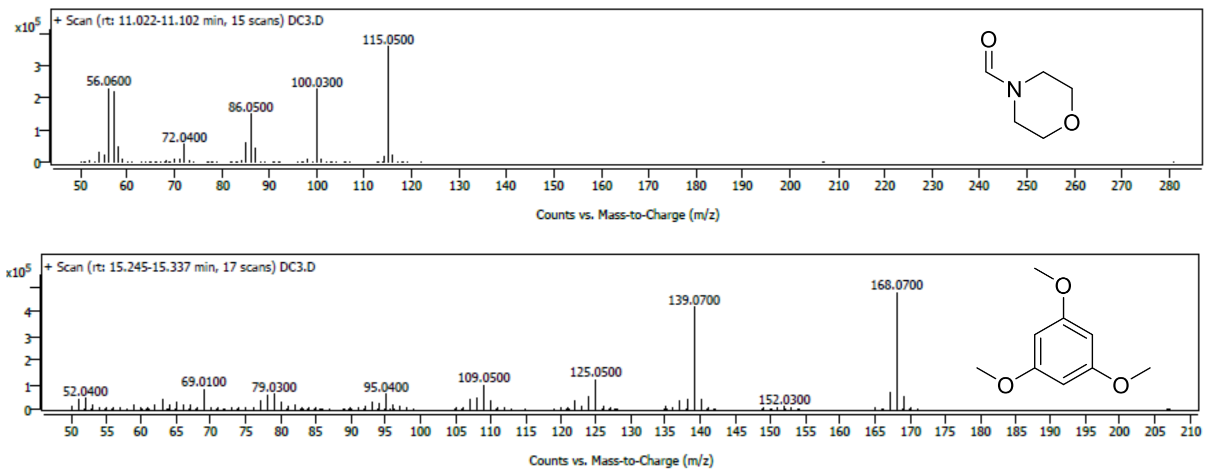

Figure S85: Mass spectra corresponding to Figure S84.

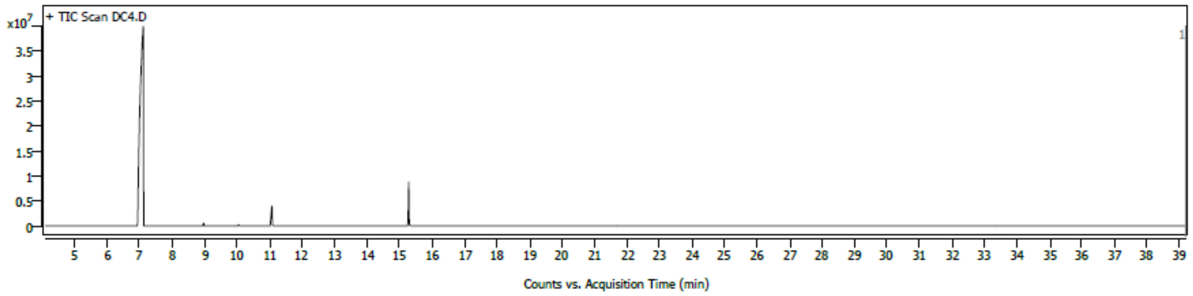

Figure S86: Gas chromatograph corresponding to Table S2, entry 4.

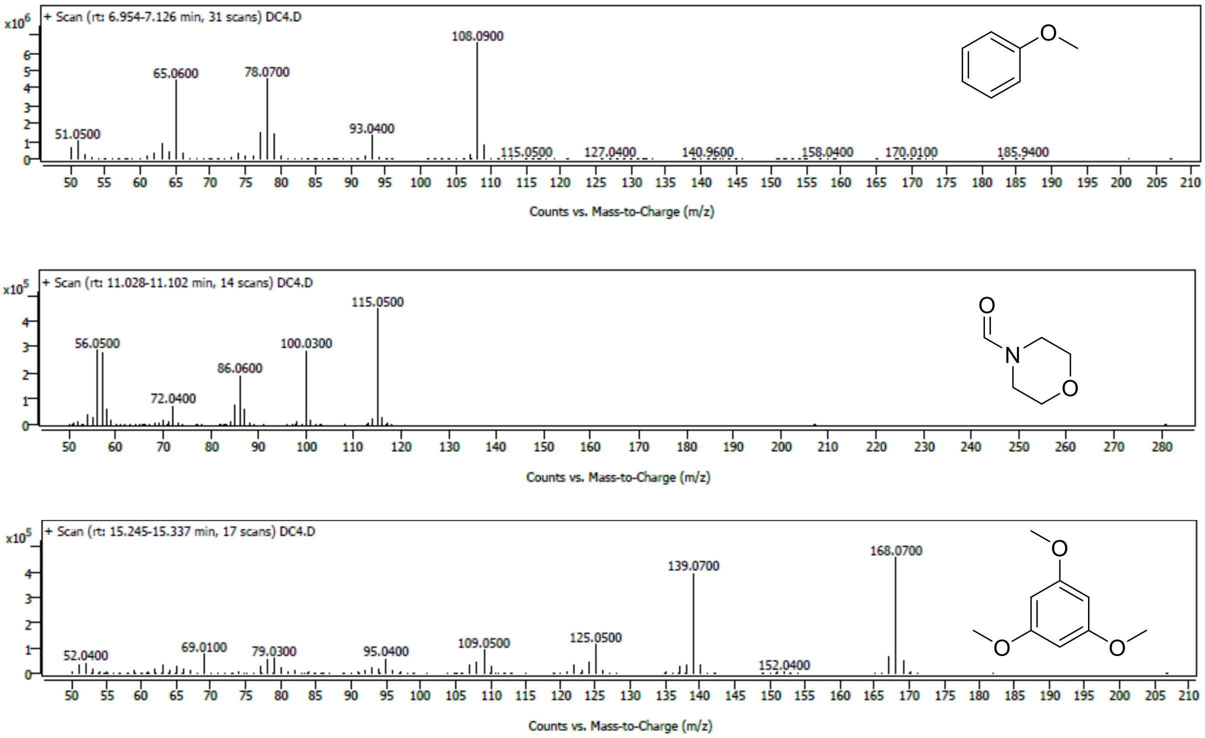

Figure S87: Mass spectra corresponding toFigure S86.

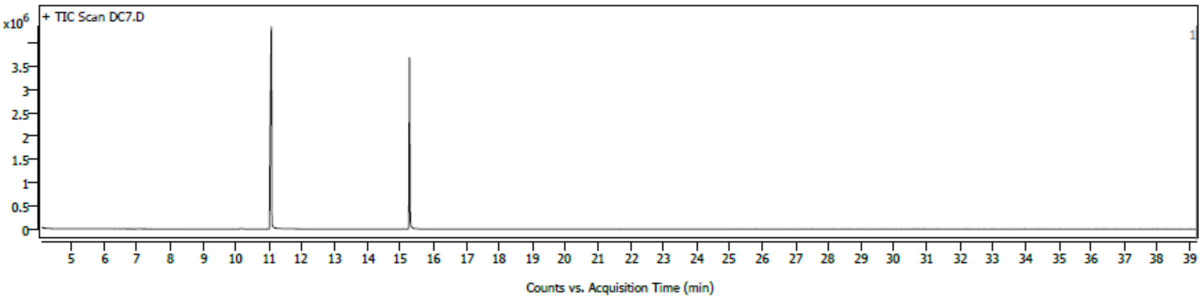

Figure S88: Gas chromatograph corresponding to Table S2, entry 5.

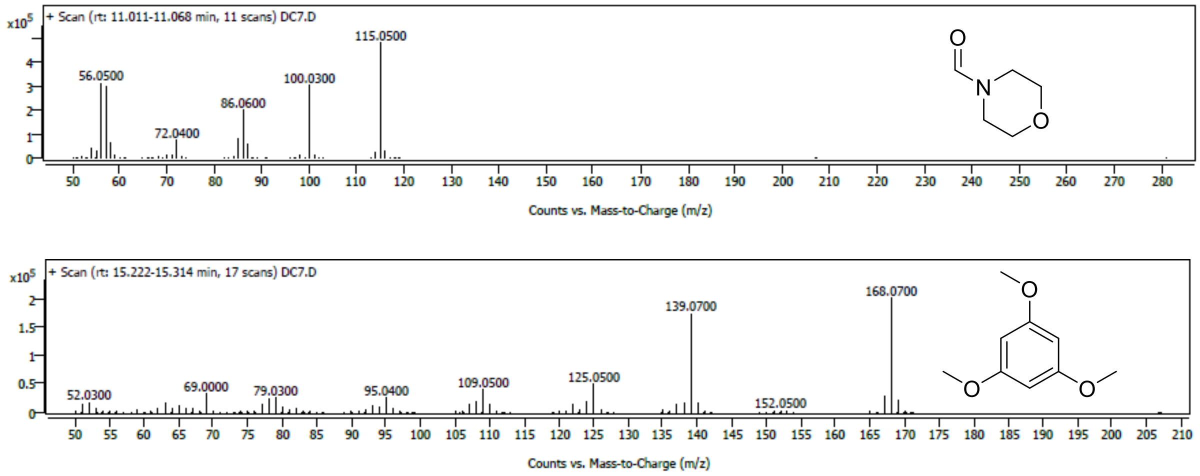

Figure S89: Mass spectra corresponding to Figure S88.

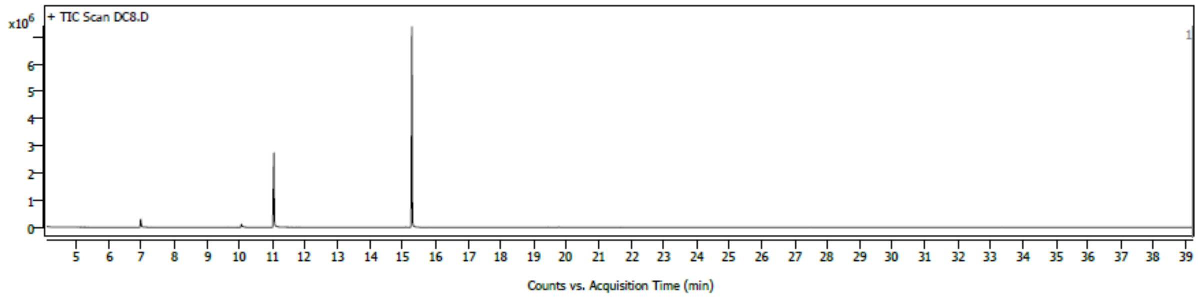

Figure S90: Gas chromatograph corresponding to Table S2, entry 6.

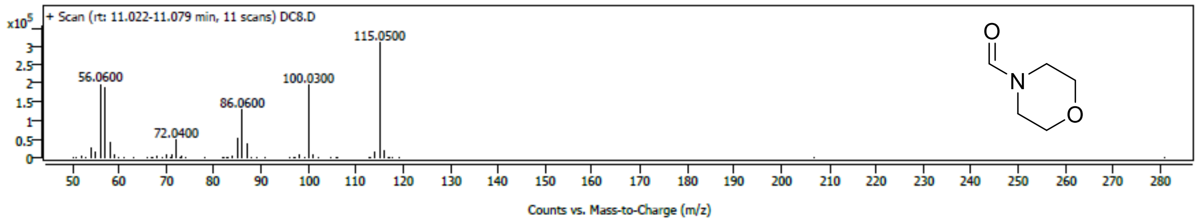

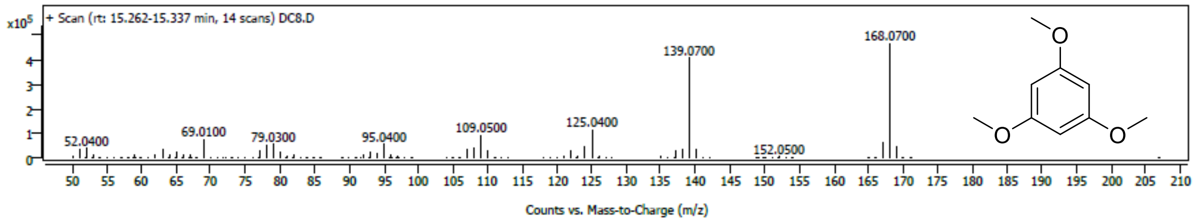

Figure S91: Mass spectra corresponding to Figure S90.

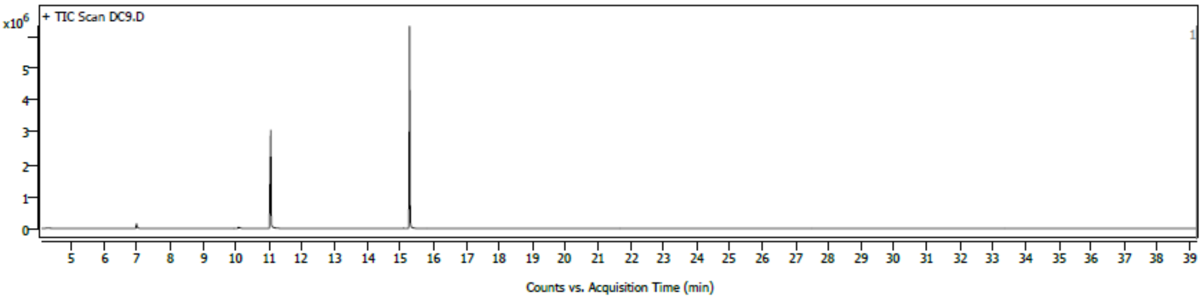

Figure S92: Gas chromatograph corresponding to Table S2, entry 7.

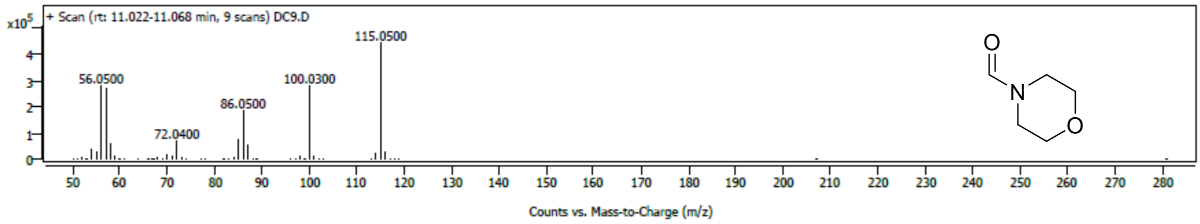

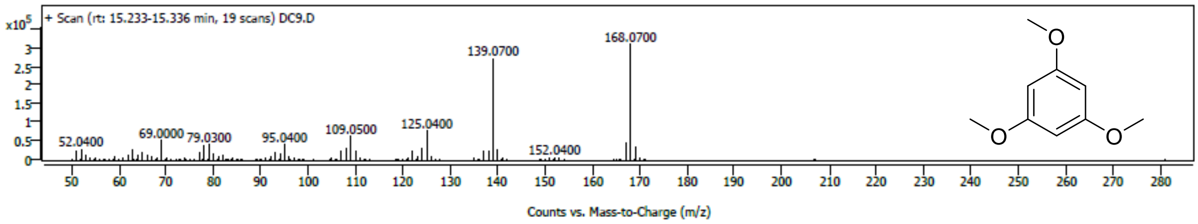

Figure S93: Mass spectra corresponding to Figure S92.

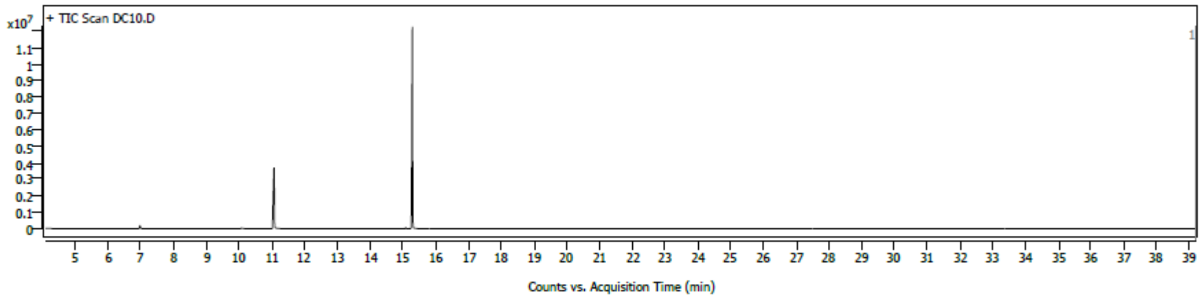

Figure S94: Gas chromatograph corresponding to Table S2, entry 8.

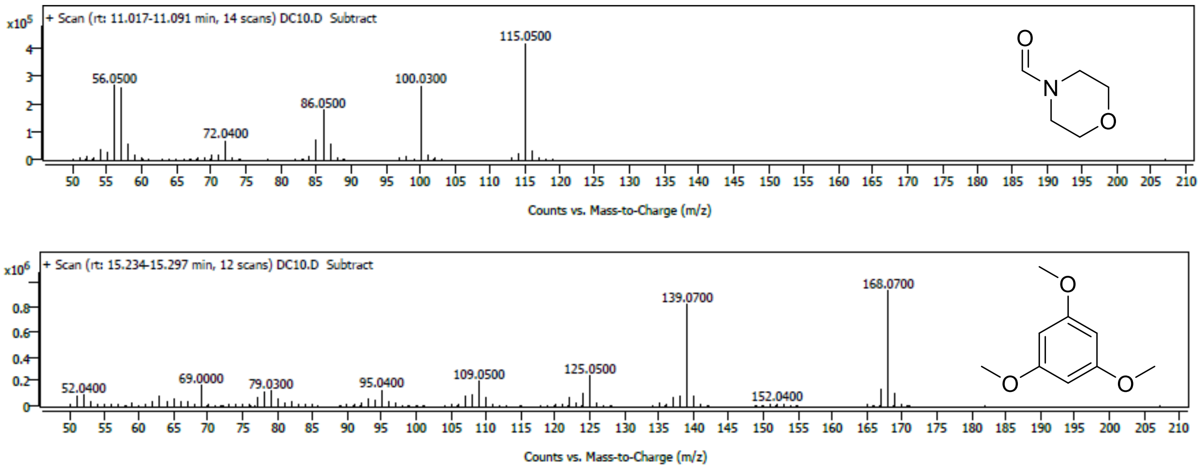

Figure S95: Mass spectra corresponding to Figure S94.

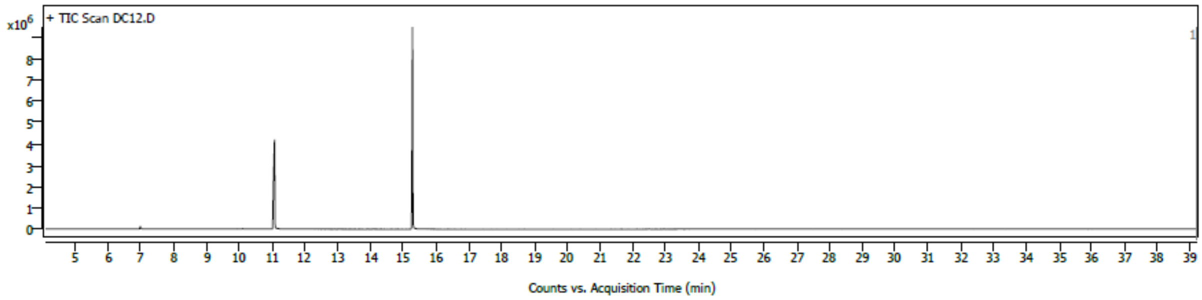

Figure S96: Gas chromatograph corresponding to Table S2, entry 9.

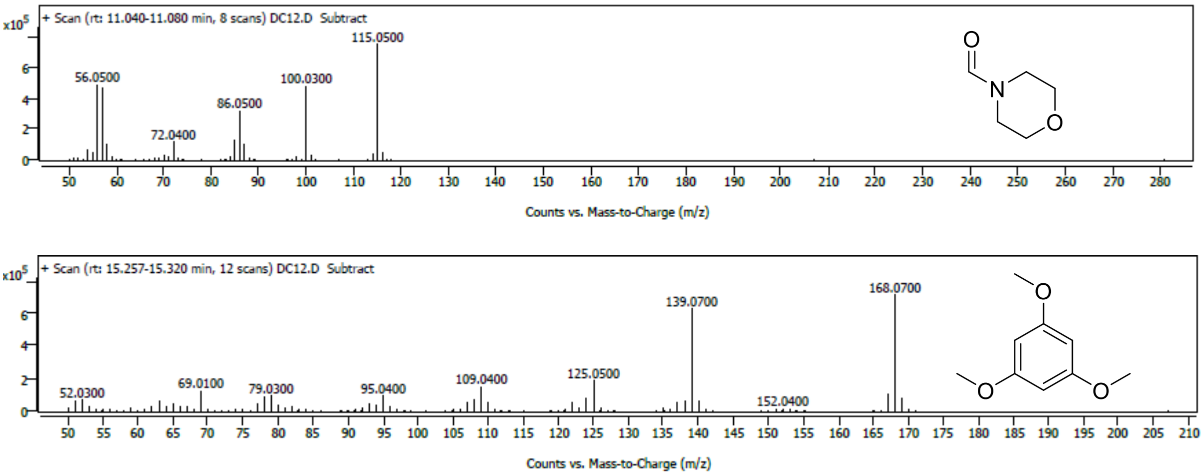

Figure S97: Mass spectra corresponding to Figure S96.

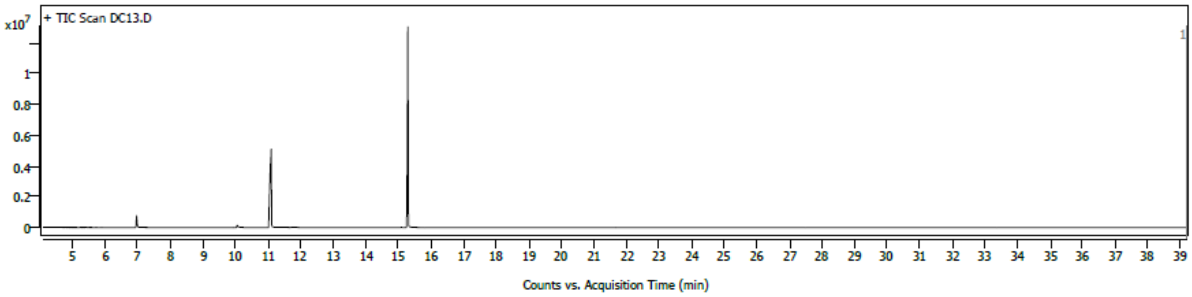

Figure S98: Gas chromatograph corresponding to Table S2, entry 10.

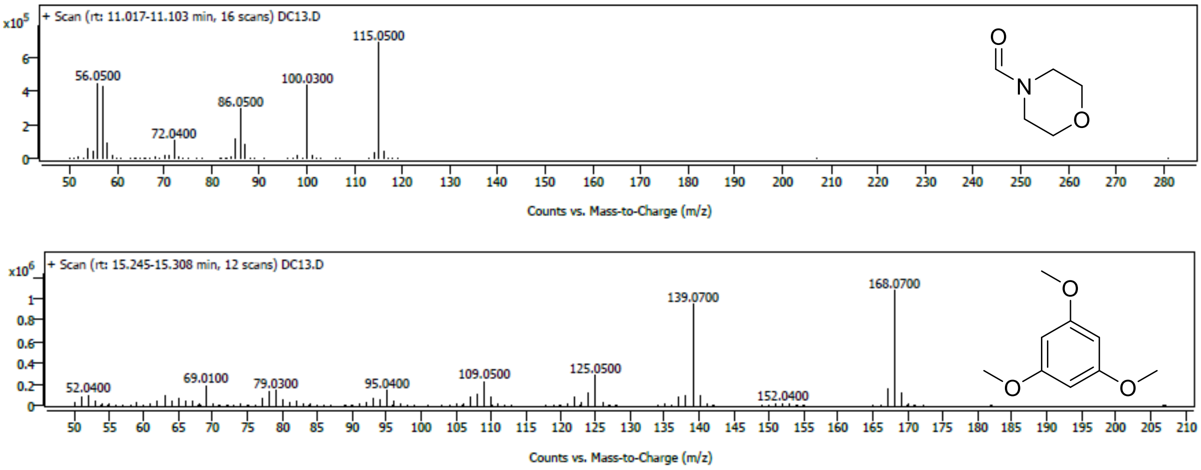

Figure S99: Mass spectra corresponding to Figure S98.

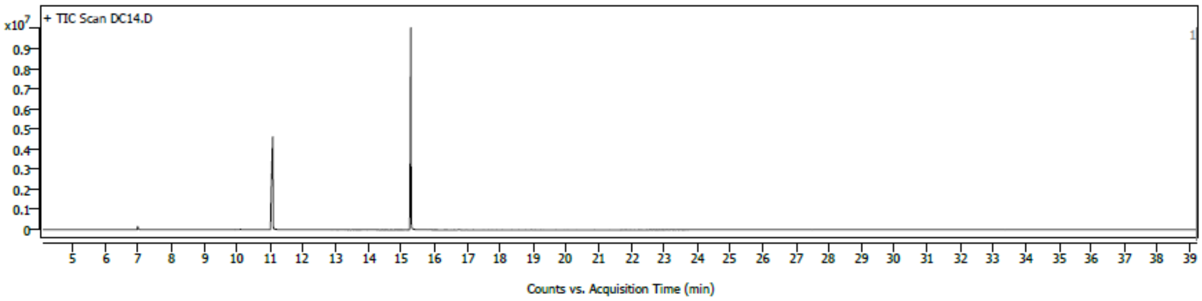

Figure S100: Gas chromatograph corresponding to Table S2, entry 11.

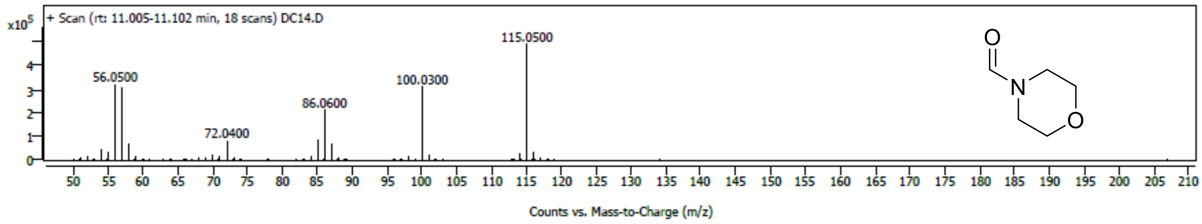

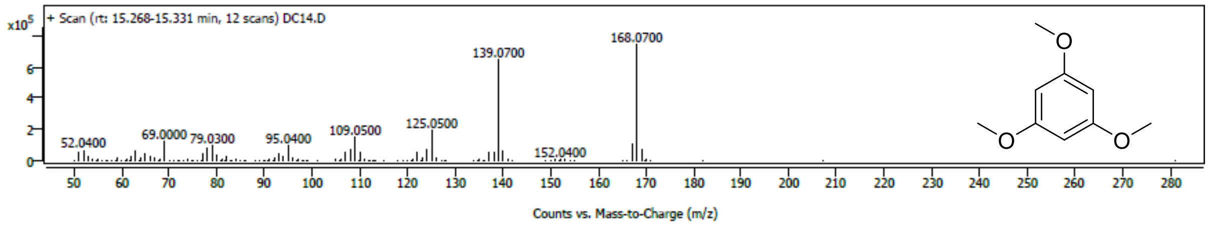

Figure S101: Mass spectra corresponding to Figure S100.

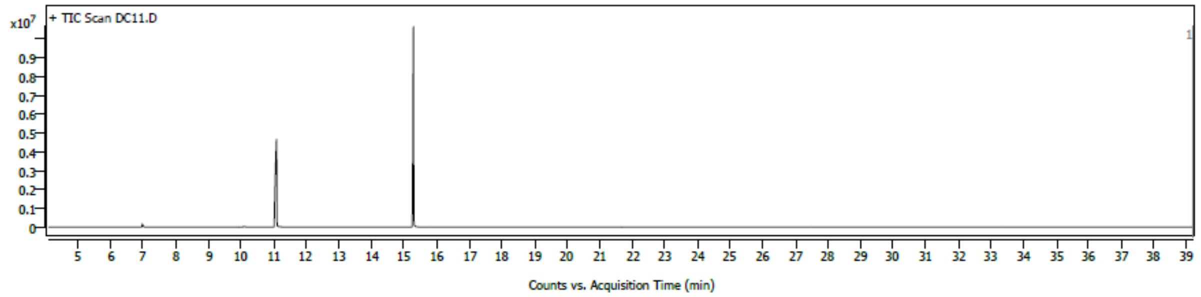

Figure S102: Gas chromatograph corresponding to Table S2, entry 12.

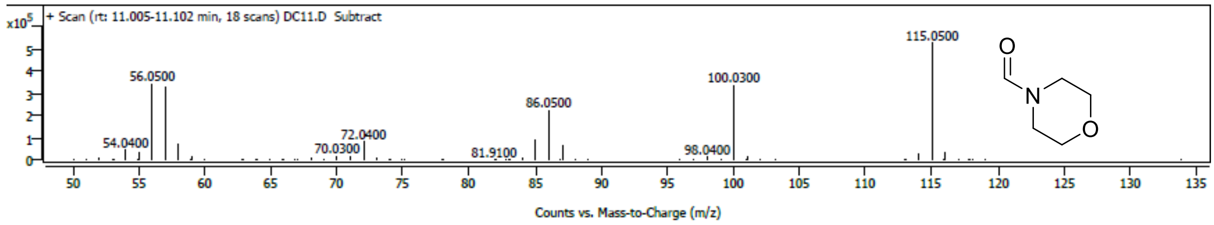

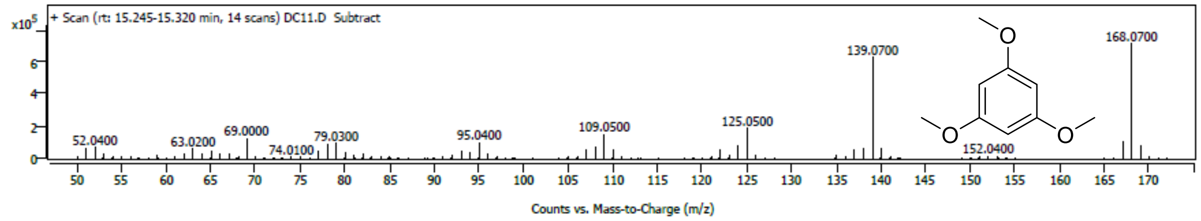

Figure S103: Mass spectra corresponding to Figure S102.

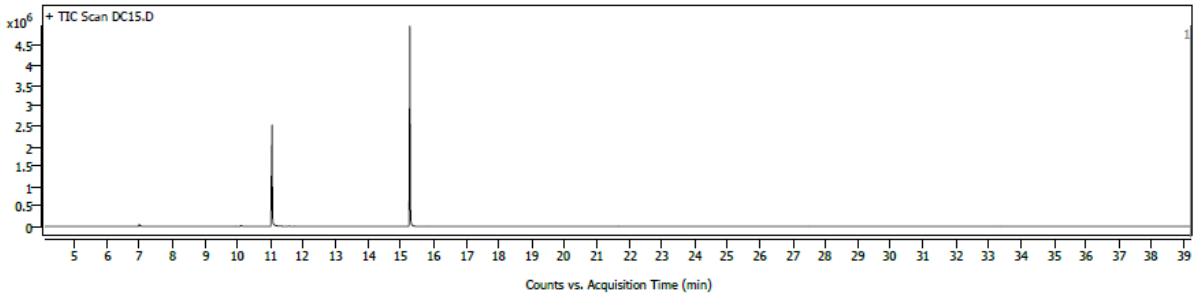

Figure S104: Gas chromatograph corresponding to Table S2, entry 13.

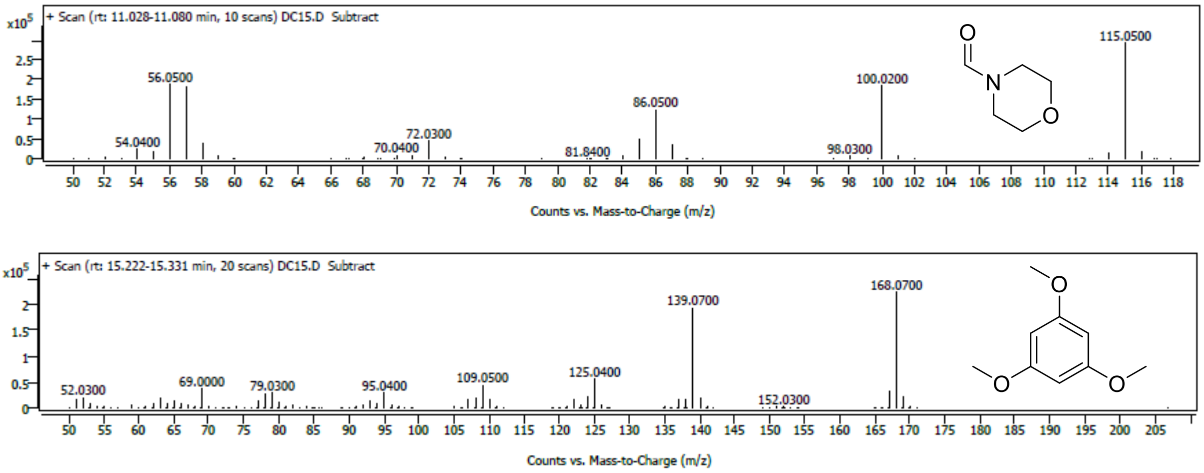

Figure S105: Mass spectra corresponding to Figure S104.

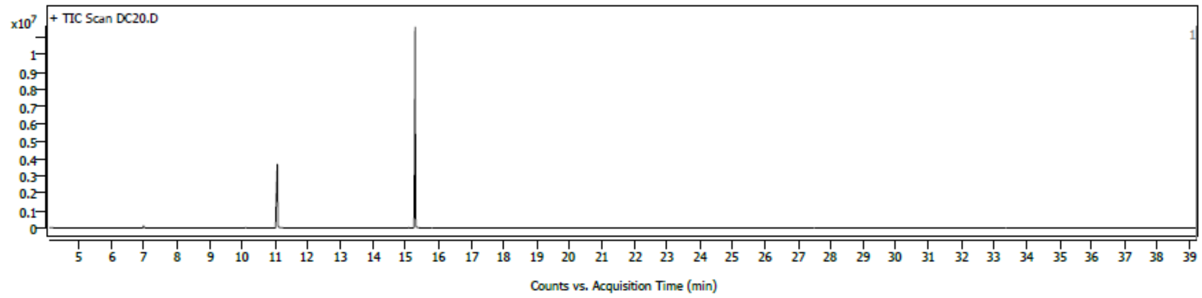

Figure S106: Gas chromatograph corresponding to Table S2, entry 14.

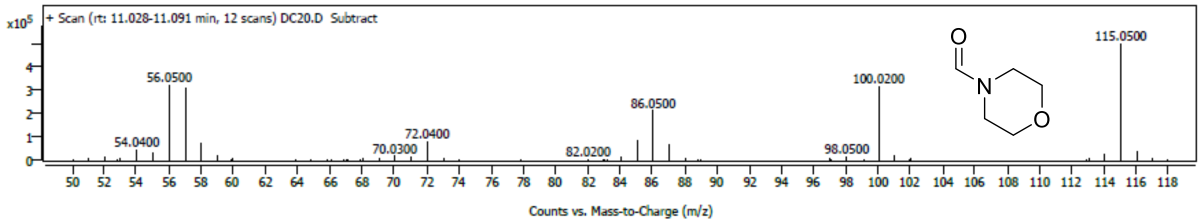

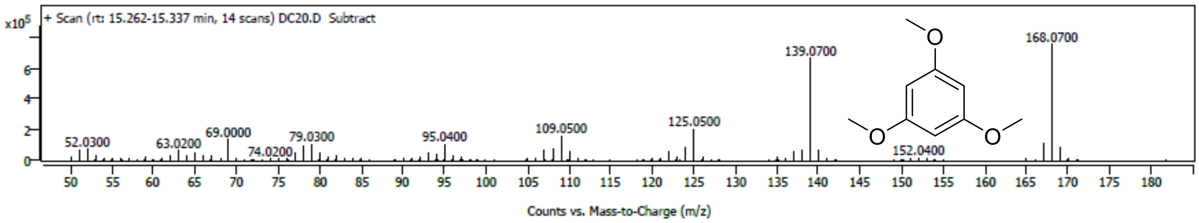

Figure S107: Mass spectra corresponding to Figure S106.

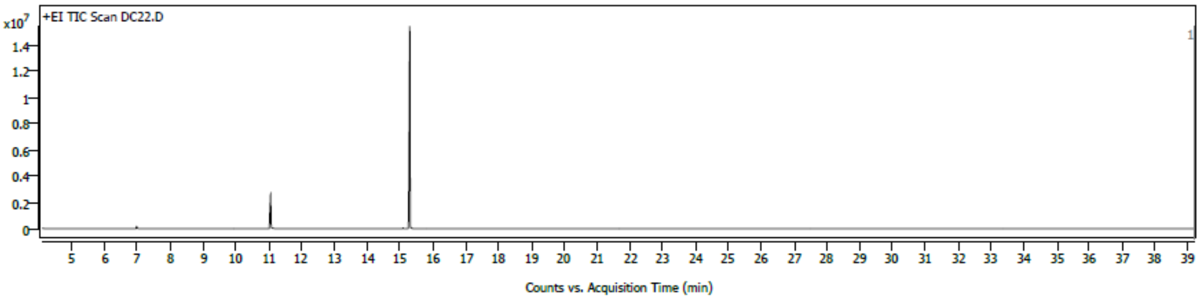

Figure S108: Gas chromatograph corresponding to Table S2, entry 15.

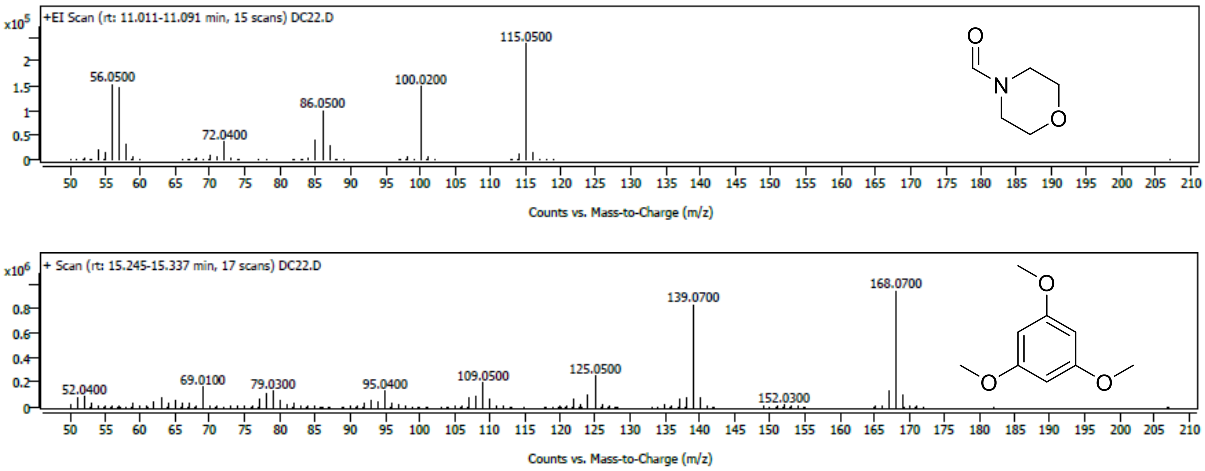

Figure S109: Mass spectra corresponding to Figure S108.

4.1.3. GC-MS data corresponding to decarbonylation corresponding to formamides

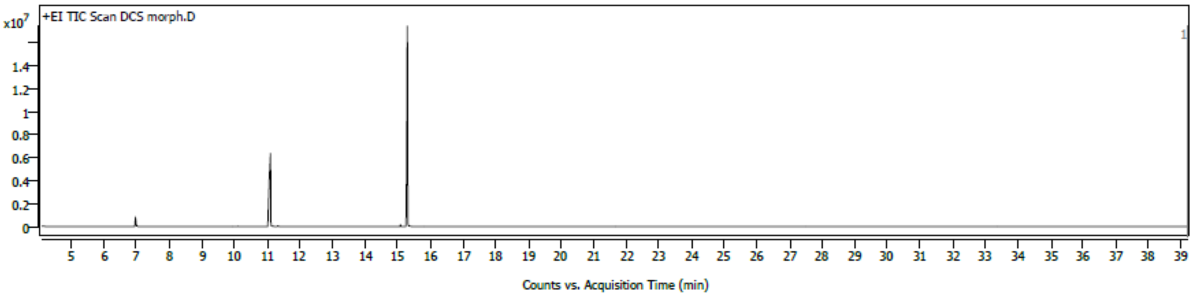

Figure S110: Gas chromatograph corresponding to Table S3, entry 1.

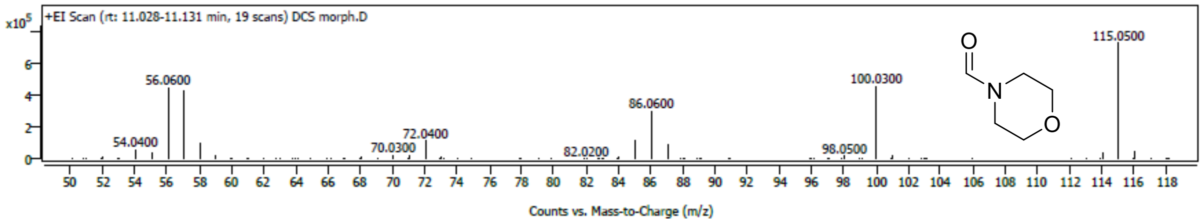

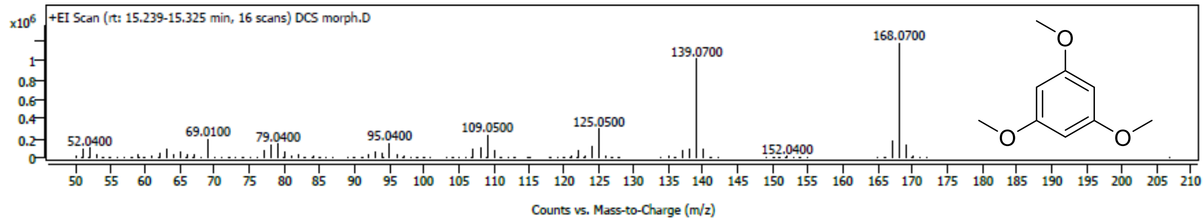

Figure S111: Mass spectra corresponding to Figure S110.

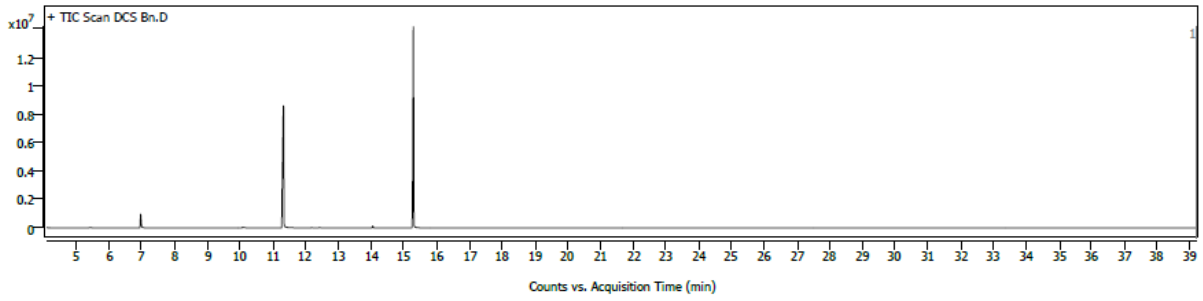

Figure S112: Gas chromatograph corresponding to Table S3, entry 2.

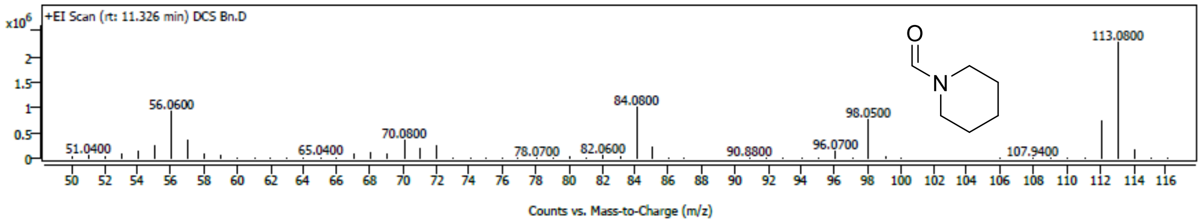

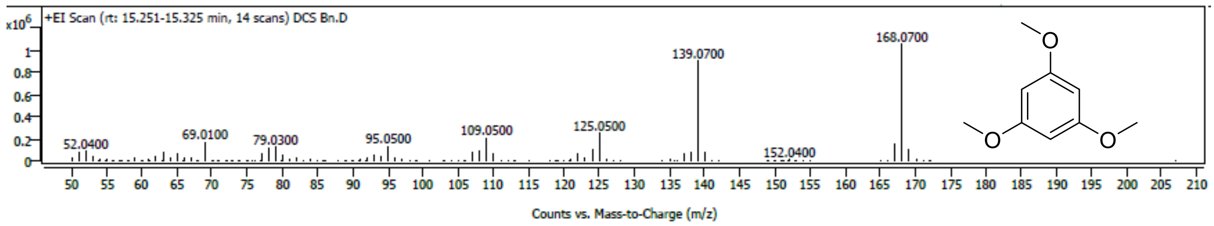

Figure S113: Mass spectra corresponding to Figure S112.

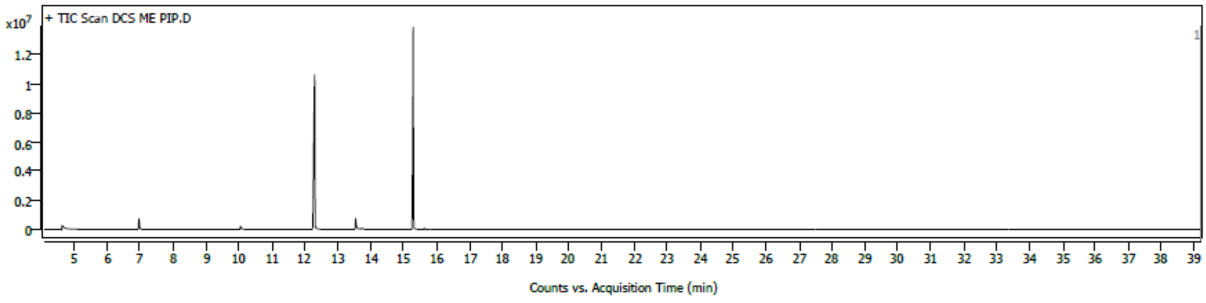

Figure S114: Gas chromatograph corresponding to Table S3, entry 3.

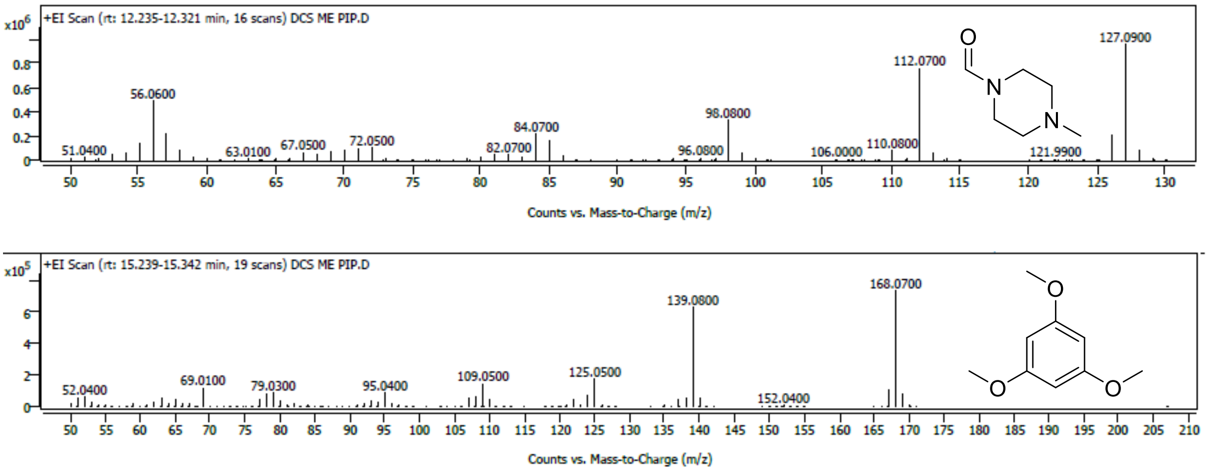

Figure S115: Mass spectra corresponding to Figure S114.

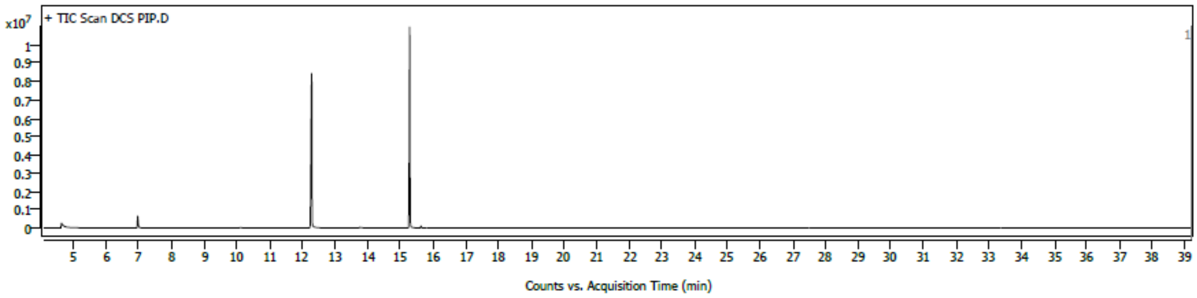

Figure S116: Gas chromatograph corresponding to Table S3, entry 4.

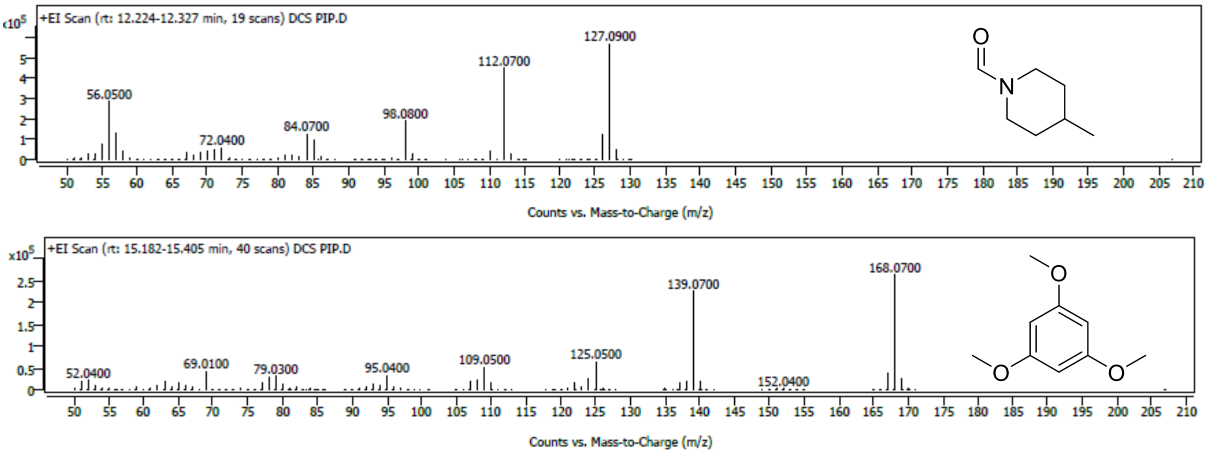

Figure S117: Mass spectra corresponding to Figure S116.

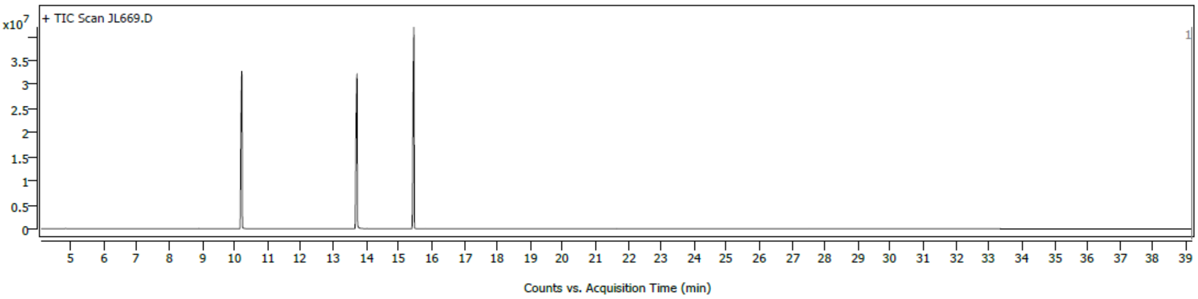

Figure S118: : Gas chromatograph corresponding to Table S3, entry 5.

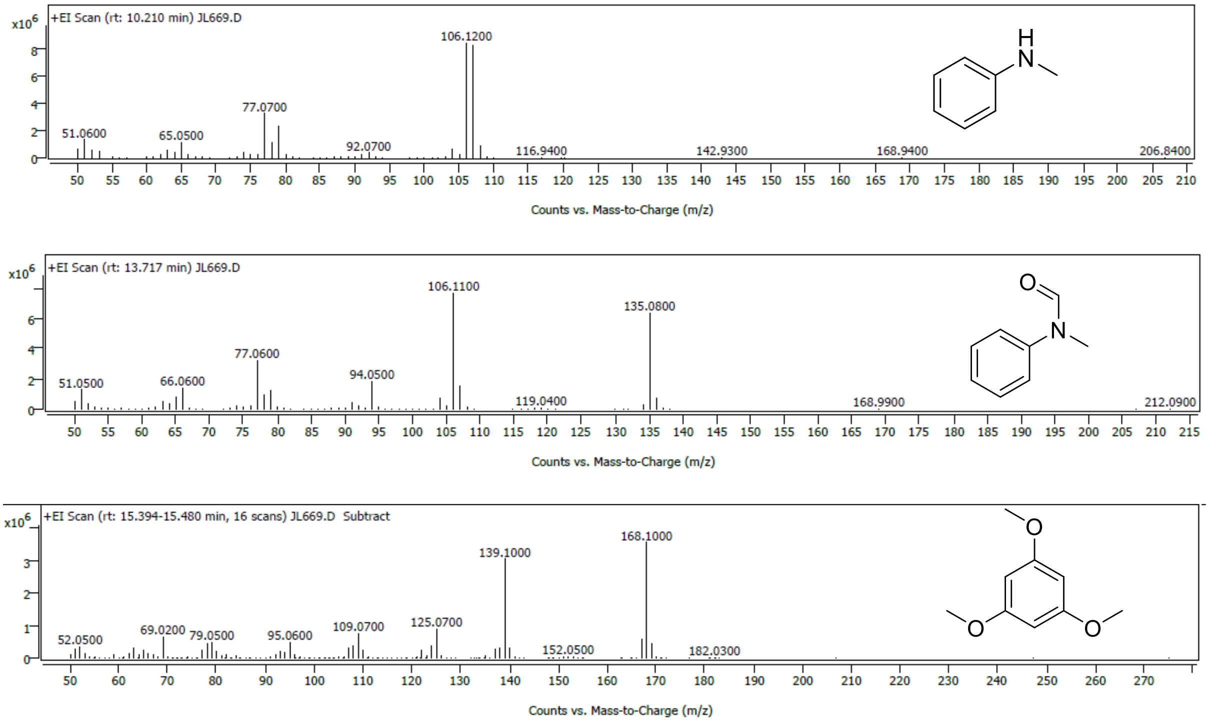

Figure S119: Mass spectra corresponding to Figure S118.

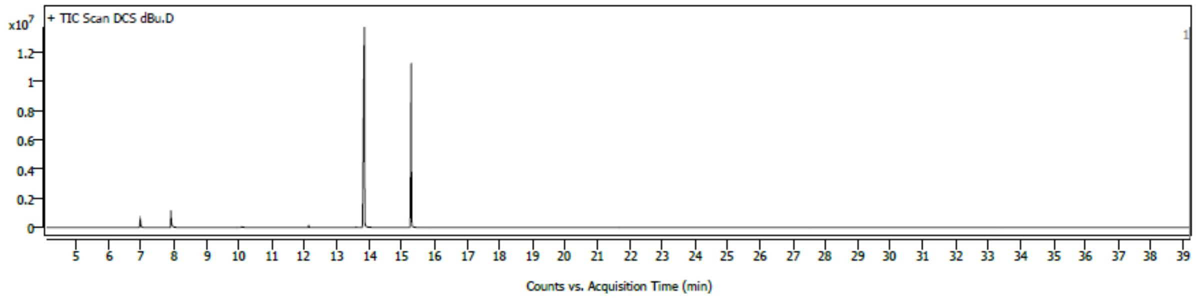

Figure S120: Gas chromatograph corresponding to Table S3, entry 5.

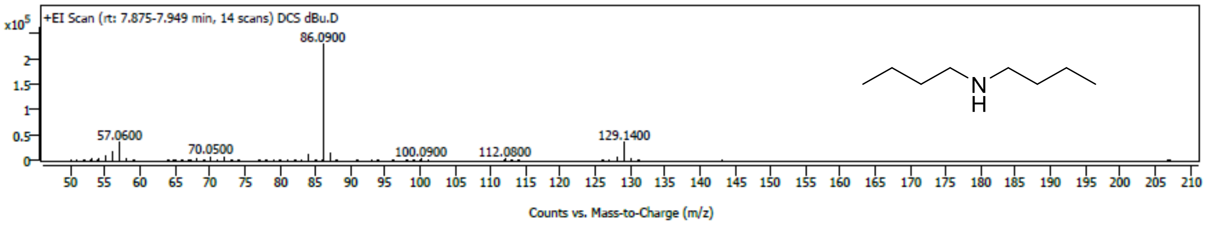

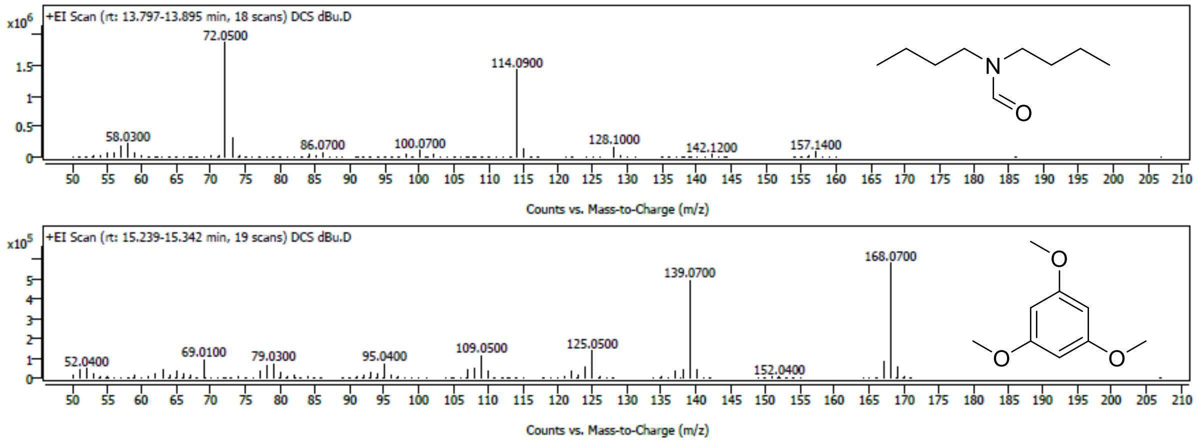

Figure S121: Mass spectra corresponding to Figure S120.

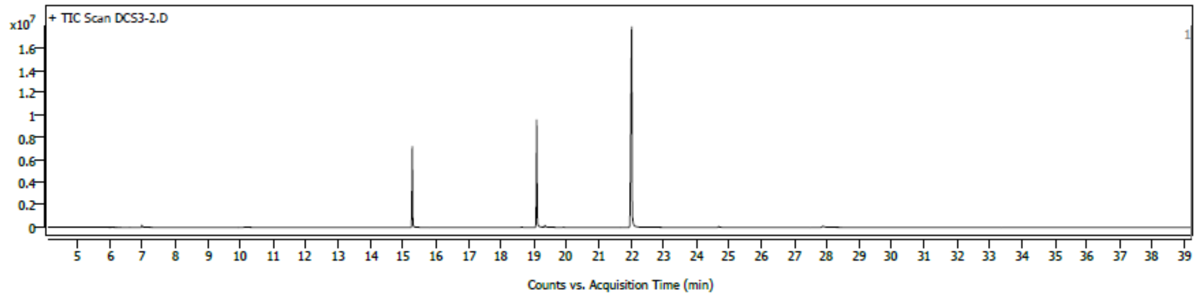

Figure S122: Gas chromatograph corresponding to Table S3, entry 6.

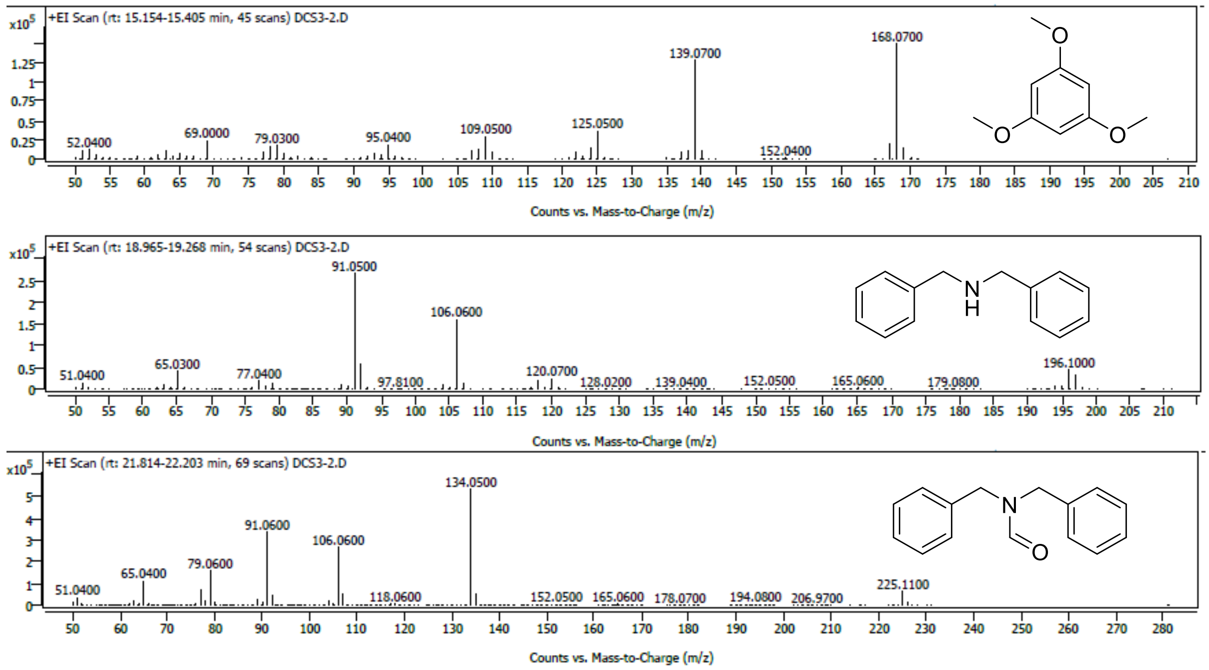

Figure S123: Mass spectra corresponding to Figure S122.

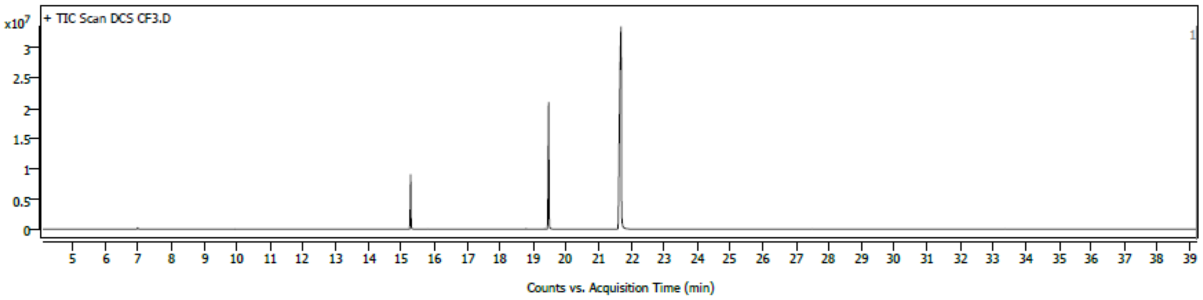

Figure S124: Gas chromatograph corresponding to Table S3, entry 7.

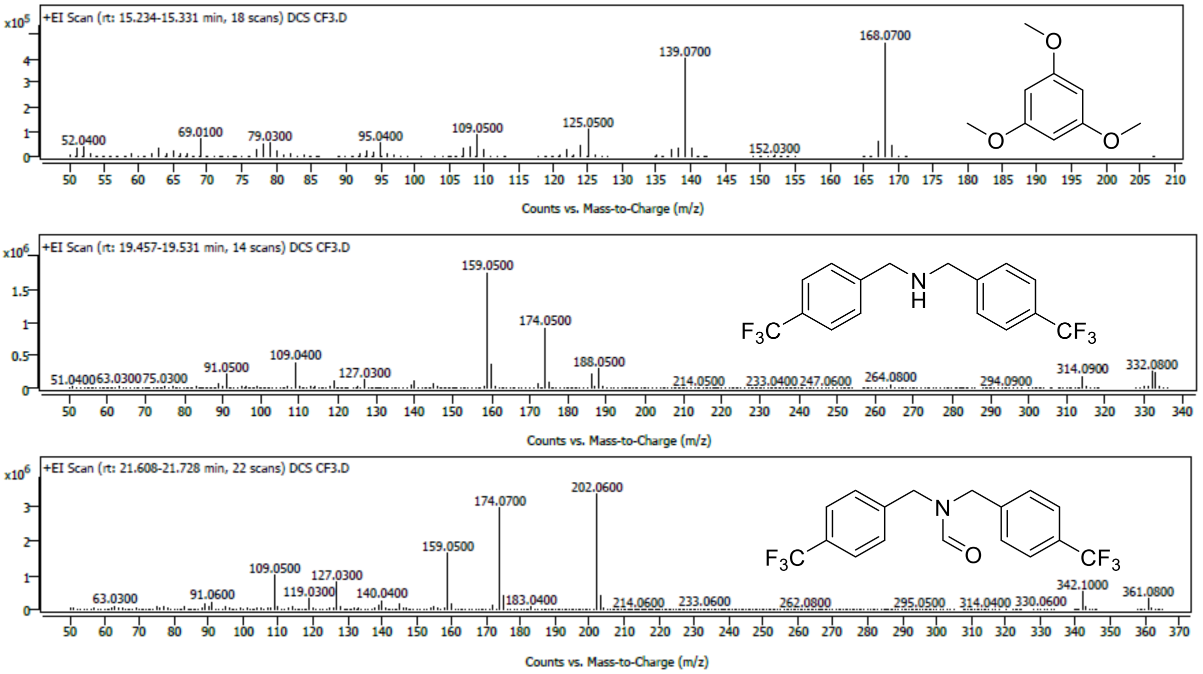

Figure S125: Mass spectra corresponding to Figure S124.

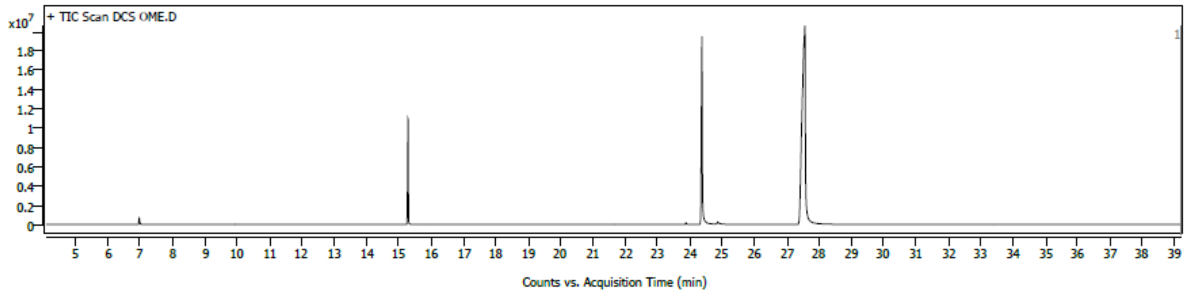

Figure S126: Gas chromatograph corresponding to Table S3, entry 8.

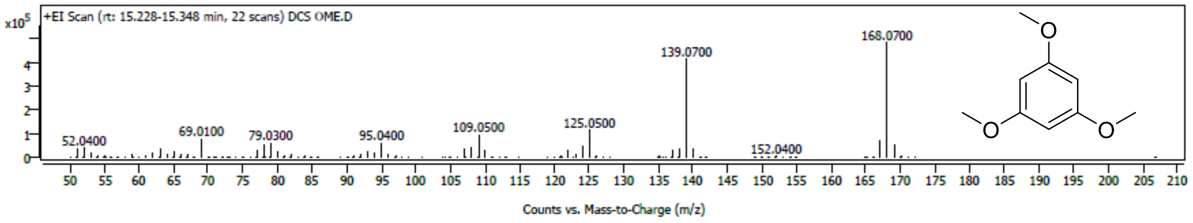

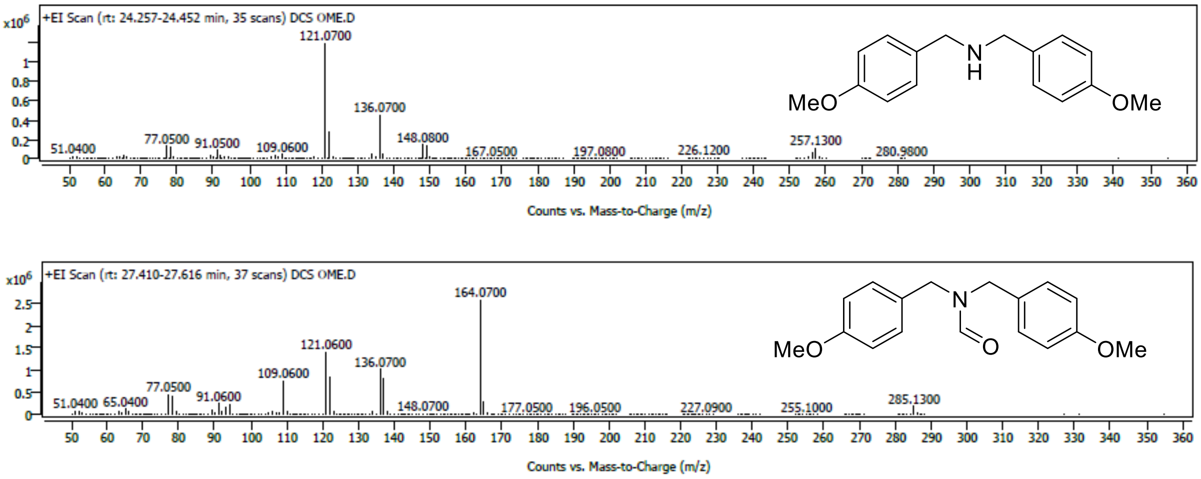

Figure S127: Mass spectra corresponding to Figure S126.

4.1.4. GC-MS data acquired from the decarbonylation of N-formyl morpholine at different morpholine concentrations

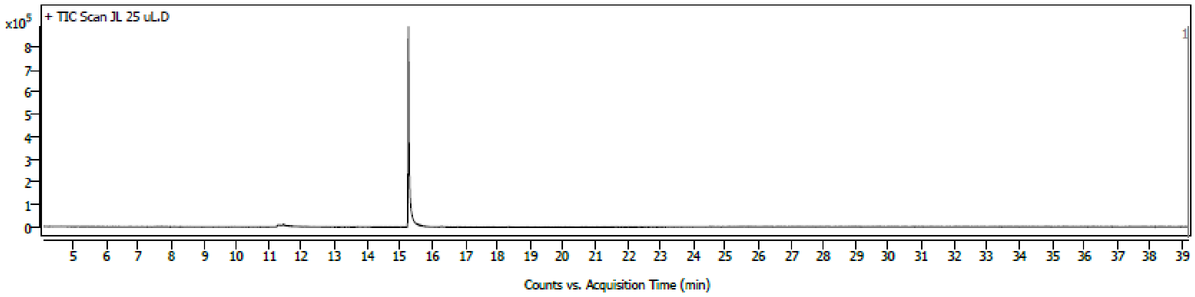

Figure S128: Gas chromatograph corresponding to decarbonylation of 0.125 M N-formylmorpholine.

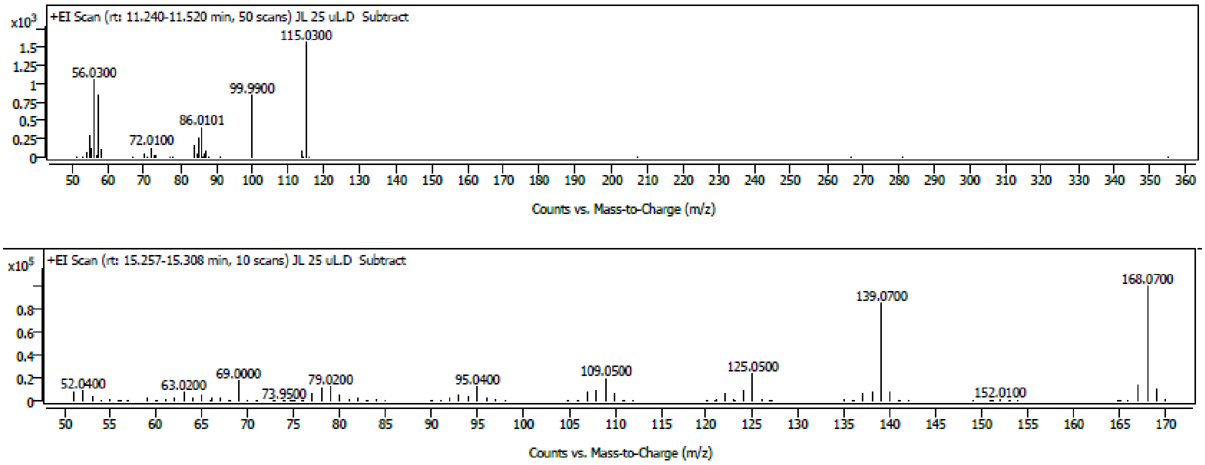

Figure S129: Mass spectra corresponding to Figure S128.

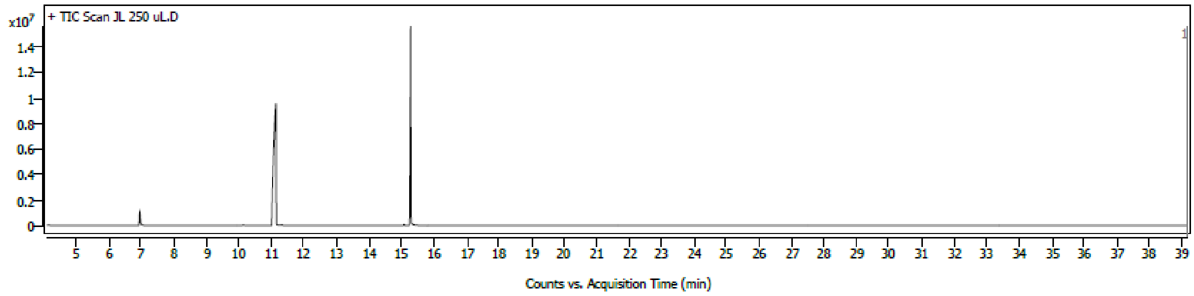

Figure S130: Gas chromatograph corresponding to decarbonylation of 1.25 M N-formylmorpholine.

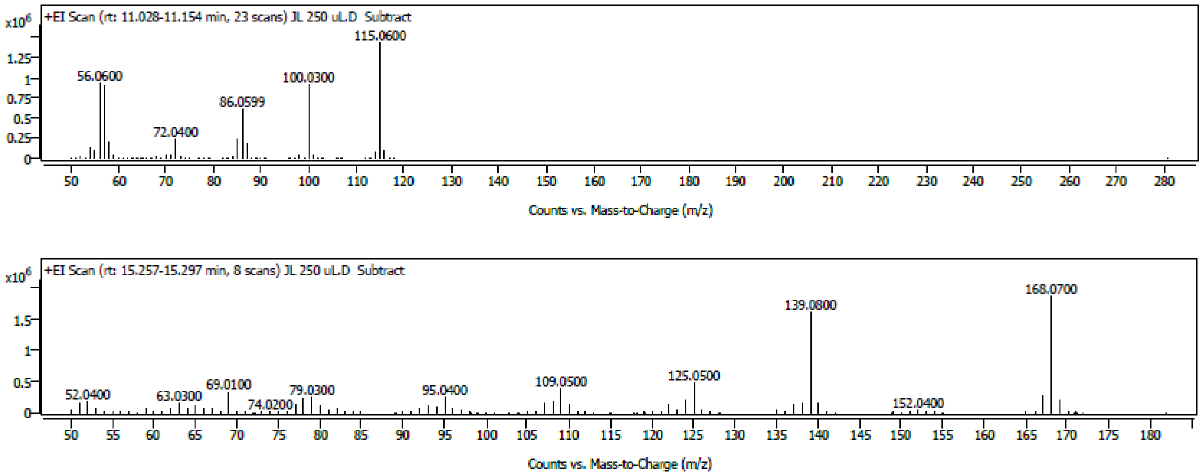

Figure S131: Mass spectra corresponding to Figure S130.

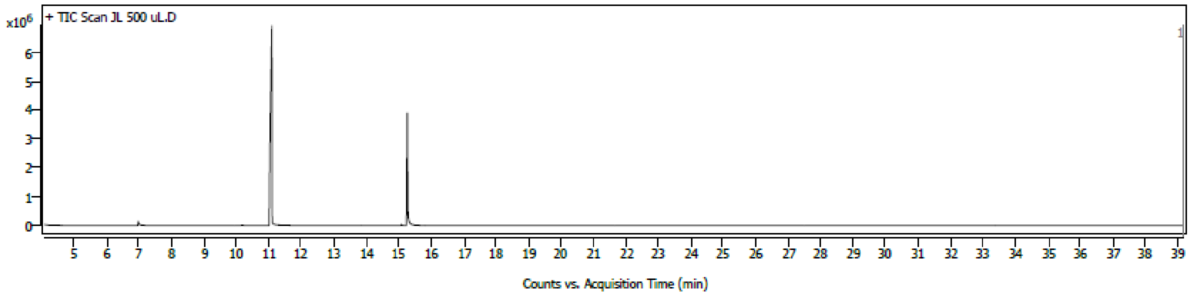

Figure S132: Gas chromatograph corresponding to decarbonylation of 2.5 M N-formylmorpholine.

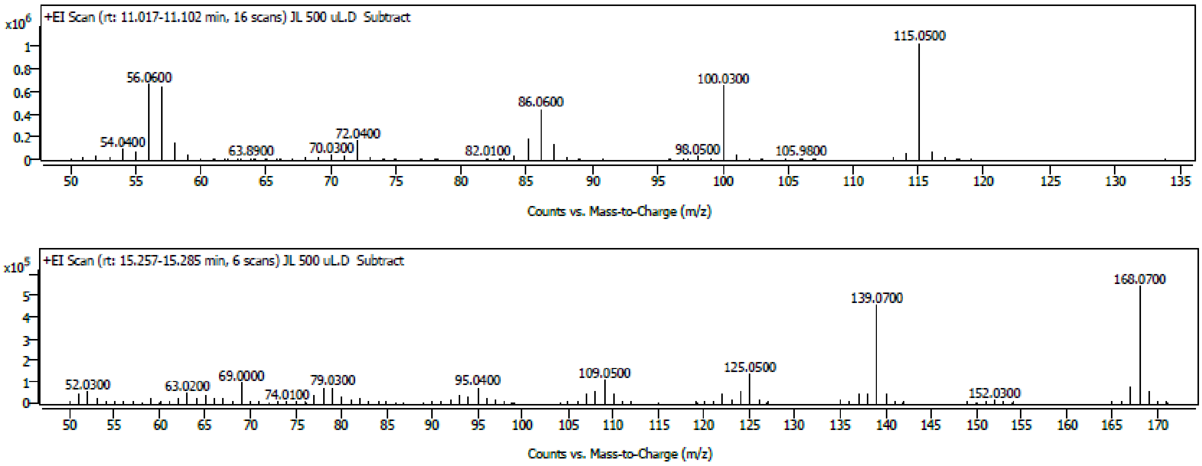

Figure S133: Mass spectra corresponding to Figure S132.

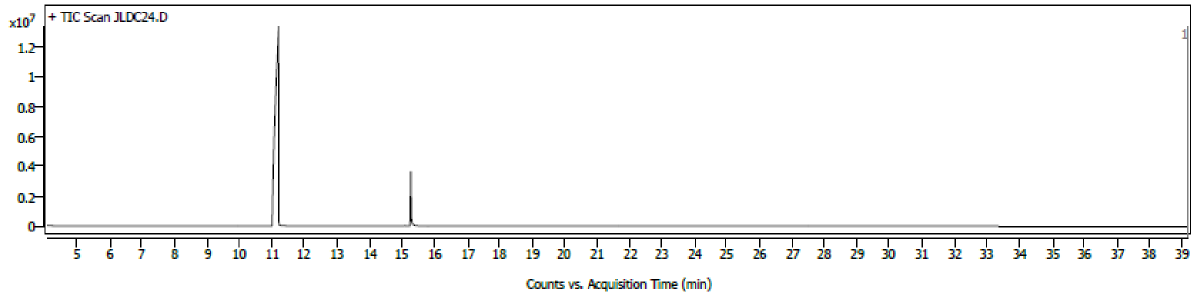

Figure S134: Gas chromatograph corresponding to decarbonylation of 4.6 M N-formylmorpholine.

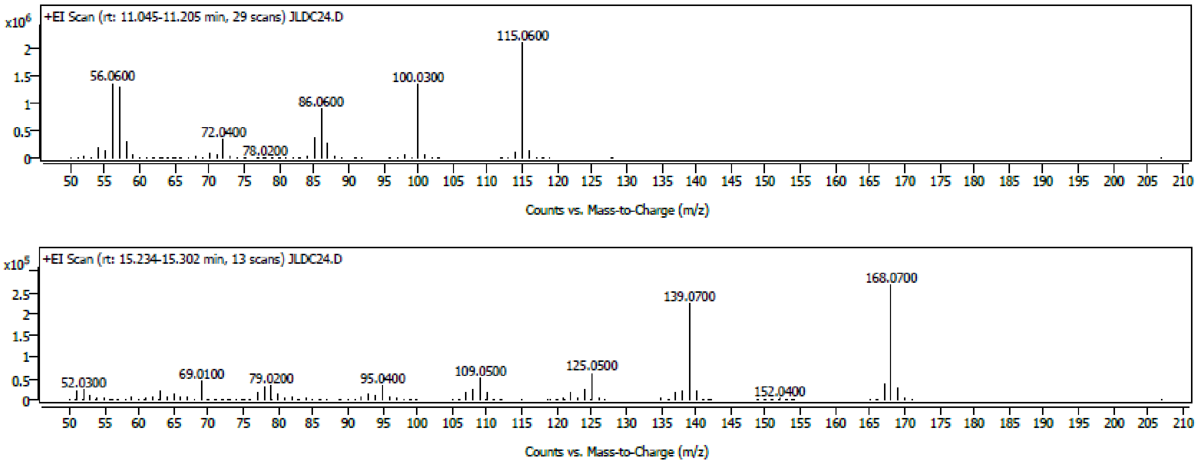

Figure S135: Mass spectra corresponding to Figure S134.

4.1.5. GC-MS data acquired from the sequential formylation and decarbonylation of morpholine

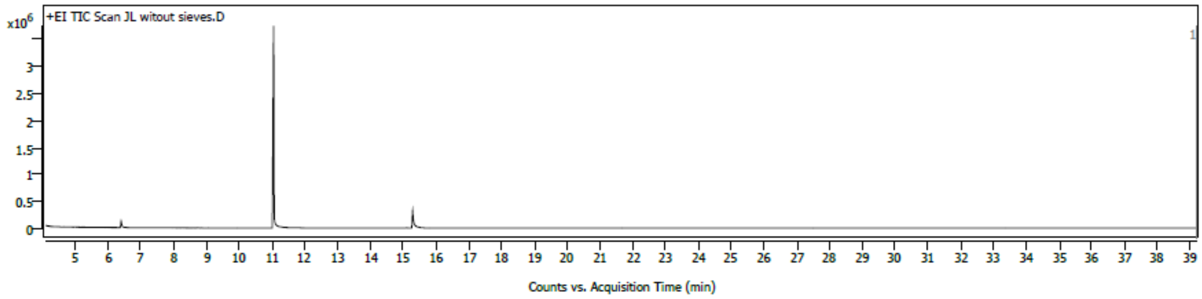

Figure S136: Gas chromatograph corresponding to sequential formylation/decarbonylation without molecular sieves present.

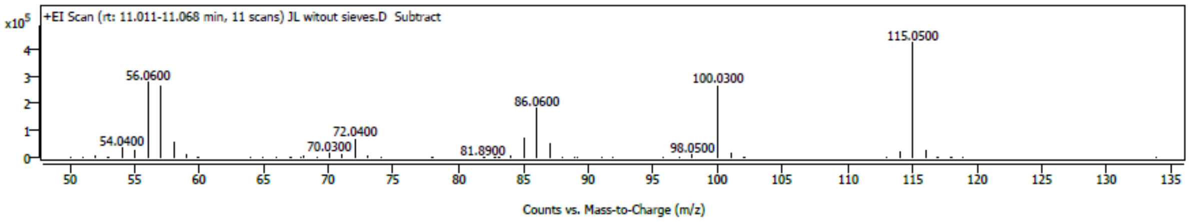

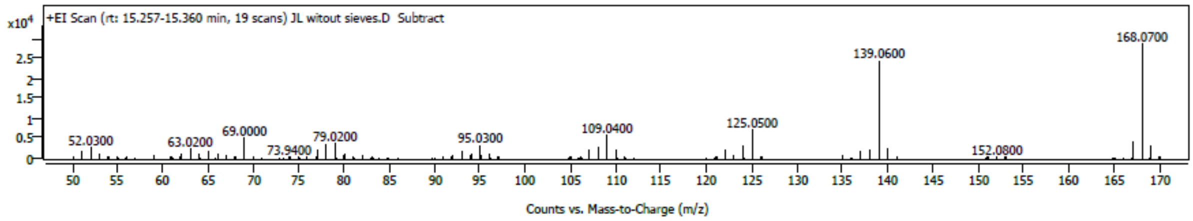

Figure S137: Mass spectra corresponding to Figure S136.

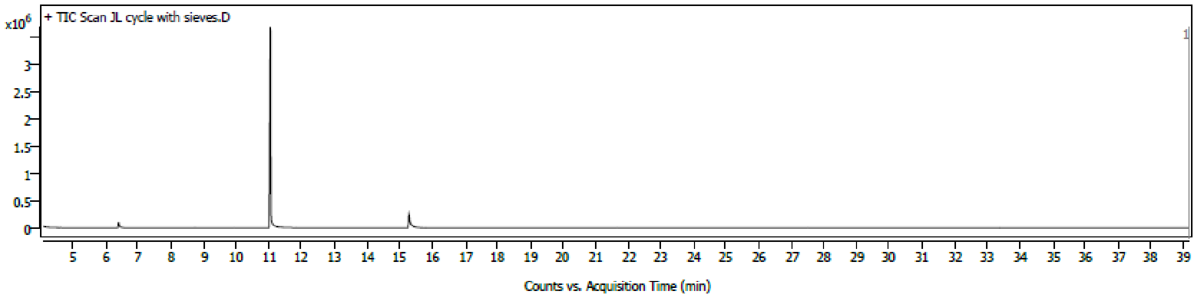

Figure S138: Gas chromatograph for sequential formylation/decarbonylation of N-formylmorpholine including drying stage over molecular sieves.

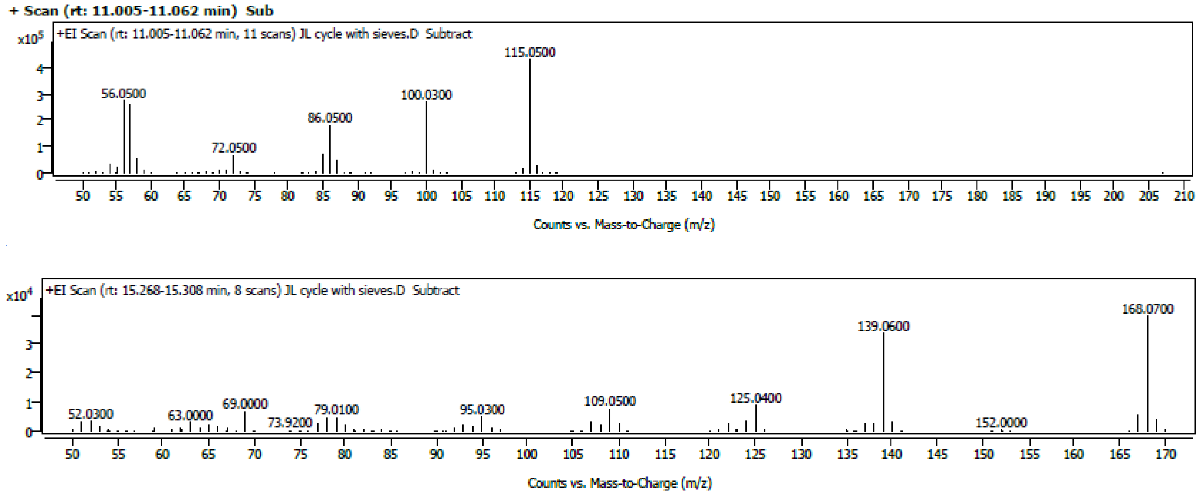

Figure S139: Mass spectra corresponding to Figure S128.

4.1.6. GC-MS data acquired from the one-pot reverse water gas shift reaction

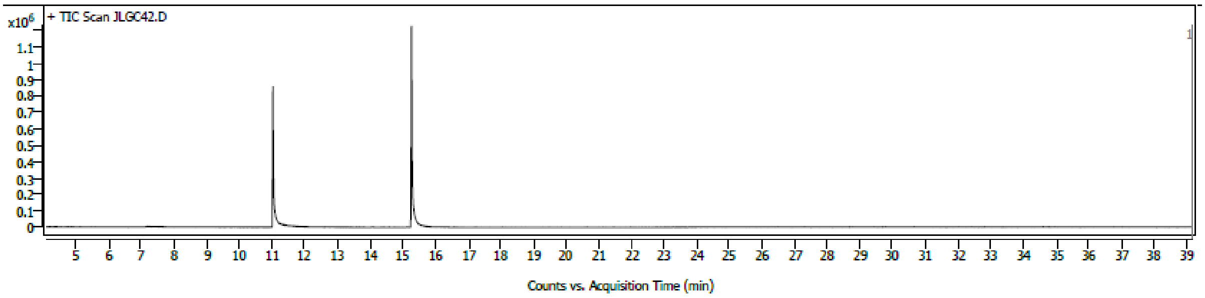

Figure S140: Gas chromatograph corresponding to Table S4, entry 1.

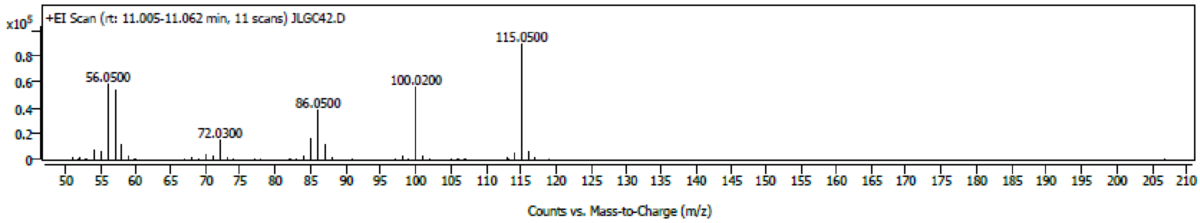

Figure S141: Mass spectra corresponding to Figure S140.

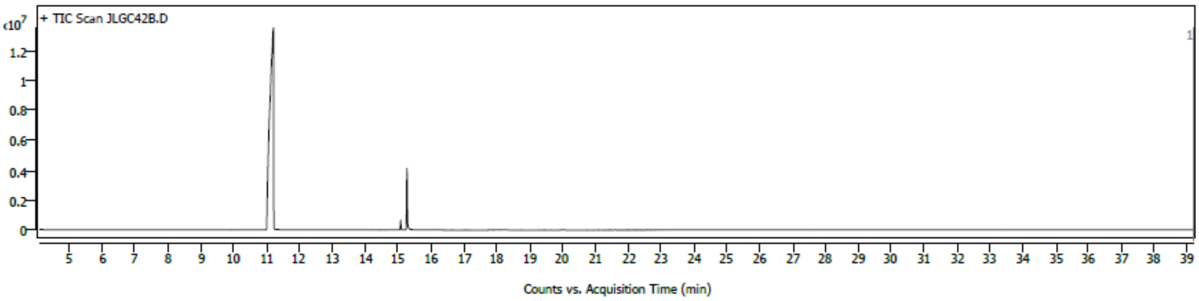

Figure S142: Gas chromatograph corresponding to Table S4, entry 2.

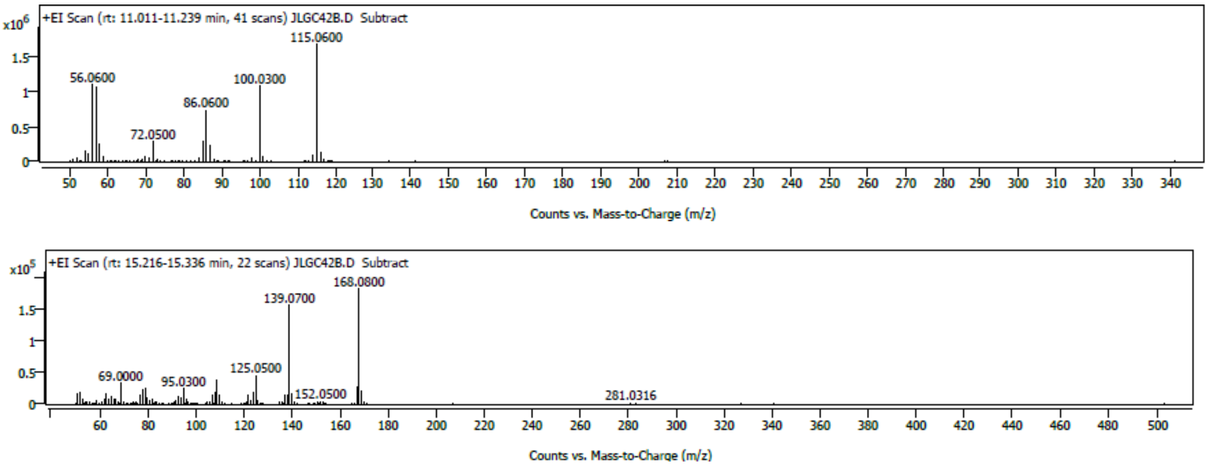

Figure S143: Mass spectra corresponding to Figure S142.

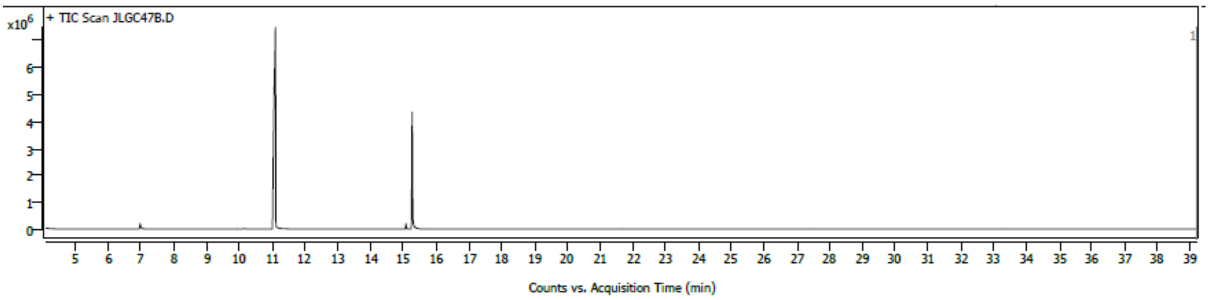

Figure S144: Gas chromatograph corresponding to Table S4, entry 3.

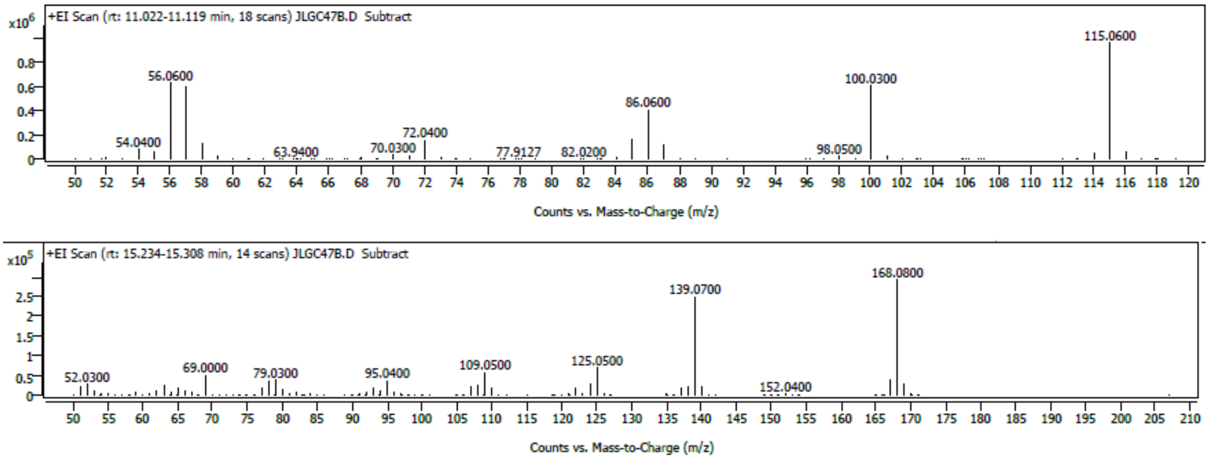

Figure S145: Mass spectra corresponding to Figure S144.

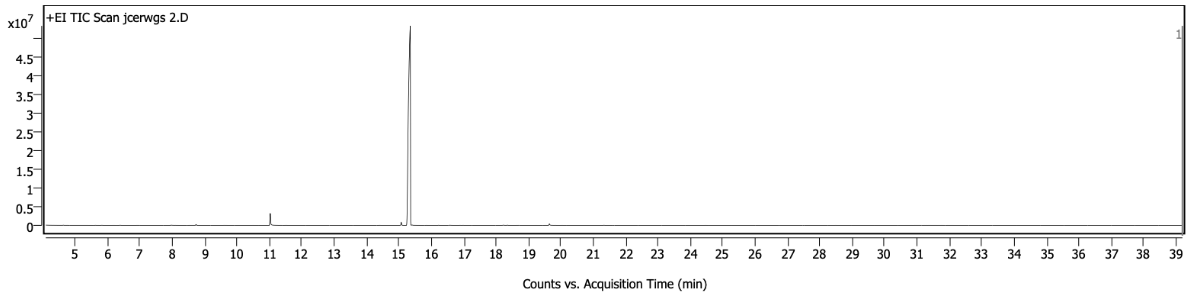

Figure S146: Gas chromatograph corresponding to Table S4, entry 4.

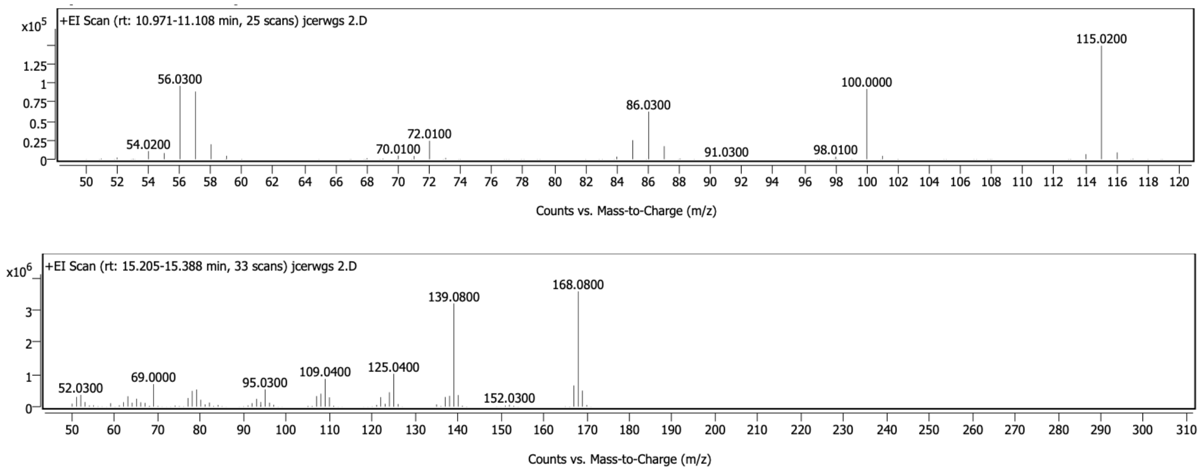

Figure S147: Mass spectra corresponding to Figure S146.

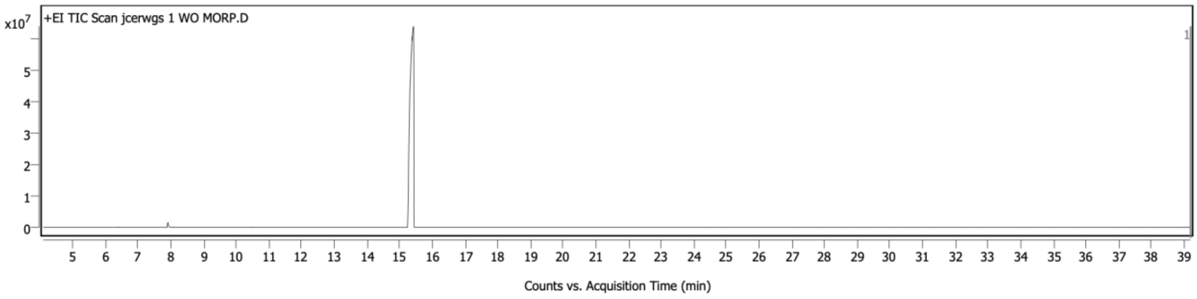

Figure S148: Gas chromatograph corresponding to Table S4, entry 5.

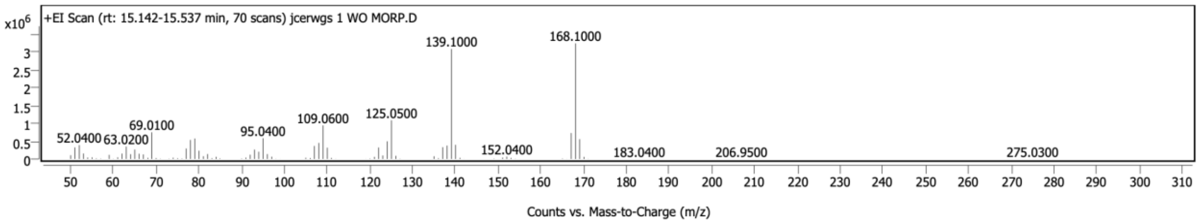

Figure S149: Mass spectrum corresponding to Figure S148.

5. GC-TCD data

5.1. GC-TCD data acquired from decarbonylation of N-formyl morpholine

Peaks at 2.9 and 3.3 min are due to trace air induced during gas sampling.

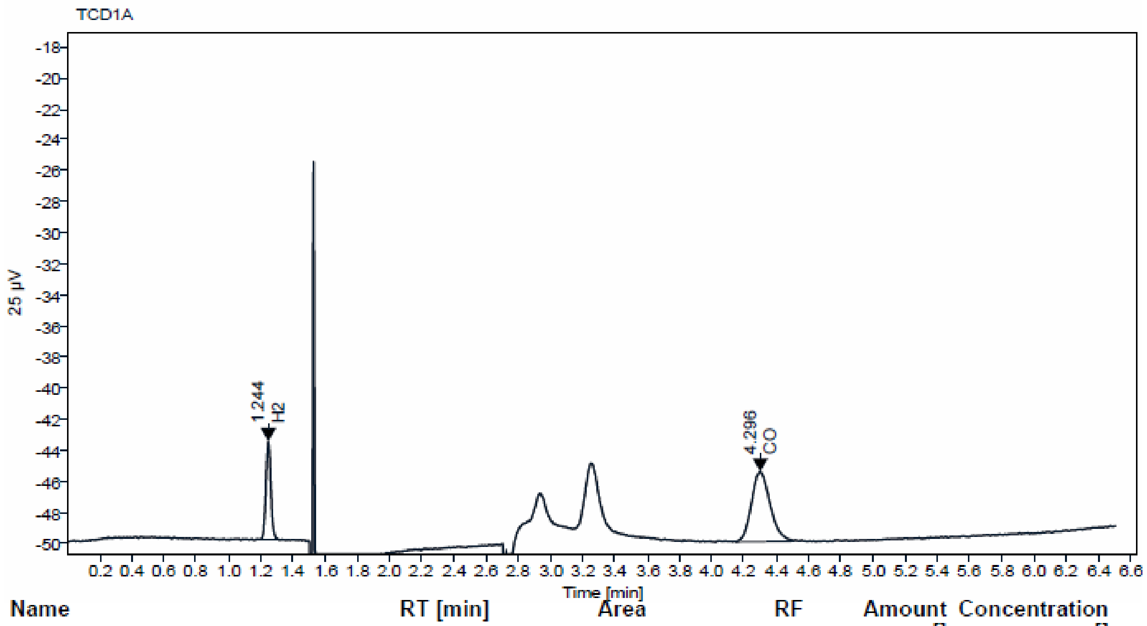

Name	RT [min]	Area	RF	Amount []	Concentration []
H2	1.244	13.6638	433.43612	0.032	0.0315
CO	4.296	36.3027	38.48605	0.943	0.9433

Figure S150: GC-TCD data corresponding to Table S2, entry 1.

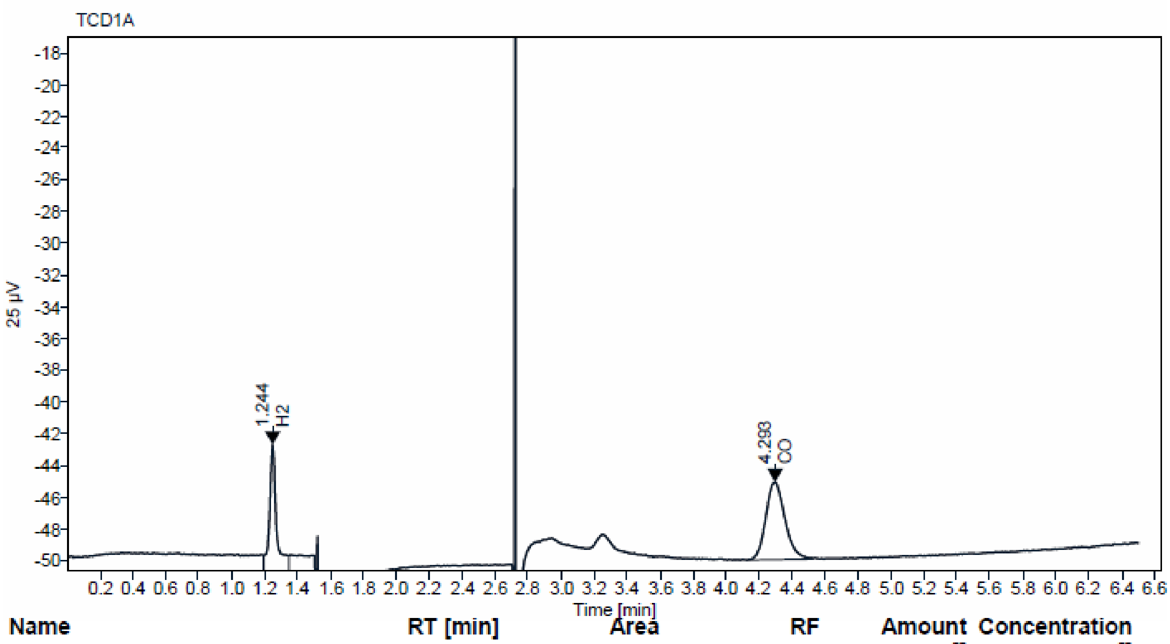

Name	RT [min]	Area	RF	Amount []	Concentration []
H2	1.244	41.9935	436.09330	0.096	0.0963
CO	4.293	39.3472	38.75112	1.015	1.0154

Figure S151: GC-TCD data corresponding to Table S2, entry 2.

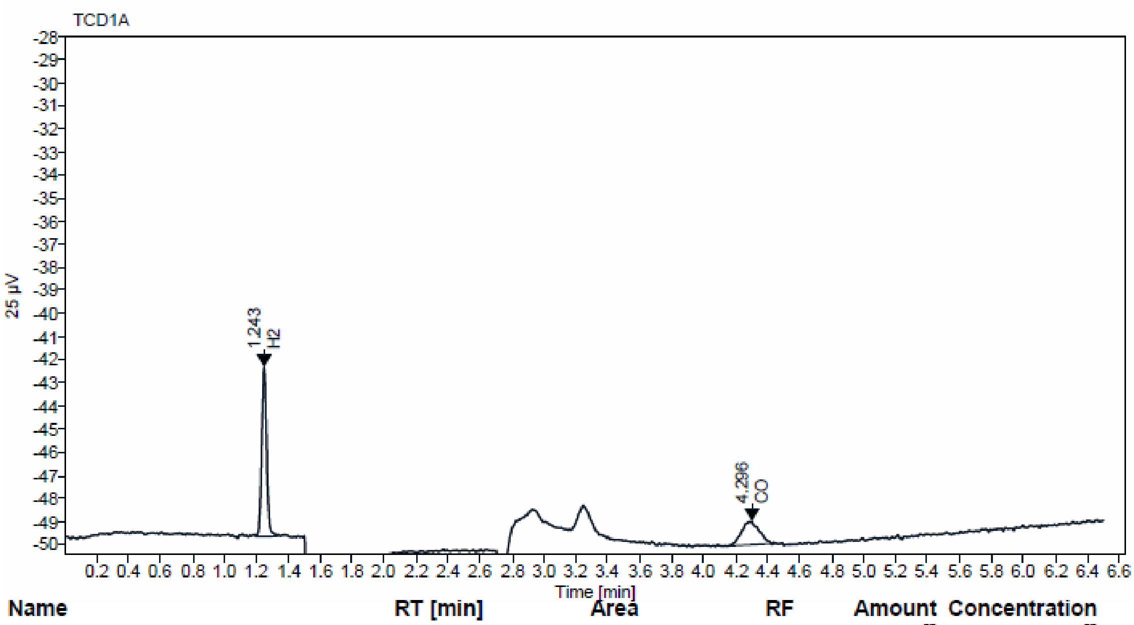

Name	RT [min]	Area	RF	Amount []	Concentration []
H2	1.243	15.8091	433.63848	0.036	0.0365
CO	4.296	7.7814	35.81738	0.217	0.2173

Figure S152: GC-TCD data corresponding to Table S2, entry 3.

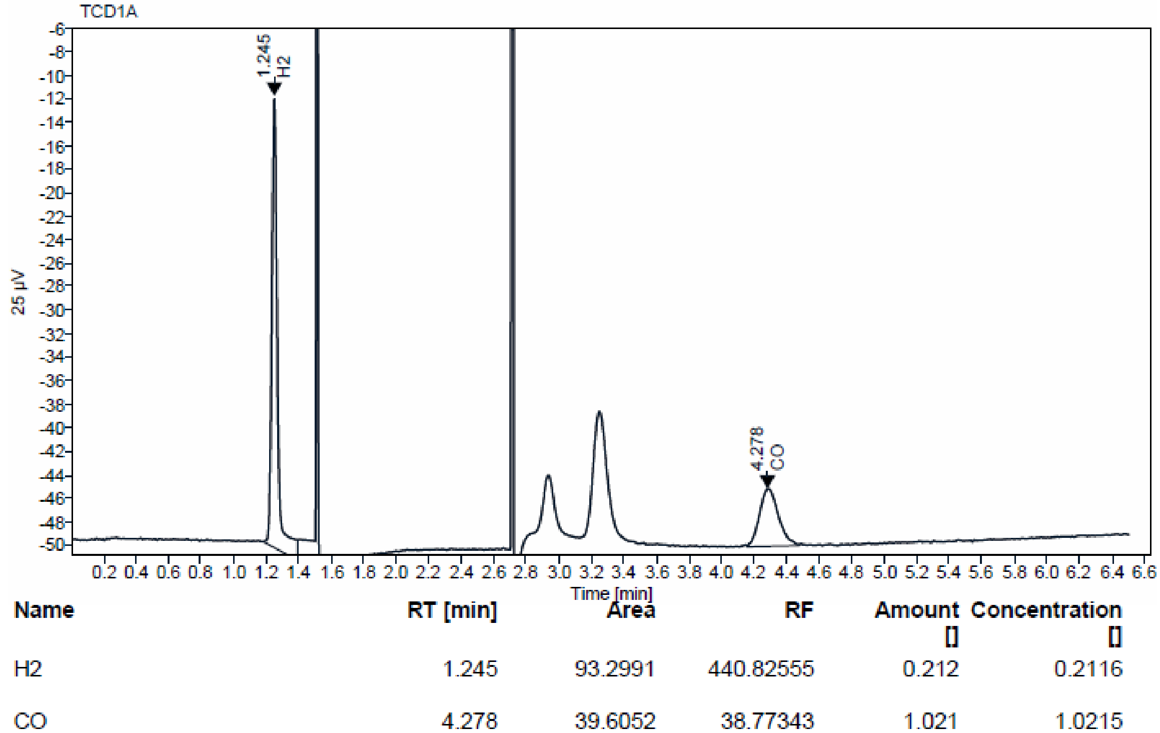

Name	RT [min]	Area	RF	Amount []	Concentration []
H2	1.245	93.2991	440.82555	0.212	0.2116
CO	4.278	39.6052	38.77343	1.021	1.0215

Figure S153: GC-TCD data corresponding to Table S2, entry 4.

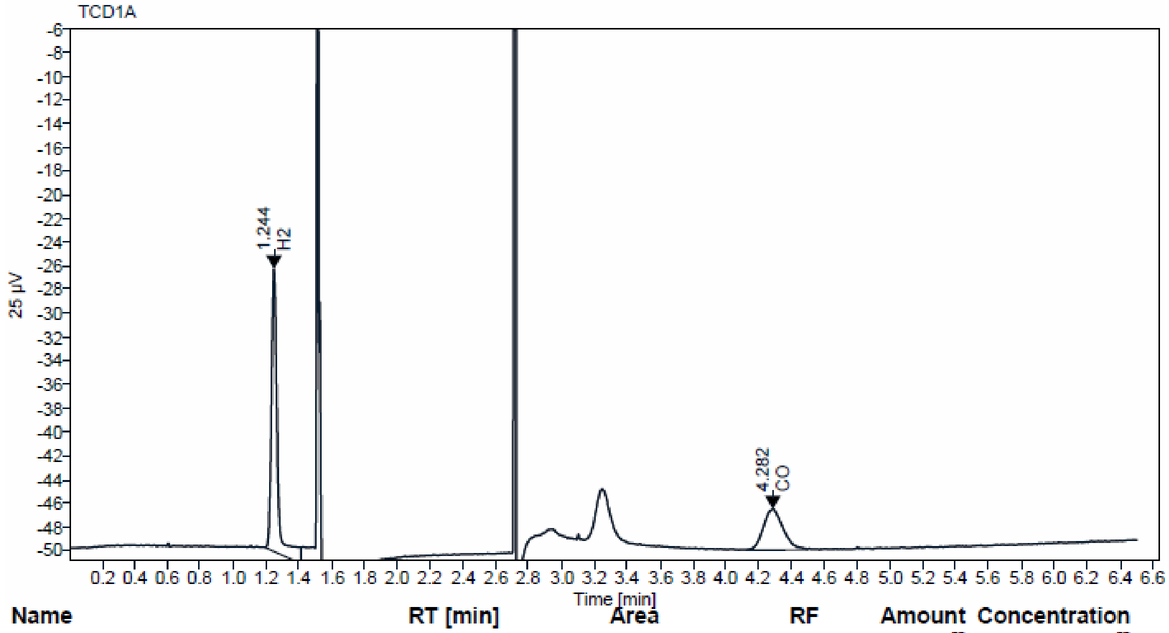

Name	RT [min]	Area	RF	Amount []	Concentration []
H2	1.244	59.4682	437.71645	0.136	0.1359
CO	4.282	28.7268	37.81143	0.760	0.7597

Figure S154: GC-TCD data corresponding to Table S2, entry 5.

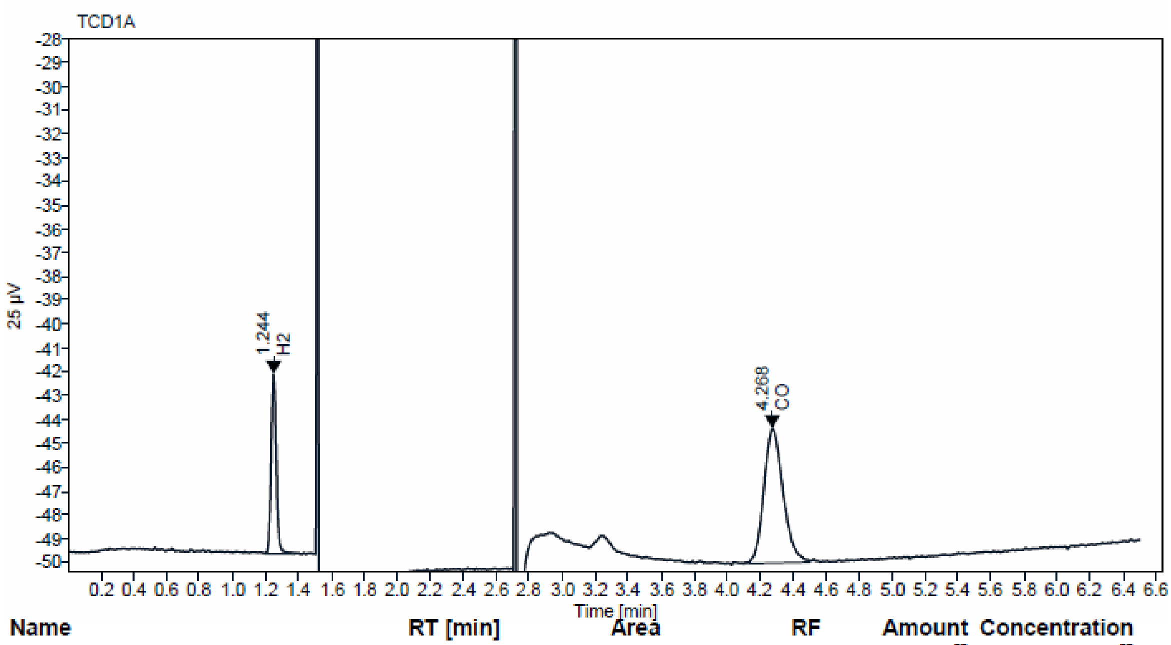

Name	RT [min]	Area	RF	Amount []	Concentration []
H2	1.244	16.5027	433.70386	0.038	0.0381
CO	4.268	46.1119	39.32856	1.172	1.1725

Figure S155: GC-TCD data corresponding to Table S2, entry 6.

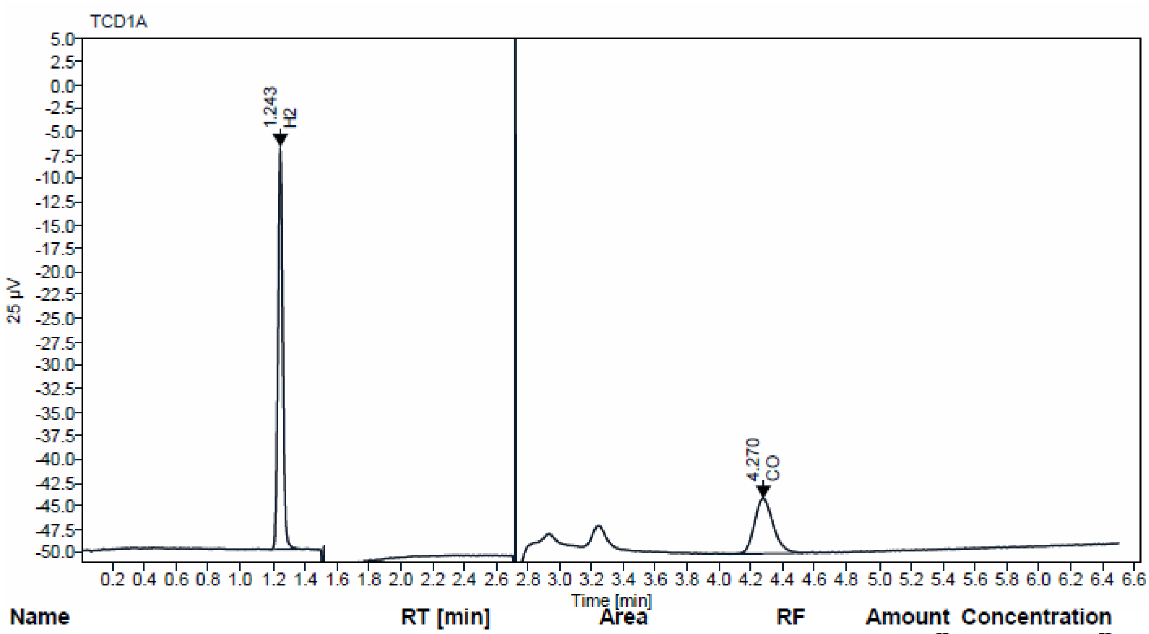

Name	RT [min]	Area	RF	Amount []	Concentration []
H2	1.243	91.4971	440.66103	0.208	0.2076
CO	4.270	47.8275	39.47260	1.212	1.2117

Figure S156: GC-TCD data corresponding to Table S2, entry 7.

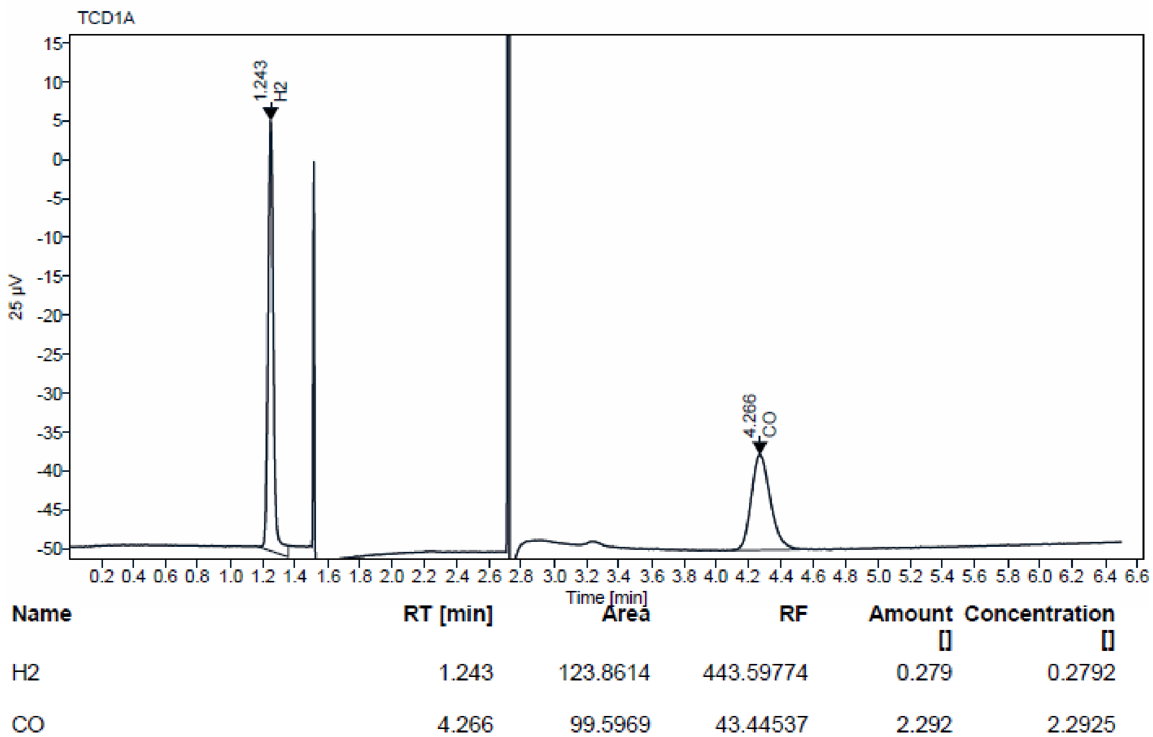

Name	RT [min]	Area	RF	Amount []	Concentration []
H2	1.243	123.8614	443.59774	0.279	0.2792
CO	4.266	99.5969	43.44537	2.292	2.2925

Figure S157: GC-TCD data corresponding to Table S2, entry 8.

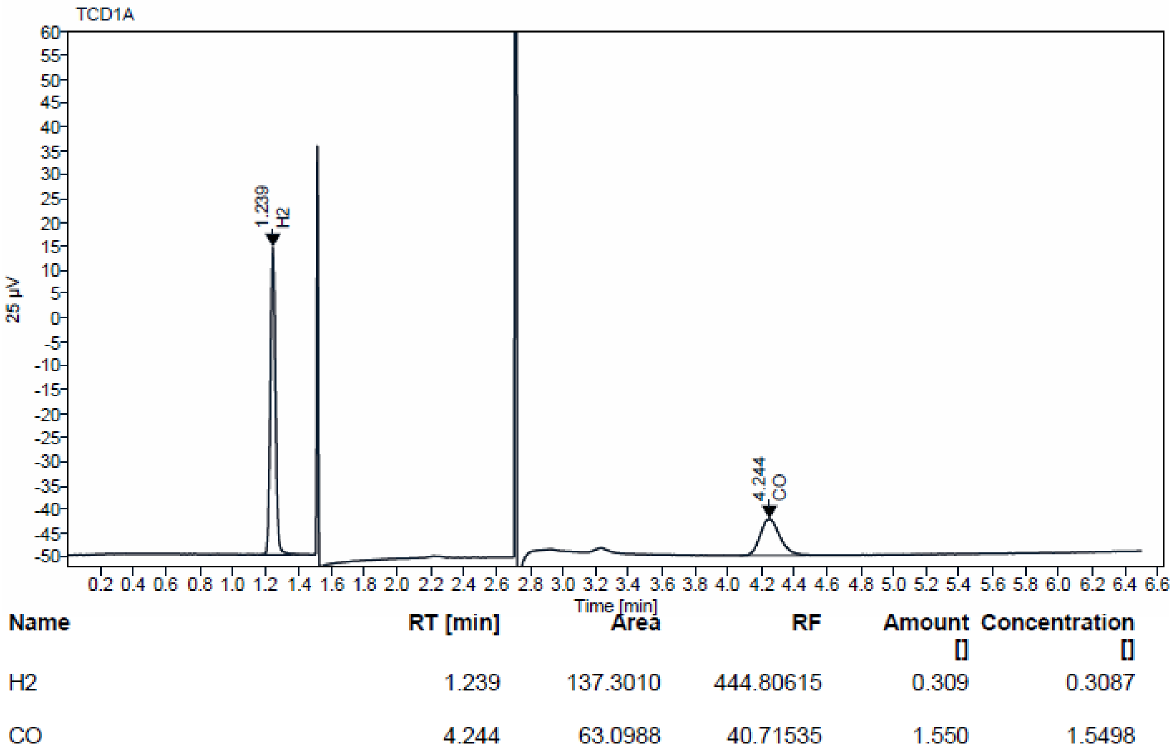

Name	RT [min]	Area	RF	Amount []	Concentration []
H2	1.239	137.3010	444.80615	0.309	0.3087
CO	4.244	63.0988	40.71535	1.550	1.5498

Figure S158: GC-TCD data corresponding to Table S2, entry 9.

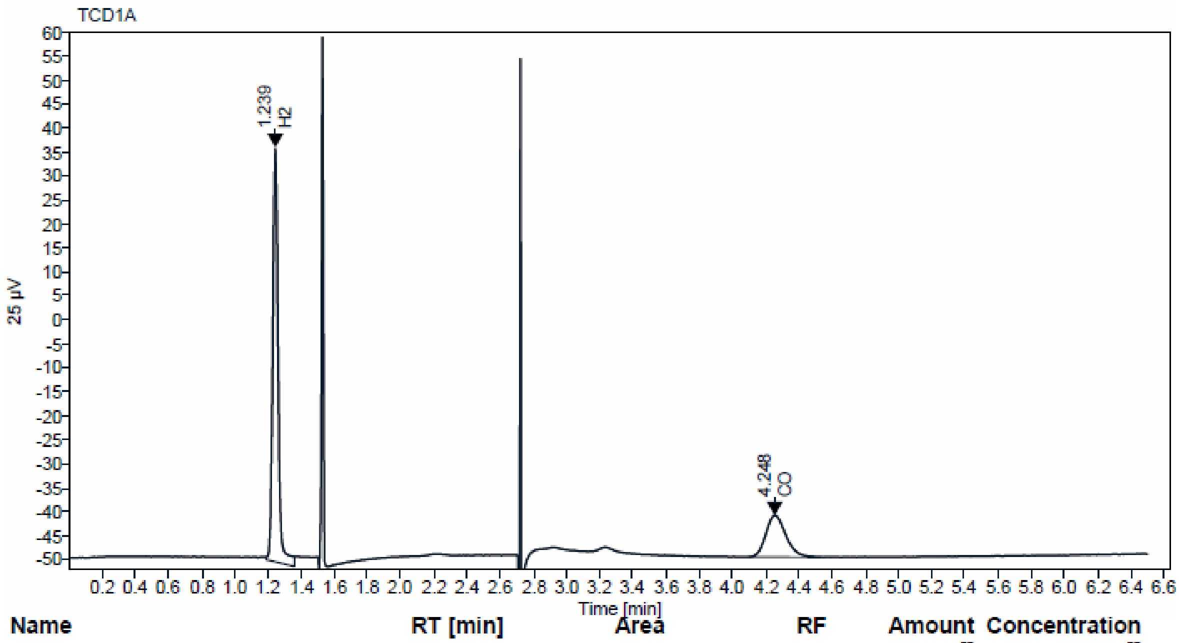

Name	RT [min]	Area	RF	Amount []	Concentration []
H2	1.239	194.9285	449.91693	0.433	0.4333
CO	4.248	73.3988	41.51724	1.768	1.7679

Figure S159: GC-TCD data corresponding to Table S2, entry 10.

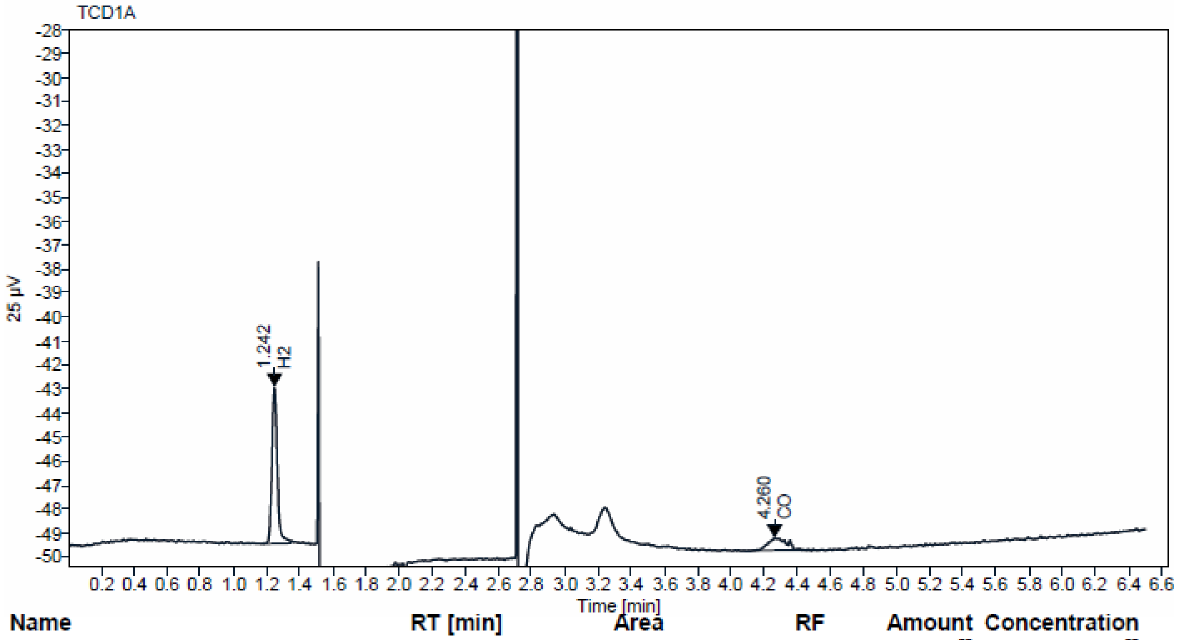

Name	RT [min]	Area	RF	Amount []	Concentration []
H2	1.242	14.6960	433.53350	0.034	0.0339
CO	4.260	3.8680	35.42022	0.109	0.1092

Figure S160: GC-TCD data corresponding to Table S2, entry 11.

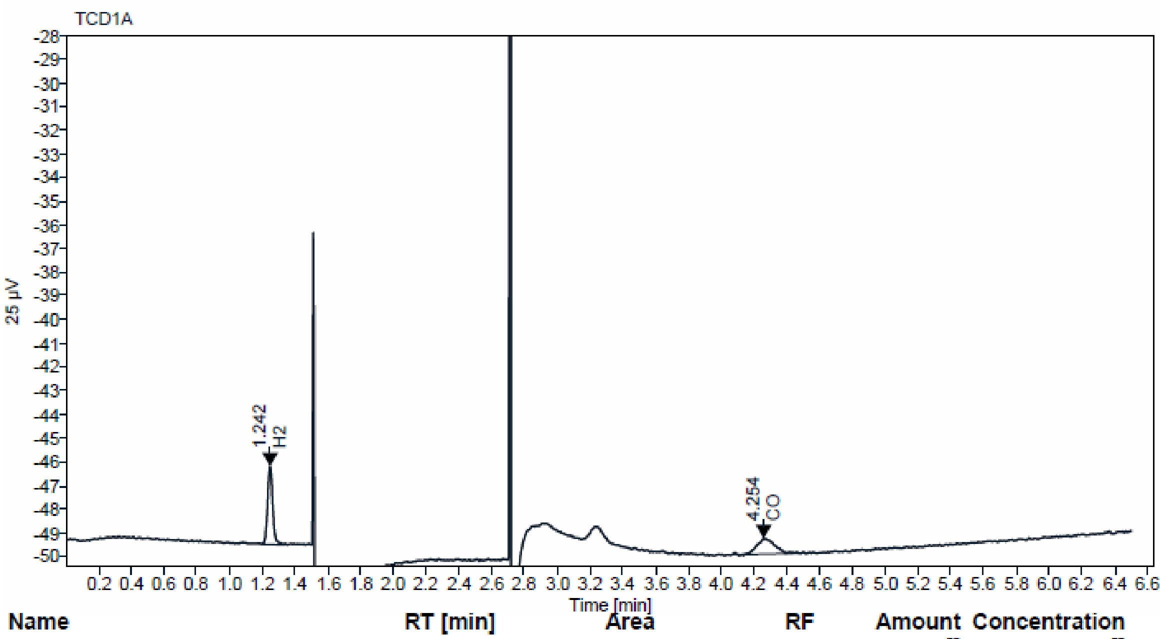

Name	RT [min]	Area	RF	Amount []	Concentration []
H2	1.242	7.1646	432.82193	0.017	0.0166
CO	4.254	4.9565	35.53156	0.139	0.1395

Figure S161: GC-TCD data corresponding to Table S2, entry 12.

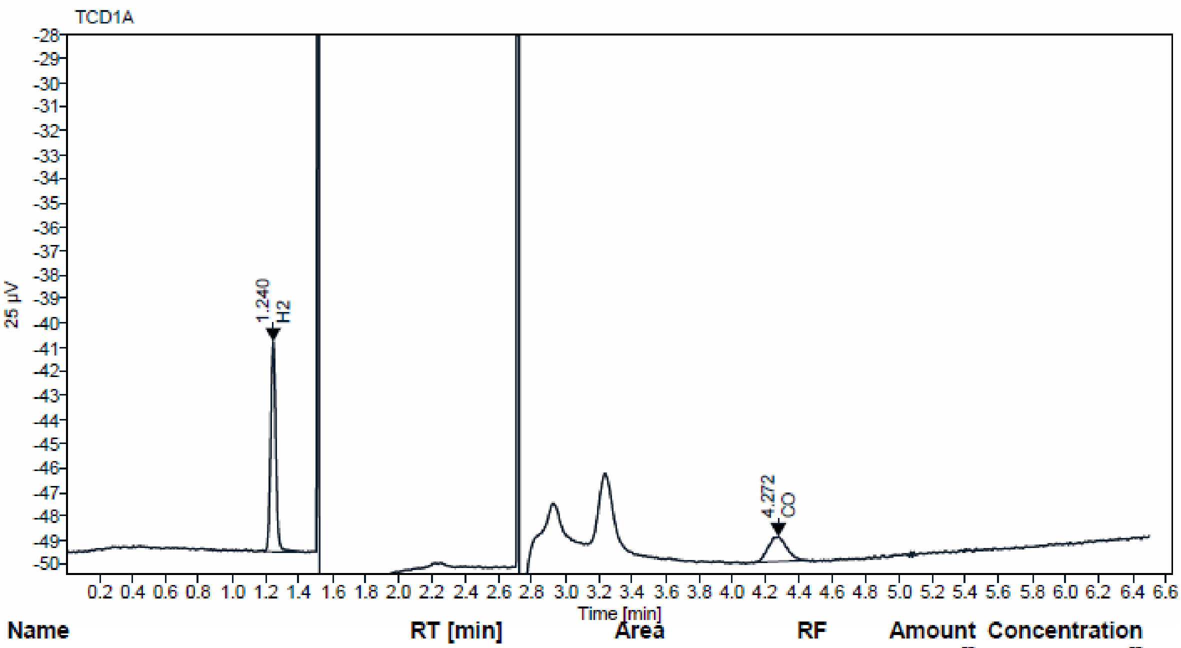

Name	RT [min]	Area	RF	Amount []	Concentration []
H2	1.240	18.8365	433.92371	0.043	0.0434
CO	4.272	7.8471	35.82398	0.219	0.2190

Figure S162: GC-TCD data corresponding to Table S2, entry 13.

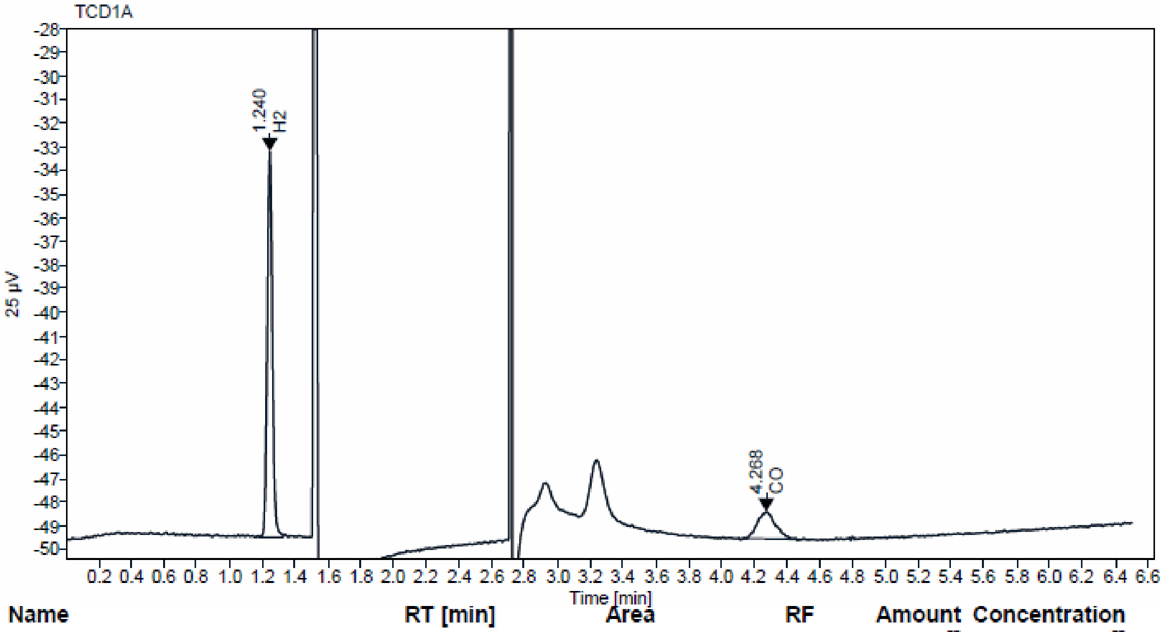

Name	RT [min]	Area	RF	Amount []	Concentration []
H2	1.240	34.8668	435.42788	0.080	0.0801
CO	4.268	8.7479	35.91415	0.244	0.2436

Figure S163: GC-TCD data corresponding to Table S2, entry 14.

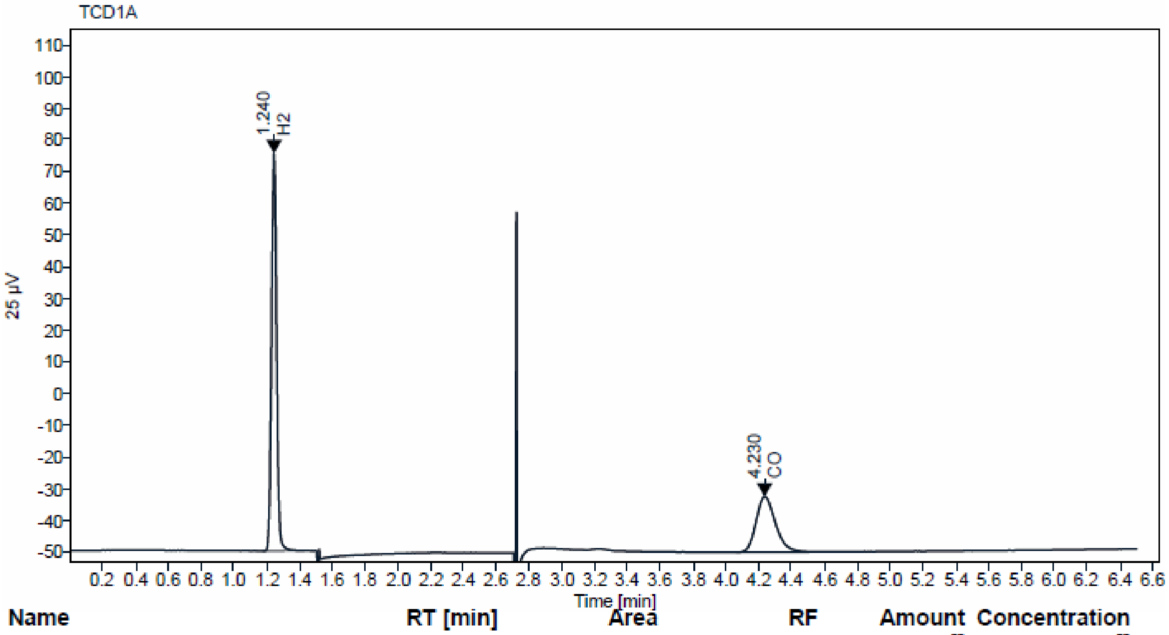

Name	RT [min]	Area	RF	Amount []	Concentration []
H2	1.240	267.4933	456.19776	0.586	0.5864
CO	4.230	143.7523	46.40541	3.098	3.0977

Figure S164: GC-TCD data corresponding to Table S2, entry 15.

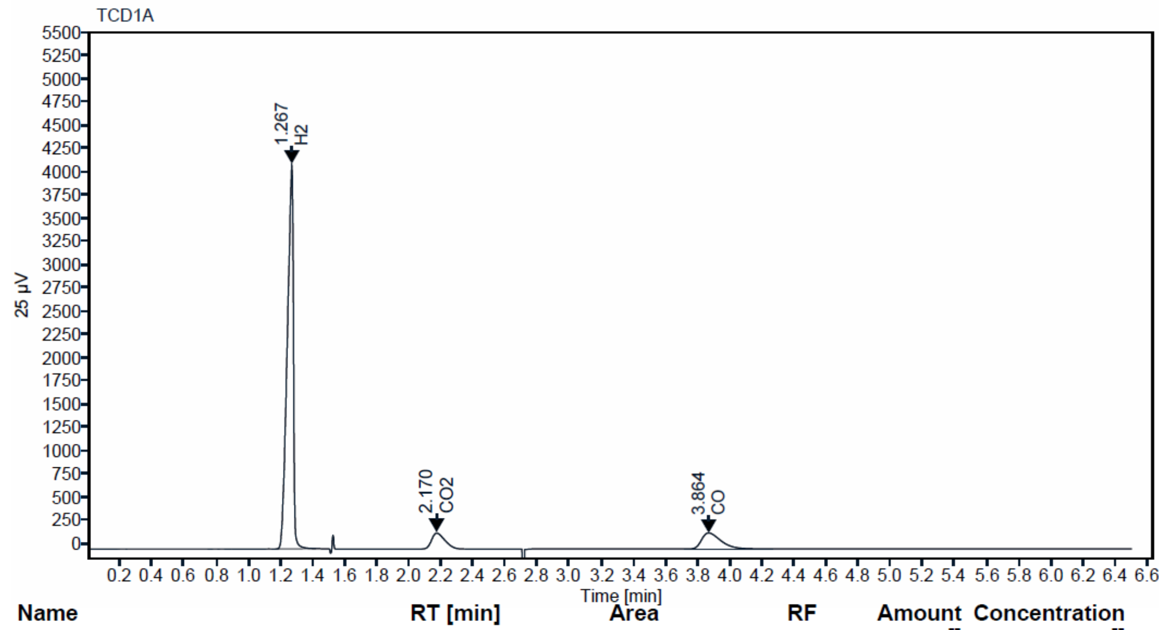

Name	RT [min]	Area	RF	Amount []	Concentration []
H2	1.267	10873.2854	455.26827	23.883	23.8832
CO	3.864	1449.4080	38.67660	37.475	37.4751
CO2	2.170	994.8240	46.05372	21.601	216.0138

Figure S165: GC-TCD data corresponding WGS control reaction.

5.2. GC-TCD data acquired from the decarbonylation of formamides

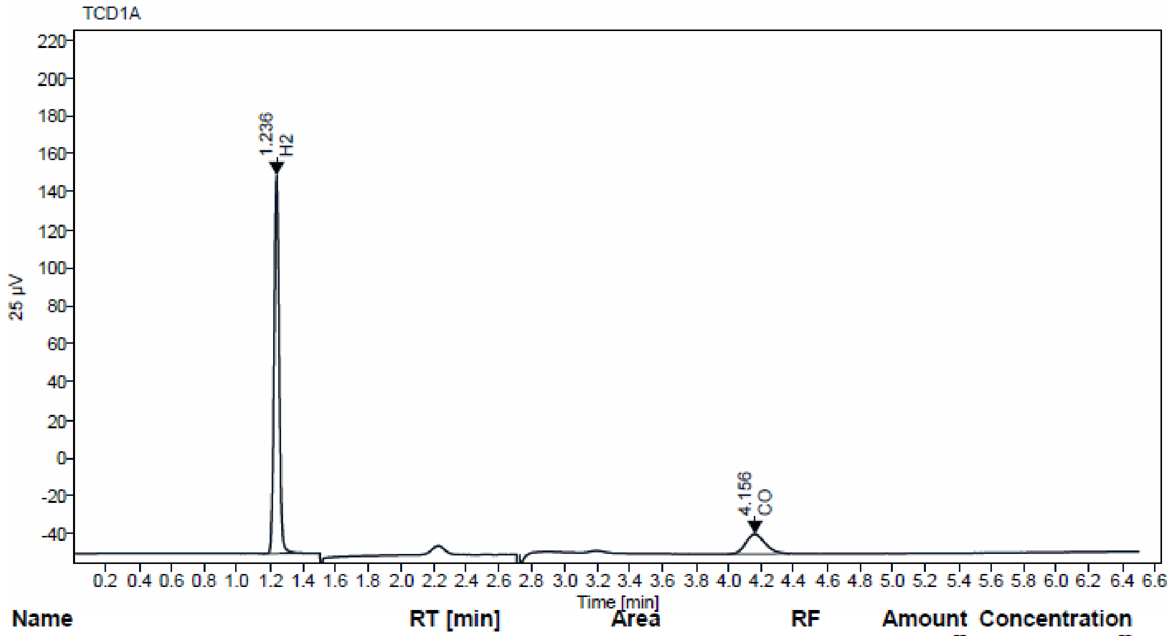

Name	RT [min]	Area	RF	Amount []	Concentration []
H2	1.236	429.2848	469.64210	0.914	0.9141
CO	4.156	86.5589	42.50439	2.036	2.0365

Figure S166: GC-TCD data corresponding to Table S3, entry 1.

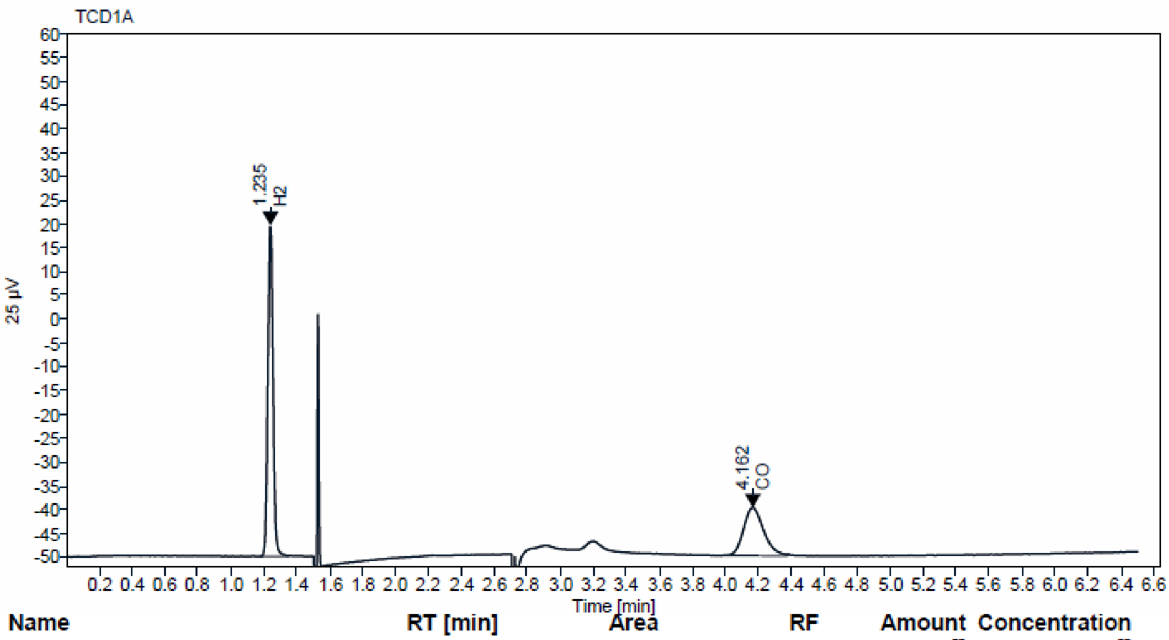

Name	RT [min]	Area	RF	Amount []	Concentration []
H2	1.235	149.5063	445.89808	0.335	0.3353
CO	4.162	85.6170	42.43504	2.018	2.0176

Figure S167: GC-TCD data corresponding to Table S3, entry 2.

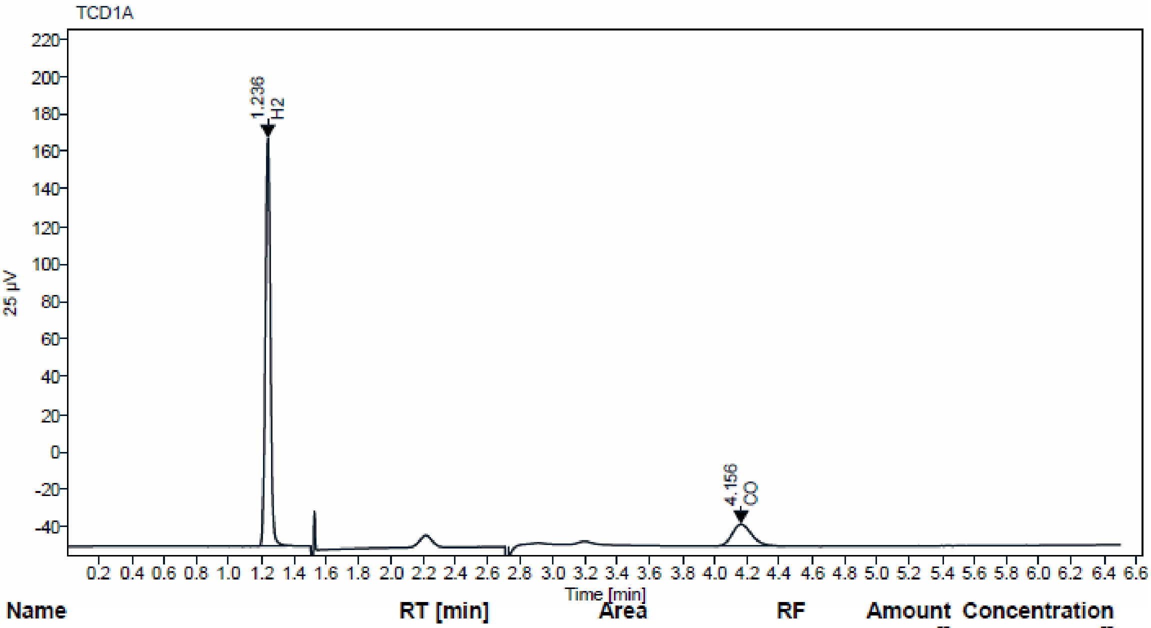

Name	RT [min]	Area	RF	Amount []	Concentration []
H2	1.236	467.6098	472.72363	0.989	0.9892
CO	4.156	98.6098	43.37533	2.273	2.2734

Figure S168: GC-TCD data corresponding to Table S3, entry 3.

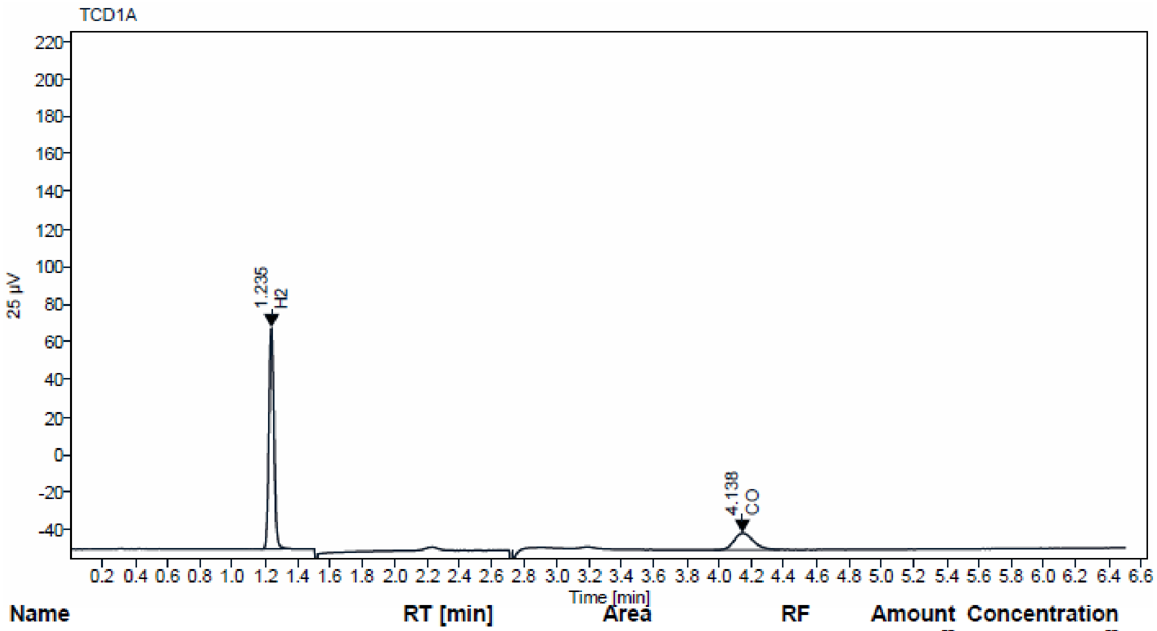

Name	RT [min]	Area	RF	Amount []	Concentration []
H2	1.235	252.2770	454.89441	0.555	0.5546
CO	4.138	73.9096	41.55631	1.779	1.7785

Figure S169: GC-TCD data corresponding to Table S3, entry 4.

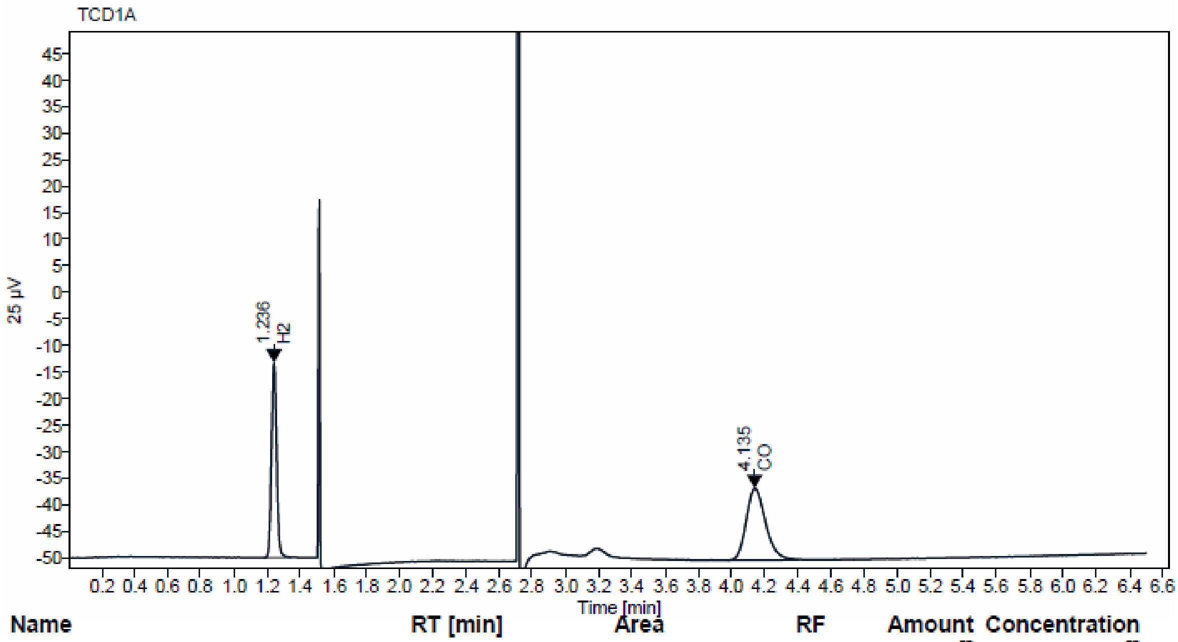

Name	RT [min]	Area	RF	Amount []	Concentration []
H2	1.236	78.5251	439.47312	0.179	0.1787
CO	4.135	109.3830	44.12980	2.479	2.4787

Figure S170: GC-TCD data corresponding to Table S3, entry 5.

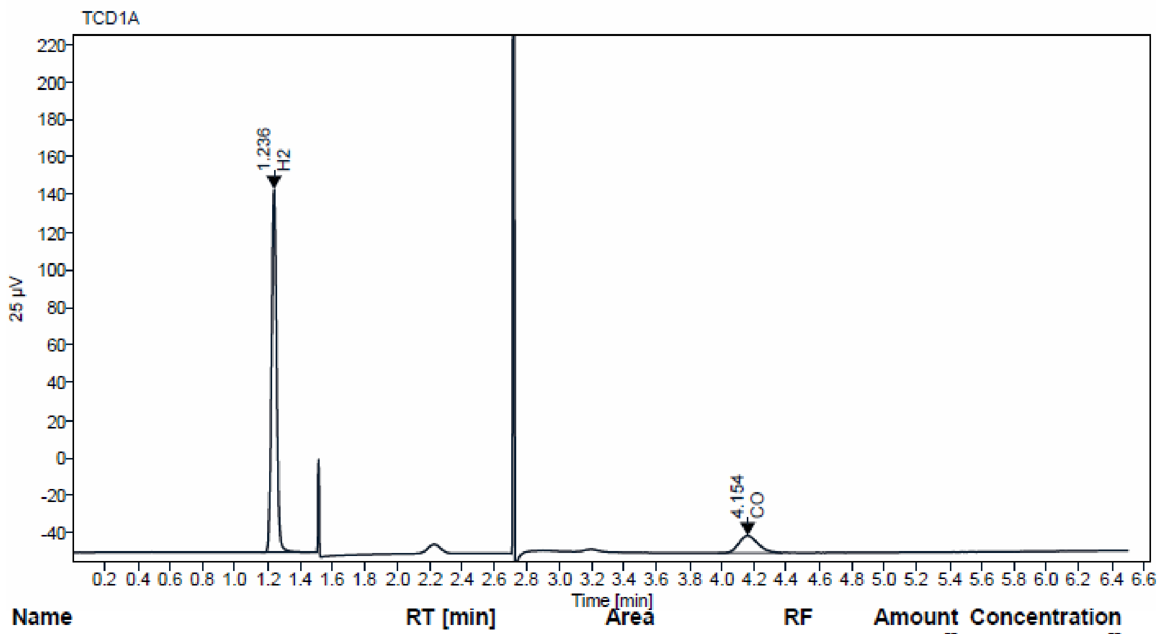

Name	RT [min]	Area	RF	Amount []	Concentration []
H2	1.236	416.0709	468.57092	0.888	0.8880
CO	4.154	76.3608	41.74293	1.829	1.8293

Figure S171: GC-TCD data corresponding to Table S3, entry 6.

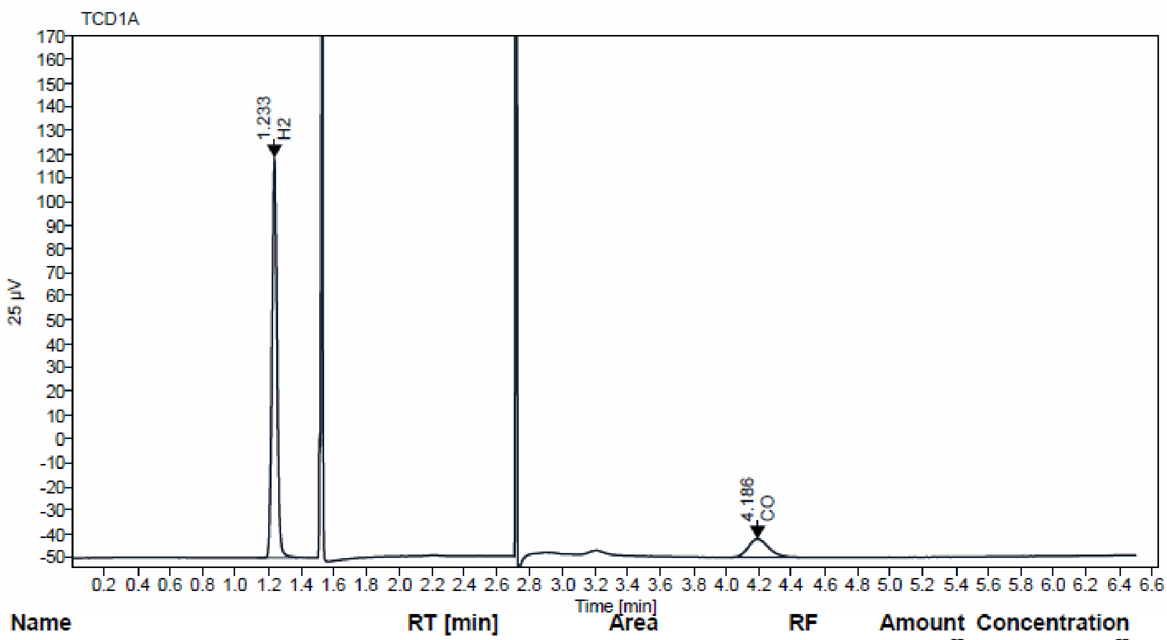

Name	RT [min]	Area	RF	Amount []	Concentration []
H2	1.233	367.6498	464.60620	0.791	0.7913
CO	4.186	68.1501	41.11201	1.658	1.6577

Figure S172: GC-TCD data corresponding to Table S3, entry 7

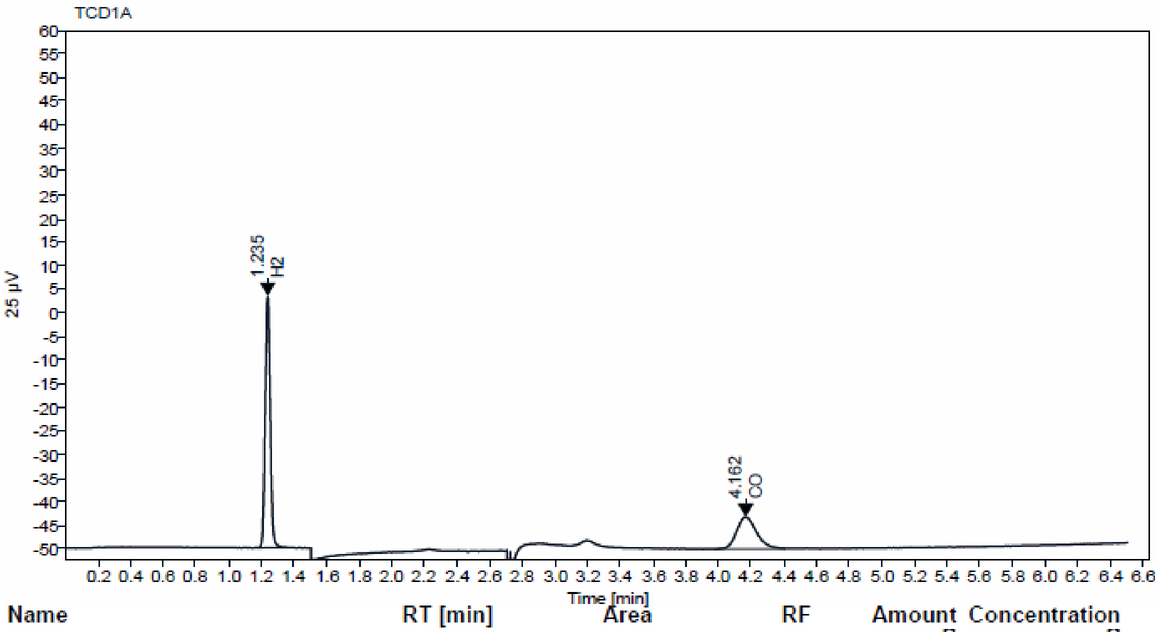

Name	RT [min]	Area	RF	Amount []	Concentration []
H2	1.235	114.2918	442.73336	0.258	0.2582
CO	4.162	57.2872	40.25042	1.423	1.4233

Figure S173: GC-TCD data corresponding to Table S3, entry 8.

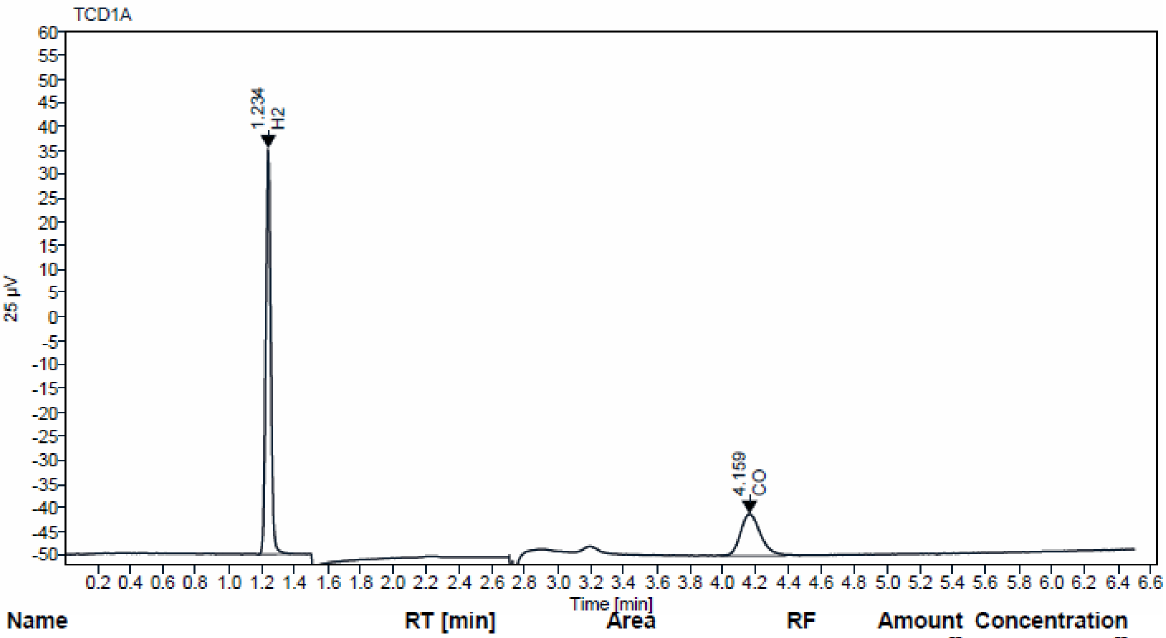

Name	RT [min]	Area	RF	Amount []	Concentration []
H2	1.234	182.9693	448.86553	0.408	0.4076
CO	4.159	73.9120	41.55650	1.779	1.7786

Figure S174: GC-TCD data corresponding to Table S3, entry 9.

5.3. GC-TCD data acquired from the decarbonylation of N-formyl morpholine at different morpholine concentrations

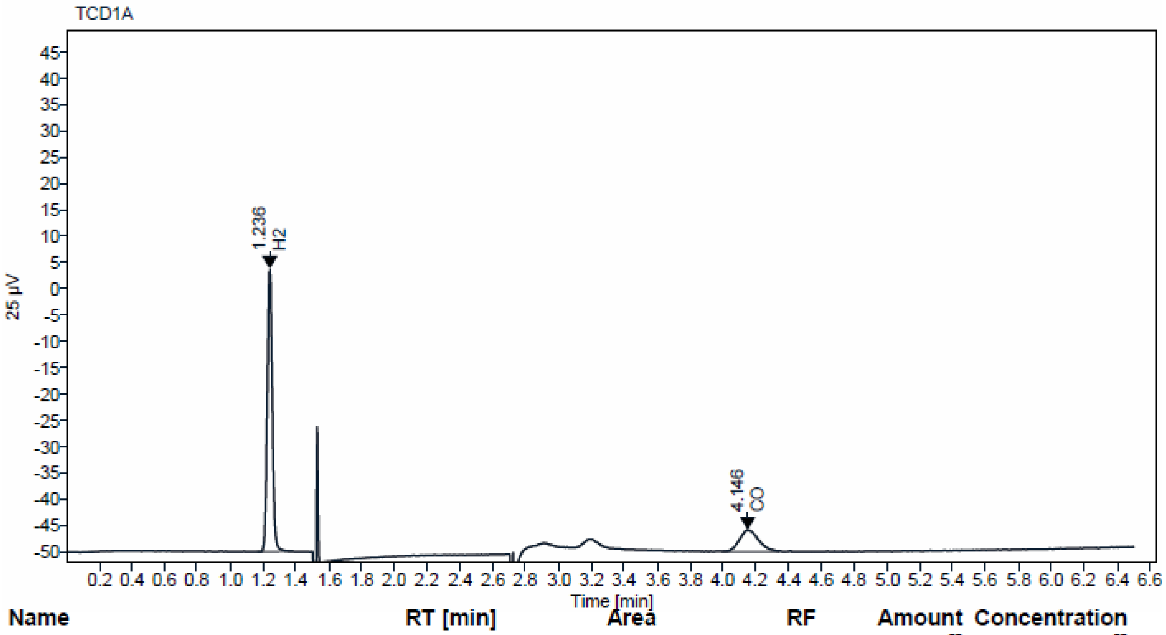

Name	RT [min]	Area	RF	Amount []	Concentration []
H2	1.236	115.0868	442.80530	0.260	0.2599
CO	4.146	33.1227	38.20556	0.867	0.8670

Figure S175: GC-TCD data for decarbonylation of 0.125 M N-formylmorpholine under standard conditions.

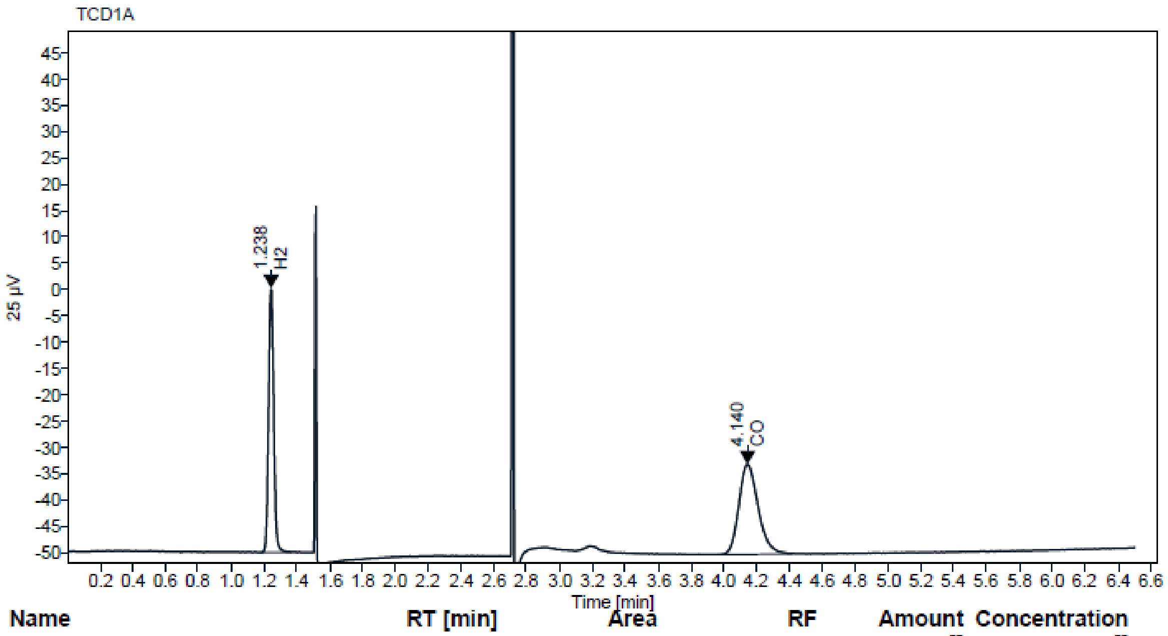

Name	RT [min]	Area	RF	Amount []	Concentration []
H2	1.238	108.0652	442.16917	0.244	0.2444
CO	4.140	139.6587	46.14386	3.027	3.0266

Figure S176: GC-TCD data for decarbonylation of 1.25 M N-formylmorpholine under standard conditions.

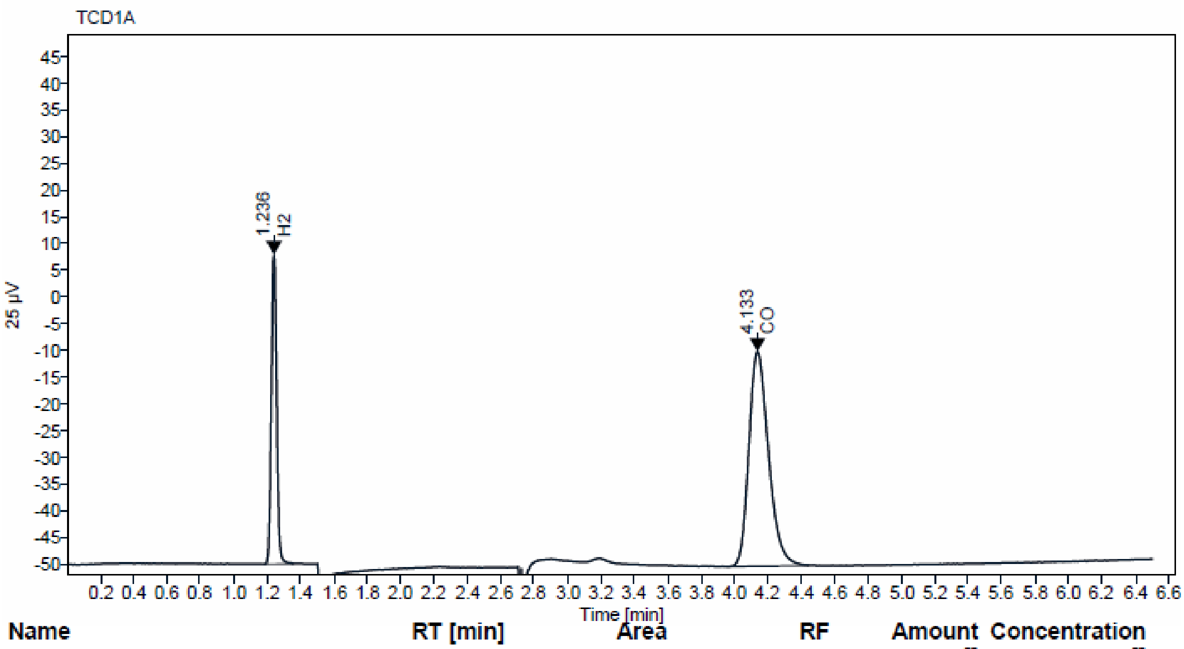

Name	RT [min]	Area	RF	Amount []	Concentration []
H2	1.236	124.5388	443.65880	0.281	0.2807
CO	4.133	327.6224	56.37896	5.811	5.8111

Figure S177: GC-TCD data for decarbonylation of 2.5 M N-formylmorpholine under standard conditions.

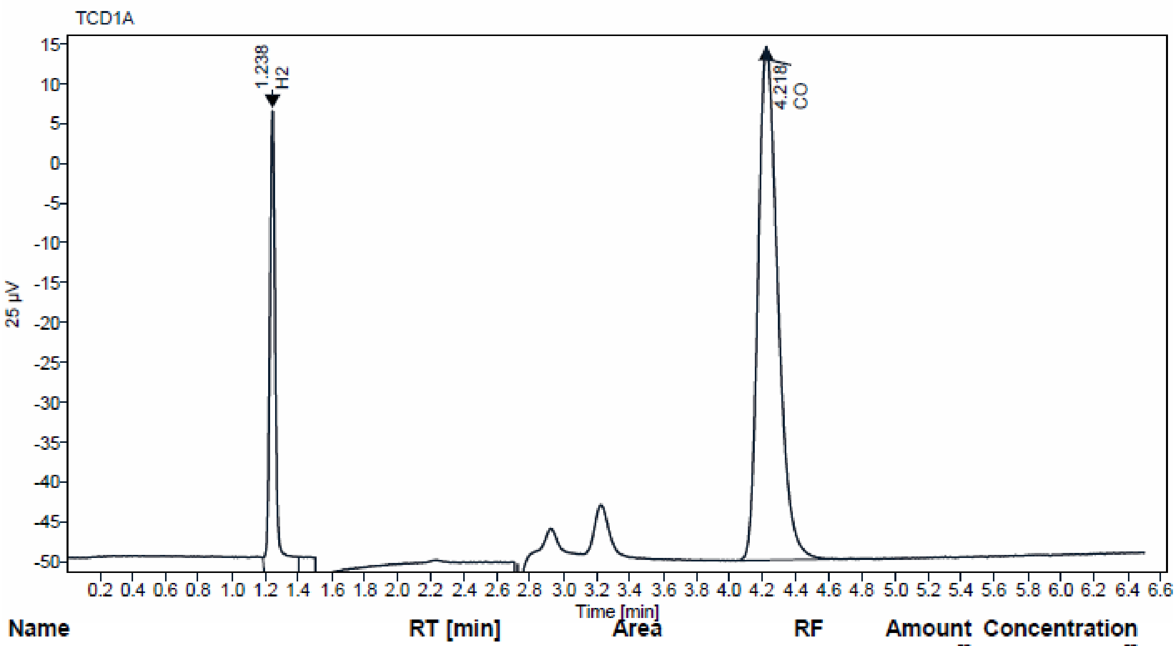

Name	RT [min]	Area	RF	Amount []	Concentration []
H2	1.238	472.8507	473.14215	0.999	0.9994
CO	4.218	533.0951	65.11310	8.187	8.1872

Figure S178: GC-TCD data for decarbonylation of 4.6 M N-formylmorpholine under standard conditions.

5.4. GC-TCD data acquired from the sequential formylation and decarbonylation of morpholine.

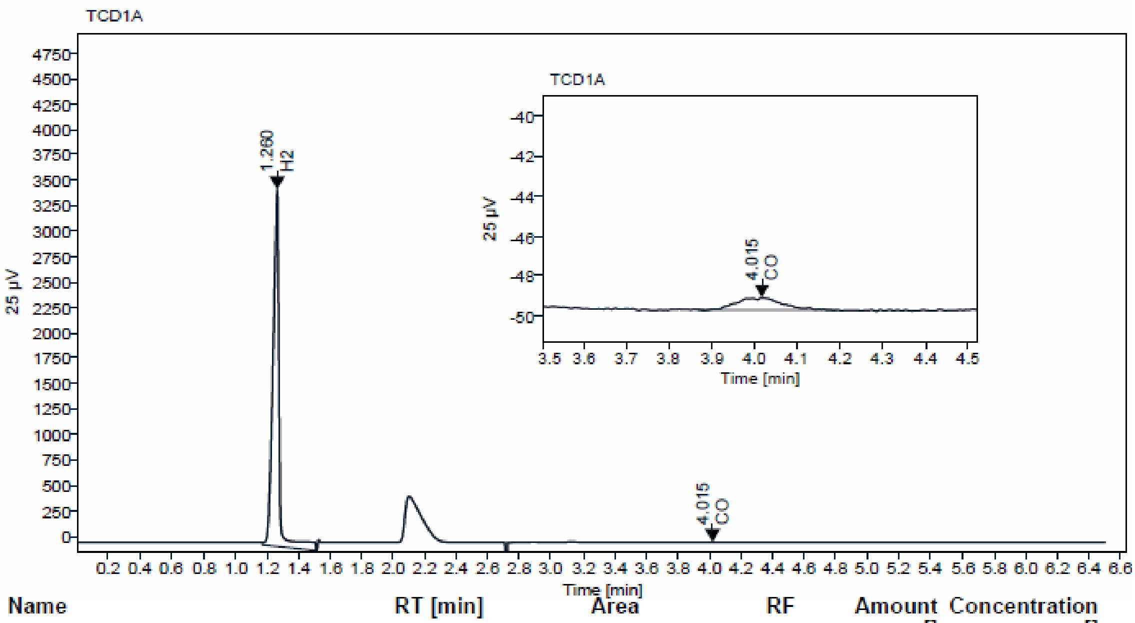

Name	RT [min]	Area	RF	Amount []	Concentration []
H2	1.260	9661.4162	881.68571	10.958	10.9579
CO	4.015	4.4730	35.48219	0.126	0.1261

Figure S179: GC-TCD data for sequential formylation and decarbonylation of morpholine (no drying stage).

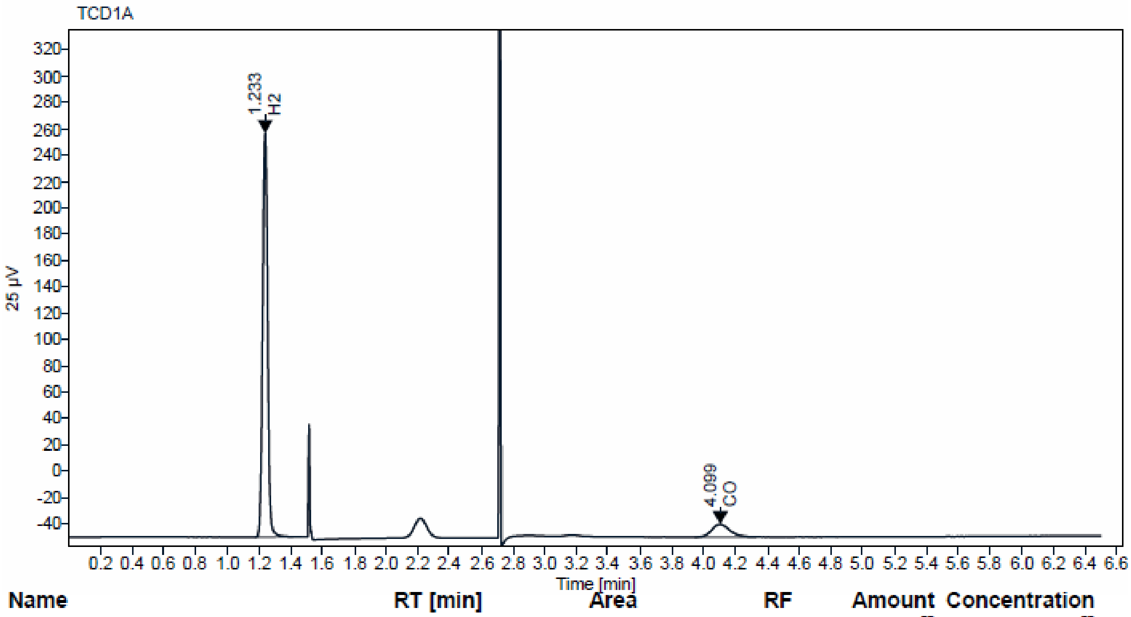

Name	RT [min]	Area	RF	Amount []	Concentration []
H2	1.233	660.5279	455.26827	1.451	1.4509
CO	4.099	75.5701	38.67660	1.954	1.9539

Figure S180: GC-TCD data for sequential formylation and decarbonylation of morpholine (with drying stage).

5.5. GC-TCD data acquired from the one-pot reverse water gas shift reaction

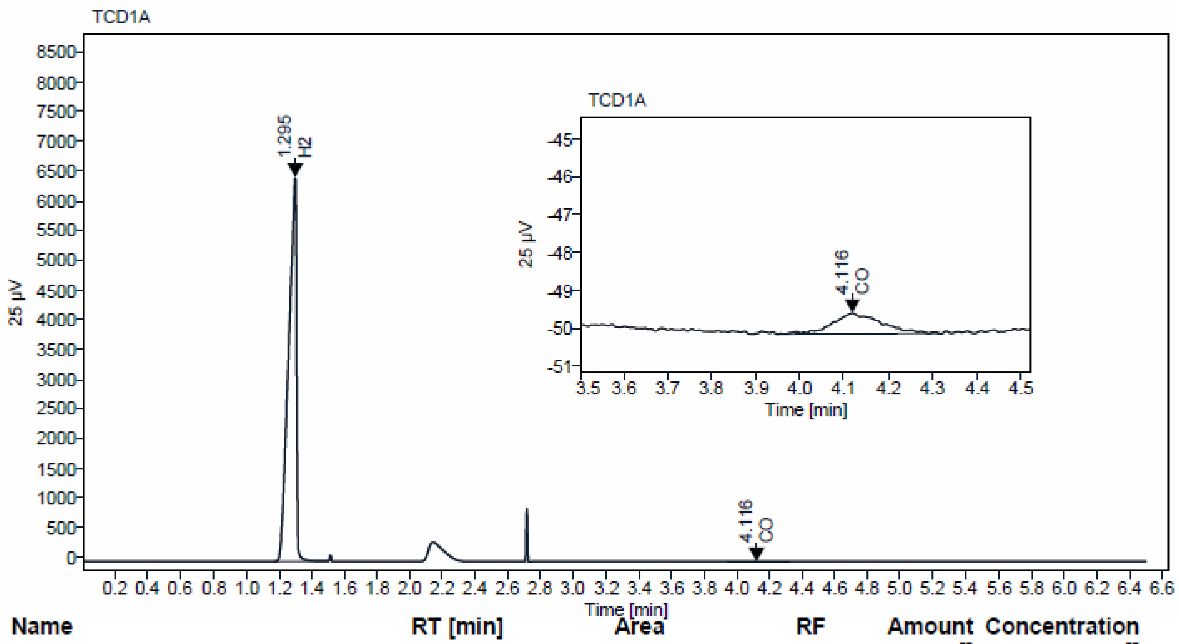

Name	RT [min]	Area	RF	Amount []	Concentration []
H2	1.295	21432.0758	1178.32333	18.189	18.1886
CO	4.116	4.1992	35.45417	0.118	0.1184

Figure S181: GC-TCD data corresponding to Table S4, entry 1.

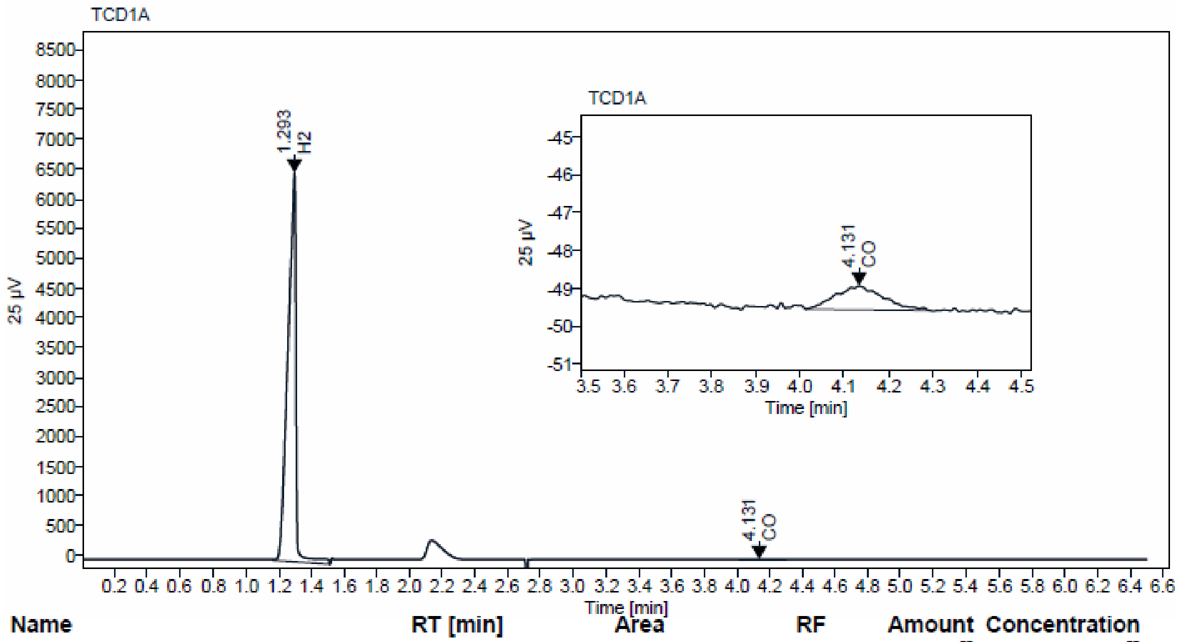

Name	RT [min]	Area	RF	Amount []	Concentration []
H2	1.293	22327.6937	1197.22945	18.649	18.6495
CO	4.131	4.7424	35.50972	0.134	0.1336

Figure S182: GC-TCD data corresponding to Table S4, entry 2.

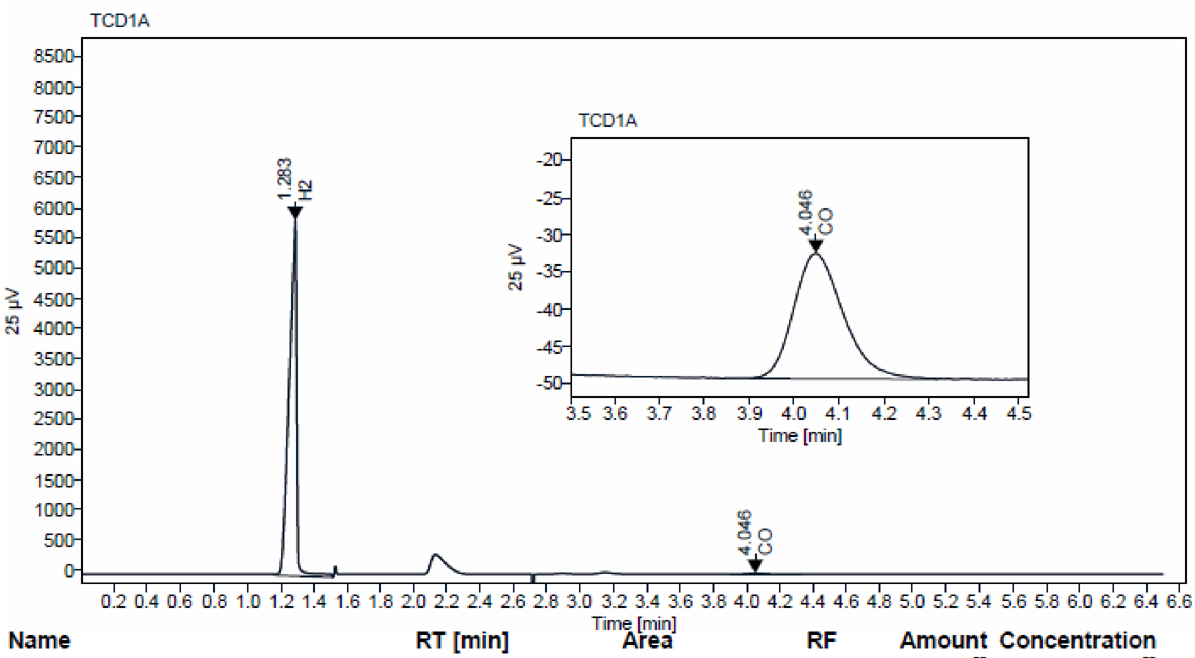

Name	RT [min]	Area	RF	Amount []	Concentration []
H2	1.283	18555.6699	455.26827	40.758	40.7577
CO	4.046	129.6313	38.67660	3.352	3.3517

Figure S183: GC-TCD data corresponding to Table S4, entry 3.

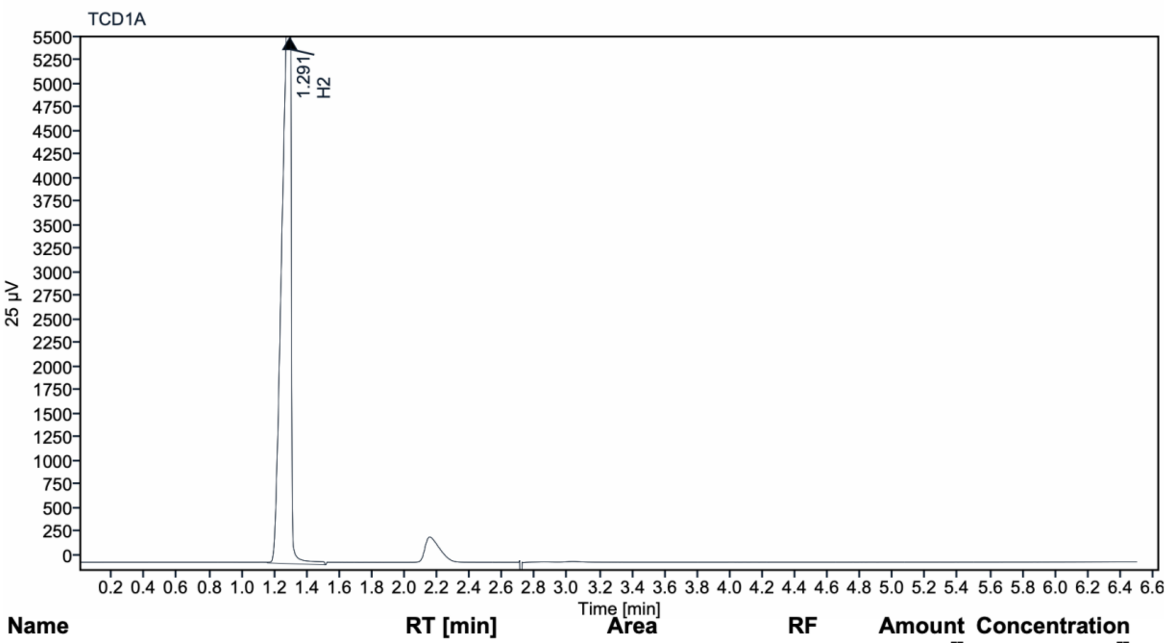

Name	RT [min]	Area	RF	Amount []	Concentration []
H2	1.291	25084.7671	1253.26901	20.015	20.0155

Figure S184: GC-TCD data corresponding to Table S4, entry 4.

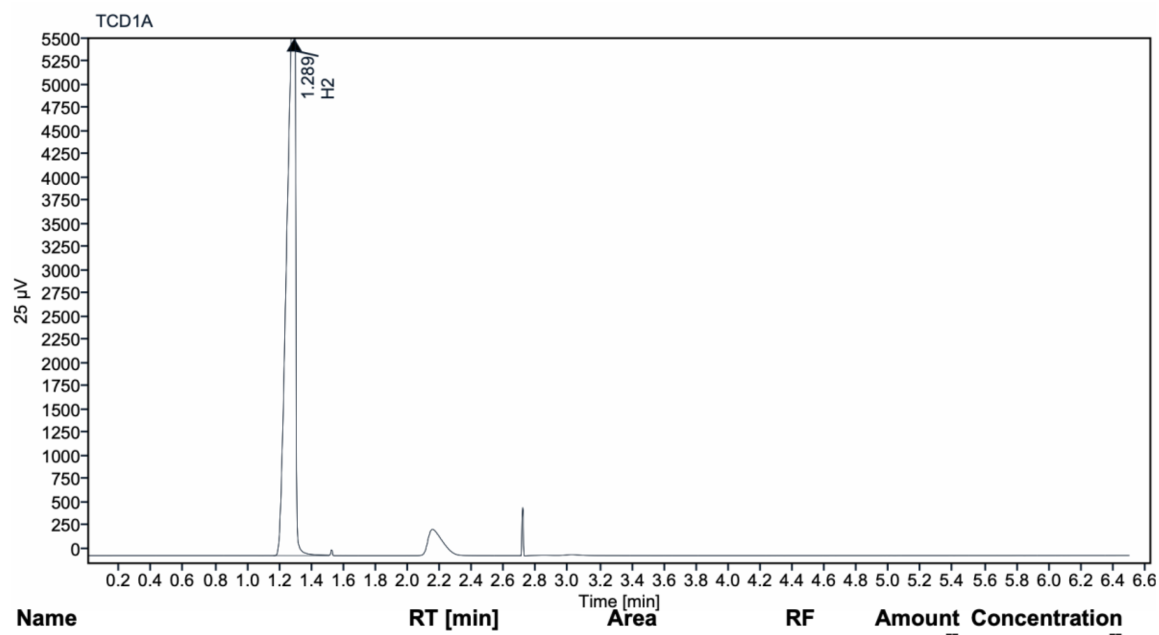

Name	RT [min]	Area	RF	Amount []	Concentration []
H2	1.289	23821.9737	1227.98707	19.399	19.3992

Figure S185: GC-TCD data corresponding to Table S4, entry 5.

6. Computation Details

6.1. General Considerations

DFT calculations were performed using the PBE0-D3(BJ)$_{PCM(THF)}$/def2-TZVP//RIBP86$_{PCM(THF)}$/def2-SVP level of theory with Gaussian16, C.01.[11] This is the same level as used in previous work from the group,[12-16] benchmarked against a series of experimental 3d transition metal hydride bond strengths[17] and including Martin, Hay and Pratt empirical entropy corrections[18] (i.e. 5.09 kcal mol^{-1} per particle[14]). As noted previously, the choice of solvent between THF (consistent with previous work[12]) and toluene (used experimentally) has little impact upon the overall thermodynamics and for continuity we retain THF. Ru was described using the Stuttgart-Dresden relativistic small-core pseudopotential[19] in conjunction with the def2-SVP and def2-TZVP valence basis sets. The stereochemistry and conformation of the metal fragment was taken as that observed in the X-ray structures, notably with the CO ligand of the [Ru](H)(CO) moiety trans to the MACHO-N atom, and the hydride trans to the bound substrates.

Not all of the stationary points for the *mer* isomers in Figure 4C in the main paper could be located for the corresponding *fac* variants. Notably, *fac*-**1C'** does not exist, an attempted optimisation affords *fac*-**1C** instead. Perhaps related to this observation, a *fac* analogue of **TSC-D** could not be found. Also, *fac*-**TSD-A** could not be fully optimised. The value in Figure 4C corresponds to a partially optimised structure with low gradients and the correct mode for the

single imaginary frequency. A number of additional candidates for intermediates with *fac* conformation have been trialled (see Figure S185 below), without evidence that one of them could be competitive to *fac*-**1C**.

6.2. Possible *fac* intermediates of **1** + N-formylmorpholine

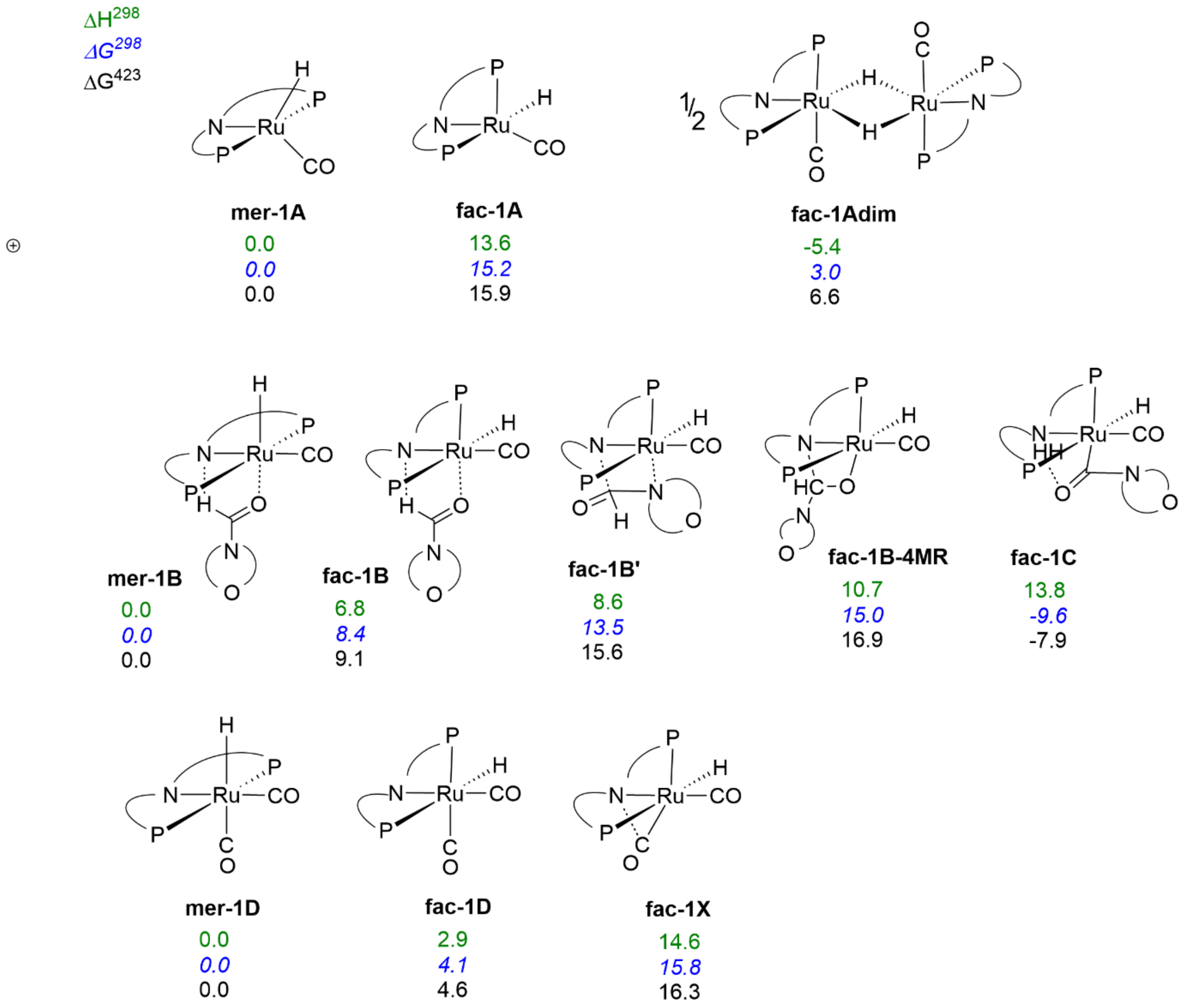

*Figure S186: Potential intermediates with fac conformations that could arise from the reaction of N-formylmorpholine and **1**, along with corresponding ΔH^{298}, ΔG^{298} and ΔG^{423} values (in kcal mol^{-1}).*

6.3. Computational raw data

CO

Frequencies, energies and thermodynamic properties:

Lowest Vibrational Mode (cm^{-1}) = 2149.2061

2nd Lowest Vibrational Mode (cm^{-1}) =

E(RB-P86) (h) = -113.22512

Thermal correction to Enthalpy (h) = 0.00820

Thermal correction to Gibbs Free Energy (h) = -0.01425

Total Entropy (cal/Kmol) = 47.251

E(RPBE1PBE) (h) = -113.231032

Optimised cartesian coordinates (Å):

C	0.0000000	0.0000000	-0.6526720
O	0.0000000	0.0000000	0.4895040

H_2O

Frequencies, energies and thermodynamic properties:

Lowest Vibrational Mode (cm^{-1}) = 1609.3992

2nd Lowest Vibrational Mode (cm^{-1}) = 3673.6140

E(RB-P86) (h) = -76.36500

Thermal correction to Enthalpy (h) = 0.02438

Thermal correction to Gibbs Free Energy (h) = 0.00290

Total Entropy (cal/Kmol) = 45.202

E(RPBE1PBE) (h) = -76.38313

Optimised cartesian coordinates (Å):

O	0.0000000	0.0000000	0.1233180
H	0.0000000	0.7574680	-0.4932730
H	0.0000000	-0.7574680	-0.4932730

CO_2

Frequencies, energies and thermodynamic properties:

Lowest Vibrational Mode (cm^{-1}) = 622.8599

2nd Lowest Vibrational Mode (cm^{-1}) =

E(RB-P86) (h) = -188.45623

Thermal correction to Enthalpy (h) = 0.01493

Thermal correction to Gibbs Free Energy (h) = -0.01007

Total Entropy (cal/Kmol) = 52.619

E(RPBE1PBE) (h) = -188.46784

Optimised cartesian coordinates (Å):

C	0.0000000	0.0000000	-0.0001190
O	0.0000000	0.0000000	1.1750910
O	0.0000000	0.0000000	-1.1750020

H_2

Frequencies, energies and thermodynamic properties:

Lowest Vibrational Mode (cm^{-1}) = 4271.1311

2nd Lowest Vibrational Mode (cm^{-1}) =

E(RB-P86) (h) = -1.17254

Thermal correction to Enthalpy (h) = 0.01303

Thermal correction to Gibbs Free Energy (h) = -0.00182

Total Entropy (cal/Kmol) = 31.263

E(RPBE1PBE) (h) = -1.16812

Optimised cartesian coordinates (Å):

H	0.0000000	0.0000000	0.3838560
H	0.0000000	0.0000000	-0.3838560

DMF

Frequencies, energies and thermodynamic properties:

Lowest Vibrational Mode (cm^{-1}) = 146.6401

2nd Lowest Vibrational Mode (cm^{-1}) = 180.3087

E(RB-P86) (h) = -248.33772

Thermal correction to Enthalpy (h) = 0.10602

Thermal correction to Gibbs Free Energy (h) = 0.07001

Total Entropy (cal/Kmol) = 75.78400

E(RPBE1PBE) (h) = -248.33175

Optimised cartesian coordinates (Å):

H	-0.7410370	-1.7718940	-0.0000780
C	-0.8625170	-0.6519300	0.0000070
N	0.3498670	-0.0173450	0.0002180
O	-1.9648600	-0.1015010	-0.0000400
C	0.4170060	1.4365870	-0.0000400
H	0.9516930	1.8096090	0.9007310
H	0.9524290	1.8091730	-0.9005520
H	-0.6184470	1.8257930	-0.0004800
C	1.5993590	-0.7596180	-0.0000450
H	2.2086700	-0.5210510	0.8995670
H	1.3853680	-1.8470050	0.0002650
H	2.2080450	-0.5214380	-0.9001930

Me_2NH

Frequencies, energies and thermodynamic properties:

Lowest Vibrational Mode (cm^{-1}) = 240.2612

2nd Lowest Vibrational Mode (cm^{-1}) = 264.2018

E(RB-P86) (h) = -135.06078

Thermal correction to Enthalpy (h) = 0.09450

Thermal correction to Gibbs Free Energy (h) = 0.06362

Total Entropy (cal/Kmol) = 65.002

E(RPBE1PBE) (h) = -135.05178

Optimised cartesian coordinates (Å):

C	-1.2166150	-0.2200520	0.0201380
H	-2.1085430	0.4357200	-0.0591770
H	-1.2935180	-0.9826860	-0.7863880
H	-1.2849060	-0.7741010	0.9957300
N	0.0000590	0.5636350	-0.1524220
H	0.0000600	1.3373140	0.5249940
C	1.2164790	-0.2199940	0.0200960
H	1.2939120	-0.9824650	-0.7864750
H	2.1085820	0.4355090	-0.0586040
H	1.2848110	-0.7744570	0.9954680

N-Methylformanilide

Frequencies, energies and thermodynamic properties:

Lowest Vibrational Mode (cm^{-1}) = 65.3293

2nd Lowest Vibrational Mode (cm^{-1}) = 114.7150

E(RB-P86) (h) = -439.94062

Thermal correction to Enthalpy (h) = 0.16073

Thermal correction to Gibbs Free Energy (h) = 0.11654

Total Entropy (cal/Kmol) = 93.001

E(RPBE1PBE) (h) = -439.91813

Optimised cartesian coordinates (Å):

H	-1.5298000	-1.6934790	-0.7379010
C	-2.0977840	-0.8139810	-0.3366790
N	-1.2608320	0.2040420	0.0833190
O	-3.3251720	-0.7724880	-0.2989530
C	-1.8645100	1.4699250	0.5041530
H	-1.3658580	1.8464310	1.4186740
H	-1.7967670	2.2443290	-0.2893440

H	-2.9333860	1.2731020	0.7073100
C	0.1555050	0.0667730	0.0388480
C	0.9709420	1.1832730	-0.2612860
C	0.7718180	-1.1785090	0.3139480
C	2.3681130	1.0470420	-0.3089360
H	0.5135730	2.1609520	-0.4713240
C	2.1685150	-1.3067420	0.2438210
H	0.1619900	-2.0432750	0.6145600
C	2.9757680	-0.1978300	-0.0673130
H	2.9863140	1.9276370	-0.5456220
H	2.6296600	-2.2834260	0.4606410
H	4.0712690	-0.3003610	-0.1079310

N-methylaniline

Frequencies, energies and thermodynamic properties:

Lowest Vibrational Mode (cm^{-1}) = 80.6377

2nd Lowest Vibrational Mode (cm^{-1}) = 158.6596

E(RB-P86) (h) = -326.67849

Thermal correction to Enthalpy (h) = 0.14914

Thermal correction to Gibbs Free Energy (h) = 0.10871

Total Entropy (cal/Kmol) = 85.080

E(RPBE1PBE) (h) = -326.64978

Optimised cartesian coordinates (Å):

C	-2.8634230	-0.3617620	0.0450930
H	-3.8347760	0.1663330	0.0006240
H	-2.8445380	-1.0966130	-0.7909430
H	-2.8311270	-0.9416930	0.9990200
N	-1.7936010	0.6056620	-0.0678880

H	-2.0318300	1.5859760	0.0824890
C	-0.4532840	0.2814290	-0.0256690
C	-0.0036720	-1.0697280	-0.0216860
C	0.5322280	1.3117760	-0.0011010
C	1.3706400	-1.3630980	-0.0048960
H	-0.7326150	-1.8934670	-0.0328530
C	1.8971960	1.0003420	0.0184720
H	0.2066300	2.3656980	-0.0001080
C	2.3355570	-0.3402630	0.0152780
H	1.6888130	-2.4188940	-0.0037900
H	2.6332350	1.8210670	0.0372810
H	3.4099570	-0.5802120	0.0305500

N-formylpiperidine

Frequencies, energies and thermodynamic properties:

Lowest Vibrational Mode (cm^{-1}) = 74.2345

2nd Lowest Vibrational Mode (cm^{-1}) = 188.3409

E(RB-P86) (h) = -364.983206385

Thermal correction to Enthalpy (h) = 0.171850

Thermal correction to Gibbs Free Energy (h) = 0.131592

Total Entropy (cal/Kmol) = 84.729

E(RPBE1PBE) (h) = -364.982750546

Optimised cartesian coordinates (Å):

N	-0.657942	0.262849	0.263500
C	-0.174953	-1.090796	0.548866
H	-1.007608	-1.784745	0.320942
H	0.049560	-1.170433	1.638810
C	1.088864	-1.405288	-0.268869

H	1.481834	-2.399326	0.033341
H	0.813976	-1.479862	-1.344655
C	1.581896	1.078841	-0.399613
H	1.329983	1.142876	-1.481764
H	2.328793	1.874356	-0.191889
C	0.310852	1.347345	0.424008
H	0.577628	1.440880	1.503716
H	-0.176811	2.297847	0.122515
C	-1.936484	0.480562	-0.167536
O	-2.799139	-0.384052	-0.334196
H	-2.125834	1.574937	-0.359667
C	2.157462	-0.313054	-0.081304
H	3.042904	-0.521467	-0.718607
H	2.518456	-0.328256	0.973029

Piperidine

Frequencies, energies and thermodynamic properties:

Lowest Vibrational Mode (cm^{-1}) = 242.6210

2nd Lowest Vibrational Mode (cm^{-1}) = 244.5524

E(RB-P86) (h) = -251.563366870

Thermal correction to Enthalpy (h) = 0.160277

Thermal correction to Gibbs Free Energy (h) = 0.124922

Total Entropy (cal/Kmol) = 74.412

E(RPBE1PBE) (h) = -251.702529388

Optimised cartesian coordinates (Å):

N	-1.386212	-0.001926	-0.317944
H	-2.387132	-0.003329	-0.086199
C	-0.754087	-1.217726	0.203602

H	-1.301527	-2.102565	-0.186902
H	-0.799474	-1.280179	1.327396
C	0.720302	-1.266581	-0.225521
H	1.204757	-2.178289	0.186301
H	0.765415	-1.344744	-1.335120
C	0.716762	1.268597	-0.225549
H	0.761600	1.346822	-1.335151
H	1.198665	2.181654	0.186245
C	-0.757498	1.215600	0.203617
H	-0.802997	1.277922	1.327439
H	-1.307406	2.098958	-0.186810
C	1.463194	0.002044	0.231876
H	1.530466	0.002156	1.344914
H	2.509074	0.003483	-0.144650

N-Formylmorpholine

Frequencies, energies and thermodynamic properties:

Lowest Vibrational Mode (cm^{-1}) = 77.3752

2nd Lowest Vibrational Mode (cm^{-1}) = 200.6285

E(RB-P86) (h) = -400.86009

Thermal correction to Enthalpy (h) = 0.14831

Thermal correction to Gibbs Free Energy (h) = 0.10853

Total Entropy (cal/Kmol) = 83.716

E(RPBE1PBE) (h) = -400.86241

Optimised cartesian coordinates (Å):

H	2.1164570	-1.5834930	-0.3382350
C	1.9260160	-0.4882500	-0.1554660
O	2.7863740	0.3779820	-0.3166300

C	-0.3468890	-1.3311390	0.4070300
C	0.1491580	1.0848580	0.5318690
C	-1.6102430	-0.9737920	-0.3896440
H	-0.6117850	-1.4535110	1.4827470
H	0.0895700	-2.2866050	0.0485380
C	-1.1386270	1.3294760	-0.2655020
H	-0.0583900	1.1930390	1.6211790
H	0.9489240	1.7987510	0.2517150
H	-2.4177900	-1.7058370	-0.1853730
H	-1.3775080	-0.9984740	-1.4837980
H	-1.6016020	2.2921240	0.0319930
H	-0.8985840	1.3759910	-1.3569790
N	0.6421480	-0.2657250	0.2611550
O	-2.1064760	0.3096650	-0.0170690

Morpholine

Frequencies, energies and thermodynamic properties:

Lowest Vibrational Mode (cm^{-1}) = 259.2210

2nd Lowest Vibrational Mode (cm^{-1}) = 262.5770

E(RB-P86) (h) = -287.58530

Thermal correction to Enthalpy (h) = 0.13688

Thermal correction to Gibbs Free Energy (h) = 0.10202

Total Entropy (cal/Kmol) = 73.368

E(RPBE1PBE) (h) = -287.58443

Optimised cartesian coordinates (Å):

C	-1.1771610	-0.7579590	0.1963110
C	-1.2052790	0.7217410	-0.1976570
C	1.2052790	0.7217410	-0.1976570

C	1.1771610	-0.7579590	0.1963110
H	-1.2894350	0.7849960	-1.3164350
H	-2.1088190	1.2048530	0.2326310
H	-1.2268580	-0.8406550	1.3116630
H	-2.0429050	-1.3009100	-0.2358400
H	1.2894350	0.7849960	-1.3164350
H	2.1088190	1.2048530	0.2326310
H	2.0429050	-1.3009100	-0.2358400
H	1.2268580	-0.8406550	1.3116630
O	0.0000000	-1.4031850	-0.2914490
N	0.0000000	1.3706370	0.3256640
H	0.0000000	2.3690670	0.0840590

N-methyl piperazine

Frequencies, energies and thermodynamic properties:

Lowest Vibrational Mode (cm-1) = 160.0826

2nd Lowest Vibrational Mode (cm-1) = 233.8035

E(RB-P86) (h) = -307.0148454

Thermal correction to Enthalpy (h) = 0.177084

Thermal correction to Gibbs Free Energy (h) = 0.138871

Total Entropy (cal/Kmol) = 80.425

E(RPBE1PBE) (h) = -307.0012302

Optimised cartesian coordinates (Å):

N	-1.817879	-0.000037	0.328728
H	-2.819634	-0.000078	0.100886
C	-1.178976	1.204195	-0.203753
H	-1.675877	2.104213	0.216721
H	-1.242765	1.275227	-1.323440

C	0.302177	1.206851	0.188485
H	0.805847	2.098219	-0.244489
H	0.379618	1.291395	1.308948
N	0.968663	0.000008	-0.304538
C	0.302337	-1.206885	0.188488
H	0.379767	-1.291541	1.308953
H	0.806049	-2.098188	-0.244606
C	-1.178815	-1.204230	-0.203784
H	-1.242785	-1.275351	-1.323442
H	-1.675540	-2.104267	0.216850
C	2.395097	0.000104	-0.023476
H	2.872785	0.897822	-0.469953
H	2.633662	0.001507	1.076032
H	2.872468	-0.898966	-0.467544

1-Formyl-4-methylpiperazine

Frequencies, energies and thermodynamic properties:

Lowest Vibrational Mode (cm-1) = 58.3084

2nd Lowest Vibrational Mode (cm-1) = 169.0934

E(RB-P86) (h) = -420.2901341

Thermal correction to Enthalpy (h) = 0.188521

Thermal correction to Gibbs Free Energy (h) = 0.14524

Total Entropy (cal/Kmol) = 91.093

E(RPBE1PBE) (h) = -420.2802604

Optimised cartesian coordinates (Å):

N	-1.114713	0.256627	0.261968
C	-0.530414	-1.030432	0.632942
H	-1.249670	-1.819841	0.340484

H	-0.390154	-1.066185	1.737337
C	0.824811	-1.215574	-0.061376
H	1.305147	-2.143326	0.315845
H	0.655793	-1.356082	-1.165391
N	1.707336	-0.078838	0.209489
C	1.106683	1.172952	-0.252236
H	0.948505	1.174426	-1.367168
H	1.793963	2.013317	-0.017115
C	-0.240931	1.411514	0.442216
H	-0.067479	1.580842	1.529798
H	-0.742051	2.310554	0.028341
C	3.048663	-0.279023	-0.321285
H	3.490798	-1.204408	0.103316
H	3.074941	-0.371754	-1.440902
H	3.700730	0.573705	-0.038735
C	-2.368085	0.346252	-0.276726
O	-3.134978	-0.597771	-0.472400
H	-2.633421	1.412266	-0.528013

1A

Frequencies, energies and thermodynamic properties:

Lowest Vibrational Mode (cm^{-1}) = 13.9404

2nd Lowest Vibrational Mode (cm^{-1}) = 15.2344

E(RB-P86) (h) = -2029.32435

Thermal correction to Enthalpy (h) = 0.52193

Thermal correction to Gibbs Free Energy (h) = 0.41854

Total Entropy (cal/Kmol) = 217.596

E(RPBE1PBE) (h) = -2029.06886

Optimised cartesian coordinates (Å):

Ru	0.0000120	-0.2277750	0.1874670
P	-2.3101820	-0.0390720	0.4493600
P	2.3101950	-0.0390330	0.4493580
O	-0.0000060	-1.9067770	-2.3436640
N	0.0000080	0.2147030	2.1681740
C	-1.2092670	0.6193490	2.8963700
H	-1.3327650	1.7335050	2.8876320
H	-1.1182240	0.3390920	3.9753830
C	1.2092620	0.6194110	2.8963710
H	1.1182380	0.3391380	3.9753810
H	1.3326880	1.7335760	2.8876460
C	2.4581580	-0.0451040	2.3110160
H	2.4774870	-1.1224940	2.5805110
H	3.4016610	0.4055730	2.6813790
C	-2.4581230	-0.0452440	2.3110250
H	-3.4016450	0.4053610	2.6814230
H	-2.4773690	-1.1226440	2.5804870
C	0.0000030	-1.2310670	-1.3727140
H	0.0000380	0.9989390	-0.8107550
C	-3.4875650	-1.3717200	-0.0806470
C	-4.8252690	-1.1096490	-0.4503350
C	-3.0220160	-2.7067270	-0.0794080
C	-5.6833800	-2.1669320	-0.8035400
H	-5.2010240	-0.0745210	-0.4655000
C	-3.8835650	-3.7612580	-0.4263740
H	-1.9726410	-2.9147770	0.1883310
C	-5.2163580	-3.4934550	-0.7899320

H	-6.7244000	-1.9503290	-1.0921900
H	-3.5088570	-4.7972620	-0.4211940
H	-5.8899250	-4.3194790	-1.0685540
C	-3.1142240	1.5161850	-0.1591630
C	-3.9666950	2.3141550	0.6356950
C	-2.8523810	1.9132520	-1.4918660
C	-4.5449060	3.4842950	0.1079260
H	-4.1902580	2.0315770	1.6757700
C	-3.4417180	3.0729320	-2.0208660
H	-2.1719830	1.3098390	-2.1139990
C	-4.2873970	3.8640280	-1.2204690
H	-5.2022230	4.1001260	0.7422750
H	-3.2320310	3.3658350	-3.0619580
H	-4.7423160	4.7789390	-1.6322050
C	3.4876720	-1.3716200	-0.0805810
C	4.8255680	-1.1095080	-0.4495550
C	3.0220520	-2.7065990	-0.0799410
C	5.6837910	-2.1667300	-0.8026590
H	5.2013790	-0.0743920	-0.4642490
C	3.8837110	-3.7610740	-0.4268190
H	1.9725460	-2.9146630	0.1872660
C	5.2166870	-3.4932340	-0.7896650
H	6.7249620	-1.9501010	-1.0907480
H	3.5089450	-4.7970590	-0.4221220
H	5.8903430	-4.3192110	-1.0682130
C	3.1141260	1.5162280	-0.1592880
C	3.9658430	2.3148130	0.6357550
C	2.8528800	1.9127080	-1.4922870

C	4.5439100	3.4849780	0.1078790
H	4.1889180	2.0327070	1.6760630
C	3.4420800	3.0724100	-2.0213830
H	2.1730630	1.3088200	-2.1145960
C	4.2870100	3.8641230	-1.2207990
H	5.2006360	4.1012920	0.7423720
H	3.2328730	3.3648480	-3.0627020
H	4.7418170	4.7790510	-1.6326180

1D

Frequencies, energies and thermodynamic properties:

Lowest Vibrational Mode (cm^{-1}) = 9.1275

2nd Lowest Vibrational Mode (cm^{-1}) = 15.1636

E(RB-P86) (h) = -2142.58916

Thermal correction to Enthalpy (h) = 0.53156

Thermal correction to Gibbs Free Energy (h) = 0.42394

Total Entropy (cal/Kmol) = 226.502

E(RPBE1PBE) (h) = -2142.34231

Optimised cartesian coordinates (Å):

Ru	-0.0000060	-0.2906000	0.2017240
P	-2.3222640	0.0004460	0.4893310
P	2.3222640	0.0003420	0.4893060
O	-0.0000630	-0.2196970	-2.8501700
N	0.0000100	-0.0164580	2.3689300
C	-1.1797160	0.6497790	2.8888990
H	-1.1841760	1.7584440	2.6626720
H	-1.2026810	0.5842670	4.0087000
C	1.1798240	0.6496030	2.8889300

H	1.2027920	0.5840210	4.0087270
H	1.1844140	1.7582800	2.6627720
C	2.4699310	0.0314320	2.3413990
H	2.5481760	-1.0335760	2.6478360
H	3.3842070	0.5553080	2.6880250
C	-2.4699010	0.0317200	2.3414290
H	-3.3841040	0.5557600	2.6879980
H	-2.5482920	-1.0332490	2.6479670
C	-0.0000530	-0.2981230	-1.6795550
H	0.0000050	1.3717770	0.1765770
C	-3.5403270	-1.2698140	-0.0864330
C	-4.8877870	-1.2021820	0.3406970
C	-3.1431780	-2.3108790	-0.9512680
C	-5.8139040	-2.1700820	-0.0808530
H	-5.2207530	-0.3871430	1.0035810
C	-4.0755100	-3.2746120	-1.3791390
H	-2.0985060	-2.3653160	-1.2944370
C	-5.4093580	-3.2080320	-0.9421930
H	-6.8591050	-2.1118660	0.2622570
H	-3.7540660	-4.0811950	-2.0568960
H	-6.1382180	-3.9642730	-1.2743820
C	-3.0757210	1.5631970	-0.1552040
C	-3.3849150	2.6593640	0.6789890
C	-3.3085080	1.6716040	-1.5461740
C	-3.9193860	3.8405700	0.1310060
H	-3.2069220	2.6060680	1.7637100
C	-3.8431710	2.8510510	-2.0891450
H	-3.0729670	0.8269840	-2.2137680

C	-4.1500850	3.9400000	-1.2515380
H	-4.1562970	4.6875640	0.7943940
H	-4.0209220	2.9197720	-3.1741290
H	-4.5685410	4.8654100	-1.6779370
C	3.5402670	-1.2699020	-0.0866130
C	4.8877620	-1.2023030	0.3404100
C	3.1430280	-2.3109410	-0.9514410
C	5.8138360	-2.1701910	-0.0812620
H	5.2207910	-0.3872930	1.0032980
C	4.0753170	-3.2746640	-1.3794300
H	2.0983190	-2.3653760	-1.2944980
C	5.4092060	-3.2081050	-0.9426050
H	6.8590670	-2.1119950	0.2617590
H	3.7538040	-4.0812260	-2.0571780
H	6.1380300	-3.9643410	-1.2748840
C	3.0757550	1.5631340	-0.1550970
C	3.3855340	2.6590150	0.6792540
C	3.3080230	1.6718420	-1.5461320
C	3.9200530	3.8402410	0.1313600
H	3.2079680	2.6054830	1.7640320
C	3.8427430	2.8513030	-2.0890130
H	3.0720110	0.8274510	-2.2138500
C	4.1502310	3.9399710	-1.2512480
H	4.1574150	4.6870130	0.7948700
H	4.0200830	2.9202620	-3.1740490
H	4.5687300	4.8653950	-1.6775760
C	0.0000570	-2.2037300	0.5197750
O	0.0000160	-3.3358300	0.7976260

TSD-A

Frequencies, energies and thermodynamic properties:

Lowest Vibrational Mode (cm^{-1}) = -27.6167

2nd Lowest Vibrational Mode (cm^{-1}) = 16.0632

E(RB-P86) (h) = -2142.54863

Thermal correction to Enthalpy (h) = 0.53061

Thermal correction to Gibbs Free Energy (h) = 0.41997

Total Entropy (cal/Kmol) = 232.848

E(RPBE1PBE) (h) = -2142.30558

Optimised cartesian coordinates (Å):

Ru	0.0362570	-0.1855490	0.1710630
P	-2.2693830	0.1279310	0.4090800
P	2.3475040	0.0253250	0.4339450
N	0.0362960	0.4346970	2.1129590
O	0.0389990	-1.9122210	-2.3303160
C	-3.0073330	1.7489730	-0.1336690
C	1.2449750	0.8783430	2.8129470
H	1.1445370	0.6844250	3.9100810
H	1.3873570	1.9875700	2.7207900
C	-2.1602070	2.8526460	-0.3759710
H	-1.0684880	2.7164560	-0.3058580
C	3.5843460	-1.2798100	-0.0208650
C	-3.5181450	-1.1027350	-0.1735620
C	3.7785480	2.5273060	0.5136000
H	3.9057900	2.3794980	1.5967020
C	-4.4195200	-1.7572060	0.6957550
H	-4.4180530	-1.5372980	1.7741570

C	3.1461970	-2.6157310	-0.1540230
H	2.0771900	-2.8496700	-0.0305820
C	2.4880400	0.1528560	2.2883190
H	2.4897040	-0.9020060	2.6359710
H	3.4375810	0.6163420	2.6270090
C	-2.4209210	0.1905140	2.2674050
H	-3.3606190	0.6795870	2.5978050
H	-2.4216030	-0.8636330	2.6163430
C	3.0959720	1.5695730	-0.2680430
C	-1.1723780	0.9056580	2.7957210
H	-1.2993320	2.0157890	2.6898470
H	-1.0870950	0.7224270	3.8957360
C	4.0631680	-3.6392580	-0.4513840
H	3.7084420	-4.6769830	-0.5556100
C	4.9545480	-0.9823660	-0.1997010
H	5.3104910	0.0561880	-0.1089210
C	-4.9449900	3.1710610	-0.5901520
H	-6.0368440	3.2894700	-0.6786350
C	-4.4067220	1.9194610	-0.2440880
H	-5.0827890	1.0683270	-0.0640920
C	0.0344060	-1.2273950	-1.3671150
C	-3.5527820	-1.4091120	-1.5548200
H	-2.8505500	-0.9148360	-2.2450840
C	-2.7017470	4.1055080	-0.7163810
H	-2.0291410	4.9577960	-0.9035870
C	4.3080840	3.6881840	-0.0826280
H	4.8329810	4.4287370	0.5416880
C	5.8688200	-2.0074150	-0.4993970

H	6.9344400	-1.7643650	-0.6382480
C	2.9536670	1.7972560	-1.6578420
H	2.4082250	1.0672630	-2.2775730
C	-4.0942590	4.2671310	-0.8257070
H	-4.5181030	5.2466030	-1.0991580
C	5.4257890	-3.3370330	-0.6250690
H	6.1434150	-4.1378770	-0.8644420
C	-5.3369280	-2.6985230	0.1917930
H	-6.0334350	-3.2017030	0.8812960
C	4.1707930	3.8998660	-1.4646730
H	4.5876860	4.8073430	-1.9295940
C	-4.4739180	-2.3434500	-2.0549060
H	-4.4895230	-2.5710190	-3.1325840
C	-5.3675990	-2.9926100	-1.1820810
H	-6.0863930	-3.7295260	-1.5741020
C	3.4944680	2.9483500	-2.2522350
H	3.3804360	3.1080480	-3.3362590
H	0.0977460	1.0009080	-0.8658010
O	-0.8074800	-3.9817660	0.2058360
C	-0.6906070	-3.1816370	1.0213880

1B

Frequencies, energies and thermodynamic properties:

Lowest Vibrational Mode (cm^{-1}) = 13.0165

2nd Lowest Vibrational Mode (cm^{-1}) = 17.3196

E(RB-P86) (h) = -2430.18932

Thermal correction to Enthalpy (h) = 0.67226

Thermal correction to Gibbs Free Energy (h) = 0.54891

Total Entropy (cal/Kmol) = 259.598

E(RPBE1PBE) (h) = -2429.94298

Optimised cartesian coordinates (Å):

Ru	0.2828370	-0.4429760	-0.0549190
P	2.4445750	0.3443890	0.3122060
P	-1.7582400	-1.5169560	0.2750700
N	0.1222920	-0.1213290	2.0524040
O	0.5410580	-0.9526810	-3.0352370
C	3.8062570	-0.9147010	0.2483150
C	-0.8271660	-0.9382030	2.7982710
H	-1.0301110	-0.4865340	3.8060100
H	-0.4327340	-1.9712580	3.0218670
C	4.6517330	-1.2081320	1.3400930
H	4.5349550	-0.6775220	2.2972990
C	-3.3050570	-1.0966280	-0.6643000
C	3.1925890	1.7709320	-0.6142610
C	-2.0530320	-4.2045760	1.2853540
H	-2.3269400	-3.7711390	2.2592410
C	4.5659900	2.0916100	-0.5160820
H	5.2358240	1.4698300	0.0998440
C	-3.3048410	0.0452970	-1.4952970
H	-2.3848350	0.6466660	-1.5771160
C	-2.1552900	-1.0662360	2.0417350
H	-2.6594280	-0.0782930	1.9769490
H	-2.8673790	-1.7685530	2.5227080
C	2.3485400	0.9393550	2.0781040
H	3.3392490	0.9971270	2.5746920
H	1.9539770	1.9758090	2.0104240

C	-1.7494450	-3.3702250	0.1878370
C	1.3538990	0.0351780	2.8167670
H	1.8554140	-0.9499640	3.0426250
H	1.1393370	0.4835810	3.8234960
C	-4.4673590	0.4077320	-2.2012040
H	-4.4568400	1.2964770	-2.8525450
C	-4.4817900	-1.8724140	-0.5536850
H	-4.4914820	-2.7744460	0.0796550
C	4.9929610	-2.5948480	-1.0811520
H	5.1202010	-3.1365370	-2.0320660
C	3.9837090	-1.6261300	-0.9622930
H	3.3180710	-1.4246420	-1.8171710
C	0.4198110	-0.7100200	-1.8803310
C	2.3523880	2.5678210	-1.4231950
H	1.2823510	2.3141550	-1.4971600
C	5.6560450	-2.1888980	1.2228500
H	6.3037120	-2.4097110	2.0864280
C	-2.0051740	-5.6061020	1.1523530
H	-2.2402000	-6.2427650	2.0203590
C	-5.6415670	-1.5056820	-1.2580570
H	-6.5536150	-2.1172250	-1.1663230
C	-1.3928240	-3.9674960	-1.0448990
H	-1.1333420	-3.3312720	-1.9066470
C	5.8319350	-2.8810170	0.0131270
H	6.6186480	-3.6468420	-0.0776040
C	-5.6362780	-0.3653830	-2.0837170
H	-6.5443770	-0.0834070	-2.6403460
C	5.0877060	3.1963820	-1.2111950

H	6.1599520	3.4370470	-1.1297620
C	-1.6610470	-6.1892560	-0.0784020
H	-1.6254410	-7.2854920	-0.1813630
C	2.8788960	3.6722860	-2.1184360
H	2.2173030	4.2860810	-2.7507430
C	4.2452760	3.9884800	-2.0140830
H	4.6573320	4.8502670	-2.5632260
C	-1.3562590	-5.3645460	-1.1785790
H	-1.0805740	-5.8131370	-2.1463690
H	0.9137910	-1.8714490	0.1899460
O	-0.6934410	1.7635330	-0.2888860
C	-0.9843030	2.4032490	0.7449300
H	-0.7886460	1.9390620	1.7470020
C	-1.8598430	4.3646110	-0.4859170
N	-1.5263760	3.6424770	0.7462790
C	-1.8681180	4.3463240	1.9851330
C	-3.3311500	4.8081580	1.9272010
O	-3.5789940	5.5841390	0.7580200
C	-3.3210750	4.8300810	-0.4249720
H	-1.6831640	3.6890030	-1.3448000
H	-1.1878070	5.2467700	-0.5841330
H	-1.6991590	3.6700730	2.8472630
H	-1.2040730	5.2328500	2.1008140
H	-3.5664290	5.4497270	2.8003090
H	-4.0038620	3.9147570	1.9525920
H	-3.5497300	5.4898690	-1.2860670
H	-3.9969000	3.9405170	-0.4728170

TSB-C

Frequencies, energies and thermodynamic properties:

Lowest Vibrational Mode (cm^{-1}) = -79.3398

2nd Lowest Vibrational Mode (cm^{-1}) = 4.3315

E(RB-P86) (h) = -2430.14513

Thermal correction to Enthalpy (h) = 0.67101

Thermal correction to Gibbs Free Energy (h) = 0.55063

Total Entropy (cal/Kmol) = 253.362

E(RPBE1PBE) (h) = -2429.90525

Optimised cartesian coordinates (Å):

Ru	-0.4702980	0.4697650	-0.1318770
P	-2.3097420	-0.9087990	0.2451620
P	1.2799470	1.9813570	0.1741440
N	-0.0761610	0.0296840	1.8582940
O	-0.7561820	0.7303940	-3.1455320
C	-3.9533940	-0.0694490	0.4291370
C	0.7979040	0.8882250	2.6582090
H	1.2081240	0.3186620	3.5322660
H	0.2421170	1.7550200	3.1100380
C	-4.8047130	-0.2567480	1.5401810
H	-4.5156060	-0.9322630	2.3596640
C	2.7657590	2.0441800	-0.9373220
C	-2.7038400	-2.3788230	-0.8170730
C	1.3421780	4.5920090	1.3979060
H	2.0561210	4.1889850	2.1325170
C	-4.0094510	-2.9032000	-0.9445590
H	-4.8508970	-2.4240410	-0.4194500
C	3.1665970	0.8406440	-1.5638970

H	2.5848390	-0.0818750	-1.3944110
C	1.9652510	1.4186360	1.8185920
H	2.6644970	0.5940290	1.5638980
H	2.5531370	2.2011710	2.3408590
C	-1.8888880	-1.6474920	1.9079430
H	-2.7751850	-2.0256620	2.4578880
H	-1.2505660	-2.5246060	1.6696490
C	0.8206990	3.7678290	0.3758490
C	-1.0896710	-0.6068400	2.7005350
H	-1.7968020	0.1424650	3.1503480
H	-0.6054870	-1.1117000	3.5764420
C	4.2966350	0.8226450	-2.4003130
H	4.6008690	-0.1190330	-2.8850340
C	3.5027830	3.2257650	-1.1736700
H	3.1957730	4.1723000	-0.7017810
C	-5.5981890	1.4703590	-0.5313600
H	-5.9034770	2.1465060	-1.3458250
C	-4.3619220	0.8082930	-0.6031680
H	-3.6984780	0.9787820	-1.4661750
C	-0.6381180	0.6229760	-1.9717480
C	-1.6369850	-2.9981420	-1.5087830
H	-0.6173000	-2.5862890	-1.4173350
C	-6.0378930	0.4187980	1.6171940
H	-6.6873980	0.2670840	2.4941200
C	0.9576520	5.9429300	1.4924610
H	1.3675880	6.5703840	2.2999960
C	4.6288250	3.2026310	-2.0167440
H	5.1942270	4.1311800	-2.1965640

C	-0.0918510	4.3251640	-0.5504070
H	-0.5205840	3.6861580	-1.3386460
C	-6.4388160	1.2801100	0.5819540
H	-7.4044740	1.8067850	0.6430030
C	5.0293360	2.0020530	-2.6299960
H	5.9092290	1.9869260	-3.2929700
C	-4.2435140	-4.0345330	-1.7468550
H	-5.2661390	-4.4337950	-1.8420040
C	0.0569180	6.4888670	0.5618100
H	-0.2425150	7.5464090	0.6365800
C	-1.8764360	-4.1319260	-2.3055510
H	-1.0396810	-4.6076510	-2.8419690
C	-3.1784050	-4.6513720	-2.4280200
H	-3.3646360	-5.5347720	-3.0596210
C	-0.4642500	5.6767870	-0.4629070
H	-1.1733870	6.0961010	-1.1943400
H	-1.4537660	1.6845830	0.0009420
O	1.4905790	-1.9532590	-0.5605420
C	1.8010150	-2.3570940	0.5717170
H	1.4074210	-1.8428810	1.4904640
C	3.2316990	-4.1976930	-0.2526030
N	2.6097720	-3.4214220	0.8227930
C	2.9696930	-3.8411920	2.1775250
C	4.4977980	-3.9340270	2.2986890
O	5.0437400	-4.7760620	1.2867560
C	4.7457900	-4.2765910	-0.0165720
H	2.9908330	-3.7040260	-1.2142550
H	2.7996340	-5.2241650	-0.2617690

H	2.5578430	-3.1132020	2.9060840
H	2.5209910	-4.8385740	2.3896390
H	4.7814430	-4.3742140	3.2760960
H	4.9381690	-2.9077820	2.2312740
H	5.2138100	-4.9708300	-0.7433770
H	5.1981980	-3.2632900	-0.1552210

1C’

Frequencies, energies and thermodynamic properties:

Lowest Vibrational Mode (cm^{-1}) = 15.9984

2nd Lowest Vibrational Mode (cm^{-1}) = 23.3695

E(RB-P86) (h) = -2430.17889

Thermal correction to Enthalpy (h) = 0.67203

Thermal correction to Gibbs Free Energy (h) = 0.55405

Total Entropy (cal/Kmol) = 248.293

E(RPBE1PBE) (h) = -2429.94487

Optimised cartesian coordinates (Å):

Ru	0.0000320	0.2365990	-0.1145950
P	-2.3015930	0.4352210	0.2414340
P	2.3017170	0.4350110	0.2414450
N	0.0000160	-0.3843490	2.0237120
O	0.0002480	1.1303200	-2.9954090
C	-3.0023100	2.1520990	0.2966770
C	1.2223880	-0.0016620	2.7762670
H	1.2667820	-0.5522680	3.7450500
H	1.1419370	1.0793060	3.0200190
C	-3.3934930	2.7941850	1.4908650
H	-3.3204110	2.2763830	2.4595270

C	3.6101870	-0.4450390	-0.7368090
C	-3.6100880	-0.4448020	-0.7368410
C	3.3936340	2.7938800	1.4910270
H	3.3205300	2.2760200	2.4596560
C	-4.9778090	-0.3051000	-0.4016600
H	-5.2807650	0.3473720	0.4335430
C	3.2429970	-1.2654750	-1.8249120
H	2.1802720	-1.3827370	-2.0868180
C	2.4930420	-0.2663520	1.9676390
H	2.6499440	-1.3561940	1.8278400
H	3.3923610	0.1209070	2.4889190
C	-2.4930300	-0.2660900	1.9676570
H	-3.3922710	0.1213160	2.4889580
H	-2.6501330	-1.3559050	1.8278550
C	3.0024560	2.1518670	0.2967980
C	-1.2223290	-0.0015600	2.7762660
H	-1.1417730	1.0794020	3.0200130
H	-1.2667730	-0.5521580	3.7450510
C	4.2329840	-1.9401670	-2.5651480
H	3.9377850	-2.5751310	-3.4155920
C	4.9779020	-0.3053980	-0.4015750
H	5.2808550	0.3470330	0.4336620
C	-3.6011760	4.1717510	-0.9505620
H	-3.6796250	4.7059490	-1.9108310
C	-3.1090390	2.8573370	-0.9251990
H	-2.8007670	2.3761900	-1.8677260
C	0.0001280	0.7765210	-1.8699900
C	-3.2428920	-1.2652810	-1.8249090

H	-2.1801610	-1.3825770	-2.0867760
C	-3.8822960	4.1145890	1.4637200
H	-4.1821620	4.6019620	2.4051840
C	3.8824530	4.1142800	1.4639660
H	4.1823010	4.6015970	2.4054650
C	5.9604610	-0.9881630	-1.1371050
H	7.0226350	-0.8767570	-0.8663930
C	3.1092310	2.8571700	-0.9250390
H	2.8009640	2.3760880	-1.8675990
C	-3.9888440	4.8058690	0.2454180
H	-4.3720300	5.8383920	0.2258460
C	5.5891550	-1.8056690	-2.2221850
H	6.3615780	-2.3352810	-2.8024600
C	-5.9603660	-0.9878610	-1.1371970
H	-7.0225450	-0.8764190	-0.8665200
C	3.9890360	4.8056260	0.2457070
H	4.3722260	5.8381480	0.2261980
C	-4.2328760	-1.9399630	-2.5651590
H	-3.9376700	-2.5749570	-3.4155790
C	-5.5890520	-1.8054140	-2.2222400
H	-6.3614760	-2.3350170	-2.8025220
C	3.6013900	4.1715750	-0.9503190
H	3.6798760	4.7058240	-1.9105560
H	0.0000260	1.8090320	0.4833630
O	0.0001370	-2.3842980	-1.6928920
C	0.0000450	-1.8234500	-0.5977670
H	0.0000010	-1.4351230	1.8233880
C	-1.1749320	-4.6274030	1.7823130

C	-1.1977800	-3.6643940	0.5932560
N	-0.0001140	-2.8019610	0.6190840
C	1.1971820	-3.6649230	0.5932360
C	1.1739070	-4.6279140	1.7822960
O	-0.0006880	-5.4358750	1.7817120
H	-2.0406110	-5.3205960	1.7388100
H	-1.2427250	-4.0452100	2.7370640
H	-2.1116920	-3.0332760	0.6333880
H	-1.2441720	-4.2490880	-0.3587260
H	2.1114030	-3.0342420	0.6333720
H	1.2432760	-4.2496180	-0.3587560
H	2.0392850	-5.3214850	1.7388020
H	1.2419620	-4.0457320	2.7370350

TSC-D

Frequencies, energies and thermodynamic properties:

Lowest Vibrational Mode (cm^{-1}) = -1350.3371

2nd Lowest Vibrational Mode (cm^{-1}) = 9.5222

E(RB-P86) (h) = -2430.16900

Thermal correction to Enthalpy (h) = 0.66693

Thermal correction to Gibbs Free Energy (h) = 0.54862

Total Entropy (cal/Kmol) = 248.996

E(RPBE1PBE) (h) = -2429.92784

Optimised cartesian coordinates (Å):

Ru	0.0000130	0.3592250	-0.1015160
P	-2.3127870	0.4610010	0.2540210
P	2.3128110	0.4608880	0.2540280
N	-0.0000040	-0.3994060	1.9649390

O	-0.0000370	1.5440390	-2.8868200
C	-3.1796550	2.1100090	0.3397260
C	1.1930420	-0.0128270	2.7386440
H	1.2307710	-0.5731960	3.7068590
H	1.1340350	1.0699710	3.0153700
C	-2.4598400	3.3094600	0.1647550
H	-1.3752560	3.2513200	-0.0196580
C	3.4919100	-0.5070780	-0.8029000
C	-3.4919000	-0.5069990	-0.8028580
C	2.4600710	3.3093060	0.1639990
H	1.3755780	3.2511900	-0.0209430
C	-4.5298170	-1.3066580	-0.2711790
H	-4.6740780	-1.3911390	0.8173210
C	3.3437440	-0.4262460	-2.2069790
H	2.5316800	0.1799630	-2.6380750
C	2.4947340	-0.2603220	1.9736100
H	2.6757830	-1.3484070	1.8562020
H	3.3682850	0.1679160	2.5059400
C	-2.4947300	-0.2601240	1.9736450
H	-3.3682400	0.1682200	2.5059560
H	-2.6758760	-1.3481990	1.8562950
C	3.1797110	2.1098680	0.3397700
C	-1.1930070	-0.0127040	2.7386480
H	-1.1339080	1.0701000	3.0153320
H	-1.2307650	-0.5730340	3.7068840
C	4.2181600	-1.1214700	-3.0587410
H	4.0880970	-1.0483720	-4.1501300
C	4.5298330	-1.3067570	-0.2712650

H	4.6740910	-1.3913080	0.8172300
C	-5.2235660	3.4159630	0.6672650
H	-6.3072400	3.4512890	0.8628490
C	-4.5716770	2.1742340	0.5856910
H	-5.1592760	1.2499220	0.7068100
C	-0.0000070	1.0586690	-1.8102590
C	-3.3437430	-0.4262360	-2.2069430
H	-2.5316860	0.1799590	-2.6380750
C	-3.1158420	4.5524740	0.2390140
H	-2.5409000	5.4815810	0.0970470
C	3.1161220	4.5522990	0.2381630
H	2.5413220	5.4813980	0.0955650
C	5.3997920	-2.0081750	-1.1270480
H	6.2021700	-2.6288880	-0.6972730
C	4.5716050	2.1740790	0.5864540
H	5.1590820	1.2497710	0.7082090
C	-4.4968980	4.6091340	0.4927110
H	-5.0104240	5.5820870	0.5514570
C	5.2477050	-1.9160400	-2.5212320
H	5.9288760	-2.4662290	-3.1895730
C	-5.3997720	-2.0081300	-1.1269230
H	-6.2021440	-2.6288250	-0.6971130
C	4.4970460	4.6089500	0.4925820
H	5.0106070	5.5818880	0.5512680
C	-4.2181540	-1.1215130	-3.0586650
H	-4.0880970	-1.0484690	-4.1500590
C	-5.2476880	-1.9160680	-2.5211110
H	-5.9288540	-2.4663000	-3.1894220

C	5.2235370	3.4157900	0.6679490
H	6.3071070	3.4511110	0.8641110
H	0.0001060	1.8748030	0.5924360
O	-0.0000440	-2.2834370	-1.7225430
C	-0.0000380	-1.6318410	-0.7091630
H	-0.0000470	-1.7418560	1.4991850
C	1.2024440	-3.5334520	0.6844240
N	-0.0000640	-2.6810310	0.6992670
C	-1.2026350	-3.5333900	0.6844220
C	-1.1740730	-4.5439880	1.8364420
O	-0.0001470	-5.3506400	1.7939740
C	1.1738230	-4.5440530	1.8364410
H	2.1098760	-2.8982170	0.7481290
H	1.2465280	-4.0735390	-0.2894830
H	-2.1100260	-2.8980990	0.7481110
H	-1.2467370	-4.0734810	-0.2894820
H	-2.0413170	-5.2327850	1.7677760
H	-1.2384620	-4.0035050	2.8151370
H	2.0410290	-5.2328990	1.7677730
H	1.2382440	-4.0035740	2.8151370

TSC-CO

Frequencies, energies and thermodynamic properties:

Lowest Vibrational Mode (cm^{-1}) = -213.7898

2nd Lowest Vibrational Mode (cm^{-1}) = 19.7721

E(RB-P86) (h) = -2430.11352

Thermal correction to Enthalpy (h) = 0.670880

Thermal correction to Gibbs Free Energy (h) = 0.553458

Total Entropy (cal/Kmol) = 247.137

E(RPBE1PBE) (h) = -2429.88354

Optimised cartesian coordinates (Å):

Ru	-0.0197870	-0.0251170	0.0870440
P	-2.3147480	0.3373300	0.3780360
P	2.2803940	0.3151130	0.4200150
N	-0.0437630	-0.0479530	2.3605400
O	-0.0927880	0.7767200	-2.8194410
C	-2.9936020	2.0559060	0.0940790
C	1.1838040	0.5571460	2.9519640
H	1.2252320	0.3556160	4.0466210
H	1.0992560	1.6564530	2.8228040
C	-4.3457050	2.3347980	0.4068450
H	-5.0014290	1.5388870	0.7958710
C	3.6985000	-0.6419140	-0.3087470
C	-3.6863620	-0.6547360	-0.3893650
C	4.1477130	2.4545580	0.7529020
H	4.7144870	1.7647310	1.3991730
C	-4.6784190	-1.3334450	0.3532170
H	-4.6797230	-1.3028200	1.4535020
C	3.7925060	-0.6877500	-1.7188890
H	3.0181570	-0.2067880	-2.3363510
C	2.4471230	0.0448570	2.2658610
H	2.5621610	-1.0478830	2.4296440
H	3.3449880	0.5380960	2.6909530
C	-2.5329030	0.0997350	2.2247760
H	-3.4246300	0.6341350	2.6117890
H	-2.6840350	-0.9846830	2.4116560

C	2.9360690	2.0418720	0.1516130
C	-1.2683700	0.5905580	2.9249070
H	-1.1561100	1.6860510	2.7843960
H	-1.3311740	0.4028690	4.0209000
C	4.8626440	-1.3506760	-2.3403850
H	4.9212810	-1.3779330	-3.4399500
C	4.6913140	-1.2841210	0.4656630
H	4.6494800	-1.2667690	1.5654390
C	-2.7222280	4.3755400	-0.6346810
H	-2.0819520	5.1697020	-1.0506710
C	-2.1898970	3.0884700	-0.4306280
H	-1.1378600	2.8741320	-0.6714190
C	-0.0591940	0.2484690	-1.7564970
C	-3.7239740	-0.7194620	-1.8031320
H	-2.9478030	-0.2100740	-2.3965350
C	-4.8730810	3.6221550	0.2137550
H	-5.9260480	3.8241180	0.4673160
C	4.6549300	3.7449640	0.5245260
H	5.5947690	4.0554580	1.0083620
C	5.7576950	-1.9556530	-0.1611570
H	6.5242530	-2.4527240	0.4546860
C	2.2590060	2.9368090	-0.7037470
H	1.3226980	2.6165930	-1.1841580
C	-4.0619490	4.6469170	-0.3097270
H	-4.4777240	5.6548910	-0.4673140
C	5.8475710	-1.9884340	-1.5634790
H	6.6837240	-2.5135720	-2.0518810
C	-5.6872380	-2.0616160	-0.3059990

H	-6.4538270	-2.5865320	0.2861550
C	3.9700960	4.6329430	-0.3261080
H	4.3712850	5.6424370	-0.5094870
C	-4.7354950	-1.4411280	-2.4569470
H	-4.7503090	-1.4829030	-3.5575270
C	-5.7193280	-2.1163100	-1.7099150
H	-6.5091960	-2.6872180	-2.2232230
C	2.7769150	4.2227970	-0.9455410
H	2.2406140	4.9076200	-1.6216200
H	-0.0038990	1.5676530	0.3002620
O	1.2621560	-2.5048310	-2.4211940
C	0.2223230	-2.0646980	-2.0054450
H	-0.0584040	-1.0491710	2.6169890
C	-1.4266830	-3.6295890	1.2782980
C	-1.2014990	-3.1250280	-0.1466170
N	0.0353470	-2.3228070	-0.2729820
C	1.1436440	-3.1537070	0.2365270
C	0.8882220	-3.6892200	1.6470690
O	-0.3308880	-4.4186440	1.7376340
H	-2.3278430	-4.2761880	1.3299140
H	-1.5896580	-2.7659420	1.9742350
H	-2.0684470	-2.5352050	-0.4999810
H	-1.1314230	-4.0278210	-0.8041780
H	2.0903720	-2.5783320	0.2079680
H	1.2922210	-4.0254280	-0.4475460
H	1.6982920	-4.3841870	1.9549850
H	0.8803400	-2.8454300	2.3886420

----

fac-1A

Frequencies, energies and thermodynamic properties:

Lowest Vibrational Mode (cm^{-1}) = 17.8403

2nd Lowest Vibrational Mode (cm^{-1}) = 24.2992

E(RB-P86) (h) = -2029.29555

Thermal correction to Enthalpy (h) = 0.521660

Thermal correction to Gibbs Free Energy (h) = 0.420831

Total Entropy (cal/Kmol) = 212.211

E(RPBE1PBE) (h) = -2029.046920

Optimised cartesian coordinates (Å):

Ru 0.018371129 -0.6997253997 0.2113654263

H 0.0601554513 -2.314986864 0.2996715877

P 2.1259031615 -1.1642416798 -0.3486165335

P -0.5977573537 1.5901302722 -0.3035813086

N -0.3880077658 -0.9521105683 -1.7880893125

C 0.5078446537 -1.9208140693 -2.4459524544

H 0.3195320534 -1.969672037 -3.5452425273

H 0.321455528 -2.9380649308 -2.0396002296

C 1.9792876796 -1.5223958774 -2.1984206847

H 2.1994717638 -0.5740410679 -2.7325064901

H 2.7224130228 -2.279151444 -2.5295713942

C -0.7022157288 0.1738302253 -2.6743481516

H -1.3176866618 -0.1816413841 -3.5389372348

H 0.2184161178 0.6229326679 -3.1359814793

C -1.4660513642 1.2681376983 -1.9268249913

H -2.4723984391 0.8979198615 -1.6337725239

H -1.6134298979 2.1852622953 -2.5338640462

C 2.9758627301 -2.6671401223 0.3577324614

C 4.2994133147 -2.9616273461 -0.0488745864

H 4.8269840177 -2.2924282452 -0.7480029902

C 4.9569698442 -4.1032391071 0.4384006588

H 5.9849593835 -4.3233273867 0.1087360814

C 4.3076866563 -4.9578494601 1.3495121493

H 4.8266084177 -5.8492699534 1.7366291873

C 2.9974269236 -4.6662214083 1.7654834096

H 2.4842079052 -5.3271897001 2.4820768245

C 2.3328809027 -3.5290054506 1.2691237846

H 1.3024571365 -3.3017172803 1.5839423809

C 3.4822244553 0.0854743898 -0.1440223812

C 4.1914342117 0.6586775359 -1.2216589781

H 3.9521432234 0.3859614504 -2.2607895671

C 5.228560181 1.5803097676 -0.9807588

H 5.7762676747 2.0159847691 -1.8315620511

C 5.5672532644 1.9381029235 0.3348477137

H 6.3818305352 2.6560069827 0.520552775

C 4.8573740224 1.3783722649 1.4147962384

H 5.1129536935 1.6580296217 2.4492732873

C 3.8196440579 0.4639292089 1.1777103504

H 3.2640503384 0.0372465829 2.0286409397

C 0.6428040714 2.9240955994 -0.6254040101

C 1.4541579793 3.3429577605 0.4559952032

H 1.3375169278 2.8691016359 1.4443066965

C 2.4053308265 4.3604963303 0.2813793044

H 3.0221464856 4.6827796762 1.1350175935

C 2.5776904384 4.9597003638 -0.9809889918

H 3.3287418243 5.7536276085 -1.118768775

C 1.7902129915 4.5370466541 -2.0650813084

H 1.9204301414 4.9978100992 -3.0573599826

C 0.8243523527 3.5279572444 -1.88952222

H 0.2110800761 3.2215788916 -2.7504106289

C -1.8907369932 2.4636524195 0.6994547333

C -2.1427074988 3.8487835966 0.5775782439

H -1.529969525 4.4632886746 -0.1012163584

C -3.1707125347 4.4499599088 1.3249389249

H -3.3576428099 5.5311714284 1.2246961262

C -3.9575091423 3.6760967199 2.1983107789

H -4.7600345003 4.1506838129 2.7851828636

C -3.7119881868 2.2969215461 2.3251636768

H -4.319733126 1.6867901946 3.0121661118

C -2.6808467467 1.6946527713 1.5827662734

H -2.4762294272 0.615774709 1.6919104619

C 0.2731752392 -0.5482049015 2.0548404786

O 0.4850268967 -0.4368294795 3.2137862638

----

fac-1B

Frequencies, energies and thermodynamic properties:

Lowest Vibrational Mode (cm^{-1}) = 14.0885

2nd Lowest Vibrational Mode (cm^{-1}) = 21.4451

E(RB-P86) (h) = -2430.173261

Thermal correction to Enthalpy (h) = 0.671916

Thermal correction to Gibbs Free Energy (h) = 0.551184

Total Entropy (cal/Kmol) = 254.102

E(RPBE1PBE) (h) = -2429.931865

Optimised cartesian coordinates (Å):

Ru 4.8017905109 2.9824267661 4.8368357577

H 5.6085369394 4.3829161339 4.6710936783

P 3.9582043204 4.1073736792 6.6124019752

P 3.3613070307 1.0710504759 4.4997696102

N 3.2291697521 3.9833444204 3.7648016845

C 2.9105179223 5.278702236 4.3678828872

H 2.0417365636 5.7653061102 3.8556017807

H 3.7766933638 5.9678315894 4.2496280001

C 2.577639387 5.1408342365 5.8745893527

H 1.6337241177 4.568149164 5.9948367181

H 2.4565642671 6.107677471 6.4083359256

C 1.9983667104 3.2414694129 3.4810332252

H 1.4493927004 3.7081135546 2.620301636

H 1.2575310541 3.2593477322 4.3303301209

C 2.2846905961 1.7747056166 3.1447881585

H 2.8983984079 1.6985178122 2.2221070698

H 1.3583709291 1.1868968464 2.9764840535

C 5.0488664746 5.367221589 7.4655906354

C 4.5198733339 6.1334701459 8.5318285356

H 3.4847801555 5.9668745739 8.8725110957

C 5.3055802417 7.1051896906 9.1735682902

H 4.8768334008 7.6966911601 9.9984867602

C 6.6374407123 7.3170262834 8.7689902739

H 7.2557154111 8.0747347153 9.2765460733

C 7.1735821298 6.5546043645 7.7173409873

H 8.2160609113 6.7109288595 7.3963512232

C 6.3827445379 5.5884661283 7.0666537879

H 6.7913851768 4.9990823341 6.2313199458

C 3.2555030737 3.2772141134 8.1209621186

C 1.9115285455 3.4040373075 8.5338153491

H 1.1993964267 4.0033851321 7.9462190532

C 1.4646941544 2.775874327 9.712499322

H 0.4133257712 2.8875804653 10.0225469421

C 2.3539377147 2.0185831464 10.4932247979

H 2.0037537074 1.5336806357 11.4183354222

C 3.6941217129 1.8795948482 10.0842712242

H 4.3974453494 1.2830755198 10.6871478087

C 4.1394687637 2.4983163764 8.9054932373

H 5.1895781632 2.3790958579 8.5926461631

C 2.186958272 0.3646844113 5.7534659237

C 2.7379165329 -0.3781064183 6.8247392026

H 3.8301235189 -0.5116294136 6.8934696329

C 1.9069688064 -0.9577323942 7.7965813399

H 2.3526850855 -1.5410590923 8.6179714773

C 0.510762674 -0.79014387 7.7257617022

H -0.1411044087 -1.2427479887 8.489791741

C -0.045323703 -0.0395988949 6.6762423449

H -1.1365783638 0.1000423633 6.6131780222

C 0.7858074658 0.5334405619 5.6947659293

H 0.325623127 1.1120190037 4.8791280602

C 4.0206872965 -0.5002262994 3.7503566542

C 3.145683998 -1.4966194853 3.257369802

H 2.0542835323 -1.3602148332 3.3316755886

C 3.6563162053 -2.6717782924 2.6808110394

H 2.9642820676 -3.4397127131 2.299185754

C 5.0478875367 -2.8708754471 2.5980245499

H 5.4476175595 -3.7952328975 2.1510600082

C 5.9240730922 -1.8889727683 3.0923039061

H 7.0138648246 -2.041471576 3.0357572861

C 5.4137535721 -0.7076624449 3.6636059171

H 6.0983590088 0.0651070868 4.0448959373

C 6.2000656752 2.2068608091 5.7783219906

O 7.061214491 1.7361833178 6.4422232536

H 4.6059963321 4.0226301211 1.9853532355

C 5.3937321874 3.2385391432 1.8516707703

O 5.6617183022 2.4726764704 2.8101492108

C 5.6440434025 4.0528290581 -0.4608125828

C 6.9788598723 2.1209204106 0.3151210803

C 5.2918267675 3.234071572 -1.7106206779

H 6.5201506834 4.7046264689 -0.6786497057

H 4.7965136915 4.6990634124 -0.156229479

C 6.5664593976 1.3994965478 -0.9750605789

H 7.9678453428 2.6117308106 0.1736650299

H 7.0514052607 1.4193701131 1.1678604874

H 5.1584162902 3.904830269 -2.5832349614

H 4.3331766191 2.6844108362 -1.5367951037

H 7.37504976 0.7175125938 -1.3068104232

H 5.6483863476 0.7903809873 -0.7842025262

N 5.980106423 3.1458507541 0.6405968954

O 6.3359756123 2.3218213045 -2.0384306652

----

fac-TSB-C

Frequencies, energies and thermodynamic properties:

Lowest Vibrational Mode (cm^{-1}) = -228.5862

2nd Lowest Vibrational Mode (cm^{-1}) = 14.1205

E(RB-P86) (h) = -2430.130796

Thermal correction to Enthalpy (h) = 0.670557

Thermal correction to Gibbs Free Energy (h) = 0.550929

Total Entropy (cal/Kmol) = 251.777

E(RPBE1PBE) (h) = -2429.8924955

Optimised cartesian coordinates (Å):

Ru -0.6502554039 -0.2418958981 -0.0278763344

H -1.5966181812 -1.5457053527 -0.1119064781

P 0.4387682389 1.923093442 -0.4356406695

P 0.9315930365 -1.8330486376 -0.3292610858

C -0.2270387667 2.2041679057 -2.1589628612

H -1.2955082706 2.4541574513 -1.9969010981

H 0.2525438167 3.0587502575 -2.6795288329

C -0.0908492742 0.9116335726 -2.9705820918

H -0.557612421 1.0360252163 -3.9753572615

H 0.9831757114 0.686366477 -3.145102712

N -0.7060697699 -0.2406539579 -2.2670390728

H -1.756974199 -0.1045966421 -2.1773975743

C -0.3032900594 -1.558934478 -2.8042796307

H -0.2313515618 -1.5338680448 -3.9161769204

H -1.0957139532 -2.2883993523 -2.5385106961

C 1.0426608287 -1.9857676503 -2.1992732083

H 1.8472597274 -1.3001589371 -2.5386130882

H 1.3279536994 -3.0123986033 -2.5065562842

C 0.006313671 3.4757573799 0.4863273508

C 0.5005855331 4.728104216 0.0521323175

H 1.1366284407 4.7927611989 -0.8457705265

C 0.1976685308 5.8998571106 0.7661334952

H 0.5853873646 6.8695814129 0.4147502219

C -0.5915259726 5.8326788151 1.9298044297

H -0.8235296846 6.7504295123 2.4936517883

C -1.0800096615 4.5899392287 2.3703211852

H -1.6991473545 4.530731189 3.2798620113

C -0.7864988715 3.4163850729 1.6521269416

H -1.1914520164 2.4482810159 1.9792599594

C 2.2784526672 2.1425136516 -0.5947600901

C 2.9372246183 2.4830813179 -1.7974523774

H 2.368719531 2.6312143103 -2.7283532554

C 4.3344514467 2.6569368892 -1.8243906232

H 4.8306872371 2.9248217159 -2.7710584796

C 5.0902412091 2.5006970423 -0.6503941154

H 6.1822945187 2.6437459309 -0.6714052567

C 4.4437759249 2.1576285096 0.5522841555

H 5.0280896227 2.0286504447 1.4768288637

C 3.0527706358 1.9708540848 0.5772759652

H 2.5584028676 1.6934357837 1.5226719004

C 0.5763367695 -3.5757413444 0.2451582495

C 1.5211268678 -4.5974352202 -0.0146521477

H 2.4677494091 -4.361582219 -0.5277486468

C 1.2710962219 -5.9198058972 0.3871559326

H 2.0135941461 -6.7048983145 0.1724253511

C 0.0809495539 -6.2385188027 1.0684598228

H -0.1115693119 -7.2746986255 1.3893528147

C -0.8565233476 -5.2280484627 1.3408060083

H -1.7881344571 -5.4672502328 1.8780235313

C -0.6124988809 -3.9045451169 0.9279293183

H -1.3509550256 -3.1127797509 1.1273819862

C 2.6807547752 -1.6682929852 0.2683745632

C 3.8094406728 -1.7237245732 -0.5785787154

H 3.6929627055 -1.8434202762 -1.6663832913

C 5.107565316 -1.6370643774 -0.0408761812

H 5.9780387022 -1.6832121308 -0.714507645

C 5.2935980886 -1.5000932181 1.3449240793

H 6.3106215109 -1.4380943619 1.7634374016

C 4.1733204156 -1.4405953888 2.1956233681

H 4.3093119728 -1.3302757349 3.283264802

C 2.8774372906 -1.5184241894 1.6619734387

H 2.0085421278 -1.4652593818 2.3376491697

C -2.9342708829 0.4931247133 -0.6269870319

O -2.4654134622 1.4677183443 0.0756939191

N -4.1503813806 -0.0349535173 -0.2150286551

C -4.7509440171 -1.1707882052 -0.8933876738

H -4.635445837 -2.1003770106 -0.2818614374

H -4.2166441785 -1.3236418446 -1.8553162775

C -6.2446943998 -0.9134967047 -1.1320169563

H -6.3615682543 -0.0832326018 -1.8744107061

H -6.7419660769 -1.8190274455 -1.5378326281

O -6.9179686298 -0.5834817212 0.0829166017

C -6.3486247961 0.5837686708 0.682134503

H -6.9261565301 0.7760033732 1.6103468932

H -6.4759759495 1.4627847806 0.0008027864

C -4.8609477567 0.3925327116 0.9925026328

H -4.3871473235 1.3359205978 1.3357389542

H -4.7454624194 -0.3702630827 1.8007865944

C -0.6164181963 -0.3168457972 1.804984768

O -0.5796478891 -0.3813482575 2.983656542

1C

Frequencies, energies and thermodynamic properties:

Lowest Vibrational Mode (cm^{-1}) = 17.6903

2nd Lowest Vibrational Mode (cm^{-1}) = 20.8332

E(RB-P86) (h) = -2430.2079887

Thermal correction to Enthalpy (h) = 0.674131

Thermal correction to Gibbs Free Energy (h) = 0.557427

Total Entropy (cal/Kmol) = 245.625

E(RPBE1PBE) (h) = -2429.9741146

Optimised cartesian coordinates (Å):

Ru 0.074608034 0.3954442713 0.1788005334

P -2.2049652174 0.7923591777 0.4749610698

P 2.4015653708 0.4568245907 0.4296292111

N 0.1282394828 0.8236063854 2.3683926826

O 0.0783540832 0.2100673505 -2.8291487434

C -2.9797700323 2.3829851712 -0.103261206

C 1.4037983765 1.3931333339 2.8571800032

H 1.4348059423 1.3703823101 3.9721668349

H 1.4569780519 2.4593903763 2.5490444852

C -4.3355582386 2.6646297911 0.1905933695

H -4.9494128147 1.9301551725 0.7375865113

C 3.5862460782 -0.9135979281 0.0105586354

C -3.4827731134 -0.4471268298 -0.045438664

C 3.8762649572 2.9368862815 0.4794113358

H 3.8682103892 2.882877058 1.5788213296

C -3.9990080257 -0.3766026039 -1.3613380865

H -3.6967586031 0.4476216582 -2.0273087807

C 3.0799491067 -2.1975282476 -0.2795996521

H 1.9895879308 -2.3558481089 -0.2822714527

C 2.5808436918 0.5992133755 2.2871723975

H 2.5429190322 -0.4490444205 2.6536861544

H 3.5599757606 1.0206353242 2.5932901306

C -2.3372964328 0.8953161896 2.3421389285

H -3.2449876847 1.4472625605 2.6594189641

H -2.4102561738 -0.1423794576 2.7275225818

C 3.2769800377 1.9223168573 -0.2975625885

C -1.0602524658 1.5454341178 2.8782428763

H -0.9944724399 2.6081296778 2.5590400429

H -1.0702253991 1.5367982127 3.993594138

C 3.9576665368 -3.2606284329 -0.5609292868

H 3.5504524831 -4.258593375 -0.7900441136

C 4.9852453603 -0.705916182 0.010261307

H 5.3966036493 0.2939820859 0.2226302126

C -2.8046317361 4.5416987265 -1.2367419242

H -2.2019526861 5.2726191232 -1.7992637882

C -2.2207645645 3.3305042225 -0.8192883044

H -1.162528112 3.1063648422 -1.0324545797

C 0.066188266 0.2290886341 -1.6440452809

C -3.8828994914 -1.5185955422 0.7840206954

H -3.489390364 -1.6136328321 1.8072775429

C -4.9141657792 3.8768454058 -0.2198612954

H -5.9692701969 4.0871539214 0.0177797843

C 4.4951481967 4.0401421954 -0.1402006509

H 4.9552185704 4.8247545177 0.4815658582

C 5.8596095323 -1.7701701886 -0.2692703811

H 6.947874689 -1.5977835742 -0.2671339733

C 3.3105531653 2.0342840525 -1.7081708152

H 2.8368071401 1.2599123228 -2.3329317121

C -4.1494529305 4.817896883 -0.9363940115

H -4.6060662339 5.766523243 -1.2612477401

C 5.3477517866 -3.0497130915 -0.556255159

H 6.0346367909 -3.8813063967 -0.7808452893

C -4.899745836 -1.3471996851 -1.8308275635

H -5.2922752332 -1.2746494208 -2.8576406987

C 4.5287057172 4.1391739464 -1.5410267029

H 5.0143576566 5.001768959 -2.0242536583

C -4.7884885429 -2.4870084826 0.3132265691

H -5.0956934146 -3.3106441676 0.9772857584

C -5.2997150843 -2.4053850174 -0.9940490072

H -6.0081216617 -3.1650953543 -1.3604832752

C 3.935352393 3.1301685479 -2.3240106941

H 3.9545650601 3.1998401752 -3.4233530963

H 0.2798577118 2.0320550948 0.0109018427

O -0.0868082667 -1.8025182891 2.2156349668

C -0.2189560942 -1.6445507381 0.9597310863

H 0.0708904279 -0.2061133603 2.6425797198

C -0.2331521773 -5.228071304 0.1936035231

C -0.8509731956 -4.051985489 0.9534754219

N -0.5757122455 -2.7891814561 0.2542303664

C -0.9526982225 -2.8352626037 -1.1534134613

C -0.3361398398 -4.0610893544 -1.8378511302

O -0.6723585393 -5.2733773313 -1.1635130758

H -0.5313891475 -6.1904056543 0.6592665805

H 0.8832551191 -5.1520205132 0.233718053

H -0.4423983583 -3.9689944938 1.9788818656

H -1.9548038795 -4.204536502 1.0288886841

H -0.6188406569 -1.90602105 -1.6476378033

H -2.0641325802 -2.8915630449 -1.2441094736

H -0.718721475 -4.1542038394 -2.8751458863

H 0.7764916083 -3.9467427804 -1.8863230478

----

fac-1C

Frequencies, energies and thermodynamic properties:

Lowest Vibrational Mode (cm^{-1}) = 8.9670

2nd Lowest Vibrational Mode (cm^{-1}) = 19.7040

E(RB-P86) (h) = -2430.2029602

Thermal correction to Enthalpy (h) = 0.673749

Thermal correction to Gibbs Free Energy (h) = 0.554348

Total Entropy (cal/Kmol) = 251.302

E(RPBE1PBE) (h) = -2429.9683009

Optimised cartesian coordinates (Å):

Ru 5.7338851425 12.1443051878 4.7244330318

H 4.2401265016 11.5845170685 4.391842946

P 7.6735461515 13.5518884639 4.8458633577

P 6.2736572545 10.0107967771 3.826945604

C 7.5141856953 14.4336072886 3.2011777279

H 6.7060861525 15.1853751926 3.309153971

H 8.444030411 14.9617368216 2.9091023484

C 7.101425512 13.4063766544 2.1470509035

H 6.946134223 13.9067440877 1.1628240047

H 7.9124979904 12.6605155363 2.0046089599

N 5.858041207 12.7028778661 2.5579785077

H 5.0751881989 13.4111916751 2.6278715636

C 5.5064284066 11.5749123733 1.6622599716

H 5.641385504 11.8644410664 0.5940698486

H 4.4313287651 11.3507588236 1.8137824813

C 6.3598226287 10.3413085046 1.9803190149

H 7.4220060266 10.5363302403 1.7245224906

H 6.0359176468 9.4593333 1.3907770837

C 7.734659905 14.9114393543 6.1119713397

C 7.2295176036 16.2094395791 5.8777820981

H 6.8152835265 16.4818486704 4.8956554925

C 7.2469906833 17.1782704271 6.8988945858

H 6.8545775464 18.1875202525 6.6954302658

C 7.7637633581 16.8644442736 8.16794504

H 7.7765490459 17.6245415403 8.9651051579

C 8.2658811141 15.5724241264 8.4120150766

H 8.6736372534 15.3148266547 9.4026051606

C 8.251032014 14.6052414792 7.393608373

H 8.6508379962 13.5986986269 7.5986494851

C 9.4576682005 13.0104041899 4.8876501001

C 10.5114493692 13.8824142986 4.5246815137

H 10.295164947 14.9123024944 4.1966559738

C 11.8467937613 13.4520272016 4.5934787451

H 12.6582462331 14.1386432269 4.3031977359

C 12.1483332089 12.150682258 5.0404909883

H 13.1965911985 11.8163891958 5.0977142861

C 11.1087198609 11.2834376952 5.4163167092

H 11.3342841395 10.2650661726 5.7701912442

C 9.7693070843 11.7104596244 5.3351785392

H 8.9543352016 11.0268752031 5.6190746655

C 4.9695825165 8.6720245591 3.9508257641

C 5.1650579821 7.438669041 3.2841808238

H 6.0889331909 7.259462376 2.7100396706

C 4.1945370513 6.4255772133 3.3536945039

H 4.3597545977 5.4737749063 2.8237550586

C 3.0192785588 6.623230138 4.1034802314

H 2.2605569353 5.8265343012 4.1626345376

C 2.8218393872 7.8400169019 4.7777695597

H 1.9071877936 8.0024346752 5.370262956

C 3.7890488318 8.860083339 4.6984559367

H 3.6283762179 9.8219888069 5.2091466123

C 7.7691499609 8.960759091 4.1855832736

C 8.7444210831 8.6025289954 3.2292192895

H 8.6562811472 8.934486418 2.1836136776

C 9.8456740905 7.8043696724 3.5951710266

H 10.5950424824 7.5323511079 2.8346168914

C 9.986874384 7.3523713453 4.9179250223

H 10.8471976382 6.7253832206 5.2008869666

C 9.0204697683 7.7059475842 5.879854625

H 9.1219041225 7.3579197199 6.9202210307

C 7.9242074986 8.5047921979 5.517771228

H 7.1786374142 8.7806179942 6.2817690449

C 4.5527187479 13.9345511517 4.7956669076

O 4.1821162819 14.4094816175 3.6797428745

N 4.0740257106 14.5863577314 5.9200676813

C 4.4278066652 14.3197180353 7.3073336862

H 5.0205289219 15.1759495332 7.7082463933

H 5.0577809973 13.4167001905 7.36068963

C 3.1616979172 14.1485834062 8.1554043086

H 2.6199231258 13.224344263 7.831155669

H 3.4243803449 14.0423344422 9.2281896107

O 2.3030268816 15.2848022689 8.0517985908

C 1.9335402036 15.5165689523 6.6927810953

H 1.2869273911 16.4187137107 6.6866463907

H 1.3357625775 14.6538303987 6.3036669387

C 3.1574885377 15.7276749231 5.7948142535

H 2.8689607187 15.8183445618 4.7303486842

H 3.6827968593 16.6636158645 6.100805693

C 5.6625589985 11.523547956 6.4455070281

O 5.6686197967 11.0298409152 7.5228964404

1C-

Frequencies, energies and thermodynamic properties:

Lowest Vibrational Mode (cm^{-1}) = 18.4248

2nd Lowest Vibrational Mode (cm^{-1}) = 23.3632

E(RB-P86) (h) = -2429.671302

Thermal correction to Enthalpy (h) = 0.656918

Thermal correction to Gibbs Free Energy (h) = 0.540044

Total Entropy (cal/Kmol) = 245.982

E(RPBE1PBE) (h) = -2429.4300021

Optimised cartesian coordinates (Å):

Ru -0.0481716838 0.34059614 0.0523304627

P -2.3504838153 0.4591328415 0.3002492781

P 2.2225678649 0.4412769435 0.5296720629

N -0.1548426631 -0.1330501347 2.2152933808

O 0.1004337069 1.500064763 -2.7342774308

C -3.1380832082 2.1431401998 0.1591809785

C 0.988296383 0.3058533422 2.9844948533

H 0.9690525947 -0.1260507253 4.0241218451

H 0.9971892956 1.4288662859 3.1436899925

C -3.6342912803 2.8665959975 1.2652941171

H -3.5859617196 2.4377071816 2.2782092708

C 3.5820156134 -0.5416162762 -0.2875297841

C -3.6086042269 -0.5357432622 -0.6532611899

C 3.3516691158 2.8744621876 1.6170133295

H 3.1682517087 2.462681717 2.6216333407

C -4.9765354725 -0.5152263865 -0.2918255807

H -5.316009134 0.1090077118 0.5514158116

C 3.2994084587 -1.3185383173 -1.4315220956

H 2.2678478171 -1.3558060076 -1.822533801

C 2.3059203027 -0.10017824 2.3143494555

H 2.3938785357 -1.2072379495 2.2765477308

H 3.2004042514 0.2886214683 2.8447787284

C -2.6138982134 -0.0839049523 2.066108637

H -3.5551348498 0.306522581 2.5066501569

H -2.7007705976 -1.1898476507 2.0181075447

C 3.0183645388 2.1258243593 0.4675560053

C -1.3672666601 0.31902089 2.863115109

H -1.3839102498 1.4432973643 3.0115688583

H -1.4558868567 -0.105979622 3.9020029526

C 4.3269636326 -2.0545516916 -2.0553982432

H 4.0979228169 -2.6556684271 -2.9505078189

C 4.9029983781 -0.5124391462 0.2194362975

H 5.1433370822 0.1039073804 1.1016569299

C -3.757459888 4.0179070777 -1.2940175317

H -3.7999455366 4.4636608835 -2.3010159503

C -3.204430091 2.7385140601 -1.1227441882

H -2.8095364375 2.1971479608 -1.997929833

C 0.0424244168 0.9903460846 -1.6644929298

C -3.1979289472 -1.3230858392 -1.7501339522

H -2.1320734728 -1.3483381522 -2.0322633139

C -4.1855466058 4.1514162043 1.0942328161

H -4.5649386101 4.7013315721 1.9708830514

C 3.9110499559 4.1615276987 1.4978663529

H 4.1635002551 4.7309270771 2.4072283401

C 5.9215805626 -1.2567168389 -0.3991187484

H 6.9453851958 -1.2300548415 0.0085011873

C 3.2530396165 2.6988117792 -0.8047361155

H 2.9871044581 2.1364412212 -1.7147007376

C -4.251230506 4.7306278349 -0.1843463891

H -4.6823071609 5.735895713 -0.3172940902

C 5.6351432926 -2.0287529289 -1.5419822987

H 6.4351030405 -2.6078641908 -2.0313713615

C -5.9150645835 -1.2789955524 -1.0061341144

H -6.9770334404 -1.2589089614 -0.7114983926

C 4.1453966225 4.718654186 0.2291223836

H 4.5825847843 5.7258446169 0.1369458149

C -4.1435834531 -2.0798232637 -2.4703648643

H -3.8132777863 -2.6885559745 -3.3278134099

C -5.4994538715 -2.0629363793 -2.1000876679

H -6.2360802262 -2.6581042216 -2.6639003999

C 3.813269218 3.9810827216 -0.9236969743

H 3.9889120523 4.4093928799 -1.9238437801

H -0.0774522584 1.9089022841 0.6499802236

O 0.1327167907 -1.8659852305 -2.026384998

C -0.0204263727 -1.7238913204 -0.777331099

C 0.5796486033 -4.0727631124 1.9596593908

C -0.4019315682 -3.0609233838 1.3612456606

N -0.1768652694 -2.9300544185 -0.0696325857

C -0.1786386899 -4.2171920294 -0.7681761114

C 0.8023780491 -5.1850561245 -0.1007964931

O 0.5362643953 -5.340802851 1.2947592869

H 0.341340793 -4.2705708961 3.0265454496

H 1.6190931948 -3.6559829892 1.9050169394

H -0.2859811999 -2.0508537958 1.8374554388

H -1.4420241461 -3.4344332868 1.5506943157

H 0.0955696275 -4.0138743218 -1.8232063768

H -1.2032276462 -4.6655119258 -0.7454041602

H 0.7295368743 -6.1980343145 -0.5517150762

H 1.8469815024 -4.8094952763 -0.250874893

----

fac-1C-

Frequencies, energies and thermodynamic properties:

Lowest Vibrational Mode (cm^{-1}) = 24.1531

2nd Lowest Vibrational Mode (cm^{-1}) = 24.8523

E(RB-P86) (h) = -2429.664735

Thermal correction to Enthalpy (h) = 0.657896

Thermal correction to Gibbs Free Energy (h) = 0.542756

Total Entropy (cal/Kmol) = 242.334

E(RPBE1PBE) (h) = -2429.426618

Optimised cartesian coordinates (Å):

Ru 0.1371981393 -0.8283420018 0.5336104738

H -0.3064172725 -2.3700361642 0.8598961414

P 1.0759024168 1.3660751074 0.6179902885

P -2.2513811096 -0.6766251197 0.685123484

C 1.0329241456 1.7469613151 2.4504088617

H 1.9697926126 1.3327931447 2.8774180488

H 1.0181672797 2.8371871612 2.6555175141

C -0.1721719758 1.0104449812 3.033491031

H -0.1748788587 1.1482710919 4.1508891544

H -1.0983365105 1.5678165607 2.6846740892

N -0.1305180595 -0.3950482811 2.6870253222

C -1.2774027632 -1.08984656 3.2356873111

H -1.4074758194 -0.8686230433 4.3311682365

H -1.1170507865 -2.188052165 3.1491434043

C -2.6162336883 -0.7249376836 2.5260280413

H -2.9667777937 0.2801239154 2.8374381251

H -3.4388653703 -1.4393259023 2.7406555556

C 2.8714147324 1.6861516868 0.201823862

C 3.8771299255 1.0433879949 0.9638985332

H 3.5964775371 0.3784464962 1.7958175379

C 5.2366585526 1.2256285243 0.6580110611

H 6.0034926064 0.7235208298 1.2699672735

C 5.6198041405 2.0391317506 -0.4254700664

H 6.6861208355 2.1790308068 -0.6654349397

C 4.6296493319 2.6689906533 -1.1991781109

H 4.9154946739 3.3058693322 -2.0522277228

C 3.2680895963 2.4948358037 -0.8875104779

H 2.5066358652 3.0005669243 -1.5013219636

C 0.2880336163 2.8353941069 -0.2223582007

C 0.3547471177 4.1514344941 0.2902701518

H 0.8718798146 4.3513716599 1.2424129788

C -0.224082324 5.2250373855 -0.4094369338

H -0.1685189808 6.2433318023 0.008723271

C -0.8648075806 5.0023352558 -1.6425294159

H -1.31435755 5.8445372155 -2.1928357744

C -0.9289453849 3.6991616165 -2.1670598516

H -1.4292316041 3.5149957462 -3.1312167662

C -0.3661207766 2.6228351658 -1.4568213103

H -0.4325319398 1.6000130794 -1.8611011656

C -3.2419337641 -2.1369318209 0.0499009677

C -4.6443116252 -2.1814151202 0.2426824218

H -5.1576320212 -1.3383994007 0.7336414198

C -5.3965093211 -3.2867691987 -0.1891354884

H -6.4862676698 -3.3046698131 -0.024686906

C -4.7603303603 -4.367038203 -0.8305834379

H -5.3495848496 -5.233597108 -1.1717502156

C -3.3697830014 -4.332312708 -1.0301450709

H -2.8617126089 -5.1728206271 -1.5302224008

C -2.6160065003 -3.2274571915 -0.5886282976

H -1.523787968 -3.2073164777 -0.7212111393

C -3.3228105084 0.702105398 0.0185337499

C -3.5991440801 1.8770600665 0.7568122259

H -3.2330688342 1.9809037205 1.7913698809

C -4.3361794254 2.9323170208 0.1904490495

H -4.547355031 3.8309249593 0.7927254337

C -4.8037759301 2.8443539893 -1.1336927674

H -5.3835962036 3.6703051815 -1.5757681859

C -4.5155413733 1.6923436358 -1.8890099877

H -4.8678215078 1.6111456966 -2.9304104619

C -3.7819140601 0.6367624661 -1.3212688097

H -3.5689107297 -0.2584778982 -1.9277037455

C 2.0440398999 -1.7197244145 0.9103709881

O 2.3152119835 -2.1994509239 2.0296735125

N 3.0351406207 -1.9223247879 -0.0869043133

C 3.114668796 -1.327315034 -1.4097731364

H 3.9467622026 -0.5808743636 -1.4377443745

H 2.1747486764 -0.7947038488 -1.63473433

C 3.3971131915 -2.4051248716 -2.4627457525

H 2.5236931529 -3.1025595929 -2.5240312963

H 3.551657646 -1.9484433377 -3.4631817339

O 4.5903441762 -3.1341627047 -2.1607777078

C 4.5090448928 -3.724604413 -0.8614480473

H 5.4777723596 -4.2401510986 -0.6883445738

H 3.6942759697 -4.4925743021 -0.8407436573

C 4.2433058959 -2.6827084141 0.2297926107

H 4.0890450724 -3.1556727314 1.2199552212

H 5.1254053191 -1.9975134743 0.302845814

C 0.0409539017 -1.0983528157 -1.2782083895

O -0.1845581743 -1.2117491256 -2.4451861211

----

fac-1D

Frequencies, energies and thermodynamic properties:

Lowest Vibrational Mode (cm^{-1}) = 13.9394

2nd Lowest Vibrational Mode (cm^{-1}) = 26.7744

E(RB-P86) (h) = -2142.58107269

Thermal correction to Enthalpy (h) = 0.531942

Thermal correction to Gibbs Free Energy (h) = 0.426245

Total Entropy (cal/Kmol) = 222.458

E(RPBE1PBE) (h) = -2142.338107

Optimised cartesian coordinates (Å):

Ru -0.2035620739 -0.7778815955 0.2532629122

H -0.0520965917 -2.4015617759 0.2443104971

P 2.1013840359 -1.1328332508 -0.4022919359

P -0.6592764174 1.5367106567 -0.2943230112

N -0.5014473958 -1.0170699845 -1.884739971

C 0.4682779419 -1.9323600095 -2.4642263771

H 0.3150905067 -2.0258861827 -3.5694209611

H 0.3260821408 -2.9500757646 -2.0366168931

C 1.9390864162 -1.4890010194 -2.2225435968

H 2.137708087 -0.5416263547 -2.7656262161

H 2.6938776485 -2.2338119856 -2.5506994181

C -0.6139089554 0.2000373601 -2.6818825297

H -1.0994122883 -0.0255687667 -3.6676433211

H 0.3787783117 0.6647352581 -2.9542975735

C -1.4358039623 1.2730626601 -1.9650051608

H -2.4626681807 0.9039143678 -1.7561884118

H -1.5272335959 2.2095386053 -2.5527748823

C 2.9516405289 -2.6369332945 0.296495062

C 4.2726710535 -2.934024663 -0.1174358005

H 4.7953315473 -2.2671706147 -0.8222476328

C 4.9334755774 -4.0733615525 0.369611185

H 5.9595349388 -4.2937506745 0.0344227591

C 4.2895962677 -4.9265382505 1.2860825278

H 4.8104090629 -5.8174997639 1.6715753728

C 2.9818085923 -4.6344201078 1.7079391057

H 2.4712358291 -5.2945015099 2.4271025028

C 2.3145576065 -3.4974374868 1.2140018686

H 1.285042873 -3.2766645146 1.5348301049

C 3.451224249 0.1199550908 -0.1955731771

C 4.1095648182 0.7486720828 -1.2743272952

H 3.824900482 0.5240897072 -2.3134316683

C 5.1535023507 1.6625270694 -1.0358588869

H 5.6615405704 2.1399398927 -1.8887906964

C 5.5507827129 1.9589614215 0.2787969824

H 6.371819366 2.6700202818 0.4620219125

C 4.8928155859 1.3437715887 1.3605437581

H 5.1944787195 1.5727613553 2.3950116201

C 3.8489241718 0.4353074716 1.1260995848

H 3.3441887555 -0.0415119373 1.9821250247

C 0.6452565537 2.8344214706 -0.5179222929

C 1.3825031407 3.2232338009 0.6251807292

H 1.1956030672 2.7293092479 1.5928013519

C 2.3446561006 4.2420289958 0.540868878

H 2.9036550975 4.5403993814 1.4416943872

C 2.5979496309 4.8757282878 -0.6903919671

H 3.3538671213 5.6741684838 -0.7564913674

C 1.8861181223 4.4806948952 -1.8356201147

H 2.0817899396 4.9670532952 -2.8046460822

C 0.913358512 3.4664947535 -1.7521347456

H 0.3628192934 3.1797905401 -2.6609973371

C -1.934073981 2.4871729297 0.6639450384

C -2.421681887 3.7196054655 0.168343214

H -2.0269835124 4.1337028776 -0.7736651637

C -3.40610651 4.430016563 0.8745531017

H -3.781636199 5.3857994059 0.4755912594

C -3.9072859461 3.9254393603 2.089822765

H -4.6767173361 4.4856233037 2.6445359618

C -3.4195702359 2.7081325296 2.5944954742

H -3.8023799745 2.3101154265 3.5476069394

C -2.4384900138 1.9910187262 1.8842758893

H -2.0589751906 1.0388681458 2.2849757884

C 0.2142525031 -0.6104527379 2.0777077826

O 0.5281896454 -0.4997180444 3.2032712171

C -2.0226777999 -1.1895145128 0.536248714

O -3.1156777876 -1.5646417208 0.694734135

----

fac-TSD-A

Frequencies, energies and thermodynamic properties:

Lowest Vibrational Mode (cm^{-1}) = -61.9033

2nd Lowest Vibrational Mode (cm^{-1}) = 21.6743

E(RB-P86) (h) = -2142.52202

Thermal correction to Enthalpy (h) = 0.530410

Thermal correction to Gibbs Free Energy (h) = 0.421104

Total Entropy (cal/Kmol) = 230.054

E(RPBE1PBE) (h) = -2142.28263

Optimised cartesian coordinates (Å):

Ru -0.110087 -1.278897 0.298953

H -1.345009 -2.302340 0.507385

P -1.877732 0.073038 0.467546

P 1.992235 -0.082150 0.504084

N -0.043142 -1.152088 2.353638

C -1.367095 -0.955720 2.969690

H -1.285411 -0.803274 4.072567

H -1.990735 -1.862160 2.813478

C -2.061885 0.269514 2.336700

H -1.515173 1.192589 2.623257

H -3.125080 0.398694 2.632258

C 0.995120 -0.394879 3.059911

H 1.138089 -0.811371 4.088965

H 0.707352 0.679956 3.217055

C 2.324981 -0.445186 2.305736

H 2.727344 -1.481373 2.310124

H 3.099401 0.214195 2.749318

C -3.553112 -0.501890 -0.116860

C -4.667234 0.356107 0.045777

H -4.536124 1.359413 0.483019

C -5.948780 -0.057077 -0.354081

H -6.807909 0.618664 -0.215759

C -6.132778 -1.325966 -0.935569

H -7.137152 -1.646622 -1.255206

C -5.029550 -2.178768 -1.110008

H -5.164258 -3.171219 -1.568943

C -3.746563 -1.770952 -0.698968

H -2.880618 -2.439541 -0.824041

C -1.866151 1.780618 -0.258794

C -1.934826 2.961584 0.511510

H -1.984604 2.914517 1.609989

C -1.954259 4.221096 -0.117995

H -2.012095 5.134003 0.495932

C -1.906162 4.314027 -1.518898

H -1.925933 5.300420 -2.008846

C -1.828456 3.140598 -2.293710

H -1.785204 3.204994 -3.392639

C -1.802883 1.884162 -1.669185

H -1.733831 0.971679 -2.283469

C 2.115170 1.753088 0.305770

C 1.855741 2.292940 -0.976332

H 1.575559 1.623229 -1.805615

C 1.954414 3.675056 -1.201236

H 1.759189 4.079309 -2.206908

C 2.291003 4.542062 -0.144215

H 2.362213 5.627169 -0.319934

C 2.531218 4.017227 1.136785

H 2.791688 4.688548 1.970666

C 2.448691 2.629911 1.361914

H 2.653393 2.240466 2.370655

C 3.527473 -0.685039 -0.344544

C 4.707132 0.089511 -0.421362

H 4.726058 1.109217 -0.004703

C 5.859123 -0.433953 -1.034025

H 6.773292 0.178506 -1.090715

C 5.845837 -1.733540 -1.574138

H 6.748993 -2.139892 -2.056776

C 4.674743 -2.509142 -1.501822

H 4.656020 -3.524931 -1.927835

C 3.519282 -1.986328 -0.894133

H 2.597029 -2.589744 -0.846014

C -0.149680 -1.503360 -1.553591

O -0.194465 -1.590879 -2.732747

C 0.634197 -4.640811 -0.158567

O -0.263844 -5.299079 -0.426714

----

fac-1Adim

Frequencies, energies and thermodynamic properties:

Lowest Vibrational Mode (cm^{-1}) = 7.9537

2nd Lowest Vibrational Mode (cm^{-1}) = 15.0510

E(RB-P86) (h) = -4058.611476

Thermal correction to Enthalpy (h) = 1.044755

Thermal correction to Gibbs Free Energy (h) = 0.871373

Total Entropy (cal/Kmol) = 364.912

E(RPBE1PBE) (h) = -4058.155798

Optimised cartesian coordinates (Å):

Ru 0.4739725175 1.169733648 0.6010355422

H -1.1307051289 0.3523852904 0.3545052722

P -0.5367225247 3.1530443124 1.3275071603

P 2.6580424779 1.9182854594 0.3264957227

N 0.2399519629 2.1267856285 -1.3443038993

C -0.9150878292 3.0036181473 -1.3678334248

H -1.0185594941 3.5238906015 -2.3541936795

H -1.8467621738 2.4071063881 -1.2354510488

C -0.8589953128 4.0847725933 -0.2504470318

H 0.0077732316 4.7576388251 -0.4247440935

H -1.7716104962 4.7135545823 -0.1824568396

C 1.393641859 2.7859953038 -1.9401319062

H 1.330424879 2.7800990138 -3.0620165775

H 1.4758115106 3.8803163301 -1.672825902

C 2.704848382 2.1154123878 -1.5232011908

H 2.7604028417 1.0788835188 -1.9162492402

H 3.5998228974 2.665731777 -1.8796259336

C -2.2289521332 2.9605358875 2.0993146481

C -3.055248268 4.0972539681 2.2662860751

H -2.707783926 5.0900838527 1.9367917329

C -4.3192077781 3.9799796806 2.8675607096

H -4.9526134421 4.8737997988 2.9855125517

C -4.7702220466 2.7271759491 3.3247901561

H -5.7594216754 2.6357169659 3.8010211031

C -3.9512768685 1.5958995917 3.1732005628

H -4.2960914776 0.6125293458 3.530353621

C -2.6892927565 1.7102237603 2.559720671

H -2.0547856008 0.8209526853 2.4275329888

C 0.1707118649 4.3645090389 2.5417169054

C 0.326443867 5.7387495847 2.2565022209

H 0.086643112 6.1334577364 1.2573889484

C 0.776041306 6.6259513582 3.2527317583

H 0.8886084489 7.6961718116 3.0163653649

C 1.072787948 6.1524314398 4.54217959

H 1.4174756255 6.8499278554 5.3220957365

C 0.9294907335 4.7822985849 4.8310609181

H 1.1650821088 4.401718331 5.8377759697

C 0.486763109 3.8937177625 3.8382449934

H 0.3797915066 2.8230706392 4.0755648708

C 3.3948382805 3.4780657626 1.0234958863

C 3.7156381753 3.512527927 2.4004441159

H 3.5012252969 2.6393741642 3.0374862838

C 4.3236782802 4.6451584515 2.9647722015

H 4.5752085039 4.6515157229 4.0370653152

C 4.6096026069 5.7675260871 2.1650822544

H 5.0884687068 6.6553895026 2.607599509

C 4.278479288 5.7498115805 0.7995712249

H 4.4958217337 6.6242881479 0.1654091711

C 3.6753820443 4.6127353286 0.2300873469

H 3.4307412345 4.6201810943 -0.843031744

C 4.0461588921 0.7393171091 0.7064772865

C 5.2325405257 0.7753586536 -0.0635015532

H 5.3288060528 1.4751308963 -0.9081091712

C 6.3082852403 -0.075162186 0.2429446827

H 7.2231737754 -0.0382094105 -0.3697719226

C 6.2196425519 -0.9658375896 1.3285865447

H 7.0650771871 -1.6295978056 1.5700395123

C 5.0470847108 -1.0040027949 2.1021228057

H 4.9676451012 -1.6980266238 2.9539555584

C 3.964015972 -0.1607126511 1.7909060585

H 3.0502748479 -0.2054919359 2.400099226

C 0.640989307 0.4770419013 2.3189672853

O 0.7700372172 0.2095465523 3.463091325

Ru -0.5965556557 -1.1668975072 -0.4807922366

H 1.0009967509 -0.3370094282 -0.2496934766

P 0.5766089298 -3.0381996112 -1.2631224794

P -2.7129835582 -2.0789036862 -0.1852043631

N -0.2398810325 -2.1428913589 1.4302660043

C 1.0295465122 -2.8481479446 1.4252402482

H 1.2167757146 -3.3653237322 2.4015773768

H 1.8619578653 -2.1220167188 1.2882495542

C 1.1046249597 -3.9127006786 0.2937477113

H 0.3612624513 -4.7166241201 0.4809122192

H 2.0992853559 -4.3949155885 0.1973966415

C -1.2892411807 -2.9880823223 1.9831936367

H -1.1946521269 -3.0638662251 3.0998821203

H -1.2466851921 -4.0594575325 1.6274244089

C -2.678592204 -2.4479143616 1.6437786767

H -2.8454490106 -1.467421745 2.1349258455

H -3.5022724815 -3.1257244079 1.9497598265

C 2.1634363873 -2.6408730386 -2.1696779982

C 3.2506392662 -3.5447203588 -2.1532860887

H 3.198613882 -4.4721821541 -1.5614496031

C 4.4110444362 -3.2835107807 -2.9020776734

H 5.2507975903 -3.9960468798 -2.870475337

C 4.4966797568 -2.1234849773 -3.6930496467

H 5.4038099632 -1.922280441 -4.2848516972

C 3.4156659445 -1.2257872046 -3.7262486833

H 3.46960179 -0.3174599732 -4.3473398968

C 2.2594024235 -1.4788123269 -2.9651104599

H 1.421393007 -0.7676127563 -2.9927637923

C -0.0520971441 -4.3407575402 -2.4276475251

C 0.0985301106 -5.7237580619 -2.1836246965

H 0.5373999693 -6.0807198612 -1.2392037352

C -0.297985037 -6.6639976817 -3.1526326971

H -0.1734530406 -7.7397095625 -2.9491581487

C -0.8404115286 -6.2348569807 -4.3763258124

H -1.1420614432 -6.9725398171 -5.1368342414

C -1.0004783463 -4.858723831 -4.6228964591

H -1.4322607191 -4.5141560417 -5.5760116362

C -0.6161095716 -3.9180533616 -3.6535511955

H -0.7513316483 -2.8429682231 -3.853440575

C -3.3678070517 -3.6133027441 -0.9998032144

C -3.8824558546 -3.5151380021 -2.3136751262

H -3.8844655582 -2.5424926416 -2.8320362599

C -4.4113881339 -4.6437229437 -2.959904397

H -4.8167646845 -4.5482158991 -3.9797487234

C -4.4244661263 -5.8912625435 -2.3084910208

H -4.8412868112 -6.7762117568 -2.8149721644

C -3.9013989044 -6.0018953937 -1.0087205924

H -3.9061430188 -6.9743667957 -0.4908472481

C -3.3760560351 -4.8708849597 -0.3568683511

H -2.9738707185 -4.979302294 0.6623521641

C -4.1924811856 -0.9651223167 -0.3731288972

C -5.4784799011 -1.4511141132 -0.0356030847

H -5.6064505643 -2.4934834345 0.2983982399

C -6.6029573343 -0.614939695 -0.1316122663

H -7.5977223436 -1.00472783 0.1376303973

C -6.4600814162 0.7134465749 -0.5759861114

H -7.3435497354 1.3666367726 -0.6572220234

C -5.1872084191 1.2008334958 -0.9169380134

H -5.0663490402 2.2385776435 -1.2659667463

C -4.0582300555 0.3671956968 -0.8110981752

H -3.0596944305 0.7531077823 -1.0648522452

C -0.9347226883 -0.4401915556 -2.1608695887

O -1.2136024943 -0.1349078838 -3.267621537

----

fac-1B'

Frequencies, energies and thermodynamic properties:

Lowest Vibrational Mode (cm^{-1}) = 4.8482

2nd Lowest Vibrational Mode (cm^{-1}) = 28.8466

E(RB-P86) (h) = -2430.157724

Thermal correction to Enthalpy (h) = 0.673140

Thermal correction to Gibbs Free Energy (h) = 0.557749

Total Entropy (cal/Kmol) = 242.860

E(RPBE1PBE) (h) = -2429.93022159

Optimised cartesian coordinates (Å):

Ru 0.5322713891 -0.2617304439 0.817742769

H 1.7695120638 0.741718661 1.1294277244

P -0.1098080644 1.7635730269 -0.0872353454

P -1.0549162298 -1.8567635196 -0.0500382508

N 1.5015892635 -0.6036097766 -1.0757397611

C 1.9636003868 0.6895232671 -1.6535533611

H 2.3934865909 0.52988099 -2.6702768831

H 2.7772052688 1.0739330578 -1.0058842474

C 0.8200398384 1.7134437972 -1.7289468872

H 0.0969582629 1.4286558958 -2.5198988375

H 1.1999922302 2.7220480571 -1.991894027

C 0.7579055321 -1.3762300513 -2.1127707634

H 1.5058840772 -1.8260172187 -2.8023598678

H 0.1331517056 -0.666211128 -2.6926591577

C -0.1184168818 -2.4883992757 -1.5389803993

H 0.5367154872 -3.3076848464 -1.1817788141

H -0.7728414306 -2.9143850523 -2.3262301724

C 0.5306744775 3.319631169 0.7243068144

C 0.3658137216 4.5616939914 0.0654354297

H -0.1553931536 4.6092752513 -0.9047495873

C 0.8545892559 5.746446238 0.6399889633

H 0.725220323 6.7038343907 0.11038515

C 1.5011660546 5.7109969183 1.8901710508

H 1.8794105119 6.6411416199 2.3434506923

C 1.6588496895 4.4842990226 2.5568459579

H 2.1593966865 4.4474741422 3.5375753139

C 1.1813761075 3.2944725497 1.9748497219

H 1.3214604738 2.3318940607 2.488701601

C -1.8251390393 2.3517968579 -0.5093297271

C -2.3928291044 2.285160843 -1.7998091986

H -1.8214395851 1.8773208956 -2.6473217678

C -3.7008726981 2.751140883 -2.0302538926

H -4.1227032755 2.7007220764 -3.0466311319

C -4.4622169743 3.2813056656 -0.9754267578

H -5.4835864454 3.6502529402 -1.1598393197

C -3.9124360043 3.3343014072 0.3191187041

H -4.5020297799 3.7431519512 1.1552295003

C -2.6077611881 2.8714182039 0.5501192635

H -2.1865838147 2.9278883958 1.5673006462

C -2.7355894846 -1.349895858 -0.6680193842

C -3.5822325802 -0.6715356934 0.2388023187

H -3.2054872975 -0.3962986051 1.2373333216

C -4.9013447268 -0.3502033747 -0.1188270442

H -5.5505442349 0.1700681583 0.6027393279

C -5.3887103089 -0.6821732329 -1.3964798569

H -6.4227361319 -0.4275894903 -1.6782767029

C -4.5466911504 -1.3335525412 -2.313814766

H -4.9164431307 -1.5906877003 -3.3194525543

C -3.2288551963 -1.6698914762 -1.951625125

H -2.5953018176 -2.1935422069 -2.6840061637

C -1.5509019196 -3.4048877844 0.8609962324

C -1.7155481711 -3.3519505592 2.2629826486

H -1.5101678958 -2.4149683325 2.803913195

C -2.1336491871 -4.4900883433 2.9761360728

H -2.2584587247 -4.43336327 4.0692381069

C -2.3816538425 -5.6969018929 2.2982979506

H -2.7025194361 -6.5901950079 2.8573821318

C -2.2167920037 -5.7592133713 0.9023876629

H -2.409223338 -6.7012102188 0.3642716789

C -1.8100267684 -4.6196301495 0.1861216674

H -1.6996243387 -4.6866850383 -0.907647573

C -0.1575416497 -0.0361517097 2.5158342627

O -0.5978134319 0.1063508396 3.6029873445

H 3.5403841821 -0.7249237194 -0.3048022914

C 2.74649542 -1.5179645697 -0.4634476236

O 2.9994051413 -2.5659058589 -1.1113945637

C 3.1757671079 -1.2536510806 2.0825232679

C 1.8435297904 -3.1340722154 1.3848571372

C 4.3617738472 -2.2109687415 2.2421390806

H 2.6566589466 -1.1509530578 3.0589976257

H 3.5306844754 -0.2455482965 1.791280863

C 3.0720745318 -4.0306182868 1.6011821818

H 1.2182064105 -3.1336980671 2.3021527244

H 1.2377023881 -3.5597403956 0.5655752297

H 5.0295311932 -1.8384057339 3.0471737431

H 4.9568452005 -2.2534205875 1.2955692944

H 2.7390595362 -5.0352765639 1.9361993012

H 3.6218419665 -4.1310329913 0.6376977948

N 2.1733973332 -1.7156367997 1.0732051725

O 3.9344270575 -3.5163665593 2.616046335

----

fac-1B-4MR

Frequencies, energies and thermodynamic properties:

Lowest Vibrational Mode (cm^{-1}) = 17.1338

2nd Lowest Vibrational Mode (cm^{-1}) = 19.8820

E(RB-P86) (h) = -2430.161776

Thermal correction to Enthalpy (h) = 0.672874

Thermal correction to Gibbs Free Energy (h) = 0.556417

Total Entropy (cal/Kmol) = 245.105

E(RPBE1PBE) (h) = -2429.9265006

Optimised cartesian coordinates (Å):

Ru -0.0485498824 -0.7309922229 -0.0733648512

H 0.1185176833 -2.345146453 -0.0685056069

P 2.1612151003 -0.9868744264 -0.5540748131

P -0.6657906358 1.578174133 -0.4480545518

N -0.3744982652 -0.9037899487 -2.2098386999

C 0.653018123 -1.790306205 -2.8046726769

H 0.5654157068 -1.8161319576 -3.9166549261

H 0.4622804824 -2.8141466226 -2.4301865638

C 2.0687548843 -1.3272815148 -2.4119931201

H 2.3255248109 -0.3874643464 -2.9416032944

H 2.8305296858 -2.0783045992 -2.7054021431

C -0.4954408188 0.3749661629 -2.9629955631

H -0.9736745271 0.1737958563 -3.9525448568

H 0.5240078243 0.7615355841 -3.1740044006

C -1.2978085748 1.4377637955 -2.207527096

H -2.3618016423 1.1419673871 -2.1109624239

H -1.2832349804 2.4039772757 -2.7509573604

C 3.070207916 -2.4748477089 0.1186181614

C 4.3923369464 -2.7409011689 -0.3112204082

H 4.8925667493 -2.0591857852 -1.0185689628

C 5.0847011038 -3.8673020927 0.1631262479

H 6.1109134718 -4.0646997431 -0.185969235

C 4.4720967997 -4.7362328196 1.086205671

H 5.0176675043 -5.6161702047 1.4628668765

C 3.16358834 -4.4738441398 1.5260589775

H 2.6780022219 -5.146289351 2.2512095156

C 2.4641661746 -3.3523628231 1.0410294306

H 1.4324892214 -3.1548575851 1.3697125474

C 3.5067634697 0.2856053454 -0.3512207963

C 4.0381862681 1.0650757553 -1.4013190173

H 3.6805291224 0.939595279 -2.434519687

C 5.0482470331 2.013138278 -1.1488378908

H 5.4567726858 2.6052992787 -1.983224236

C 5.5367505695 2.1995997784 0.1549582792

H 6.3304307726 2.9382671076 0.349292516

C 5.0038295579 1.4370795551 1.2117671902

H 5.3768798461 1.5774014023 2.2388954977

C 3.9968717734 0.4921673275 0.9613159506

H 3.5921098176 -0.1011999734 1.7978269209

C 0.5352827866 2.9862370777 -0.4188924291

C 1.3968078601 3.0738879759 0.6992050656

H 1.3658771648 2.287512547 1.470448782

C 2.2876856496 4.151794116 0.8331316525

H 2.9456396078 4.2097345988 1.7141808443

C 2.3492013746 5.1443791587 -0.1621195322

H 3.0535844335 5.985473779 -0.0622597255

C 1.5108086712 5.056237156 -1.2875045706

H 1.5551642985 5.8280034033 -2.0725945299

C 0.6035790592 3.9881506165 -1.4126659647

H -0.058410739 3.9527329232 -2.2917314491

C -2.1295111543 2.2855592247 0.4556470562

C -2.1483479424 3.5912596936 0.9936686785

H -1.2775864484 4.2535720494 0.8718901434

C -3.2801556315 4.0556924004 1.6897117467

H -3.2818668688 5.0766492678 2.1044559203

C -4.4020871754 3.2249348874 1.8540218676

H -5.2869709852 3.5914208144 2.3986975271

C -4.3867652824 1.9213429944 1.3223908299

H -5.2608424612 1.2626846177 1.4519473937

C -3.2580310592 1.4473045879 0.632417221

H -3.2313009953 0.4224777701 0.2205841417

C 0.0979312151 -0.6595987833 1.7635520581

O 0.2076530179 -0.604649259 2.9381821114

H -2.5069487383 -0.9778267446 -2.6855925689

C -1.8797171381 -1.5333820006 -1.9298993982

O -2.1042439332 -1.2071905309 -0.6392560939

C -2.2583908729 -3.3631310348 -3.609181162

C -1.8305430175 -3.976700247 -1.2680579232

C -3.3852033358 -4.4080687972 -3.6234825364

H -1.3442954167 -3.8136096067 -4.074019866

H -2.5478732586 -2.4905411344 -4.2344297104

C -2.9810695984 -4.9827506159 -1.3810776096

H -0.8627942212 -4.5209348941 -1.4174815188

H -1.8135294868 -3.5128863798 -0.2627854019

H -3.4903418242 -4.8599487071 -4.6320080546

H -4.3512787976 -3.9097291629 -3.3551020239

H -2.7977319419 -5.8655927733 -0.7339190696

H -3.9342960362 -4.4942604911 -1.056480834

N -2.0074398958 -2.9164932767 -2.2487843297

O -3.1171098424 -5.4755728403 -2.7172962489

----

fac-1X

Frequencies, energies and thermodynamic properties:

Lowest Vibrational Mode (cm^{-1}) = 7.3450

2nd Lowest Vibrational Mode (cm^{-1}) = 20.2849

E(RB-P86) (h) = -2142.5664486

Thermal correction to Enthalpy (h) = 0.532957

Thermal correction to Gibbs Free Energy (h) = 0.427310

Total Entropy (cal/Kmol) = 222.353

E(RPBE1PBE) (h) = -2142.3204778

Optimised cartesian coordinates (Å):

Ru -0.0222070455 -1.3535676963 0.1237047417

H -1.2978735237 -2.3805320165 0.149524996

P -1.9676856384 0.0387063934 0.3276158653

P 1.9281333394 0.0066230082 0.434566341

N -0.0327011504 -1.389245321 2.2530775675

C -1.4208761547 -1.3562829225 2.7580634769

H -1.4233594838 -1.3317289746 3.8719683326

H -1.9239689405 -2.2938372889 2.4456077649

C -2.1725386897 -0.1266765771 2.207509914

H -1.7613305529 0.7955328061 2.6664706725

H -3.2490668099 -0.1647172492 2.4726239575

C 0.9353678634 -0.5557539521 3.0049170275

H 1.1330308425 -1.0233003403 3.9982959926

H 0.4690766989 0.4358363757 3.1977006397

C 2.2529517262 -0.367369346 2.2492074594

H 2.8360705768 -1.311463044 2.2347966083

H 2.8901845022 0.3931385741 2.7438457674

C -3.588153017 -0.5882850225 -0.3457060381

C -4.8113243488 -0.0153701321 0.0761043662

H -4.8141890622 0.8147218567 0.801588078

C -6.0326878785 -0.4894706234 -0.430734453

H -6.978040159 -0.0386450525 -0.0887088625

C -6.0480348007 -1.5329598013 -1.3759966604

H -7.0061469515 -1.9019132293 -1.7755283253

C -4.8369589137 -2.0989410595 -1.8095545758

H -4.8403528128 -2.9121994505 -2.5526977604

C -3.613083414 -1.6313614077 -1.2945517761

H -2.6644243279 -2.08189047 -1.6245850943

C -2.1807372054 1.8601017731 -0.0053143419

C -1.9836334015 2.8770856774 0.9548375957

H -1.731153535 2.6284373809 1.9968020666

C -2.1143824934 4.2332723611 0.6004714253

H -1.9665477279 5.009826967 1.3678603955

C -2.4346063339 4.5960563047 -0.7184410395

H -2.5399637544 5.657718723 -0.9923209033

C -2.6165498379 3.591342095 -1.6878789117

H -2.8634908199 3.8624538938 -2.726861355

C -2.4873072387 2.2388642587 -1.3358347418

H -2.6359637585 1.4636805469 -2.1056017675

C 1.92775809 1.8499797084 0.2571510931

C 1.5180126861 2.3764736814 -0.9909367437

H 1.190601805 1.6926048393 -1.791344907

C 1.5259285686 3.7617982138 -1.2188568847

H 1.2137877873 4.1554709394 -2.1989303706

C 1.9190519282 4.644412511 -0.195157169

H 1.9182908764 5.731674622 -0.3716353959

C 2.3065202233 4.1326422703 1.0549901979

H 2.6109081204 4.8166604875 1.863229057

C 2.3164904147 2.7430987004 1.2799215816

H 2.6392691091 2.3661657523 2.2625587894

C 3.5369445236 -0.4782506175 -0.3576491551

C 4.575532734 0.4484936064 -0.5988259056

H 4.4398931635 1.5103829943 -0.3410256235

C 5.7870512345 0.0219553169 -1.1723042011

H 6.5887363837 0.7544929016 -1.3584170071

C 5.975038693 -1.3309004409 -1.5073748624

H 6.9238844054 -1.661885821 -1.9588179658

C 4.9443346766 -2.2586640284 -1.2695368882

H 5.0819360075 -3.3190914868 -1.5345135879

C 3.7294413902 -1.8353333687 -0.7037020676

H 2.9156534527 -2.5578435172 -0.5297746424

C 0.2435162513 -1.8791297465 -1.6378129339

O 0.4255143743 -2.2467494775 -2.7431333182

C 0.5510325673 -2.6384033486 1.4560226493

O 0.8755572159 -3.6896878303 1.9481752862

----

7. References

(1) Liu, X.; Werner, T. "Selective Construction of C−C and C=C Bonds by Manganese Catalyzed Coupling of Alcohols with Phosphorus Ylides." *Adv. Synth. Catal.* (2021), *363* (4), 1096–1104. https://doi.org/10.1002/adsc.202001209.

(2) Han, Z.; Rong, L.; Wu, J.; Zhang, L.; Wang, Z.; Ding, K. "Catalytic Hydrogenation of Cyclic Carbonates: A Practical Approach from CO_2 and Epoxides to Methanol and Diols." *Angew. Chem. Int. Ed.* (2012), *51* (52), 13041–13045. https://doi.org/10.1002/anie.201207781.

(3) Zhang, L.; Han, Z.; Zhao, X.; Wang, Z.; Ding, K. "Highly Efficient Ruthenium-Catalyzed N-Formylation of Amines with H_2 and CO_2." *Angew. Chem. Int. Ed.* (2015), *54* (21), 6186–6189. https://doi.org/10.1002/anie.201500939.

(4) Nguyen, T. V. Q.; Yoo, W.; Kobayashi, S. “Effective Formylation of Amines with Carbon Dioxide and Diphenylsilane Catalyzed by Chelating Bis(*Tz* NHC) Rhodium Complexes.” *Angew. Chem. Int. Ed.* (2015), *54* (32), 9209–9212. https://doi.org/10.1002/anie.201504072.

(5) Cui, X.; Zhang, Y.; Deng, Y.; Shi, F. “Amine Formylation via Carbon Dioxide Recycling Catalyzed by a Simple and Efficient Heterogeneous Palladium Catalyst.” *Chem. Commun.* (2014), *50* (2), 189–191. https://doi.org/10.1039/C3CC46427J.

(6) Kumar, A.; Sharma, P.; Sharma, N.; Kumar, Y.; Mahajan, D. “Catalyst Free *N* -Formylation of Aromatic and Aliphatic Amines Exploiting Reductive Formylation of CO_2 Using $NaBH_4$.” *RSC Adv.* (2021), *11* (41), 25777–25787. https://doi.org/10.1039/D1RA04848A.

(7) CrysAlisPro v1.171.44.123a Rigaku Oxford Diffraction, Rigaku Corporation, Tokyo, Japan, 2025.

(8) Sheldrick, G. M. “SHELXT – Integrated space-group and crystal structure determination.” *Acta Crystallogr., Sect. A: Found. Adv.* (2015), 71, 3-8. doi: 10.1107/S2053273314026370

(9) Sheldrick, G. M. “Crystal structure refinement with SHELXL.” *Acta Crystallogr., Sect. C: Struct. Chem.* (2015), 71, 3-8. Doi: 10.1107/S2053229614024218

(10) Dolomanov, O. V.; Bourhis, L. J.; Gildea, R. J.; Howard, J. A. K.; Puschmann, H. “OLEX2: a complete structure solution, refinement and analysis program.” *J. Appl. Crystallogr.* (2009), 42, 339-341. doi: 10.1107/S0021889808042726

(11) M. J. Frisch, G. W. Trucks, H. B. Schlegel, G. E. Scuseria, M. A. Robb, J. R. Cheeseman, G. Scalmani, V. Barone, G. A. Petersson, H. Nakatsuji, X. Li, M. Caricato, A. V. Marenich, J. Bloino, B. G. Janesko, R. Gomperts, B. Mennucci, H. P. Hratchian, J. V. Ortiz, A. F. Izmaylov, J. L. Sonnenberg, D. Williams-Young, F. Ding, F. Lipparini, F. Egidi, J. Goings, B. Peng, A. Petrone, T. Henderson, D. Ranasinghe, V. G. Zakrzewski, J. Gao, N. Rega, G. Zheng, W. Liang, M. Hada, M. Ehara, K. Toyota, R. Fukuda, J. Hasegawa, M. Ishida, T. Nakajima, Y. Honda, O. Kitao, H. Nakai, T. Vreven, K. Throssell, J. A. Montgomery, Jr., J. E. Peralta, F. Ogliaro, M. J. Bearpark, J. J. Heyd, E. N. Brothers, K. N. Kudin, V. N. Staroverov, T. A. Keith, R. Kobayashi, J. Normand, K. Raghavachari, A. P. Rendell, J. C. Burant, S. S. Iyengar, J. Tomasi, M. Cossi, J. M. Millam, M. Klene, C. Adamo, R. Cammi, J. W. Ochterski, R. L. Martin, K. Morokuma, O. Farkas, J. B. Foresman and D. J. Fox, Gaussian 16, Revision C.01, Gaussian Inc., Wallingford CT, 2019.

(12) Brodie, C. N.; Owen, A. E.; Kolb, J. S.; Bühl, M.; Kumar, A. “Synthesis of Polyethyleneimines from the Manganese-Catalysed Coupling of Ethylene Glycol and Ethylenediamine” *Angew. Chem. Int. Ed.* (2023), 62, e202306655. https://doi.org/10.1002/anie.202306655

(13) Owen, A. E.; Preiss, A.; McLuskie, A.; Gao, C.; Peters, G.; Bühl, M.; Kumar, A. ”Manganese catalysed dehydrogenative synthesis of polyureas from diformamide and diamines” *ACS Catal.* (2022), 12, 6923-6933. https://doi.org/10.1039/D3CY00284E.

(14) Owen A. E.; Preiss A.; McLuskie, A.; Gao, C.; Peters, G.; Bühl, M.; Kumar, A. "Correction to "Manganese Catalyzed Dehydrogenative Synthesis of Urea Derivatives and Polyureas"" *ACS Catal.* (2023), 13, 10796-10797. https://doi.org/10.1021/acscatal.3c02871.

(15) Oates, C. L.; Goodfellow, A. S.; Bühl M.; Clarke, M.; *Angew.* "Rational Design of a Facially Coordinating P,N,N Ligand for Manganese-Catalysed Enantioselective Hydrogenation of Cyclic Ketones." *Angew. Chem. Int. Ed.* (2023), 62, e202212479. https://doi.org/10.1002/anie.202212479.

(16) Oates, C. L.; Goodfellow, A. S.; Bühl M.; Clarke, M.; "Manganese catalysed enantioselective hydrogenation of in situ-synthesised imines: efficient asymmetric synthesis of amino-indane derivative." *Green Chem.* (2023), 25, 3864-3868. https://doi.org/10.1039/d3gc00399j.

(17) Goodfellow A. S.; Bühl, M.; "Hydricity of 3d Transition Metal Complexes from Density Functional Theory: A Benchmarking Study" *Molecules* (2021), 26, 4072. https://doi.org/10.3390/molecules26134072

(18) Martin, R. L.; Hays P. J.; Pratt, L. R.; "Hydrolysis of Ferric Ion in Water and Conformational Equilibrium" *J. Phys. Chem. A* (1998), 102, 3565-3573. https://doi.org/10.1021/jp980229p

(19) Andrae, D.; Haeussermann, U.; Dolg, M.; Stoll, H.; Preuss H.; "Energy-Adjusted ab initio Pseudopotentials for the Second and Third Row Transition Elements." Theor. Chem. Acc., 1990, 17, 123-41. https://doi.org/10.1007/BF01114537
